# Photoredox radical conjugate addition of dithiane-2-carboxylate promoted by an iridium(iii) phenyl-tetrazole complex: a formal radical methylation of Michael acceptors†
†Electronic supplementary information (ESI) available: Experimental procedures, product characterizations, NMR spectra, compound preparation procedures, screening tests, and photophysical and electrochemical data. See DOI: 10.1039/c6sc03374a



**DOI:** 10.1039/c6sc03374a

**Published:** 2016-11-03

**Authors:** Andrea Gualandi, Elia Matteucci, Filippo Monti, Andrea Baschieri, Nicola Armaroli, Letizia Sambri, Pier Giorgio Cozzi

**Affiliations:** a Dipartimento di Chimica “G. Ciamician” , ALMA MATER STUDIORUM Università di Bologna , Via Selmi 2 , 40126 Bologna , Italy . Email: piergiorgio.cozzi@unibo.it; b Dipartimento di Chimica Industriale “Toso Montanari” , ALMA MATER STUDIORUM Università di Bologna , Viale Risorgimento 4 , 40136 , Bologna , Italy . Email: letizia.sambri@unibo.it; c Istituto per la Sintesi Organica e la Fotoreattività , Consiglio Nazionale delle Ricerche , Via P. Gobetti 101 , 40129 Bologna , Italy . Email: nicola.armaroli@isof.cnr.it

## Abstract

A readily accessible iridium(iii) phenyl-tetrazole complex ([Ir(ptrz)_2_(*t*Bu-bpy)]^+^, **2**; Hptrz = 2-methyl-5-phenyl-tetrazole; *t*Bu-bpy = 4,4′-di-*tert*-butyl-2,2′-bipyridine) is shown to be a versatile catalyst for a new photocatalytic Michael reaction. Under light irradiation in the presence of **2**, a dithiane 2-carboxylic acid, obtained by simple hydrolysis of a commercially available ethyl ester, generates a 1,3-dithiane radical capable of performing addition to a variety of Michael acceptors (*e.g.*, unsaturated ketones, esters, amides and malonates). This broad scope reaction with high yields is a formal photo-redox addition of the elusive methyl radical and the adducts obtained can be starting materials for a variety of functionalized products. The excited-state oxidation potential of catalyst **2** allows selective formation of radicals only from α-heterosubstituted carboxylates. Chemical modification of this metal complex can tune the electrochemical properties, opening a route to new highly selective catalytic photo-oxidation reactions.

## Abstract 2

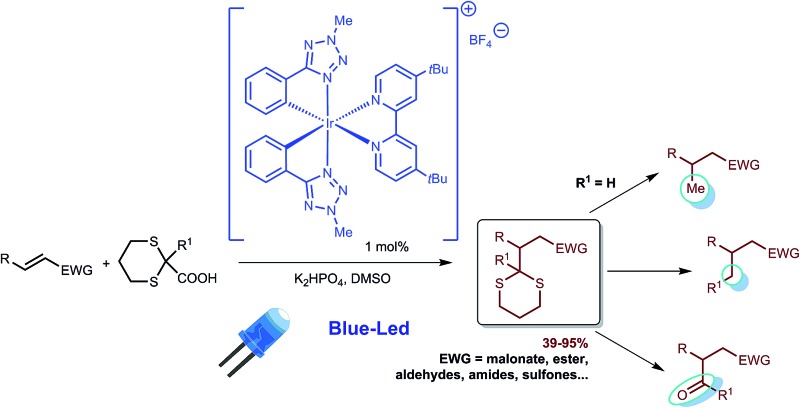
An iridium(iii) phenyl-tetrazole complex is a versatile catalyst for a new photocatalytic Michael reaction.

## Introduction

**Fig. 1:**
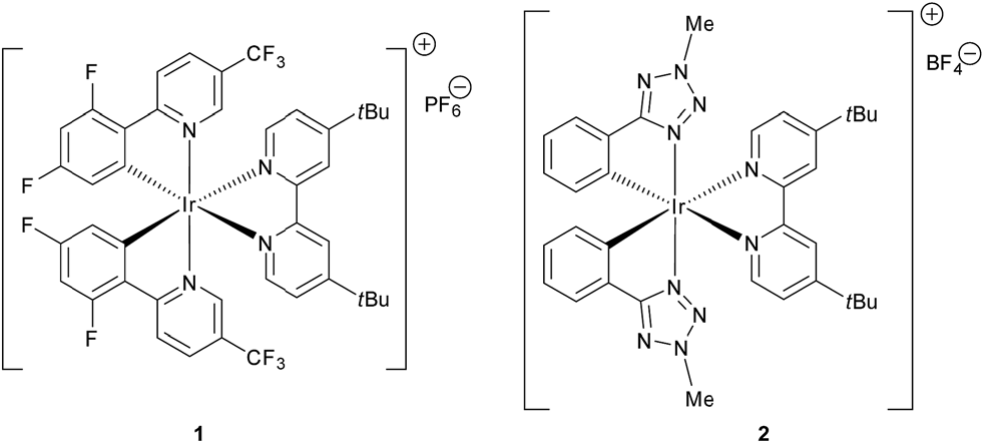
Structures of the iridium complexes **1** and **2**.

Photoredox catalysis has recently emerged as a mild and efficient method for the generation of radicals, as it is possible to take advantage of the photophysical properties^1^ of suitable organic dyes^2^ and transition metal complexes exhibiting tailored electrochemical and photophysical properties.^3,4^ In particular, MacMillan *et al.* investigated 1,4-conjugate addition (Michael reaction)^5^ of radicals^6^ in connection with a series of electrophilic olefins. In these reactions a photoredox-mediated CO_2_-extrusion mechanism is operative and a broad array of Michael acceptors have been used. Simple or α-functionalized (N; O) carboxylic acids are employed as Michael donors without the need for organometallic mediated activation or propagation. Recently, Overman *et al.* have also explored *N*-phthalimidoyl oxalate derivatives of tertiary alcohols for reductive coupling of tertiary radicals with Michael acceptors, using visible light and [Ru(bpy)_3_][PF_6_]_2_.^7^ The key relevant characteristics of both the Overman and MacMillan radical generation methodologies were recently merged in a new powerful protocol.^8^ A typical procedure entails the addition of 1–2 mol% of the photocatalyst Ir[dF(CF_3_)ppy]_2_(*t*Bu-bpy)[PF_6_] (**1**, Fig. 1), which acts as a strong oxidant in the first step of the photocatalytic cycle.

Although these methodologies are extremely effective and use cheap and abundant starting materials, there are still some inherent limitations. Primarily, the commercially available iridium photocatalyst **1** is expensive and a Suzuki coupling is necessary for its preparation.^9^ Furthermore, the generation of primary radicals under photocatalytic conditions after extrusion of CO_2_ is challenging, and just one example where primary radicals are intercepted by fluorinating agents is reported in the literature.^10^ However, to the best of our knowledge, the generation and reaction of primary radicals is still an open issue in photocatalytic Michael reactions. Furthermore, addition of methyl radicals, recently reported using an ambitious methodology based on a Minisci type reaction,^11,12^ has still not been developed in the area of photocatalysis. In the above described context, the present study has two main goals: (i) to demonstrate the use of an alternative, simply prepared and tunable iridium(iii) photocatalyst (**2**, Fig. 1) for radical reactions, and (ii) to propose a new methodology for effective photocatalytic methylation.

Notably, Baran has recently shown that zinc bis[(phenylsulfonyl)methanesulfinate] can be effectively used for introducing a methyl group through a two-step synthetic procedure, in which a radical mediated process is involved in the first step.^13^ As a possible synthetic equivalent for a methyl group, we propose herein the chameleonic dithiane group, opening a route to the use of dithianecarboxylate as a Michael donor in photoredox catalysis. A secondary radical is formed, which is stabilized by the presence of two sulfur atoms, and can be used as a versatile synthetic equivalent. Remarkably, the dithiane can be not only replaced by a methyl group by treatment with RANEY®-nickel, but it is also possible to take advantage of its flexibility, allowing the installation of different functional groups such as aldehydes and ketones, upon the radical reaction.

## Optimization of the photocatalytic reaction and scope

Dithianes, introduced by Corey and Seebach^14^ are widely used in the synthesis of natural products.^15^ For instance, they were exploited by Smith for the application of linchpins in the synthesis of complex natural molecules.^16^ However, the radical reactivity of dithianes has been only rarely reported in the literature, mainly through the installation of a radical initiator group (phenylseleno, xanthate, TEMPO or chloro) at the 2-position for the generation of C-2 centred radicals.^17^ Direct radical addition of 1,3-dithianes to alkenes was shown to occur in an intramolecular fashion. Quite interestingly, Nishida and co-workers reported an intramolecular photocatalytic addition for the 1,3-dithiane, and this reaction has also been reported with other radical initiators.^18^ Recently, Leow and co-workers reported the photocatalytic addition of 1,3-benzodithioles to several Michael acceptors.^19^ However, only aryl or alkyl C2-substituted benzodithiole could be used, while aryl dithiane was found to be completely unreactive. On the other hand, Koike and Akita reported the reaction of potassium 1,3-dithian-2-yl trifluoroborate with terminal olefins bearing electron withdrawing groups^20^ which act as a synthetic equivalent of carbonyl groups.

**Scheme 1:**
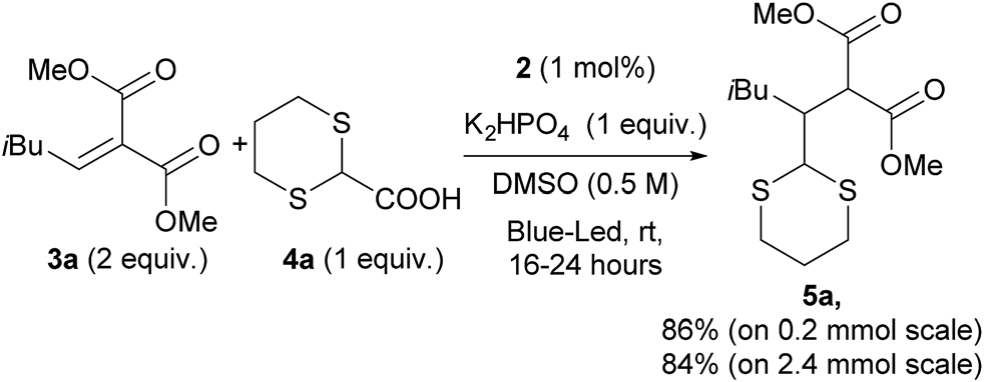
Optimized reaction conditions.

Based on the previous work by MacMillan^5^ and on the basis of the recent report on the practical use of commercially available dithiane carboxylate in organocatalytic reactions,^21^ we used this cheap and commercially available starting material as a suitable reagent for installing various unsubstituted or substituted dithianes by photocatalytic reactions. Accordingly, we have carefully investigated the addition of dithiane carboxylate (**4a**) to alkylidene malonate (**3a**) (Scheme 1) in the presence of different photocatalysts, by varying the solvent and other reaction conditions (see ESI for all complexes tested and full details†). Although the commercially available iridium catalyst **1** was found to be effective, we have investigated the catalytic activity of alternative iridium complexes, which can be more easily prepared, such as phenyl-tetrazole and pyridyl-triazole derivatives (see ESI†), without any Suzuki coupling. Only complexes containing phenyl tetrazoles as cyclometalating ligands showed an efficiency comparable to catalyst **1**. These compounds were recently reported by some of us^22^ as emitting materials in electroluminescent devices and here we show that they can also be successfully utilized to promote photocatalytic reactions.

Notably, by careful tuning of the pristine ligand structure, this class of complexes may provide remarkable opportunities to enhance reaction selectivity (*vide infra*). The photoredox radical conjugate addition of dithiane-2-carboxylate has been used to test our approach, giving full conversions and high isolated yields when the reaction is conducted in DMSO and in the presence of K_2_HPO_4_ as an inorganic base (see ESI for details†). MacMillan has shown that the presence of the latter is crucial to form the carboxylate, allowing oxidation and affording the release of CO_2_; the use of organic bases gave poorer results. Among all of the iridium catalysts tested (see ESI for full details,† and for the results obtained with catalyst **1**), complex [Ir(ptrz)_2_(*t*Bu-bpy)][BF_4_] (**2**, Fig. 1) gave the best results under the optimized reaction conditions (Scheme 1), also proving to be highly photostable in DMSO after prolonged irradiation (see ESI†). Specifically, complex **2** is easily obtained through a two-step synthesis^22^ involving a facile silver-assisted cyclometalation reaction of 2-methyl-5-phenyl-2*H*-tetrazole with IrCl_3_ and, notably, the solvato complex intermediate can be a useful precursor for other appropriately designed iridium(iii) complexes.

**Scheme 2:**
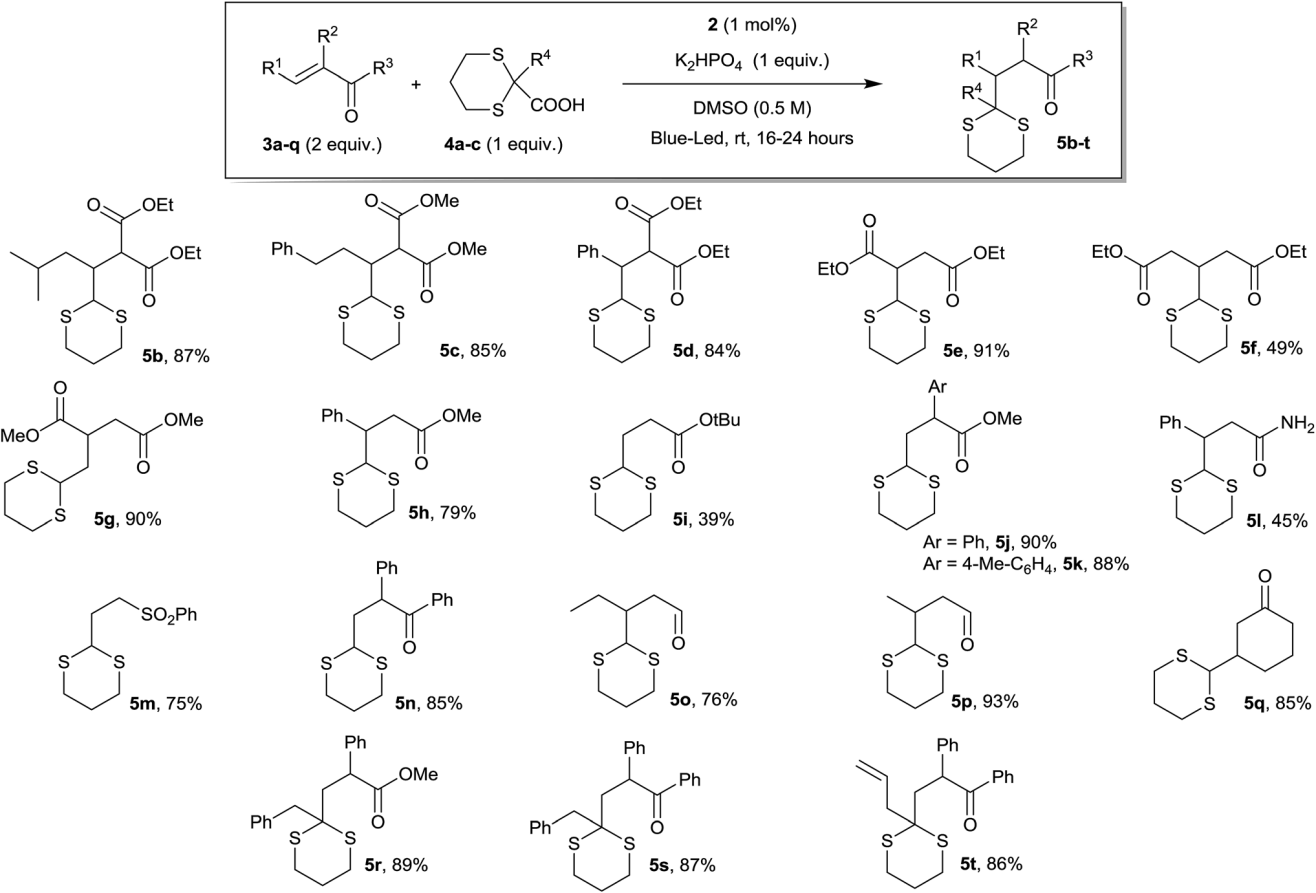
Scope of the reaction with different Michael-acceptor derivatives.

To test the general validity of our approach, we investigated a variety of Michael acceptors and the results obtained are summarized in Scheme 2. Differently substituted Michael acceptors can participate in the dithiane conjugate addition protocol. The mild reaction conditions reported in Scheme 2 are compatible with a wide range of functional groups (*e.g.*, malonates, esters, amides, ketones, aldehydes, sulfones, *etc.*), which together provide a versatile group for further functionalization and transformation. Unsaturated ketones and aldehydes are well tolerated in both cyclic and acyclic forms (*e.g.*, products **5n–q**). In addition, this protocol could be further applied to other electrophilic alkenes, including α,β-unsaturated amides, sulfones, and malonates, as well as acrylates and fumarates, to afford a variety of alkylated dithianes in good to excellent yields. In terms of substituents present in the Michael acceptors, β-substituents are tolerated, as well as α-aryl and α-alkyl groups. It is also worth noting that the tailored synthesis of 2-substituted dithiane-2-carboxylates is possible (see ESI for details†) with a wide range of products (*e.g.*, products **5r–t**). In addition, it was possible to scale up the reaction without any problem from 0.2 mmol to 2.4 mmol.

**Scheme 3:**
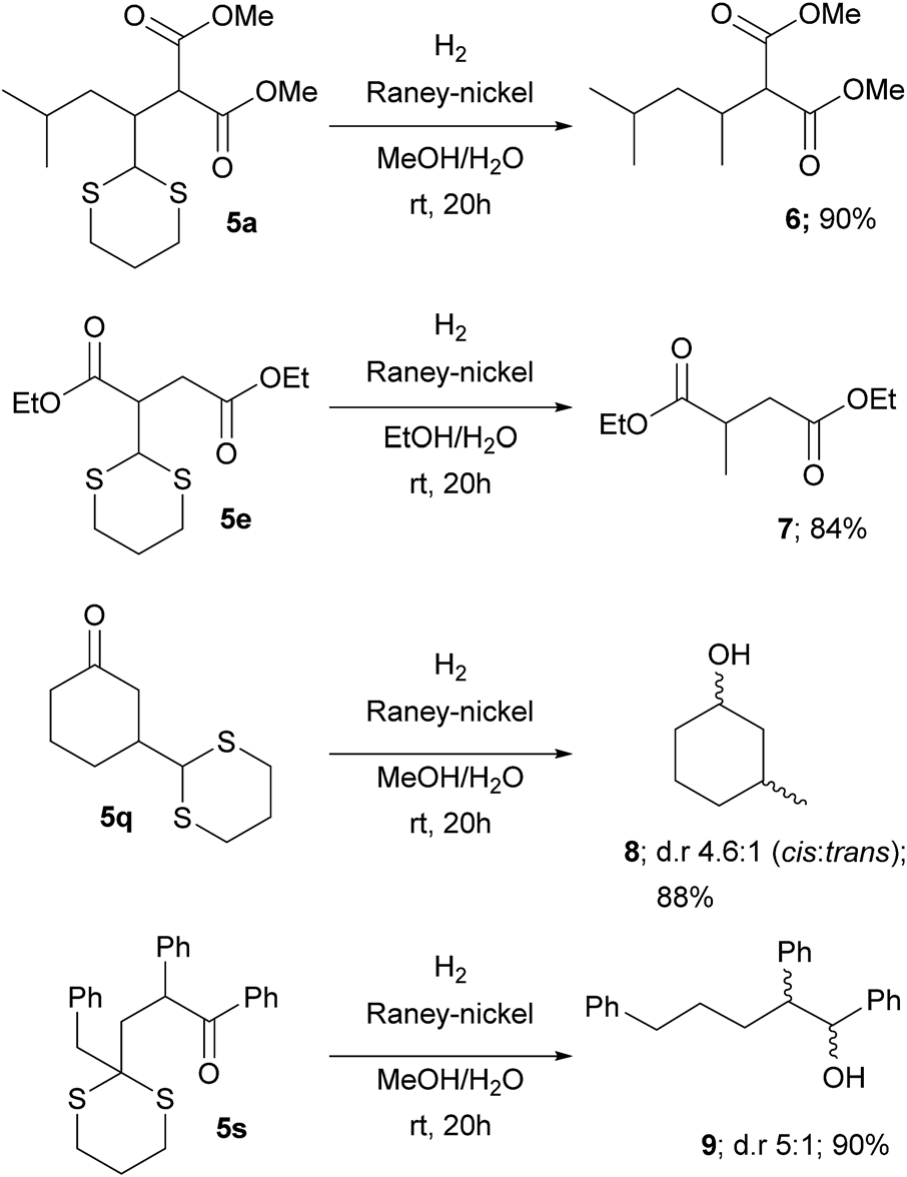
Introduction of a methyl group by reaction with RANEY®-nickel.

**Scheme 4:**
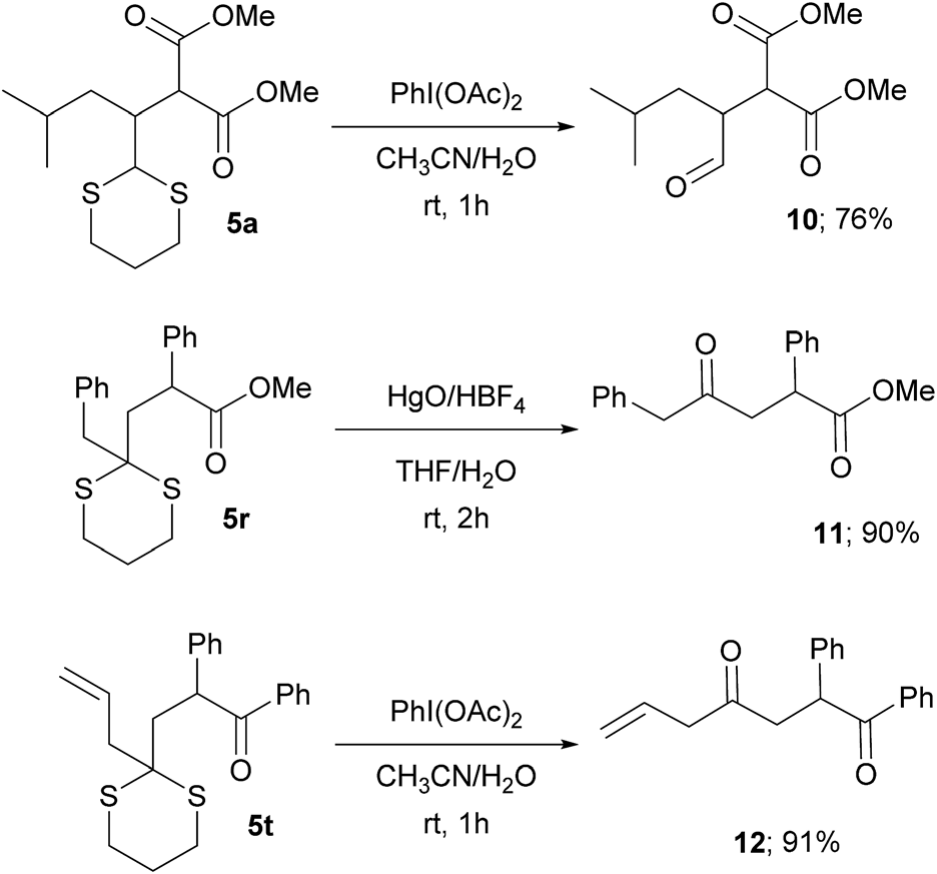
Oxidative deprotection of dithiane adducts.

This protocol avoids the use of strong bases and allows the direct addition of a versatile dithiane under very mild reaction conditions. The methodology, by coupling the reaction with RANEY®-nickel desulfurization realized on the crude reaction mixture, gave direct access to the corresponding methyl group in a straightforward way (Scheme 3). When ketones are present, the direct treatment can lead to their reduction, as observed for **5q** and **5s**. Moderate to good diastereoselection is observed for the reaction. On the other hand, it is possible to transform the dithiane with the corresponding carbonyl group by an easy deprotection reaction under well-established conditions^23^ as is reported with selected examples, giving the desired products in high yields (Scheme 4). Eventually, substituted or unsubstituted dithiane can be used for further modifications.

## Photocatalyst selectivity

**Scheme 5:**
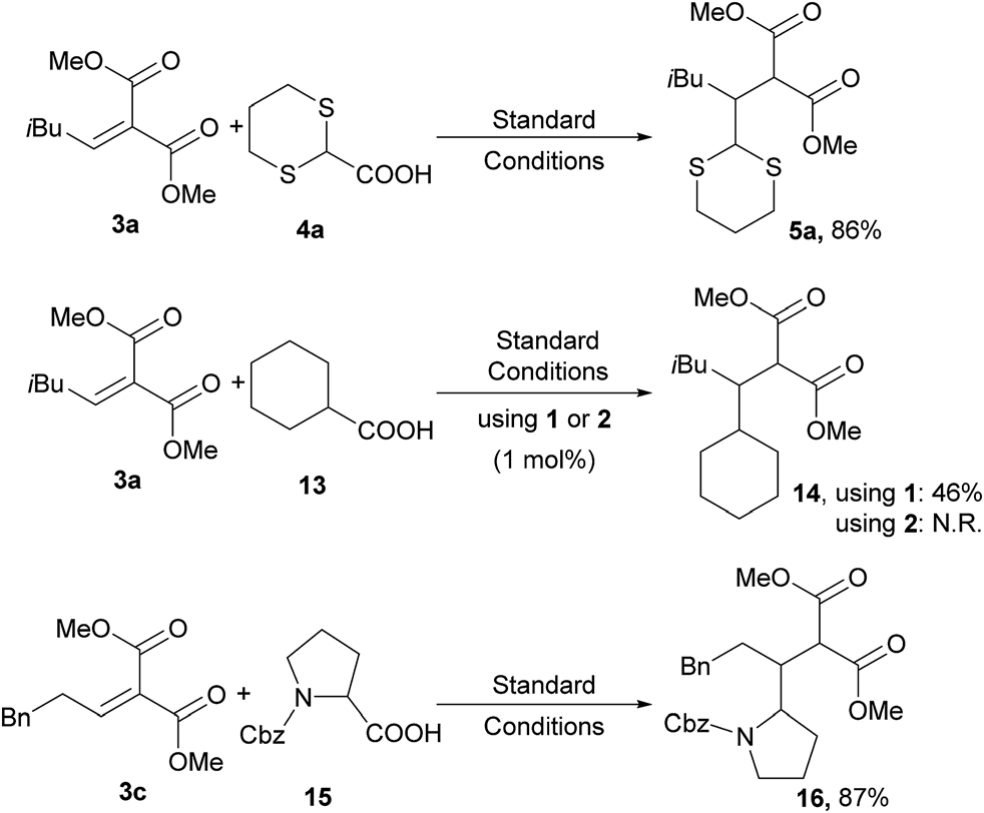
Selective generation of radicals from carboxylate using the iridium photocatalyst **2**.

An interesting feature of the catalyst **2** is its selectivity towards oxidation of substrates. In fact, while the photocatalyst **1** is not able to discriminate between functionalized or unfunctionalized carboxylic acids, the complex **2** is able to selectively oxidize only α-functionalized acids (*e.g.*, **4a** and **15** in Scheme 5). Accordingly, no photo-generated radicals can be formed from unfunctionalized derivatives like, for instance, cyclohexanecarboxylic acid (**13** in Scheme 5).^24^

## Electrochemical and photophysical studies of the catalytic system

The photo-oxidation selectivity of the complex **2**, compared to the iridium catalyst **1**, can be easily understood by comparing the excited-state redox potentials, estimated by combining photophysical and electrochemical data (see ESI for details†).

**Fig. 2:**
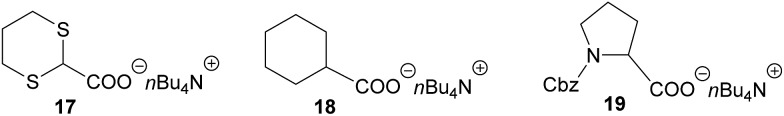
Tetrabutylammonium carboxylates used in electrochemical characterization.

The redox potentials of the two above mentioned iridium photocatalysts (*i.e.*, **1** and **2**), together with those of the tetrabutylammonium carboxylates of selected carboxylic acids (Fig. 2) are gathered in Fig. 2, Fig. S3† and Table 1.

**Table 1:** Electrochemical data determined by cyclic voltammetry and square-wave voltammetry

	*E*_ox_ [V]	*E*_red_ [V]	Δ*E*_redox_ ^*c*^ [V]	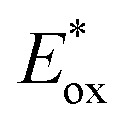 ^*d*^ [V]	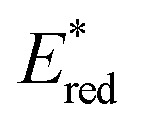 ^*d*^ [V]
**17**	+0.39^*a*^	—	—	—	—
**18**	+0.58^*a*^	—	—	—	—
**19**	+0.37^*a*^	—	—	—	—
**3a** ^*b*^	—	–2.42^*b*^	—	—	—
**1**	+1.32	–1.73	3.05	≈–1.1	≈+0.7
**2**	+1.11	–1.89	3.00	≈–1.2	≈+0.4

^*a*^All of the measurements were performed in room-temperature acetonitrile solution + 0.1 M TBAPF_6_. All of the redox potentials are referenced to the ferrocene/ferrocenium couple, which was used as an internal standard.

^*b*^An irreversible redox process with an estimated error of ±0.05 V.

^*c*^Δ*E*_redox_ = *E*_ox_ – Δ*E*_red_.

^*d*^ 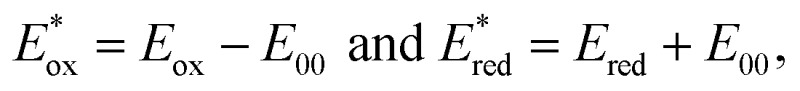 where *E*_00_ is the energy gap between the ground and excited states determined spectroscopically using the data reported in Fig. S4. Estimated error: ±0.1 V.

The electrochemical and photophysical experiments were carried out on carboxylates – instead of pristine carboxylic acids – because only after deprotonation by K_2_HPO_4_ are the acid derivatives oxidized by the iridium photocatalyst, leading to CO_2_ release and their subsequent radical addition of Michael acceptors.^5–7^ Tetrabutylammonium was selected as the counterion to increase the solubility of the carboxylates in acetonitrile and in order to have the same cation as that of the supporting electrolyte used for the electrochemical experiments.^25^

The oxidation potential of the unfunctionalized cyclohexanecarboxylate **18** is approx. 0.2 V higher when compared to that of the more electron-rich heterocyclic compounds **17** and **19** (see Table 1). Therefore, a stronger oxidant is required to activate **18**, while the two functionalized derivatives **17** and **19** are more prone to oxidation.

It is worth noting that, despite both iridium complexes having virtually the same redox gap (of around 3 eV), the oxidation and reduction processes of **2** occur at more positive potentials (approx. +0.2 V) when compared to the polyfluorinated **1** complex. This is due to the lack of electron-withdrawing fluorine substituents in the new tetrazole-based complex. It is well-known that iridium-based photocatalysts (*e.g.*, **1** and **2**) are strongly oxidizing species once excited to their lowest excited state.^5–7^ The excited-state redox potential of these molecules 
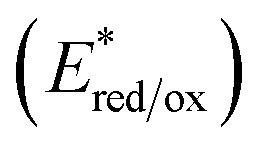
 can be roughly estimated using a simplified version of the so-called Rehm–Weller equation:^26^ 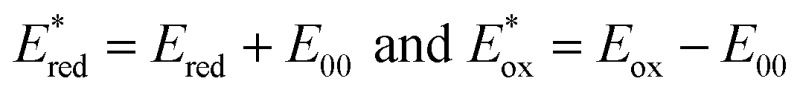
where *E*_00_ is the energy gap between the ground and excited states determined spectroscopically (see Fig. S4† for details about its determination). Despite the fact that this approach can lead only to estimates with uncertainties of 0.1 V or more,^27^ the 
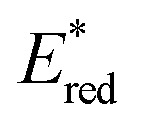
 values of **1** and **2** (Table 1) do provide an explanation for the photo-oxidation selectivity of the complex **2**, compared to the commercially available iridium catalyst **1**. In fact, while the excited-state reduction potential of **2** is not high enough to oxidize **18** (*i.e.*, +0.4 V < +0.58 V), this is not the case for the complex **1**, having 
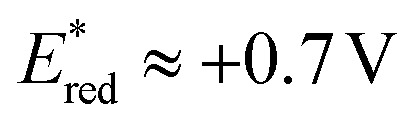
 (*i.e.*, well above the oxidation potential of **18**; see Table 1).

## Stern–Volmer experiments

**Fig. 3:**
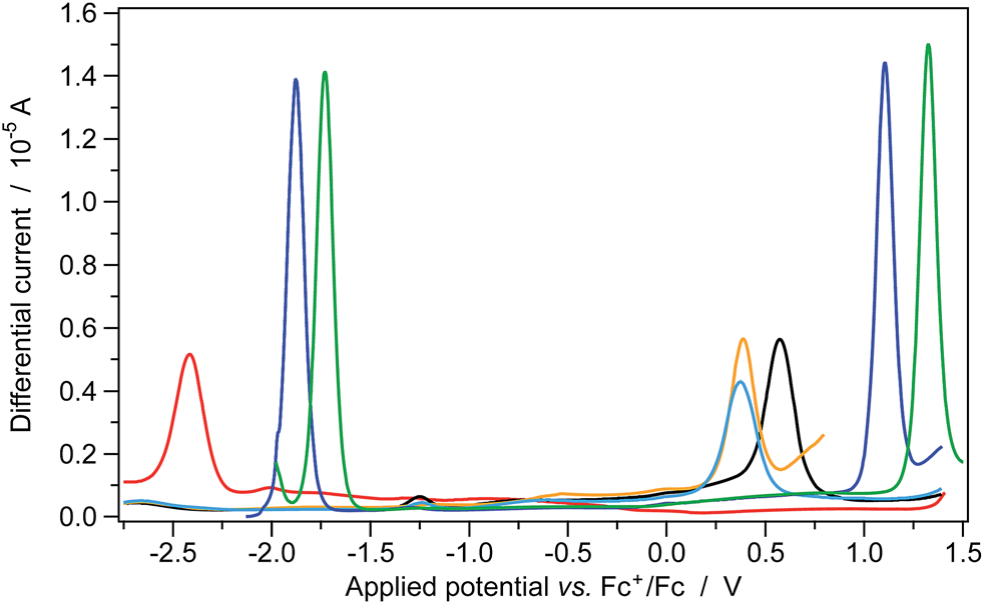
Square-wave voltammograms of carboxylate derivatives **17** (orange), **18** (black) and **19** (light blue), iridium photocatalysts **1** (green) and **2** (dark blue), and the archetypal Michael acceptor **3a** (red); sample concentration: 1 mM. Experiments were carried out in room-temperature acetonitrile solutions and recorded at a scan rate of 100 mV s^–1^ with a square-wave amplitude of ±20 mV and a frequency of 25 Hz.

This scenario is also corroborated by the Stern–Volmer quenching experiments which were carried out to explore in more detail the intramolecular reductive quenching of the photocatalyst phosphorescence operated by the carboxylate substrates. The results are summarized in Table 2 and depicted in Fig. 3. As expected, both **1** and **2** are quenched by the presence of **17** due to a bimolecular quenching process having approximately the same rate constant for both photocatalysts (*k*_q_ ≈ 7 × 10^8^ M^–1^ s^–1^, see Table 2).

**Table 2:** Stern–Volmer experiments performed with the photocatalysts^*a*^

	Photocatalyst **1** ^*a*^		Photocatalyst **2** ^*a*^	
	*K*_SV_ [mM^–1^]	*k*_q_ ^*b*^ [10^8^ M^–1^ s^–1^]	*K*_SV_ [M^–1^]	*k*_q_ ^*b*^ [10^8^ M^–1^ s^–1^]
**17**	1.56 ± 0.05	6.4 ± 0.2	0.89 ± 0.07	7.6 ± 0.6
**18**	0.24 ± 0.03	1.0 ± 0.1	0.039 ± 0.006	0.32 ± 0.05
**3a**	Quenching not observed^*c*^		Quenching not observed^*c*^	

^*a*^All of the experiments were carried out in oxygen-free acetonitrile at 298 K with a photocatalyst concentration of 0.015 mM, with excitation at 330 nm. Data are reported with a ±95% confidence interval. In all cases, the quality of the fitting is assured by a *R* ^2^ > 0.98.

^*b*^ *k*_q_ = *K*_SV_/*τ*_0_, where *τ*_0_ is the unquenched excited-state lifetime of the photocatalyst.

^*c*^There is no evidence of there being correlation between the excited-state quenching of the photocatalyst and the increasing amounts of **3a** up to a concentration of 4 mM.

On the other hand, the unsubstituted carboxylate **18** is able to quench the excited state of **1** with a *k*_q_ that is more than three times higher than in the case of **2** (*i.e.*, 1.0 *vs.* 0.3 × 10^8^ M^–1^ s^–1^, see Table 2).

**Fig. 4:**
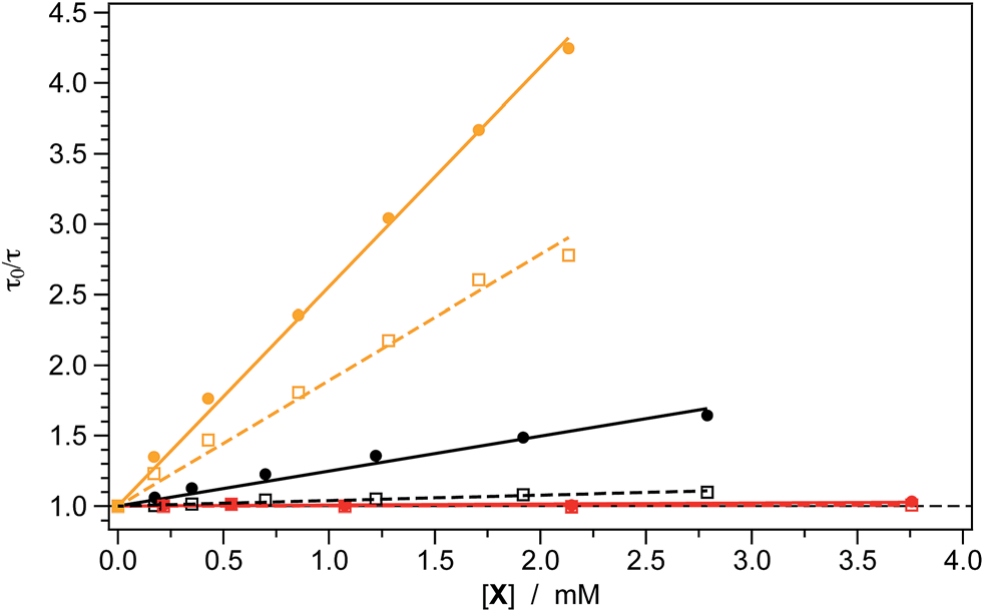
Stern–Volmer plots showing the quenching of the excited-state lifetime of the photocatalysts **1** (full circles and lines) and **2** (empty squares and dashed lines) in the presence of increasing amounts of the carboxylate derivatives **17** (orange) or **18** (black). Notably, no quenching is observed if the Michael acceptor **3a** (red) is added to both iridium complexes. Experiments were carried out in oxygen-free acetonitrile solution at 298 K with a photocatalyst concentration of 0.015 mM, with excitation at 330 nm.

As shown in Fig. 4, the excited-state lifetime of both **1** and **2** is virtually unaffected by the presence of the Michael acceptor **3a** (at least for concentrations up to 3.75 mM). This evidence is in accordance with the electrochemical data reported in Table 1, showing that the excited-state redox potentials of both of the iridium photocatalysts cannot promote any redox process on **3a**.

## Determination of quantum yield

In order to estimate the efficiency of our photocatalyst **2** and to assess the potential presence of an important radical chain contribution to the catalytic cycle, we evaluated the quantum yield of the reaction between the Michael acceptor **3a** and the carboxylate **17**, under the optimized conditions reported in the ESI.† The reaction was irradiated at 334 nm with a 100 W Hg lamp; see the Experimental section for further details. The choice of such an excitation wavelength was dictated by: (i) the higher molar absorptivity of the photocatalyst compared to 450 nm blue LED excitation;^22^ (ii) the high reliability of the potassium ferrioxalate actinometer in this spectral region, which is not the case for *λ* > 450 nm;^28^ (iii) the still high selectivity of excitation, since all the reagents are optically transparent for *λ* > 300 nm (see Fig. S6†).

The determined quantum yield of the reaction is 0.28 ± 0.05. This value indicates that a radical chain mechanism is unlikely in our reaction; however, it could not be totally ruled out.^29^

## Proposed reaction mechanism

**Fig. 5:**
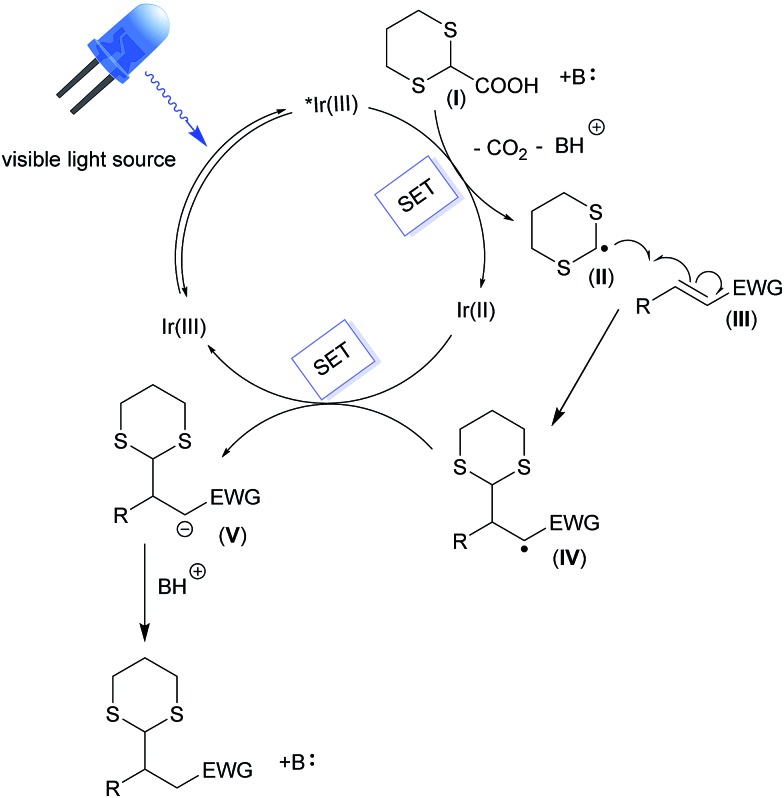
Proposed reaction mechanism.

The above illustrated experimental findings corroborate the reaction mechanism depicted in Fig. 5. Upon light absorption in the visible part of the electromagnetic spectrum, the iridium photocatalyst (Ir^III^) is excited to its lowest electronic excited state (*Ir^III^). This initial event is the only one possible, since all of the other molecules are transparent to visible light (see Fig. S5 and S6†). Next, the photoexcited complex is able to oxidize a suitable carboxylate derivative (**I**) inducing its decarboxylation and the formation of the corresponding radical species (**II**). Subsequently, the addition of this radical to the Michael acceptor (**III**) affords the radical intermediate **IV**, which undergoes a second SET event from the reduced iridium complex (Ir^II^). The photocatalyst is then restored and **IV** is converted into the carbanionic intermediate **V**. The latter can be easily protonated, leading to the final reaction products.

## Conclusions

In conclusion, we have developed a practical and effective photocatalytic addition of dithiane-2-carboxylates to Michael-type acceptors promoted by the iridium(iii) complex **2**, which is used for the first time as a powerful photocatalyst. The reaction has broad scope and allows the introduction of dithiane in a variety of Michael acceptors (unsaturated ketones, esters, amides, malonates, *etc.*) in high yields. The adducts can be further functionalized with established chemistry by oxidative deprotection or alkylation. In particular, this photocatalytic reaction can be used to introduce a methyl group to unsaturated compounds, after elimination of dithiane by RANEY®-nickel. The photocatalyst **2** is easily accessible and its redox properties can be finely tuned by chemical modification of the ligands, affording a brand new class of new Ir(iii) photocatalysts. The electrochemical potential of **2** allows tailored oxidation and this opens the way to its selective use in photocatalysis to activate substrates. Studies on a stereoselective variant of the reaction proposed here are under active investigation in our laboratories.

## Experimental section

### General procedure

In a Schlenk tube with a rotaflo® stopcock under an argon atmosphere at r.t., catalyst **2** (1.7 mg, 0.002 mmol), 1,3-dithiane-2-carboxylic acid **4a** (0.2 mmol, 0.032 g) and K_2_HPO_4_ (0.2 mmol, 0.034 g) were dissolved in 400 μL of DMSO. After 2 min, the Michael acceptor (0.4 mmol) was added. The reaction mixture was carefully degassed *via* freeze–pump–thaw (three times), and the vessel was refilled with argon. The Schlenk tube was stirred and irradiated with a blue LED positioned approximately at a 10 cm distance from the reaction vessel. After 16 h of irradiation, NaHCO_3_ sat. solution (2 mL) was added and the mixture was extracted with ethyl acetate (4 × 5 mL). The collected organic layers were dried over Na_2_SO_4_, filtered and concentrated under reduced pressure to give the crude products. Column chromatography on silica (cyclohexane:ethyl acetate or cyclohexane:Et_2_O) afforded pure compounds.

### Electrochemistry

Voltammetric experiments were performed using a Metrohm AutoLab PGSTAT 302 electrochemical workstation in combination with the NOVA software package. All of the measurements were carried out at room temperature in acetonitrile solutions with a sample concentration of approx. 1 mM using 0.1 M tetrabutylammonium hexafluorophosphate (electrochemical grade, TBAPF_6_) as the supporting electrolyte. Oxygen was removed from the solutions by bubbling them with argon for 20 minutes. All of the experiments were carried out using a three-electrode setup (BioLogic VC-4 cell, with a cell volume of 5 mL) with a glassy-carbon working electrode (1.6 mm diameter), the Ag/AgNO_3_ redox couple (0.01 M in acetonitrile with 0.1 M TBAClO_4_ supporting electrolyte) as the reference electrode and a platinum wire as the counter electrode. At the end of each measurement, ferrocene was added as the internal reference. Cyclic voltammograms (CV) were typically recorded at a scan rate of 200 mV s^–1^, but several rates were used to check reversibility (in the range between 50 and 2000 mV s^–1^). Osteryoung square-wave voltammograms (OSWV) were recorded with a scan rate of 100 mV s^–1^, a SW amplitude of ±20 mV and a frequency of 25 Hz.

### Photophysical measurements

All of the spectroscopic investigations were carried out in spectrofluorimetric grade acetonitrile using fluorimetric Suprasil® quartz cuvettes with a 10.00 mm path length. Absorption spectra were recorded with a Perkin-Elmer Lambda 950 spectrophotometer. All photoluminescence experiments were performed in oxygen-free solution, by removing oxygen through argon bubbling for 20 minutes. The uncorrected emission spectra were obtained with an Edinburgh Instruments FLS920 spectrometer equipped with a Peltier-cooled Hamamatsu R928 photomultiplier tube (PMT) (185–850 nm). An Edinburgh Xe 900 (450 W xenon arc lamp) was used as the excitation light source. The corrected spectra were obtained *via* a calibration curve supplied with the instrument. The photoluminescence quantum yields (PLQY) in solution were obtained from the corrected spectra on a wavelength scale (nm) and measured according to the approach described by Demas and Crosby^30^ using an air-equilibrated water solution of quinine sulfate in 1 N H_2_SO_4_ as the reference (PLQY = 0.546).^31^ The emission lifetimes (*τ*) were measured through the time-correlated single photon counting (TCSPC) technique using an HORIBA Jobin Yvon IBH FluoroHub controlling a spectrometer equipped with a pulsed NanoLED (*λ*_exc_ = 330 nm; FWHM = 11 nm) as the excitation source and a red-sensitive Hamamatsu R-3237-01 PMT (185–850 nm) as the detector. Analysis of the luminescence decay profiles was accomplished with DAS6 Decay Analysis Software provided by the manufacturer, and the quality of the fitting was assessed with the *χ* ^2^ value close to unity and with the residuals regularly distributed along the time axis. Samples were excited at 340 nm for the evaluation of PLQYs and at 330 nm for *τ* determination. Experimental uncertainties are estimated to be ±10% for *τ* determinations, ±20% for PLQY, and ±2 nm and ±5 nm for absorption and emission peaks, respectively. Stern–Volmer quenching experiments were performed at room-temperature under oxygen-free conditions (in an argon-saturated environment) using 3 mL of acetonitrile solution containing the appropriate iridium photocatalyst (with a concentration of 1.5 × 10^–5^ M) and increasing amounts of quencher. For determination of the photocatalytic quantum yield, the reaction was carried out in spectrofluorimetric grade DMSO and placed in a Suprasil® quartz cuvette with a 2.00 mm path length. The reaction mixture was excited at 334 nm, using a 100 W Hg lamp equipped with an appropriate dichroic filter. The photon flux was estimated using a ferrioxalate actinometer, following the procedure reported by Montalti *et al.* ^28^ The conversion of the reaction was determined by ^1^H-NMR.
