# Supplementary material for: Photoredox radical conjugate addition of dithiane-2-carboxylate promoted by an iridium(iii) phenyl-tetrazole complex: a formal radical methylation of Michael acceptors
Source: Chem Sci. 2016 Nov 3;8(2):1613–20. doi: 10.1039/c6sc03374a (PMC5364518; doi:10.1039/c6sc03374a)
Supplement: Supplementary file 1 [file SC-008-C6SC03374A-s001.pdf]

Electronic Supplementary Material (ESI) for Chemical Science.
This journal is © The Royal Society of Chemistry 2016

Photoredox Radical Conjugate Addition of Dithiane-2-carboxylate Promoted by an Iridium(III) Phenyl-tetrazole Complex: a Formal Radical Methylation of Michael Acceptors

Andrea Gualandi,[a] Elia Matteucci,[b] Filippo Monti,[c] Andrea Baschieri,[a] Nicola Armaroli,[c]* Letizia Sambri,[b]* and Pier Giorgio Cozzi[a]*

a. Dipartimento di Chimica "G. Ciamician", ALMA MATER STUDIORUM Università di Bologna, Via Selmi 2, 40126 Bologna, Italy E-mail: piergiorgio.cozzi@unibo.it

b. Dipartimento di Chimica Industriale "Toso Montanari", Università di Bologna, Viale Risorgimento 4, 40136, Bologna, Italy E-mail: letizia.sambri@unibo.it

c. Istituto per la Sintesi Organica e la Fotoreattività, Consiglio Nazionale delle Ricerche, Via P. Gobetti 101, 40129 Bologna, Italy. E-mail: nicola.armaroli@isof.cnr.it

Electronic Supplementary Information

Table of contents:

General methods and materials	S3
Optimization of radical conjugate addition of dithiane-2-carboxylate	S4
Preparation of the starting Michael acceptors	S5
Preparation of 2-substituted 1,3-dithiane-2-carboxylate **4b,c**	S7
Photoredox radical conjugate addition of carboxylates	S8
Characterization data of products **5a-t, 15** and **16**	S8
Reductive removal of the dithiane moiety	S13
Hydrolysis of the dithiane moiety	S14
Reaction in presence of TEMPO	S15
Preparation of ammonium salts **17, 18, 19**	S16
Photoredox radical conjugate addition of salt **17**	S17
Photostability of Ir(III) complex **2**	S17
Photophysical and electrochemical data	S19
Determination of quantum yield	S21
References	S21
Copies of NMR spectra	S23
Copies of IR spectra	S61

General methods and materials. ^{1}H NMR spectra were recorded on Varian Gemini 200, Varian Mercury 400. Chemical shifts are reported in ppm from TMS with the solvent resonance as the internal standard (deuterochloroform: δ = 7.24 ppm). Data are reported as follows: chemical shift, multiplicity (s = singlet, d = duplet, t = triplet, q = quartet, dd = double duplet, dt = double triplet, bs = broad signal, m = multiplet), coupling constants (Hz). ^{13}C NMR spectra were recorded on Varian Gemini 200, Varian MR400 spectrometers. Chemical shifts are reported in ppm from TMS with the solvent as the internal standard (deuterochloroform: δ = 77.0 ppm). GC-MS spectra were taken by EI ionization at 70 eV on a Hewlett-Packard 5971 with GC injection. LC-electrospray ionization mass spectra (ESI-MS) were obtained with Agilent Technologies MSD1100 single-quadrupole mass spectrometer. They are reported as: *m/z* (rel. intense). IR spectra were recorded using Bruker Alpha FTIR spectrometer equipped with single reflection diamond ATR accessory. Chromatographic purification was done with 240-400 mesh silica gel. Purification on preparative thin layer chromatography was done on Merck TLC silica gel 60 F_{254}.

Materials. If not otherwise stated, all reactions were carried out in flame dried glassware under nitrogen atmosphere. Anhydrous solvents were supplied by Aldrich in Sureseal® bottles and were used as received avoiding further purification.

Reagents were purchased from Aldrich and AlfaAesar, and used without further purification unless otherwise stated. Compounds **3e**, **3f**, **3h**, **3i**, **3l**, **3m**, **3o**, **3p**, **3q** and **25** were commercially available. 1,3-dithianecarboxylic acid **4a** was prepared according reported procedure.[1] Iridium complexes **1**,[2] **2**,[3] **19-20**,[4] **21**,[3] **22**,[2] and **23-24**[4] were prepared according to described procedures.

Optimization of radical conjugate addition of dithiane-2-carboxylate

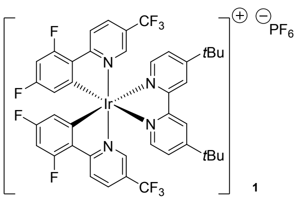
1

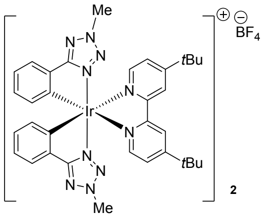
2

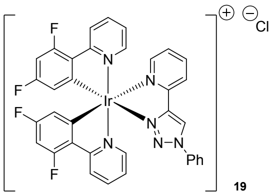
19

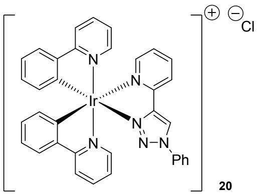
20

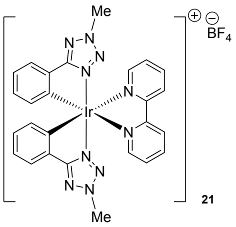
21

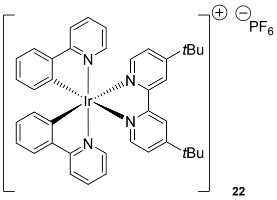
22

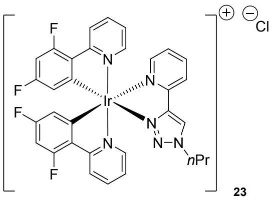
23

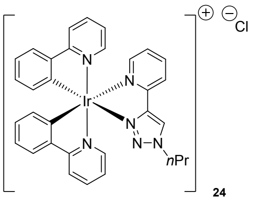
24

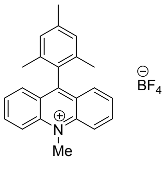
25

Table S1. Iridium catalyst screening.[a]

Entry	Catalyst	Conversion (%)[b]
1	**1**	93
2	**2**	97
3	**19**	18
4	**20**	17
5	**21**	72
6	**22**	47
7	**23**	13
8	**24**	13
9	**25**	0

[a] The reactions were performed at r.t. with 0.1 mmol of **4a**, 2 equiv of malonate (**3a**) and 1 equiv of K_2HPO_4, in the presence of 1 mol% of photocatalyst and in DMSO (0.2 M), and stopped after 16 h. [b] Determined by ^{1}H NMR using internal standard.

Table S2. Solvent screening.[a]

Entry	Solvent	Conversion (%)[b]
1	DMSO	97
2	DMF	53
3	DCE	11
4	EtOH	34
5	EtOH:H_2O (8:2)	45

[a] The reactions were performed at r.t. with 0.1 mmol of **4a**, 2 equiv of malonate (**3a**) and 1 equiv of K_2HPO_4, in the presence of 1 mol% of iridium complex **2** and in reported solvent (0.2 M), and stopped after 16 h. [b] Determined by ^{1}H NMR using internal standard.

Table S3. Base screening.[a]

Entry	Base (equiv.)	Solvent	Conversion (%)[b]
1	Lutidine (1)	DMSO	65
2	Lutidine (0.5)	DMSO	35
3	-	DMSO	2
4	K_2HPO_4 (1.5)	DMSO	75
5	K_2HPO_4 (1)	DMSO	97
6	$CsCO_3$ (1)	DMSO	0
7	DBU (1)	DMSO	45
8	$CsCO_3$ (1)	DMF	0
9	DBU (1)	DMF	30

[a] The reactions were performed at r.t. with 0.1 mmol of **4a**, 2 equiv of malonate (**3a**) and base, in the presence of 1 mol% of iridium complex **2** and in reported solvent (0.2 M), and stopped after 16 h. [b] Determined by ^{1}H NMR using internal standard.

Table S4

Entry	Conversion (%)[b]	Variation from standard conditions
1	97	-
2	0	No Light
3	0	No iridium complex
4	37	**4a**:**3a** 1.5:1
5	42	**4a**:**3a** 1:1
6	60	**4a**:**3a** 1:1.5
7	94	Temperature controlled at 25±3°C

[a] Standard conditions: 0.1 mmol of **4a**, 2 equiv of malonate (**3a**) and 1 equiv of K_2HPO_4, in the presence of 1 mol% of iridium complex **2** and in DMSO (0.2 M), and stopped after 16 h. [b] Determined by ^1H NMR using internal standard.

Preparation of starting Michael acceptors

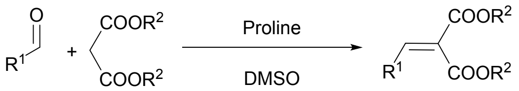

3a-d

3a, R[1] = *i*Bu, R[2] = Me
3b, R[1] = *i*Bu, R[2] = Et
3c, R[1] = BnCH2, R[2] = Me
3d, R[1] = Ph, R[2] = Et

Compounds **3a-d** were prepared following reported procedure.[5] To a solution of proline (1 mmol, 0.115 g) in DMSO (3.3 mL) aldehyde (10 mmol) was added. After 10 min. malonate (10 mmol) was slowly added and the reaction stirred overnight. The reaction mixture was diluted with Et_2O (30 mL) and washed with water (20 mL), $NaHCO_3$ sat. sln. (20 mL) and brine (20 mL). The organic phase was concentrated under reduced pressure and the residue was purified by column chromatography on silica (cyclohexane:ethyl acetate from 95:5 to 90:10) to give pure **3a-d** as white oils (65-84% yields). Spectroscopic data were according to the literature.[6]

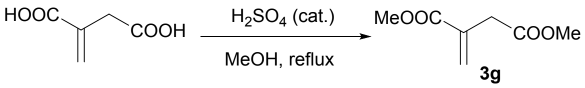

3g

To a solution of itaconic acid (3.9 mmol, 0.500 g) in MeOH (10 mL) some drops of concentrated sulfuric acid were added. The reaction was refluxed for 8 hours, cooled at room temperature and the solvent was evaporated under reduced pressure. The residue was dissolved in Et_2O (30 mL) and washed with $NaHCO_3$ sat. sln. (2 x 10 mL) and brine (20 mL). The organic phase was concentrated under reduced pressure to give **3g** (94%, 3.7 mmol, 0.576 g) as colorless oil. The product was used in the next step without purification.

^1H NMR (400 MHz, $CDCl_3$, 25°C): δ = 6.27 (s, 1H), 5.67 (s, 1H), 3.71 (s, 3H), 3.64 (s, 3H), 3.29 (s, 2H); ^{13}C NMR (100 MHz, $CDCl_3$, 25°C): δ =171.0, 166.5, 133.6, 128.4, 52.0, 51.9, 37.4.

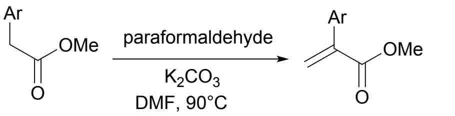

3j,k

3j, Ar = Ph

3k, Ar = 4-Me-C_6H_4

Compounds **3j,k** were prepared following reported procedure.[7] A mixture of ester (7.0 mmol, 1.044 g, 1 mL), paraformaldehyde (10.5 mmol, 0.313 g) and K_2CO_3 (7.0 mmol, 0.960 g) in DMF (5 mL) was heated at 100°C for 6h. The mixture was cooled at room temperature, water (20 mL) and Et_2O (30 mL) were added and the organic layer was separated. The aqueous layer was extracted with Et_2O (2 x 20 mL). The collected organic layers were washed with brine (10 mL), dried over Na_2SO_4 and concentrated under reduced pressure to give the crude products. Column chromatography on silica (cyclohexane:Et_2O from 100:0 to 90:5) afforded pure **3j,k** as colorless oils.

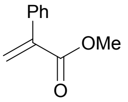

3j: colorless oil; 73% yield; ^{1}H NMR (400 MHz, $CDCl_3$, 25°C): δ = 7.45 – 7.28 (m, 5H), 6.36 (d, J=1.2, 1H), 5.88 (d, J=1.2, 1H), 3.81 (s, 3H); ^{13}C NMR (100 MHz, $CDCl_3$, 25°C): δ = 167.2, 141.3, 136.6, 128.2 (2C), 128.1, 128.0 (2C), 126.8, 52.1.

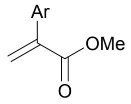

Ar = 4-Me-C_6H_4

3k: colorless oil, 85% yield; ^{1}H NMR (400 MHz, $CDCl_3$, 25°C): δ = 7.33 – 7.28 (m, 2H), 7.19 – 7.14 (m, 2H), 6.31 (d, J=1.3, 1H), 5.86 (d, J=1.3, 1H), 3.81 (s, 3H), 2.36 (s, 3H). ^{13}C NMR (100 MHz, $CDCl_3$, 25°C): δ = 167.4, 141.1, 138.0, 133.8, 128.8 (2C), 128.1 (2C), 126.0, 52.1, 21.1.

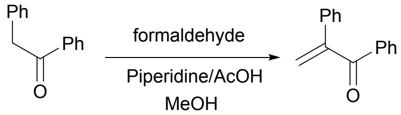

3n

Compounds **3n** were prepared following reported procedure.[8] A mixture of deoxibenzoin (5.1 mmol, 1.0 g), formaldehyde (38% in water, 1.8 mL), piperidine (0.66 mmol, 65 μL) and acetic acid (1.1 mmol, 63 μL) in MeOH (13 mL) was refluxed for 6h. The mixture was cooled at room temperature and the volatiles were evaporated under reduced pressure. The residue was dissolved in ethyl acetate (30 mL) and washed with 1 M HCl (10 mL), $NaHCO_3$ sat. sln. (10 mL) and brine (20 mL). The organic layer was dried over Na_2SO_4 and concentrated under reduced pressure to give the crude product. Column chromatography on silica (cyclohexane:ethyl acetate from 95:5 to 80:20) afforded pure **3n** as colorless oil (94%, 4.8 mmol, 1.0 g).

(**3n**): ^{1}H NMR (400 MHz, $CDCl_3$, 25°C): δ = 7.95 – 7.87 (m, 2H), 7.58 – 7.47 (m, 1H), 7.47 – 7.41 (m, 4H), 7.41 – 7.24 (m, 3H), 6.06 (s, 1H), 5.63 (s, 1H); ^{13}C NMR (100 MHz, $CDCl_3$, 25°C): δ =

241.8, 216.7, 167.4, 156.2, 156.2, 152.2, 149.1 (2C), 147.8 (2C), 147.6 (2C), 146.2 (2C), 140.0.

Preparation of 2-substituted 1,3-dithiane-2-carboxylate 4b,c

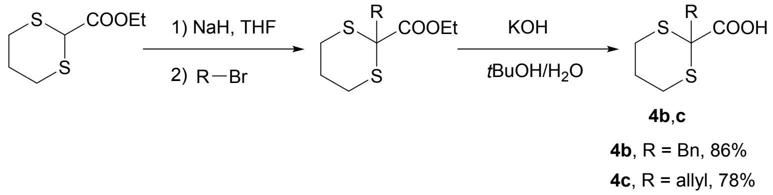

To a solution of 1,3-dithiane-2carboxylate (0.95 mmol, 0.183 g, 154 µL) in THF (3 mL), NaH (60% suspension in mineral oil, 1.9 mmol, 0.076 g) was slowly added at 0°C. The reaction mixture was gently heated at 70°C for 20 min. and cooled at room temperature. Alkylating agent (1.4 mmol) was added and the reaction mixture was stirred overnight. After that, the reaction was cooled at 0°C, water (10 mL) and ethyl acetate (20 mL) were added and the organic layer was separated. The aqueous layer was extracted with ethyl acetate (2 x 20 mL). The collected organic layers were washed with brine (10 mL), dried over Na_2SO_4 and concentrated under reduced pressure to give the crude ester product.

The residue was dissolved in *t*BuOH (4 mL) and 1M KOH aq. (4 mL) was added. After 24 hours volatiles were evaporated under reduced pressure, water (10 mL) and Et_2O (20 mL) were added and the organic layer was separated. The aqueous layer was extracted with Et_2O (2 x 10 mL). 5 M HCl aq. was slowly added to aqueous layer until pH = 1, and they were extracted with DCM (4 x 20 mL). The collected organic layers were washed with brine (10 mL), dried over Na_2SO_4 and concentrated under reduced pressure to give pure compounds as white solids, that were used in the photocatalytic reaction without further purification.

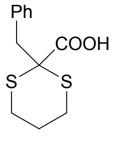

4b: White solid, 87% yield (two steps); m.p. = 79°C; ^{1}H NMR (400 MHz, $CDCl_3$, 25°C): δ = 7.39 – 7.32 (m, 2H), 7.31 – 7.25 (m, 3H), 3.42 – 3.31 (s, 2H), 3.26 (ddd, *J*=14.8, 12.8, 2.6, 2H), 2.76 – 2.63 (m, 2H), 2.18 – 2.08 (m, 1H), 1.91 – 1.76 (m, 1H); ^{13}C NMR (100 MHz, $CDCl_3$, 25°C): δ = 176.7, 134.0, 130.8 (2C), 128.0 (2C), 127.5, 53.2, 44.3, 29.6, 28.0, 24.1; HRMS calcd for $C_{12}H_{14}O_2S_2$: 254.0435; found: 254.0430.

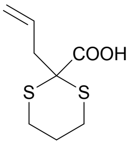

4c: White solid, 83% yield (two steps); m.p. = 56°C;^{1}H NMR (400 MHz, $CDCl_3$, 25°C): δ= 11.57 (bs, 1H), 5.95 – 5.78 (m, 1H), 5.26 – 5.10 (m, 2H), 3.33 (ddd, *J*=14.1, 12.9, 2.6, 2H), 2.73 (d, *J*=7.1, 2H), 2.63 (ddd, *J*=14.2, 4.2, 3.1, 2H), 2.26 – 2.00 (m, 1H), 1.91 – 1.70 (m, 1H); ^{13}C NMR (100 MHz, $CDCl_3$, 25°C): δ= 177.2, 131.1, 119.9, 50.2, 42.7,

27.6 (2C), 24.1; HRMS calcd for $C_8H_{12}O_2S_2$: 204.0279; found: 204.0274.

Photoredox radical conjugate addition of carboxylates

General procedure on 0.2 mmol scale: In a Schlenk tube with rotaflo stopcock under argon atmosphere at r.t., catalyst **2** (1.7 mg, 0.002 mmol), 1,3-dithiane-2-carboxylic acid **4a** (0.2 mmol, 0.032g) and K_2HPO_4 (0.2 mmol, 0.034 g), were dissolved in 400 μL DMSO. After 2 min, Michael acceptor (0.4 mmol) was added. The reaction mixture was carefully degassed via freeze-pump thaw (three times), and the vessel refilled with argon. The Schlenk tube was stirred and irradiated with a blu LED positioned approximately at 10 cm distance from the reaction vessel. After 16 h of irradiation, $NaHCO_3$ sat. sln. (2 mL) was added and the mixture was extracted with ethyl acetate (4 x 5 mL). The collected organic layers were dried over Na_2SO_4, filtered and concentrated under reduced pressure to give crude products. Column chromatography on silica (cyclohexane:ethyl acetate or cyclohexane:Et_2O) afforded pure compounds.

General procedure on 2.4 mmol scale: The reaction was conducted applying the same protocol used for 0.2 mmol scale but using: catalyst **2** (10.4 mg, 0.012 mmol), 1,3-dithiane-2-carboxylic acid **4a** (2.4 mmol, 0.393 g) and K_2HPO_4 (2.4 mmol, 0.417 g) and Michael acceptor **5a** (4.8 mmol, 0.960 g), were dissolved in 4.8 mL DMSO. Product **5a** was obtained in 84% yield.

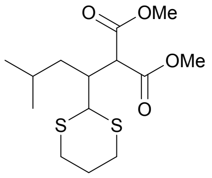

5a: colorless oil, 86% yield; ^{1}H NMR (400 MHz, $CDCl_3$, 25°C): δ = 4.31 (d, J=4.4, 1H), 3.77 (d, J=7.0, 1H), 3.73 (s, 3H), 3.71 (s, 3H), 2.94 – 2.71 (m, 4H), 2.71 – 2.55 (m, 1H), 2.14 – 1.98 (m, 1H), 1.93 – 1.75 (m, 1H), 1.68 (ddd, J=14.0, 7.3, 5.7, 1H), 1.59 (dt, J=13.4, 6.6, 1H), 1.36 – 1.26 (m, 1H), 0.90 (d, J=6.5, 3H), 0.87 (d, J=6.5, 3H); ^{13}C NMR (100 MHz, $CDCl_3$, 25°C): δ = 169.0, 168.9, 53.6, 52.6, 52.4, 52.2, 41.1, 38.5, 31.0, 30.5, 26.5, 25.9, 22.5, 22.4.; HRMS calcd for $C_{14}H_{24}O_4S_2$: 320.1116; found: 320.1108.

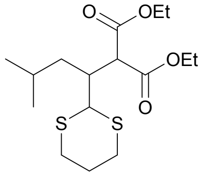

5b: colorless oil, 87% yield; ^{1}H NMR (400 MHz, $CDCl_3$, 25°C): δ = 4.33 (d, J=4.3, 1H), 4.25 – 4.11 (m, 4H), 3.72 (d, J=7.0, 1H), 2.93 – 2.72 (m, 4H), 2.68 – 2.57 (m, 1H), 2.11 – 2.00 (m, 1H), 1.89 – 1.73 (m, 1H), 1.73 – 1.55 (m, 3H), 1.26 (t, J=7.2, 6H), 0.88 (dd, J=10.0, 6.4, 6H); ^{13}C NMR (100 MHz, $CDCl_3$, 25°C): δ = 168.7, 168.5, 61.5, 61.4, 54.1, 52.4, 40.9, 38.6, 31.1, 30.6, 26.5, 26.0, 22.6, 22.5, 14.0 (2C); EI-MS: m/z = 347 (2), 303 (10, M-OEt), 257 (26), 188 (76), 145 (100), 119 (65); HRMS calcd for $C_{16}H_{28}O_4S_2$: 348.1429; found: 348.1422.

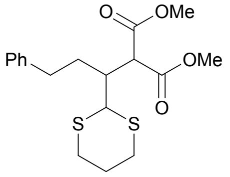

5c: colorless oil, 85% yield; ^{1}H NMR (400 MHz, $CDCl_3$, 25°C): δ = 7.30 – 7.22 (m, 2H), 7.21 – 7.08 (m, 3H), 4.31 (d, J=5.3, 1H), 3.86 (d, J=6.8, 1H), 3.73 (s, 3H), 3.72 (s, 3H), 2.87 – 2.77 (m, 4H), 2.76 – 2.63 (m, 3H), 2.17 – 1.99 (m, 2H), 1.94 – 1.74 (m, 2H); ^{13}C NMR (100 MHz, $CDCl_3$, 25°C): δ = 169.0, 168.8, 141.7, 128.4 (2C), 128.3 (2C), 125.9, 53.4, 52.7, 52.5, 51.5, 42.7, 34.9, 31.8, 30.7, 30.3, 25.9; EI-MS: m/z = 236 (56), 145 (100), 119 (65), 91 (45); HRMS calcd for $C_{18}H_{24}O_4S_2$: 368.1116; found: 368.119.

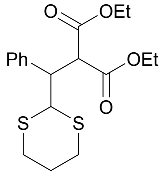

5d: white solid; m.p. = 77 °C, 84% yield; ^{1}H NMR (400 MHz, $CDCl_3$, 25°C): δ = 7.60 – 6.97 (m, 5H), 4.57 (d, J=6.1, 1H), 4.21 (q, J=6.9, 2H), 4.17 (d, J=10.7, 1H), 3.90 (dd, J=10.7, 6.1, 1H), 3.84 (q, J=7.2, 2H), 2.91 – 2.67 (m, 4H), 1.98 (m, 1H), 1.76 (m, 1H), 1.25 (t, J=6.9, 3H), 0.89 (t, J=7.1, 3H); ^{13}C NMR (100 MHz, $CDCl_3$, 25°C): δ = 167.9, 167.4, 136.7, 129.4 (2C), 127.9 (2C), 127.8, 61.7, 61.3, 54.9, 51.3, 49.4, 30.5, 30.4, 25.6, 14.0, 13.6; HRMS calcd for $C_{18}H_{24}O_4S_2$: 368.1116; found: 368.1109.

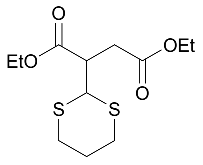

5e: colorless oil, 91% yield; ^{1}H NMR (400 MHz, $CDCl_3$, 25°C): δ = 4.26 – 4.15 (m, 3H), 4.15 – 4.08 (m, 2H), 3.37 (dddd, J=9.5, 7.1, 4.8, 0.9, 1H), 3.01 – 2.70 (m, 6H), 2.13 – 1.99 (m, 1H), 1.88 (m, 1H), 1.25 (m, 6H); ^{13}C NMR (100 MHz, $CDCl_3$, 25°C): δ = 171.6, 171.5, 61.3, 60.8, 47.6, 46.1, 34.1, 29.3, 29.1, 25.3, 14.1, 14.0; ESI-MS: m/z = 292 (53), 263 (2, M-Et), 218 (43), 158 (34), 145 (36), 119 (100); HRMS calcd for $C_{12}H_{20}O_4S_2$: 292.0803; found: 292.0796.

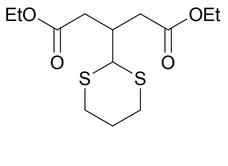

5f: yellowish oil, 49% yield; ^{1}H NMR (400 MHz, $CDCl_3$, 25°C): δ = 4.29 (d, J=5.3, 1H), 4.15 – 4.08 (m, 4H), 2.89 – 2.79 (m, 6H), 2.74 (dd, J=16.5, 5.7, 1H), 2.55 (dd, J=12.4, 6.4, 1H), 2.48 (dd, J=16.5, 7.3, 1H), 2.11 – 2.02 (m, 1H), 1.89 – 1.77 (m, 1H), 1.33 – 1.16 (m, 6H); ^{13}C NMR (100 MHz, $CDCl_3$, 25°C): δ = 172.0 (2C), 60.6 (2C), 51.1 (2C), 39.8, 36.0 (2C), 29.9, 25.9, 14.2 (2C); EI-MS: m/z = 306 (36), 261 (43) 218 (47), 145 874), 119 (100); HRMS calcd for $C_{13}H_{22}O_4S_2$: 306.0960; found: 306.0955.

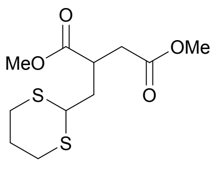

5g: colorless oil, 90% yield; ^{1}H NMR (400 MHz, $CDCl_3$, 25°C): δ = 3.99 (dd, J=8.0, 6.9, 1H), 3.68 (s, 3H), 3.64 (s, 3H), 3.18 – 3.08 (m, 1H), 2.86 – 2.75 (m, 4H), 2.68 (dd, J=16.5, 8.2, 1H), 2.54 (dd, J=16.5, 5.7, 1H), 2.19 (ddd, J=14.4, 7.7, 6.9, 1H), 2.05 (m, 1H), 1.89 (m, 2H); ^{13}C NMR (100 MHz, $CDCl_3$, 25°C): δ = 174.2, 171.6, 52.1, 51.8, 44.4, 38.4, 36.7, 35.6, 29.7, 29.6, 25.7; HRMS calcd for $C_{11}H_{18}O_4S_2$: 278.0647; found:

278.0639.

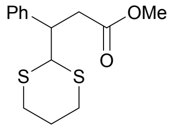

5h: yellowish oil, 79% yield; ^{1}H NMR (400 MHz, $CDCl_3$, 25°C): δ = 7.29 – 7.11 (m, 5H), 4.12 (d, *J*=8.7, 1H), 3.56 (s, 3H), 3.24 (dd, *J*=13.4, 4.2, 1H), 3.12 (m, 1H), 2.97 – 2.87 (m, 3H), 2.86 – 2.73 (m, 2H), 2.17 – 2.01 (m, 1H), 2.00 – 1.86 (m, 1H); ^{13}C NMR (100 MHz, $CDCl_3$, 25°C): δ = 172.6, 138.6, 128.8 (2C), 128.4 (2C), 126.5, 52.6, 51.7, 47.6, 36.4, 29.7, 28.9, 25.6; EI-MS: *m/z* = 282 (42), 191 (35), 159 (29), 119 (100), 91 (87); HRMS calcd for $C_{14}H_{18}O_2S_2$: 282.0748; found: 282.0741.

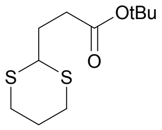

5i: colorless oil, 39% yield; ^{1}H NMR (400 MHz, $CDCl_3$, 25°C): δ = 4.03 (t, *J*=7.0, 1H), 2.88 – 2.73 (m, 4H), 2.43 (dd, *J*=8.0, 7.1, 2H), 2.11 – 1.97 (m, 3H), 1.92 – 1.81 (m, 1H), 1.43 (s, 9H); ^{13}C NMR (100 MHz, $CDCl_3$, 25°C): δ = 172.8, 77.2, 46.4, 32.5, 30.5, 29.9 (2C), 28.1 (3C), 25.8; HRMS calcd for $C_{11}H_{20}O_2S_2$: 248.0905; found: 248.0901.

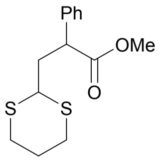

5j: yellowish oil; 90% yield; ^{1}H NMR (400 MHz, $CDCl_3$, 25°C): δ = 7.36 – 7.21 (m, 5H), 3.96 (t, *J*=7.6, 1H), 3.75 (dd, *J*=8.7, 6.3, 1H), 3.64 (s, 3H), 2.87 – 2.73 (m, 4H), 2.55 (ddd, *J*=14.2, 8.7, 7.6, 1H), 2.21 (ddd, *J*=14.2, 8.0, 7.6, 1H), 2.05 (m, 1H), 1.86 (m, 1H); ^{13}C NMR (100 MHz, $CDCl_3$, 25°C): δ = 173.6, 137.9, 128.8 (2C), 128.0 (2C), 127.5, 52.1, 48.1, 44.3, 38.4, 29.6, 29.4, 25.8; ESI-MS: *m/z* = 251.0 $[M-OMe]^+$, 283.0 $[M+H]^+$, 305.0 $[M+Na]^+$; HRMS calcd for $C_{14}H_{18}O_2S_2$: 282.0748; found: 282.0741.

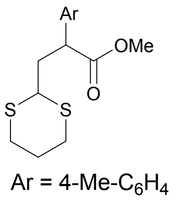

5k: yellowish oil, 88% yield; ^{1}H NMR (400 MHz, $CDCl_3$, 25°C): δ =7.21 – 7.15 (m, 2H), 7.15 – 7.09 (m, 2H), 3.92 (t, *J*=7.6, 1H), 3.75 (dd, *J*=8.7, 6.2, 1H), 3.64 (s, 3H), 2.90 – 2.65 (m, 4H), 2.52 (ddd, *J*=14.1, 8.7, 7.1, 1H), 2.19 (ddd, *J*=14.1, 8.1, 6.2, 1H), 2.31 (s, 3H), 2.12 – 1.97 (m, 1H), 1.94 – 1.78 (m, 1H); ^{13}C NMR (100 MHz, $CDCl_3$, 25°C): δ = 173.7, 137.2, 134.9, 129.5 (2C), 127.8 (2C), 52.1, 47.7, 44.3, 38.4, 29.6, 29.3, 25.8, 21.0; HRMS calcd for $C_{15}H_{20}O_2S_2$: 296.0905; found: 296.0899.

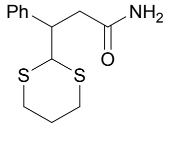

5l: sticky solid, 45% yield; ^{1}H NMR (400 MHz, $CDCl_3$, 25°C): δ = 7.31 – 7.15 (m, 5H), 5.32 (bs, 1H), 5.24 (bs, 1H), 4.27 (d, *J*=8.4, 1H), 3.24 (dd, *J*=13.5, 4.8, 1H), 2.99 – 2.82 (m, 5H), 2.68 (ddd, *J*=13.5, 8.5, 4.7, 1H), 2.18 – 2.07 (m, 1H), 1.97 – 1.82 (m, 1H); ^{13}C NMR (100 MHz, $CDCl_3$, 25°C): δ = 173.2, 138.8, 129.0 (2C), 128.6 (2C), 126.6, 54.0, 48.3, 36.3, 30.3, 29.7, 25.8; HRMS calcd for $C_{13}H_{17}NOS_2$: 267.0752; found: 267.0745.

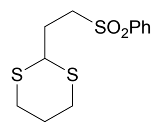

5m: sticky solid, 75% yield; ^{1}H NMR (400 MHz, $CDCl_3$, 25°C): δ = 7.94 – 7.86 (m, 2H), 7.71 – 7.61 (m, 1H), 7.61 – 7.52 (m, 2H), 4.00 (t, *J*=7.0, 1H), 3.37 – 3.21 (m, 2H), 2.90 – 2.72 (m, 4H), 2.28 – 2.12 (m, 2H), 2.14 – 1.97 (m, 1H), 1.91 – 1.74 (m, 1H); ^{13}C NMR (100 MHz, $CDCl_3$, 25°C): δ = 138.8, 133.8, 129.4 (2C), 128.0 (2C), 53.3, 44.9, 29.5 (2C), 28.4, 25.5; EI-MS: *m/z* = 288 (64), 146 (100), 119 (76), 77 (87); HRMS calcd for $C_{12}H_{16}O_2S_3$: 288.0312; found: 288.0306.

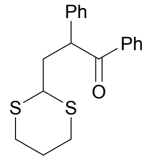

5n: colorless oil, 85% yield; ^{1}H NMR (400 MHz, $CDCl_3$, 25°C): δ = 8.18 – 7.88 (m, 2H), 7.63 – 6.99 (m, 8H), 4.98 (t, *J*=7.2, 1H), 3.83 (dd, *J*=8.2, 6.9, 1H), 2.97 – 2.57 (m, 5H), 2.24 (dt, *J*=14.1, 7.2, 1H), 2.13 – 1.94 (m, 1H), 1.94 – 1.74 (m, 1H); ^{13}C NMR (100 MHz, $CDCl_3$, 25°C): δ = 198.6, 138.4, 136.4, 132.9, 129.0 (2C), 128.8 (2C), 128.5 (2C), 128.3 (2C), 127.3, 50.0, 44.7, 38.8, 29.6, 29.5, 25.9; ESI-MS: *m/z* = 329.2 $[M+H]^+$, 351.0 $[M+Na]^+$; HRMS calcd for $C_{19}H_{20}OS_2$: 328.0956; found: 328.0947.

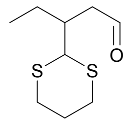

5o: colorless oil, 76% yield; ^{1}H NMR (400 MHz, $CDCl_3$, 25°C): δ = 9.72 (s, 1H), 4.13 (d, *J*=4.4, 1H), 2.88 – 2.66 (m, 4H), 2.40 (dd, *J*=17.6, 7.0, 1H), 2.35 – 2.21 (m, 1H), 2.12 – 1.93 (m, 1H), 1.86 – 1.70 (m, 1H), 1.69 – 1.52 (m, 1H), 1.52 – 1.44 (m, 1H), 1.43 – 1.29 (m, 1H), 0.86 (t, *J*=7.5, 3H); ^{13}C NMR (100 MHz, $CDCl_3$, 25°C): δ = 201.1, 52.0, 45.6, 39.5, 30.5, 30.4, 26.0, 24.7, 11.6; HRMS calcd for $C_9H_{16}OS_2$: 204.0643; found: 204.0638.

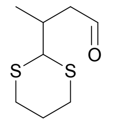

5p: colorless oil, 93% yield; ^{1}H NMR (400 MHz, $CDCl_3$, 25°C): δ = 9.75 (t, *J*=1.5, 1H), 4.08 (d, *J*=4.9, 1H), 2.87 – 2.82 (m, 4H), 2.80 (d, *J*=4.9, 1H), 2.63 – 2.50 (m, 1H), 2.44 (ddd, *J*=17.2, 8.0, 1.9, 1H), 2.17 – 2.00 (m, 1H), 1.93 – 1.71 (m, 1H), 1.13 (d, *J*=6.8, 3H); ^{13}C NMR (100 MHz, $CDCl_3$, 25°C): δ = 200.9, 53.9, 48.0, 33.1, 30.5, 30.3, 26.0, 17.8; HRMS calcd for $C_8H_{14}OS_2$: 190.0486; found: 150.0481.

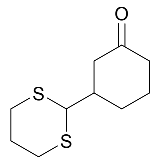

5q: colorless oil, 85% yield; ^{1}H NMR (400 MHz, $CDCl_3$, 25°C): δ = 4.07 (d, *J*=5.0, 1H), 2.97 – 2.75 (m, 4H), 2.54 (ddt, *J*=14.2, 4.2, 2.0, 1H), 2.48 – 2.32 (m, 2H), 2.30 – 2.03 (m, 5H), 1.92 – 1.76 (m, 1H), 1.74 – 1.51 (m, 2H); ^{13}C NMR (100 MHz, $CDCl_3$, 25°C): δ = 210.4, 53.5, 45.2, 43.5, 41.1, 30.7, 30.7, 28.4, 26.1, 24.8; HRMS calcd for $C_{10}H_{16}OS_2$: 216.0643; found: 216.0638.

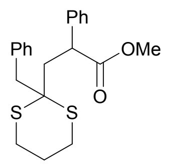

5r: The product was obtained using general procedure with **4b** instead of **4a**; yellowish oil, 89% yield; ^{1}H NMR (400 MHz, $CDCl_3$, 25°C): δ = 7.49 – 6.93 (m,

10H), 3.98 (dd, *J*=9.1, 2.4, 1H), 3.63 (s, 3H), 3.24 (dd, *J*=14.5, 8.7, 1H), 3.19 (d, *J*=12.8, 1H), 3.14 (d, *J*=14.2, 1H), 2.99 (ddd, *J*=14.5, 10.3, 2.9, 1H), 2.87 (ddd, *J*=14.2, 10.2, 3.0, 1H), 2.77 – 2.63 (m, 2H), 2.06 (dd, *J*=15.0, 2.4, 1H), 1.98 (m, 1H), 1.83 (m, 1H); ^{13}C NMR (100 MHz, $CDCl_3$, 25°C): δ = 174.5, 139.8, 135.1, 131.0 (2C), 128.7 (2C), 127.9 (2C), 127.8 (2C), 127.2, 127.0, 53.2, 52.3, 48.2, 45.9, 41.6, 26.9, 26.2, 24.4; HRMS calcd for $C_{21}H_{24}O_2S_2$: 372.1218; found: 372.1210.

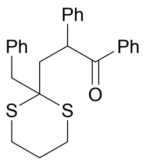

5s: The product was obtained using general procedure with **4b** instead of **4a**; colorless oil, 87% yield; ^{1}H NMR (400 MHz, $CDCl_3$, 25°C): δ = 8.03 – 7.91 (m, 2H), 7.57 – 7.43 (m, 1H), 7.41 – 7.34 (dd, *J*=8.3, 6.8, 2H), 7.31 – 7.20 (m, 9H), 7.17 – 7.10 (m, 1H), 5.03 (dd, *J*=9.1, 1.7, 1H), 3.61 (dd, *J*=14.9, 9.1, 1H), 3.32 (d, *J*=14.0, 1H), 3.29 (d, *J*=14.0, 1H), 3.02 – 2.82 (m, 2H), 2.87 (ddd, *J*=14.4, 6.9, 3.1, 1H), 2.63 (ddd, *J*=14.4, 6.8, 3.2, 1H), 2.10 (dd, *J*=14.9, 1.7, 1H), 2.02 – 1.87 (m, 1H), 1.9 – 1.78 (m, 1H); ^{13}C NMR (100 MHz, $CDCl_3$, 25°C): δ = 198.7, 139.7, 137.1, 135.4, 132.5, 131.1 (2C), 128.9 (2C), 128.8 (2C), 128.4 (2C), 128.3 (2C), 127.9 (2C), 127.0, 126.9, 53.6, 49.8, 46.8, 42.2, 26.9, 26.3, 24.5; HRMS calcd for $C_{26}H_{26}OS_2$: 418.1425; found: 418.1416.

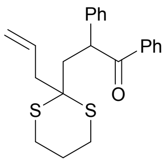

5t: The product was obtained using general procedure with **4c** instead of **4a**; yellowish oil, 86% yield; ^{1}H NMR (400 MHz, $CDCl_3$, 25°C): δ = 8.03 – 7.93 (m, 2H), 7.51 – 7.43 (m, 1H), 7.42 – 7.34 (m, 2H), 7.34 – 7.29 (m, 2H), 7.28 – 7.20 (m, 2H), 7.20 – 7.11 (m, 1H), 5.97 – 5.79 (m, 1H), 5.18 – 5.12 (m, 1H), 5.12 – 5.07 (m, 1H), 4.99 (dd, *J*=8.9, 1.9, 1H), 3.54 (dd, *J*=14.9, 8.9, 1H), 2.87-2.81 (m, 2H), 2.78 – 2.67 (m, 1H), 2.67 – 2.55 (m, 3H), 2.10 (dd, *J*=14.9, 1.9, 1H), 1.96-1.93 (m, 1H), 1.91 – 1.78 (m, 1H); ^{13}C NMR (100 MHz, $CDCl_3$, 25°C): δ = 198.6, 140.0, 137.1, 132.6, 132.4, 129.0 (2C), 128.7 (2C), 128.4 (2C), 128.2 (2C), 127.0, 118.8, 52.6, 49.7, 44.3, 42.2, 26.5, 26.0, 24.7; HRMS calcd for $C_{22}H_{24}OS_2$: 368.1269; found: 368.1260.

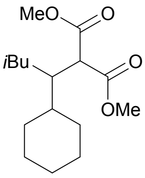

14: The product was obtained using general procedure with cyclohexylcarboxylic acid instead of **4a** and catalyst **1** replaced **2**; colorless oil, 46% yield; ^{1}H NMR (400 MHz, $CDCl_3$, 25°C): δ = 3.69 (d, *J*=5.9, 6H), 3.45 (d, *J*=7.5, 1H), 2.14 – 2.07 (m, 1H), 1.76 – 1.56 (m, 5H), 1.38 – 1.25 (m, 3H), 1.21 – 1.01 (m, 6H), 0.85 (d, *J*=4.0, 3H), 0.83 (d, *J*=4.0, 3H); ^{13}C NMR (100 MHz, $CDCl_3$, 25°C): δ =170.0, 169.9, 54.4, 52.3, 52.1, 41.7, 40.7, 38.2, 30.5, 29.20, 26.9, 26.8, 26.6, 26.5, 22.8, 22.6.

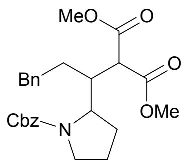

16: The product was obtained using general procedure with *rac*-Cbz-proline instead of **4a**; sticky solid, 87% yield; The molecules present different diastereoisomer and different conformers, NMR signal appears very broad and some ^{13}C NMR signals were missed. ^{1}H NMR (400 MHz, $CDCl_3$, 25°C): δ = 7.56 – 6.76 (m, 10H), 5.11 (bs, 2H), 4.22 – 3.91 (m, 1H), 3.75 – 3.50 (m, 9H), 3.19 (m, 1H), 3.09 (dq, *J*=8.5, 4.3, 1H), 2.80 – 2.37 (m, 3H), 2.07 – 1.90 (m, 1H), 1.90 – 1.59 (m, 3H); ^{13}CNMR (100 MHz, $CDCl_3$, 25°C): δ = 169.2, 155.6, 141.8, 136.6, 128.4 (2C), 128.3 (2C) 128.2 (2C), 128.1, (2C), 127.9 (2C), 125.8, 66.8, 60.3, 58.7, 52.3, 48.2, 46.9, 40.6, 31.9, 29.7, 27.0; ESI-MS: *m/z* = 454.2 $[M+H]^+$, 476.2 $[M+Na]^+$.

Reductive removal of the dithiane moiety

General Procedure: To a solution of **5a** (0.1 mmol, 0.032 g) in methanol (2 mL), Ni-Raney (0.450 g, slurry in water) was added and the reaction was keep under H_2 atmosphere (1 atm). After 3h the reaction mixture was filtered through a Celite® pad washed with DCM and the organic solvent was removed under reduce pressure. The residue was diluted with ethyl acetate (10 mL), the organic layer was separated, and the aqueous layer was extracted with ethyl acetate (2 x 5 mL). The collected organic layers were washed with brine (5 mL), dried over Na_2SO_4 and concentrated under reduce pressure.

Flash chromatography (cyclohexane:diethyl ether, 95:5) of the residue give **6** in 91% yield (0.091 mmol, 0.02 g).

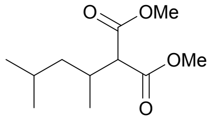

6: yellowish oil, 90% yield; ^{1}H NMR (400 MHz, $CDCl_3$, 25°C): δ = 3.71 (s, 6H), 3.22 (d, *J*=7.8, 1H), 2.35 – 2.24 (m, 1H), 1.61 (m, 1H), 1.15 – 1.05 (m, 2H), 0.94 (d, *J*=6.7, 3H), 0.87 (d, *J*=6.5, 3H), 0.85 (d, *J*=6.5, 3H); ^{13}C NMR (100 MHz, $CDCl_3$, 25°C): δ = 169.3 (2C), 57.8, 52.2, 52.1, 31.4, 29.7, 25.2, 23.7, 21.3, 16.9; EI-MS: *m/z* = 201 (6, M-Me), 185 (10, M-OMe), 132 (100), 100 (56), 83 (45), 69 (50); HRMS calcd for $C_{11}H_{20}O_4$: 216.1362; found: 216.1351.

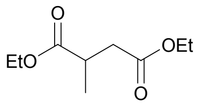

7: yellowish oil, 84% yield; Spectral proprieties were in according with literature.[9] ^{1}H NMR (400 MHz, $CDCl_3$, 25°C): δ = 4.24 – 4.04 (m, 4H), 2.95 – 2.81 (m, 1H), 2.70 (dd, *J*=16.4, 8.1, 1H), 2.37 (dd, *J*=16.4, 6.1, 1H), 1.26 – 1.21 (m, 6H), 1.20 (d, *J*=7.1, 3H); ^{13}C NMR (100 MHz, $CDCl_3$, 25°C): δ = 175.2, 171.8, 60.5, 60.4, 37.7, 35.8, 16.9, 14.1, 14.0.

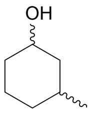

8: yellowish oil, 88% yield; Spectral proprieties were in according with literature.[10]

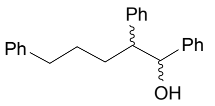

9: yellowish oil, 90% yield; ^{1}H NMR (400 MHz, $CDCl_3$, 25°C): δ = 7.38 – 7.23 (m, 4H), 7.27 – 7.09 (m, 6H), 7.13 – 7.02 (m, 3H), 7.05 – 6.91 (m, 2H), 4.68 (d, *J*=8.4, 1H), 2.97 – 2.80 (m, 1H), 2.47 (ddd, *J*=14.5, 8.6, 6.4, 1H), 2.34 (ddd, *J*=14.5, 8.8, 6.9, 1H), 1.67 – 1.53 (m, 1H), 1.49 – 1.24 (m, 3H); ^{13}C NMR (100 MHz, $CDCl_3$, 25°C): δ = 142.7, 142.2, 141.1, 128.8 (2C), 128.6 (2C), 128.3 (2C), 128.2 (2C), 128.1 (2C), 127.8, 127.0, 126.9 (2C), 126.4, 125.5, 78.8, 54.2, 35.6, 29.1; ESI-MS: *m/z* = 317.1 $[M+H]^+$, 339.2 $[M+Na]^+$; HRMS calcd for $C_{23}H_{24}O$: 316.1827; found: 316.1821.

Hydrolysis of the dithiane moiety

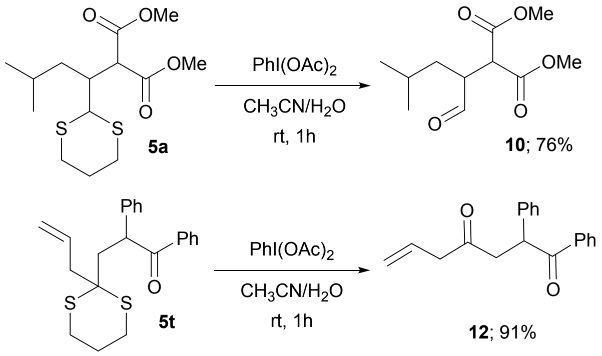

PhI(OAc)$_2$ method:[11] Bis(trifluoroacetoxy)iodobenzene (52 mg, 0.16 mmol) was added at 0°C to a stirred solution of **5a** or **5t** (0.1 mmol), water (1 mL) and CH_3CN (9 mL). After it was stirred at room temperature for 30 min, the reaction was quenched with $NaHCO_3$ sat. sln. (10 ml) and the volatiles were evaporated. The residue was dissolved in ethyl acetate (20 mL) and the organic layer was separated. The aqueous layer was extracted with ethyl acetate (3 x 10 mL). The collected organic layers were washed with brine (10 mL), dried over Na_2SO_4 and concentrated under reduced pressure to give the crude product. Column chromatography on silica (cyclohexane:ethyl acetate from 95:5 to 80:20) afforded pure products.

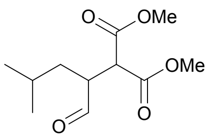

10: yellowish oil, 76% yield; ^{1}H NMR (400 MHz, $CDCl_3$, 25°C): δ = 9.77 (d, *J*=1.2, 1H), 3.75 (s, 3H), 3.73 (d, *J*=7.8, 1H), 3.72 (s, 3H), 3.17 – 3.01 (m, 1H), 1.62 – 1.64 (m, 1H), 1.37 – 1.20 (m, 2H), 0.94 (d, *J*=6.3, 3H), 0.91 (d, *J*=6.2, 3H); ^{13}C NMR (50 MHz, $CDCl_3$, 25°C): δ = 198.0, 168.5 (2C), 52.8, 52.1, 48.3, 36.5, 29.7, 25.9, 22.9, 22.1; HRMS calcd for $C_{11}H_{18}O_5$: 230.1154; found: 230.1149.

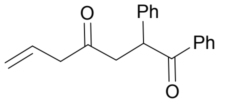

12: yellowish oil, 91% yield; ^{1}H NMR (400 MHz, $CDCl_3$, 25°C): δ = 8.08 – 7.76 (m, 2H), 7.47 – 7.42 (m, 1H), 7.39 – 7.31 (m, 2H), 7.30 – 7.21 (m, 4H), 7.21 – 7.15 (m, 1H), 5.89 (m, 1H), 5.16 (m, 2H), 5.13 – 5.06 (m, 1H), 3.59 (dd, J=18.0, 10.0, 1H), 3.27 – 3.13 (m, 2H), 2.76 (dd, J=18.0, 4.0, 1H); ^{13}C NMR (100 MHz, $CDCl_3$, 25°C): δ = 206.7, 198.8, 138.5, 136.3, 132.9, 130.2, 129.1 (2C), 128.9 (2C), 128.4 (2C), 128.1 (2C), 127.3, 119.0, 48.7, 47.7, 46.8; ESI-MS: m/z = 279.0 $[M+H]^+$, 301.0 $[M+Na]^+$; HRMS calcd for $C_{19}H_{18}O_2$: 278.1307; found: 278.1302.

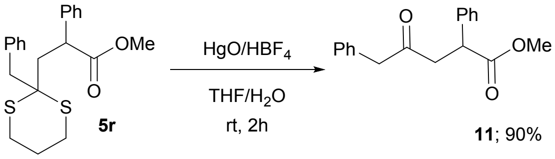

HgO/HBF$_4$ method: To a suspension of HgO (0.18 mmol, 0.039 g) in HBF_4 (48% in water, 0.2 mL) and THF (1 mL) a solution of **5r** (0.09 mmol, 0.034 g) in THF (1 mL) was added. After 2 hours the reaction was quenched adding 10 mL of $NaHCO_3$ sat. sln. until basic pH. The mixture was filtered through a Celite® pad washing with ethyl acetate. Water (10 mL) was added and the organic layer was separated. The aqueous layer was extracted with ethyl acetate (3 x 10 mL). The collected organic layers were washed with brine (10 mL), dried over Na_2SO_4 and concentrated under reduced pressure to give the crude product. Column chromatography on silica (cyclohexane:ethyl acetate from 95:5 to 90:10) afforded pure product **11** (90%, 0.08 mmol, 0.021 g) as yellowish oil.

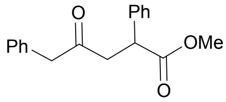

11: ^{1}H NMR (400 MHz, $CDCl_3$, 25°C): δ = 7.49 – 6.86 (m, 10H), 4.07 (dd, J=10.2, 4.5, 1H), 3.70 (s, 2H), 3.62 (s, 3H), 3.36 (dd, J=18.0, 10.2, 1H), 2.71 (dd, J=18.0, 4.5, 1H); ^{13}C NMR (100 MHz, $CDCl_3$, 25°C): δ = 205.9, 173.6, 138.0, 133.7, 129.4 (2C), 128.8 (2C), 128.7 (2C), 127.7 (2C), 127.5, 127.1, 52.3, 50.1, 46.2, 45.5; HRMS calcd for $C_{18}H_{18}O_3$: 282.1256; found: 282.1251.

Reaction in presence of TEMPO

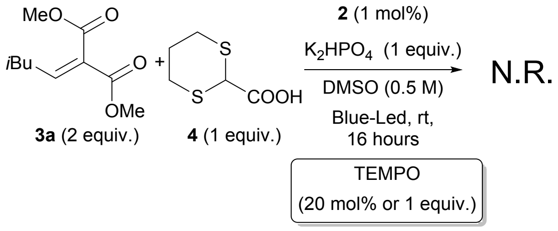

Evidence of a possible radical mechanism was determinate by performing the reaction in the presence of radical scavengers like TEMPO (2,2,6,6-tetramethylpiperidine 1-oxyl).
Two different experiments were conducted in the presence of a catalytic (20 mol%) and stoichiometric amount of TEMPO. In both cases no formation of product or adduct between TEMPO and reaction intermediates were observed.

Preparation of ammonium salts 17, 18, 19

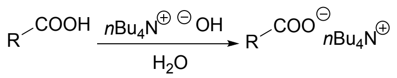

General procedure:[12] To a solution of **4a** (0.3 mmol, 0.05g) in water (5 mL) nBu$_4$NOH*30H$_2$O (0.3 mmol, 0.240 g) was added and the reaction mixture was heated at 60°C for 2 hours. Water was evaporated under reduced pressure and the residue was dried under high vacuum for 2 hours to give pure **17** (96%, 0,29 mmol, 0.117 g) as white solid.

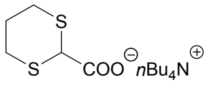

17: sticky solid, quantitative; ^{1}H NMR (400 MHz, CDCl$_3$, 25°C): δ = 4.70 (s, 1H), 3.45 – 3.23 (m, 8H), 2.99 (ddd, J=14.0, 6.3, 3.0, 2H), 2.75 (ddd, J=13.7, 10.5, 2.7, 2H), 2.08 – 1.94 (m, 1H), 1.90 – 1.76 (m, 1H), 1.68 – 1.55 (m, 8H), 1.53 – 1.33 (m, 8H), 0.99 (t, J=7.3, 12H); ^{13}C NMR (100 MHz, CDCl$_3$, 25°C): δ = 170.6, 58.5 (4C), 52.9, 30.2 (2C), 26.2, 23.9 (4C), 19.6 (4C), 13.6 (4C).

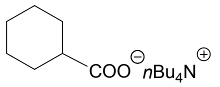

18: sticky solid, quantitative; ^{1}H NMR (400 MHz, CDCl$_3$, 25°C): δ = 3.46 – 3.17 (m, 8H), 2.90 (bs, 1H), 2.11 – 2.00 (m, 1H), 1.95 – 1.85 (m, 2H), 1.77 – 1.54 (m, 10H), 1.51 – 1.30 (m, 10H), 1.31 – 1.10 (m, 3H), 0.97 (t, J=7.3, 12H); ^{13}C NMR (100 MHz, CDCl$_3$, 25°C): δ = 58.9 (4C), 48.0, 31.0 (2C), 26.6 (2C), 24.2 (4C), 19.9, 19.8 (4C), 13.7 (4C), carboxylic carbon was not detected also using long acquisition time with 4s delay.

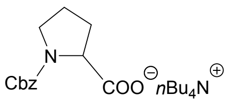

19: sticky solid, quantitative; The compound is present as two different conformers (A and B). ^{1}H NMR (400 MHz, $CDCl_3$, 25°C): δ = 7.42 – 7.01 (m, 10H, A+B), 5.20 (d, J=13.2, 1H), 5.11 (d, J=12.7, 1H, B), 4.99 (d, J=13.0, 1H, B), 4.27 (t, J=5.9, 1H, A), 4.15 (dd, J=7.8, 3.1, 2H, A), 3.74 – 3.58 (m, 2H, B), 3.42 (td, J=17.5, 8.1, 2H, A), 3.35 – 3.16 (m, 8H, A+B), 2.26 – 2.00 (m, 2H, A+B), 1.81 – 1.67 (m, 2H, A+B), 1.68 – 1.54 (m, 8H, A+B), 1.47 – 1.27 (m, 8H, A+B), 0.96 (t, J=7.3, 12H, A+B); ^{13}C NMR (100 MHz, $CDCl_3$, 25°C): δ =171.2 (A), 171.1 (B), 154.7 (A+B), 128.4 (B, 2C), 128.3 (A, 2C), 127.9 (A, 2C), 127.6 (B, 2C), 127.4 (A), 127.2 (B), 66.9 (A), 66.5 (B), 58.8 (A+B, 4C), 46.8 (A), 46.4 (B), 30.6 (B), 29.6 (A), 24.4 (A), 24.1 (B), 24.0 (A+B, 4C), 23.3 (A), 23.2 (B), 23.1 (C), 19.6 (A+B, 4C), 13.6 (A+B, 4C), carboxylic carbon was not detected also using long acquisition time with 4s delay.

Photoredox radical conjugate addition of salt 17

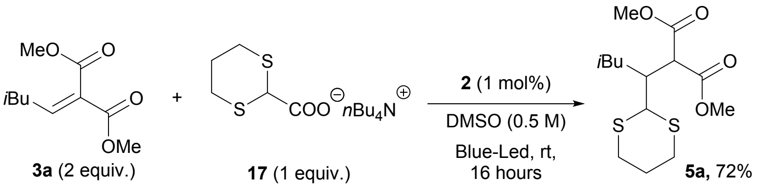

To determinate if tetrabutylammonium salt **17** is a suitable substrate for the photoredox radical conjugate addition we tested it in the reaction using general procedure without the presence of K_2HPO_4 as base. Product **5a** was obtained in 72% of yield, demonstrating the possibility to use tetrabutylammonium carboxylate as substrate.

Photostability of Ir(III) complex 2

In order to test its photostability during the reaction, complex **2** was dissolved in degassed deuterated DMSO and was irradiated using blue light LED for 24 hours in order to replicate the reaction conditions. Any possible photolysis was monitored by ^{1}H NMR spectroscopy. As reported in Figure S1, less than 5% of complex **2** undergoes to photochemical decomposition after 24 hours of irradiation, revealing its good photostability under reaction conditions, differently from complex **1**.[13]

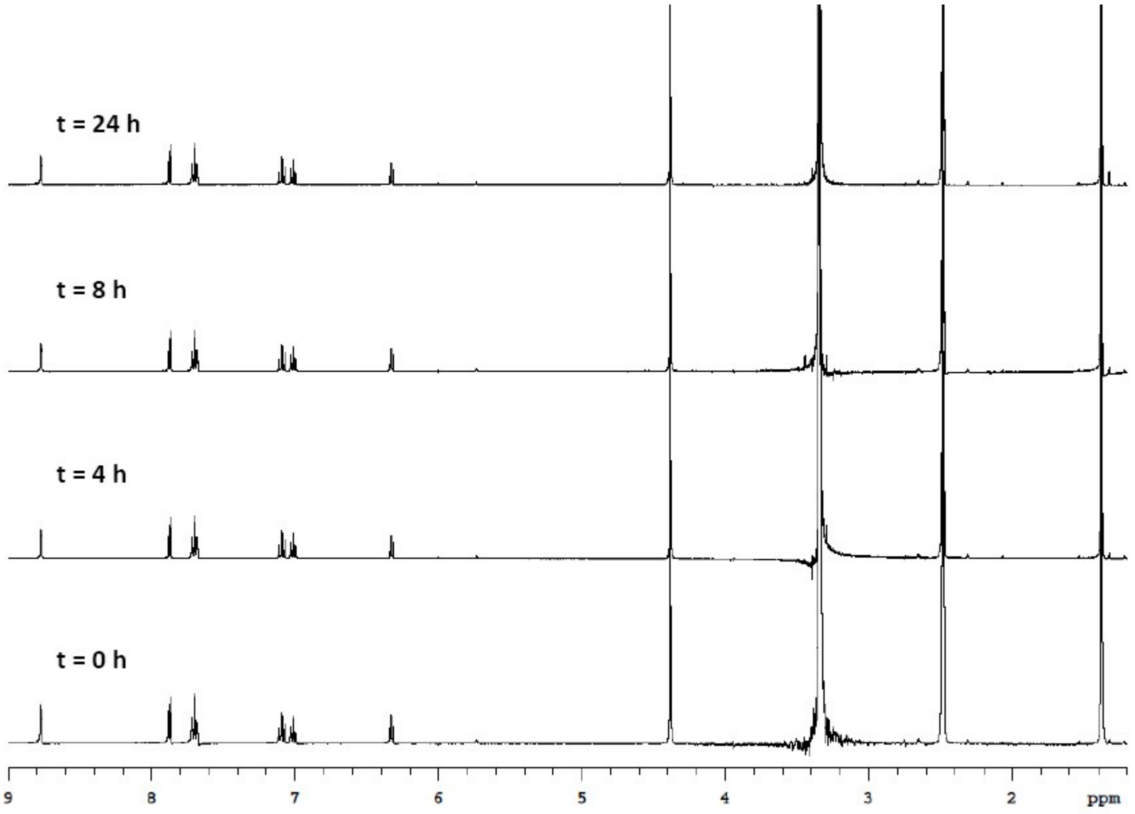

Figure S1. ^{1}H NMR spectra for complex **2** in deuterated DMSO solution irradiated with blue light LED for increasing times. The solution was degassed by nitrogen gas for 15 minutes.

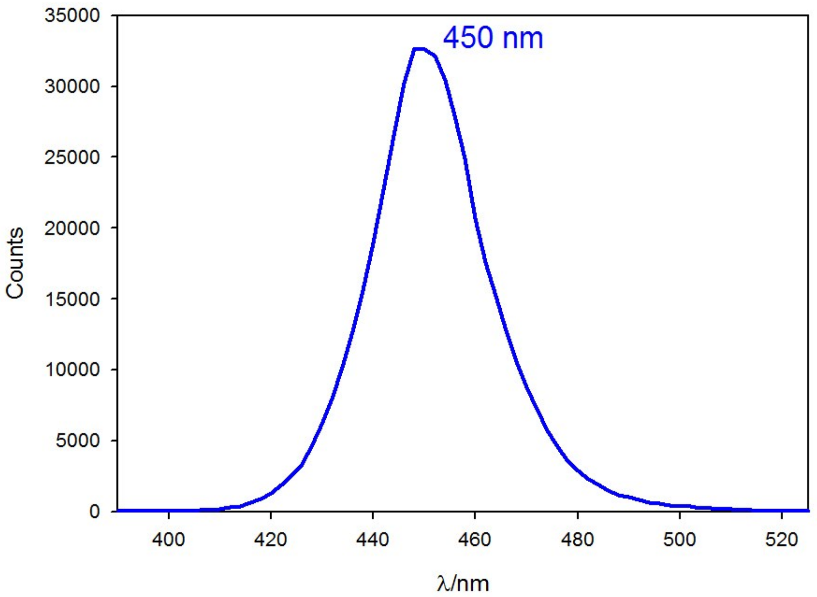

Figure S2. Emission profile of the 24W Blue LED strip used to irradiate the solutions.

Photophysical and electrochemical data

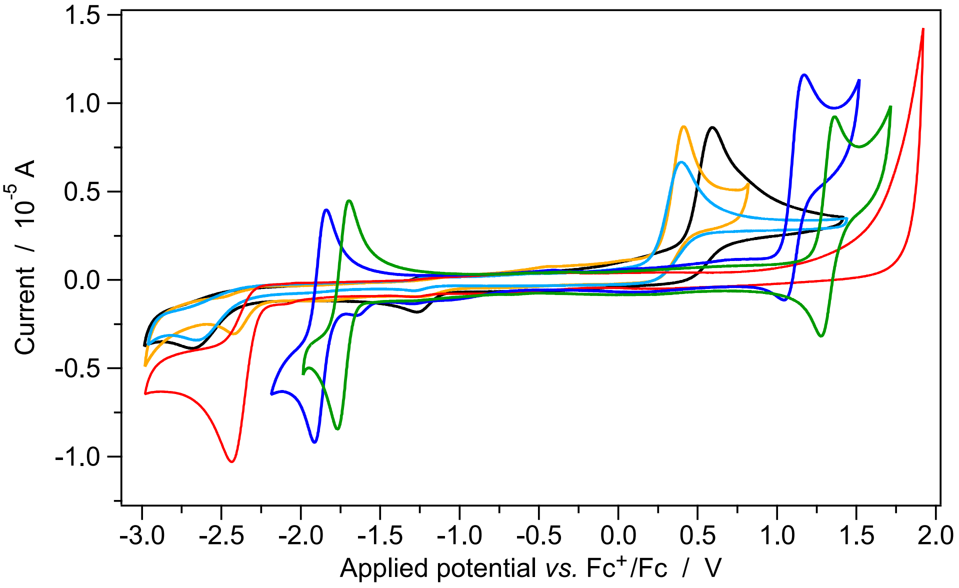

Figure S3. Cyclic voltammograms of the **17** (orange), **18** (black) and **19** (light blue) substrates, together with the one of the Michael acceptor **3a** (red) and of the iridium(III) photocatalysts **1** (green) and **2** (dark blue). The voltammograms are recorded at a scan rate of 200 mV s^{-1} in room-temperature acetonitrile in the presence of $TBAPF_6$ 0.1 M as supporting electrolyte.

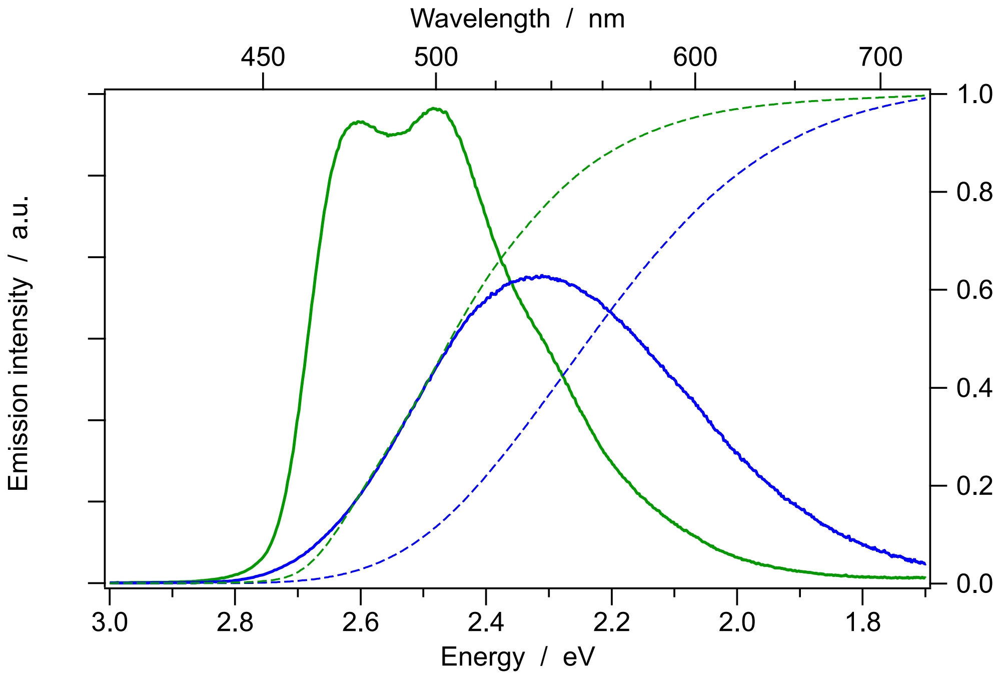

Figure S4. Full lines: corrected and normalized emission spectra of photocatalyst **1** (green) and **2** (dark blue) in room-temperature acetonitrile solution, reported in relative quanta per energy interval. Dashed lines: integrals of the spectra; the mean photon energy is calculated as the value at which the integral is 50%.

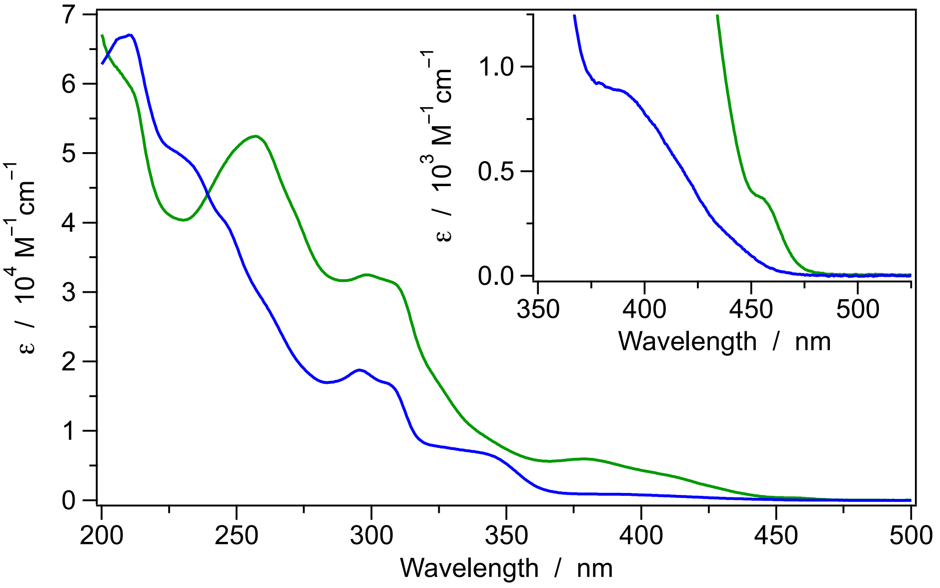

Figure S5. Absorption spectra of photocatalyst **1** (green) and **2** (dark blue) in room-temperature acetonitrile solution. The lowest-energy electronic transition, attributed to the direct population of the emitting state, is magnified in the inset for each of the two iridium(III) photocatalysts.

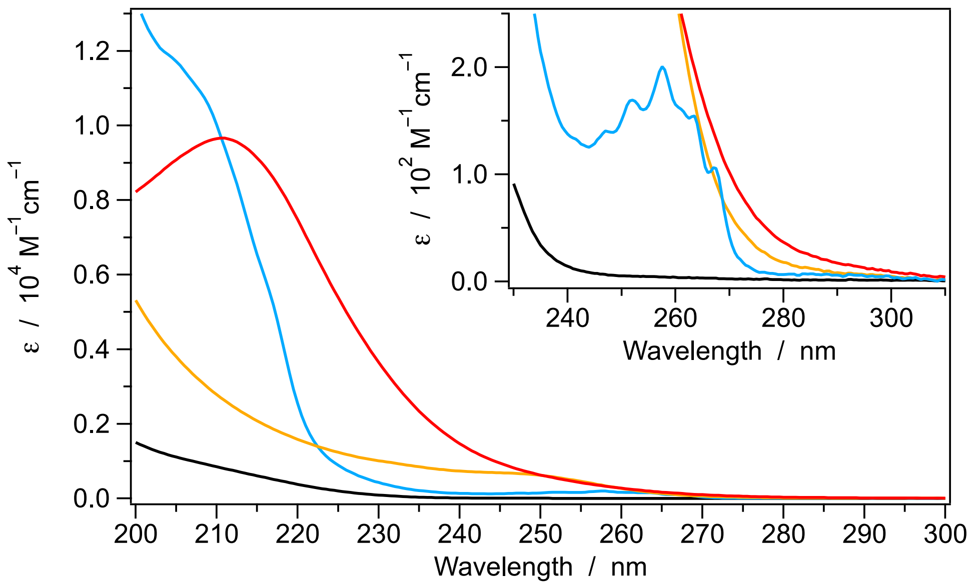

Figure S6. Absorption spectra of the **17** (orange), **18** (black) and **19** (light blue) substrates, together with the one of the Michael acceptor **3a** (red), in room-temperature acetonitrile solution. The lowest-energy transitions are magnified in the inset for the sake of clarity.

Determination of quantum yield

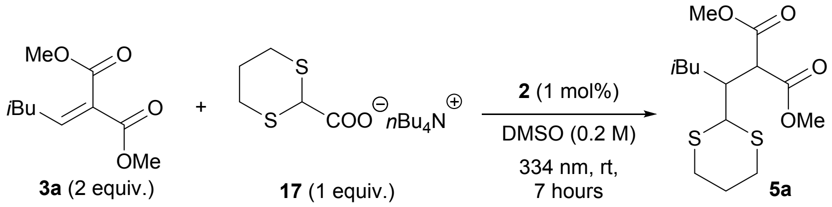

The reaction was carried out in spectrofluorimetric grade DMSO and placed in a Suprasil® quartz cuvette with a 2.00 mm path length. The cuvette was charged with the ammonium carboxylate **17** (81 mg, 0.20 mmol, 1 equiv), the Michael acceptor **3a** (80 mg, 0.40 mmol, 2 equiv), the iridium photocatalyst **2** (1.7 mg, 0.0020 mmol), and 500 µl of DMSO. Before irradiating the sample, dissolved oxygen was removed from the cuvette by argon bubbling for 15 minutes. The reaction mixture was excited at 334 nm for exactly 7 hours, using a 100 W Hg lamp equipped with an appropriate dichroic filter. During this process, the solution was stirred and kept at constant temperature.

The photon flux coming from the lamp to the sample in the cuvette was estimated using the ferrioxalate actinometer (photon flux: 1.97 nmol/s).[14] This experimental value was also confirmed by using a calibrated silicon diode (light power: 0.6 mW, corresponding to a photon flux of 2 nmol/s at 334 nm). In our experimental conditions, all the incident light is quantitatively absorbed by the iridium photocatalyst (A > 3 at 334 nm).

The product formation was determined by ^{1}H-NMR based on the relative conversion of the carboxylate derivative.

After irradiation, the estimated conversion was 7%, corresponding to 14 µmol of product formed.

$$\Phi = \frac{moles\ of\ product\ formed}{moles\ of\ photons\ absorbed} = \frac{14 \cdot 10^{-6} mol}{1.97 \cdot 10^{-9} \frac{mol}{s} \cdot 2.5 \cdot 10^{4}\, s} = 0.28 \pm 0.05$$

References

1 E. Massolo, M. Benaglia, A. Genoni, R. Annunziata, G. Celentano and N. Gaggero, *Org. Biomol. Chem.*, 2015, **13**, 5591-5596.

2 M. S. Lowry, J. I. Goldsmith, J. D. Slinker, R. Rohl, R. A. Pascal Jr., G. G. Malliaras and S. Bernhard, *Chem. Mater.*, 2005, **17**, 5712-5719.

[3] F. Monti, A. Baschieri, I. Gualandi, J. J. Serrano-Pérez, J. M. Junquera-Hernández, D. Tonelli, A. Mazzanti, S. Muzzioli, S. Stagni, C. Roldan-Carmona, A. Pertegás, H. J. Bolink, E. Ortí, L. Sambri and N. Armaroli, *Inorg. Chem.*, 2014, **53**, 7709-7721.

[4] M. Felici, P. Contreras-Carballada, Y. Vida, J. M. M. Smits, R. J. M. Nolte, L. De Cola, R. M. Williams, M. C. Feiters, *Chem. Eur. J.*, 2009, **15**, 13124–13134.

[5] G. Cardillo, S. Fabbroni, L. Gentilucci, M. Gianotti and A. Tolomelli, *Synth. Comm.* 2003, **33**, 1587-1594.

[6] a) G. Cardillo, L. Gentilucci, M. Gianotti, R. Perciaccante and A. Tolomelli, *J. Org. Chem.* 2001, **66**, 8657-8660; b) P. Ramesh, B. Shalini and N. W. Fadnavis, *RSC Adv.,* 2014, **4**, 7368-7373

[7] a) M. J. Lavecchia, R. Puig de la Bellacasa, J. I. Borrell and C. N. Cavasotto, *Biorg. Med. Chem.*, 2016, **24**, 768-778; b) J. Pietruszka and M. Schölzel, *Adv. Synth. Catal.*, 2012, **354**, 751-756.

[8] V. Gembus, J.-J. Bonnet, F. Janin, P. Bohn, V. Levacher and J.-F. Briere, *Org. Biomol. Chem.*, 2010, **8**, 3287-3293.

[9] Y. Chen, E. Kervio and J. Retey, *Helv. Chim. Acta*, 2002, **85**, 552-558.

[10] K. Kamata, K. Yonehara, Y. Nakagawa, K. Uehara and N. Mizuno, *Nature Chemistry* 2010, **2**, 478–483.

[11] Z. Meng and S. Danishefsky, *Angew. Chem. Int. Ed.*, 2005, **44**, 1511-1513.

[12] C. R. Allen, P. L. Richard, A. J. Ward, L. G. A. van de Water, A. F. Masters and T. Maschmeyer, *Tetrahedron Letters*, 2006, **47**, 7367-7370

[13] C. Yang, F. Mehmood, T. L. Lam, S. L.-F. Chan, Y. Wu, C.-S. Yeung, X. Guan, K. Li, C. Y.-S. Chung, C.-Y. Zhou, T. Zou, and C.-M. Che, *Chem. Sci.* **2016**, *7*, 3123-3136.

[14] M. Montalti, A. Credi, L. Prodi , and M. T. Gandolfi, Handbook of Photochemistry, Third Edition, CRC Press, 2006, chapter 12.

Copies of NMR spectra

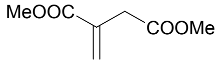

3g

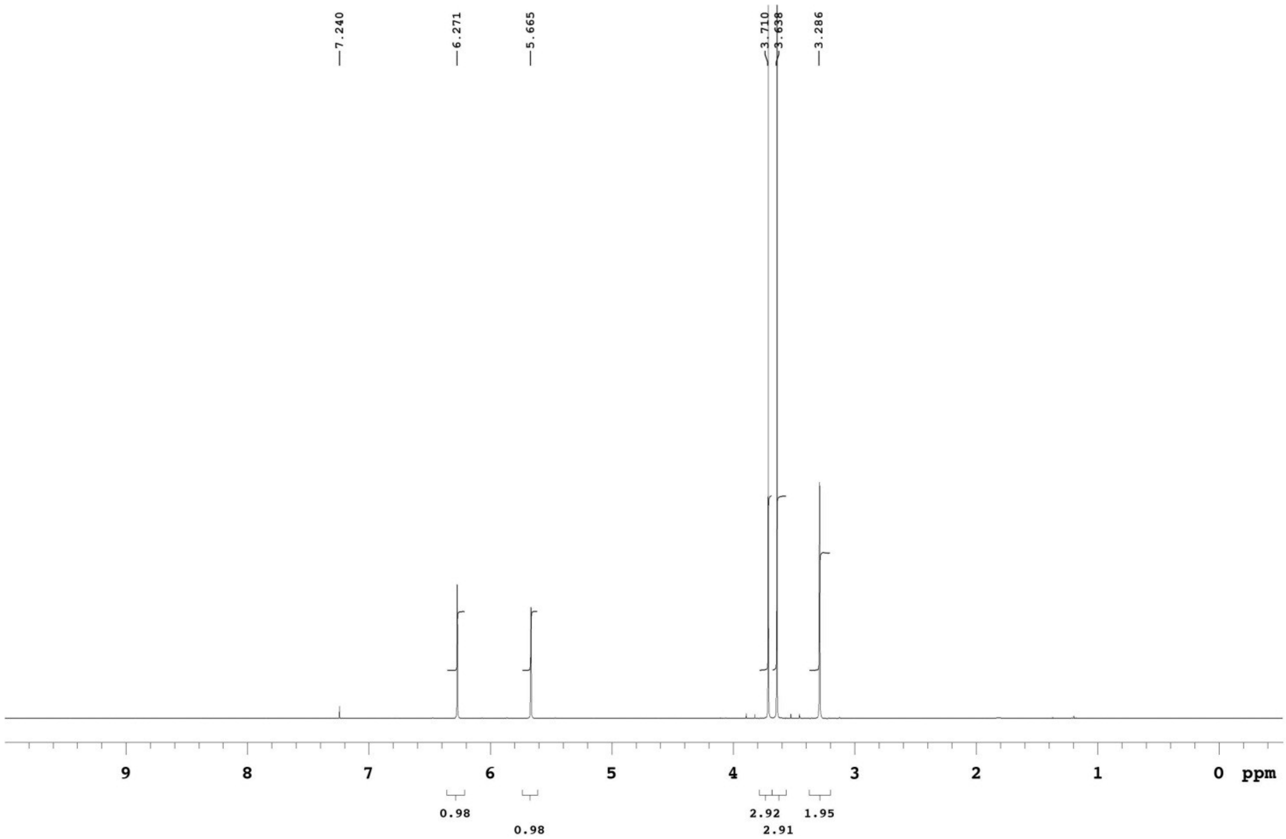

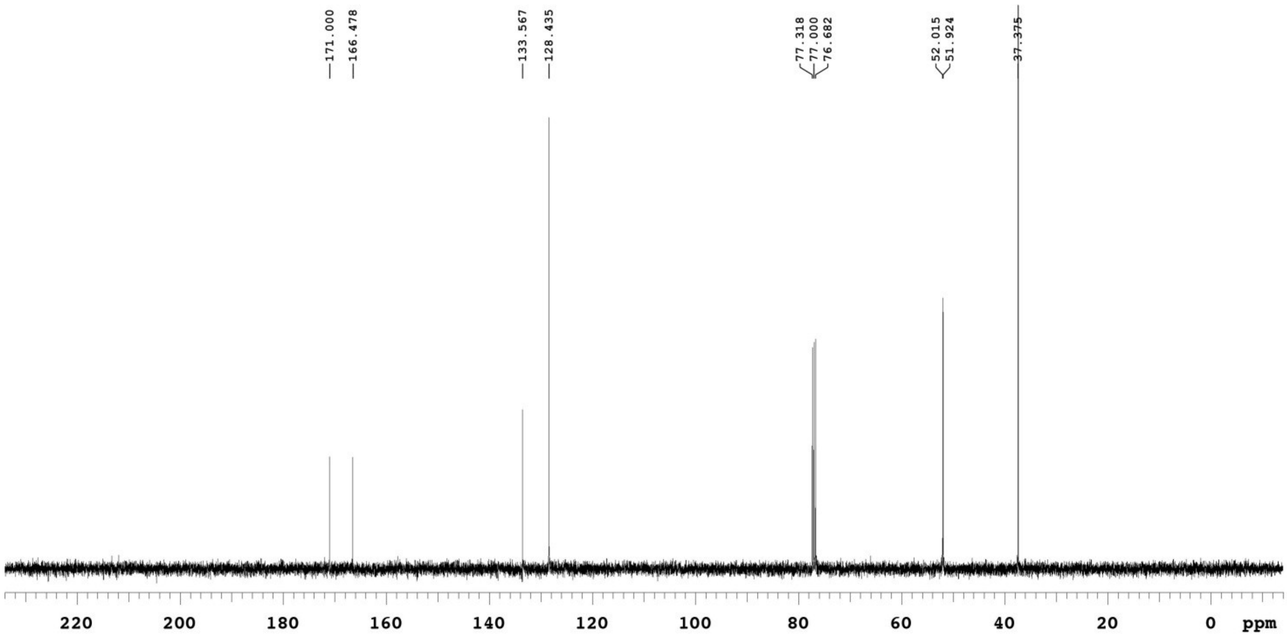

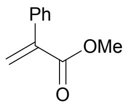
Ph
OMe
O

3j

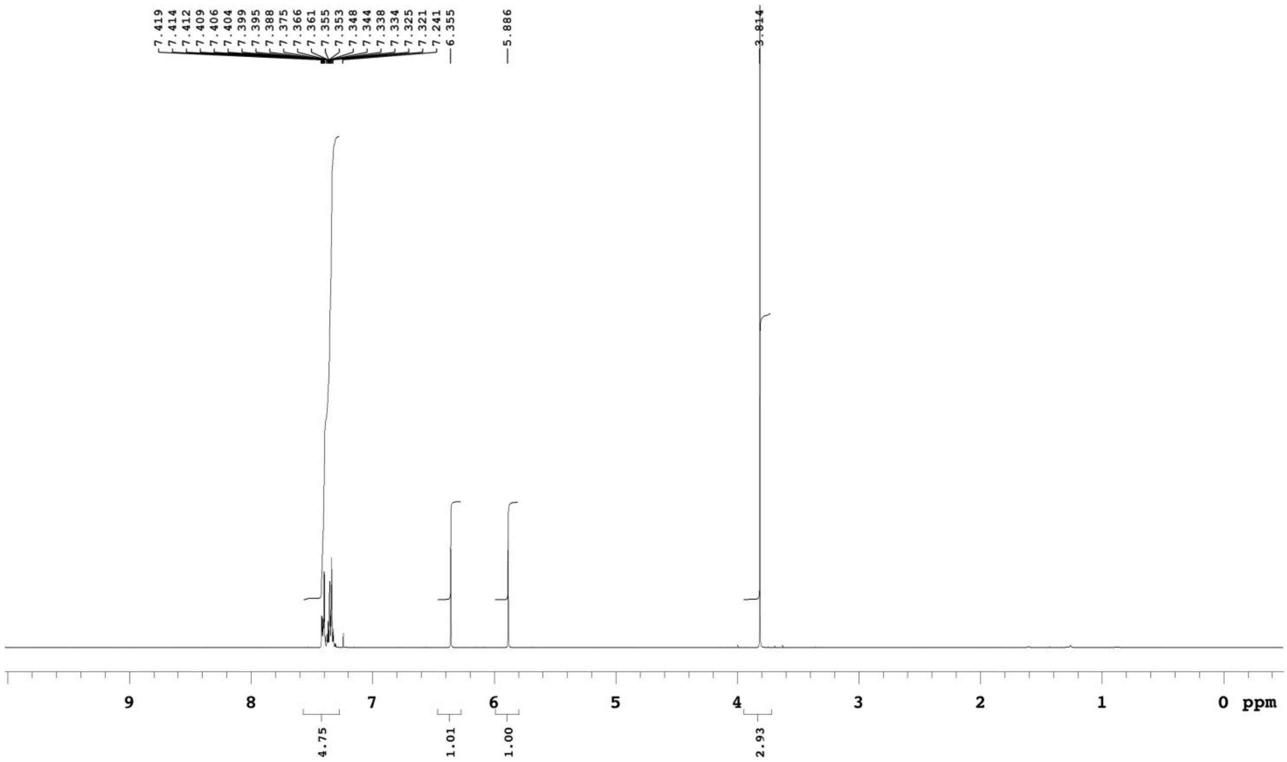
7.419
7.414
7.412
7.409
7.406
7.404
7.399
7.395
7.388
7.375
7.366
7.361
7.355
7.353
7.348
7.344
7.338
7.334
7.325
7.321
7.241
6.355
5.886
3.814
9
8
7
6
5
4
3
2
1
0 ppm
4.75
1.01
1.00
2.93

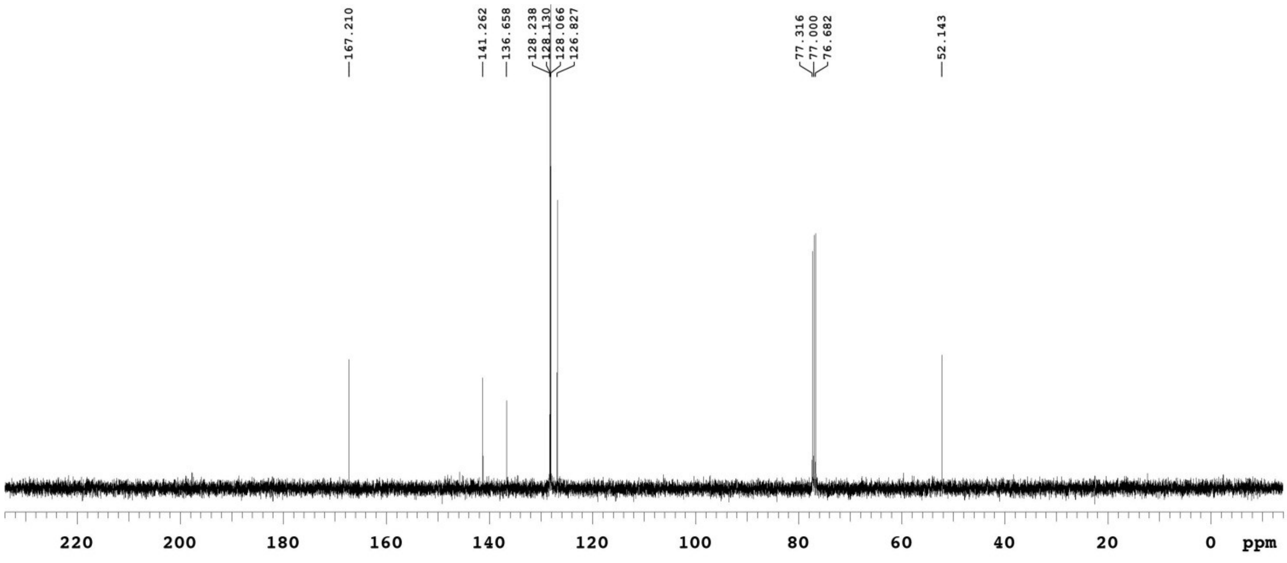
167.210
141.262
136.658
128.238
128.130
128.066
126.827
77.316
77.000
76.682
52.143
220
200
180
160
140
120
100
80
60
40
20
0 ppm

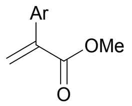

Ar = 4-Me-C_6H_4 **3k**

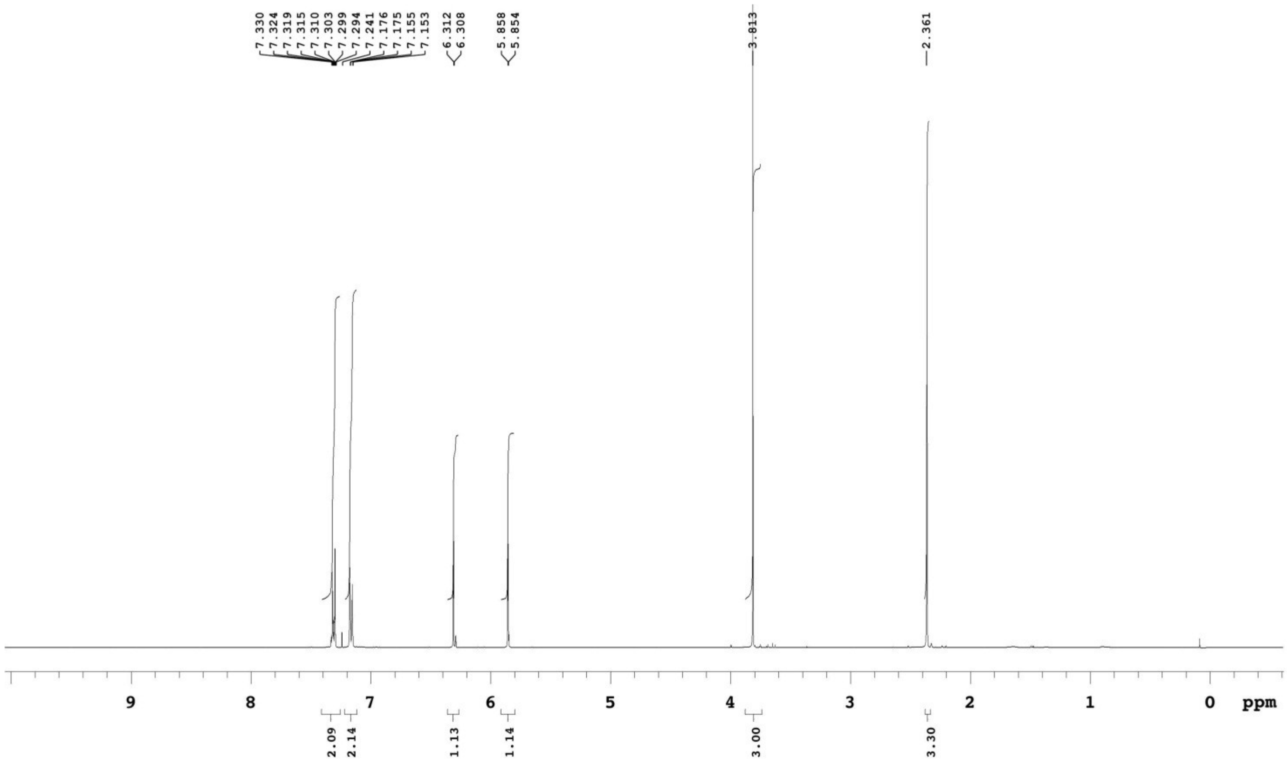

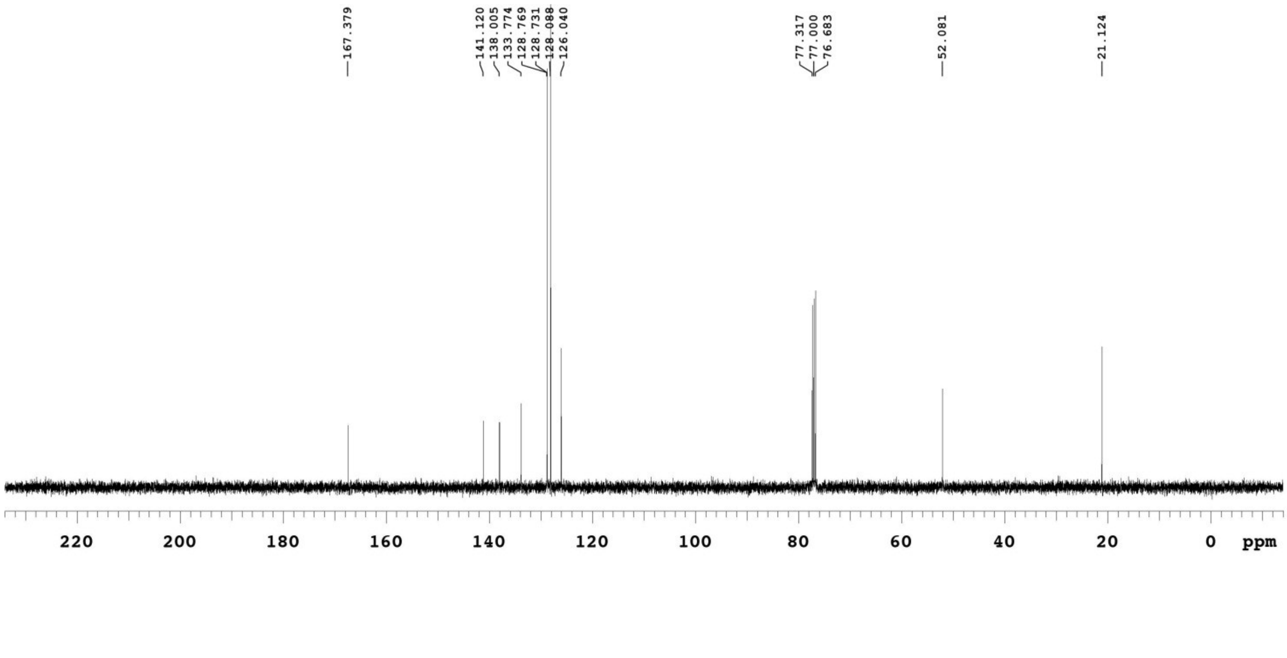

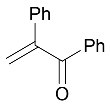
Ph
Ph
O

3n

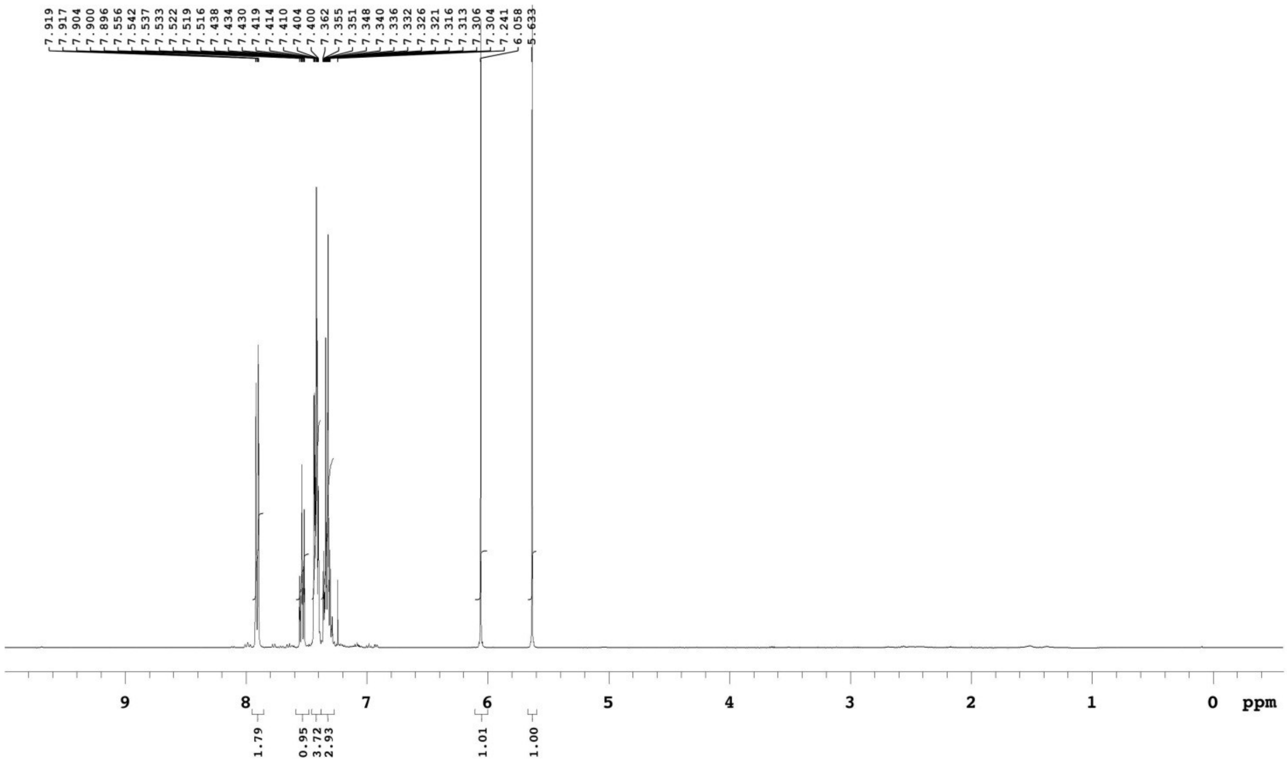
7.919
7.917
7.904
7.900
7.896
7.556
7.542
7.537
7.533
7.522
7.519
7.516
7.438
7.434
7.430
7.419
7.414
7.410
7.404
7.400
7.362
7.355
7.351
7.348
7.340
7.336
7.332
7.326
7.321
7.316
7.313
7.306
7.304
7.241
6.058
5.633
9
8
7
6
5
4
3
2
1
0
ppm
1.79
0.95
3.72
2.93
1.01
1.00

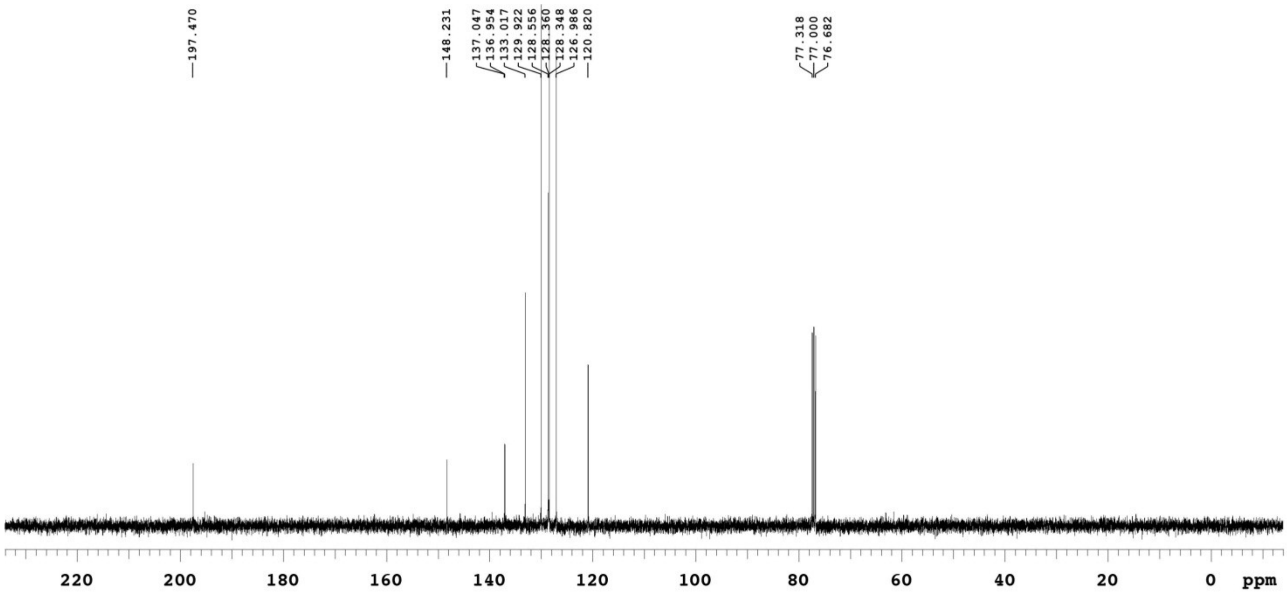
197.470
148.231
137.047
136.954
133.017
129.922
128.556
128.360
128.348
126.986
120.820
77.318
77.000
76.682
220
200
180
160
140
120
100
80
60
40
20
0
ppm

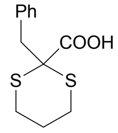

4b

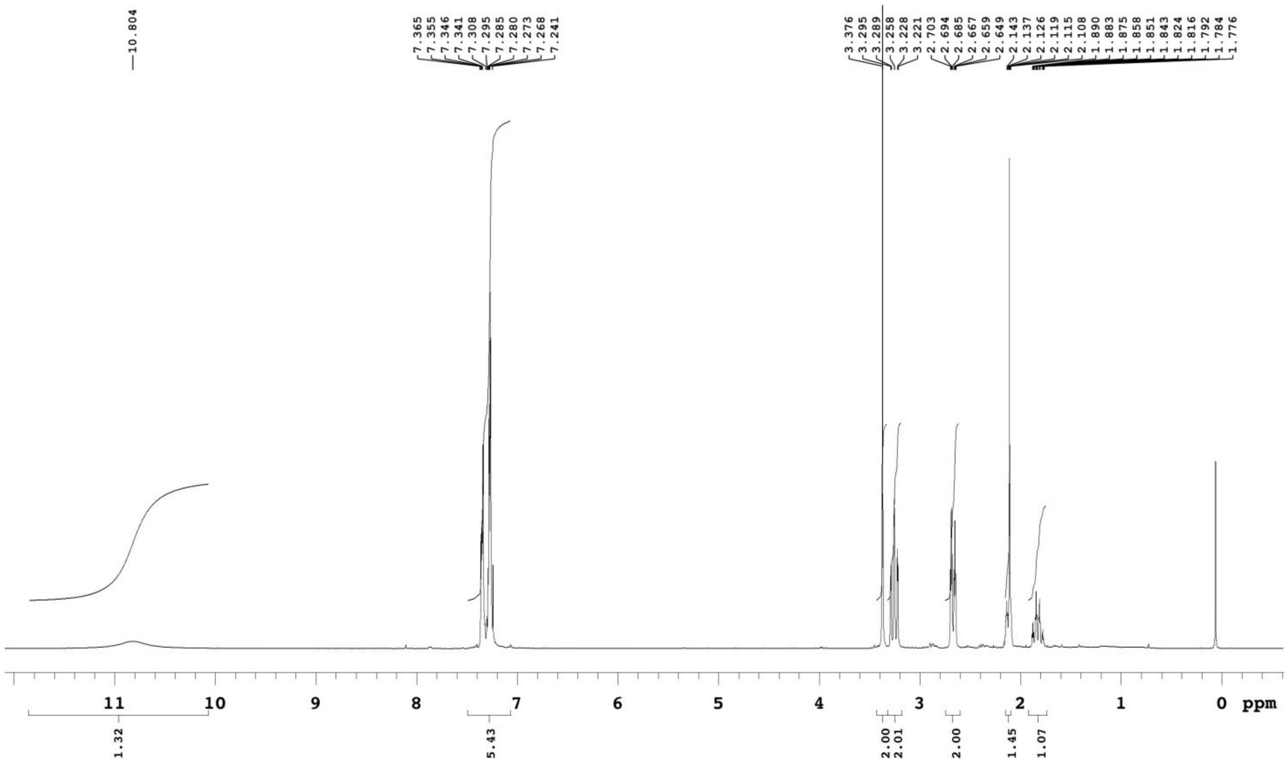

10.804
7.365
7.355
7.346
7.341
7.308
7.295
7.285
7.280
7.273
7.268
7.241
3.376
3.295
3.289
3.258
3.228
3.221
2.703
2.694
2.685
2.667
2.659
2.649
2.143
2.137
2.126
2.119
2.115
2.108
1.890
1.883
1.875
1.858
1.851
1.843
1.824
1.816
1.792
1.784
1.776
11
10
9
8
7
6
5
4
3
2
1
0 ppm
1.32
5.43
2.00
2.01
2.00
1.45
1.07

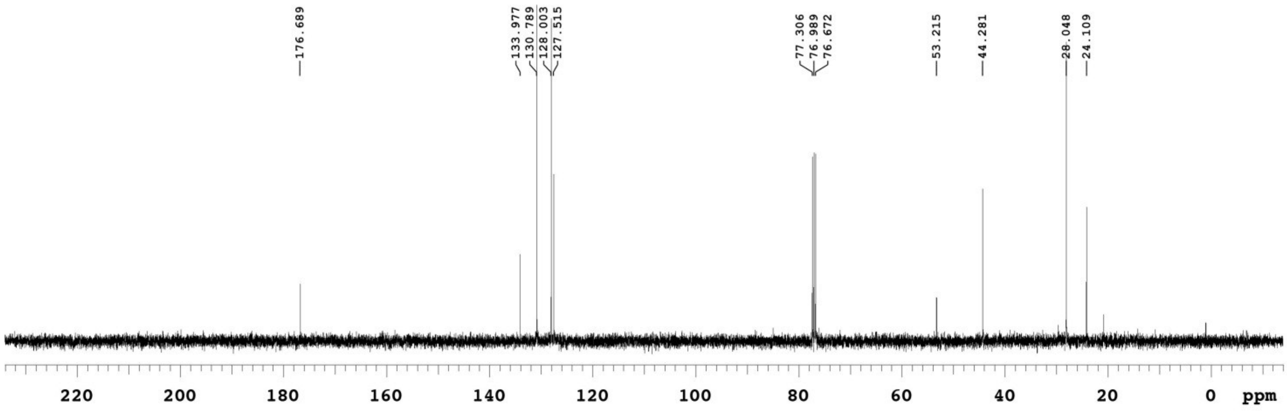

176.689
133.977
130.789
128.003
127.515
77.306
76.989
76.672
53.215
44.281
28.048
24.109
220
200
180
160
140
120
100
80
60
40
20
0 ppm

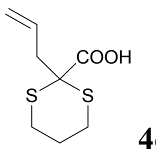
COOH
S
S

4c

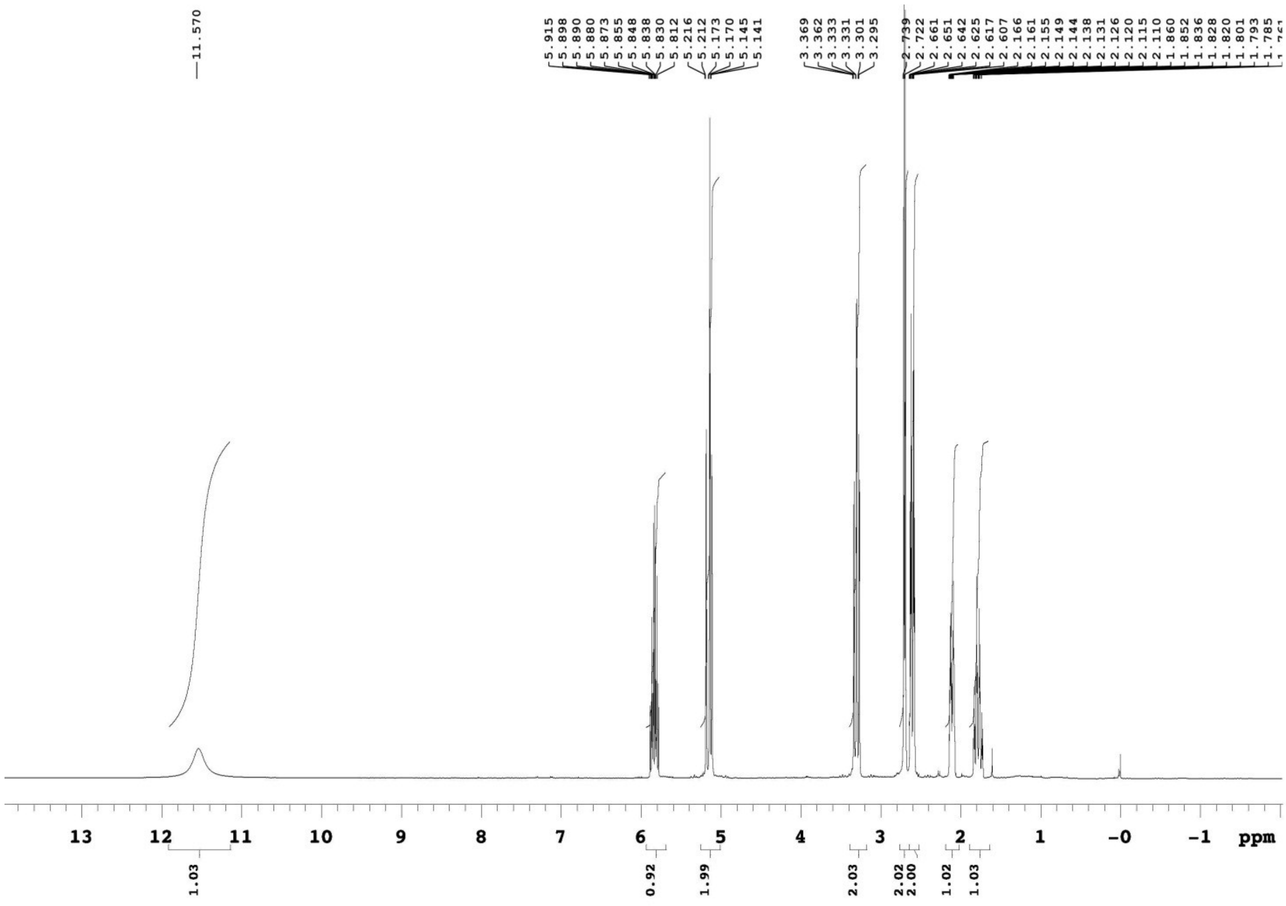
11.570
5.915 5.898 5.890 5.880 5.873 5.855 5.848 5.838 5.830 5.812 5.216 5.212 5.212 5.173 5.170 5.145 5.141
3.369 3.362 3.333 3.331 3.301 3.295
2.739 2.722 2.661 2.651 2.642 2.625 2.617 2.607 2.166 2.161 2.155 2.149 2.144 2.138 2.131 2.126 2.120 2.115 2.110 1.860 1.852 1.836 1.828 1.820 1.801 1.793 1.785
13 12 11 10 9 8 7 6 5 4 3 2 1 -0 -1 ppm
1.03
0.92
1.99
2.03
2.02
2.00
1.02
1.03

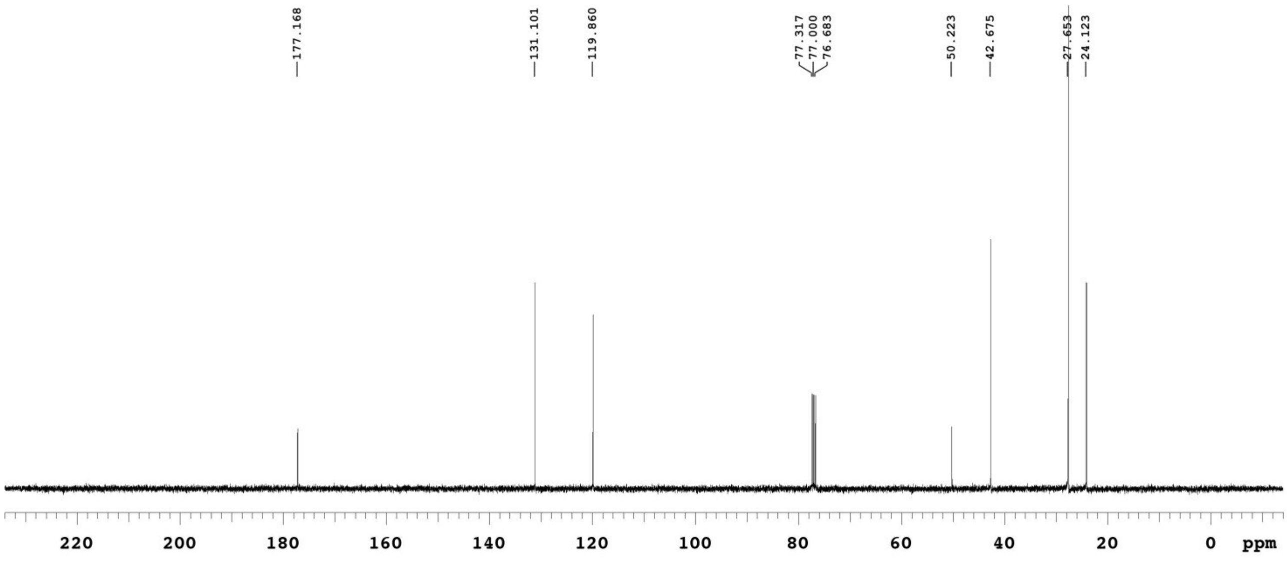
177.168
131.101
119.860
77.317
77.000
76.683
50.223
42.675
27.653
24.123
220 200 180 160 140 120 100 80 60 40 20 0 ppm

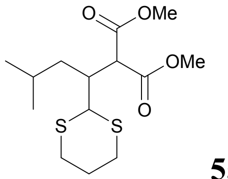
O
OMe
OMe
O
S
S

5a

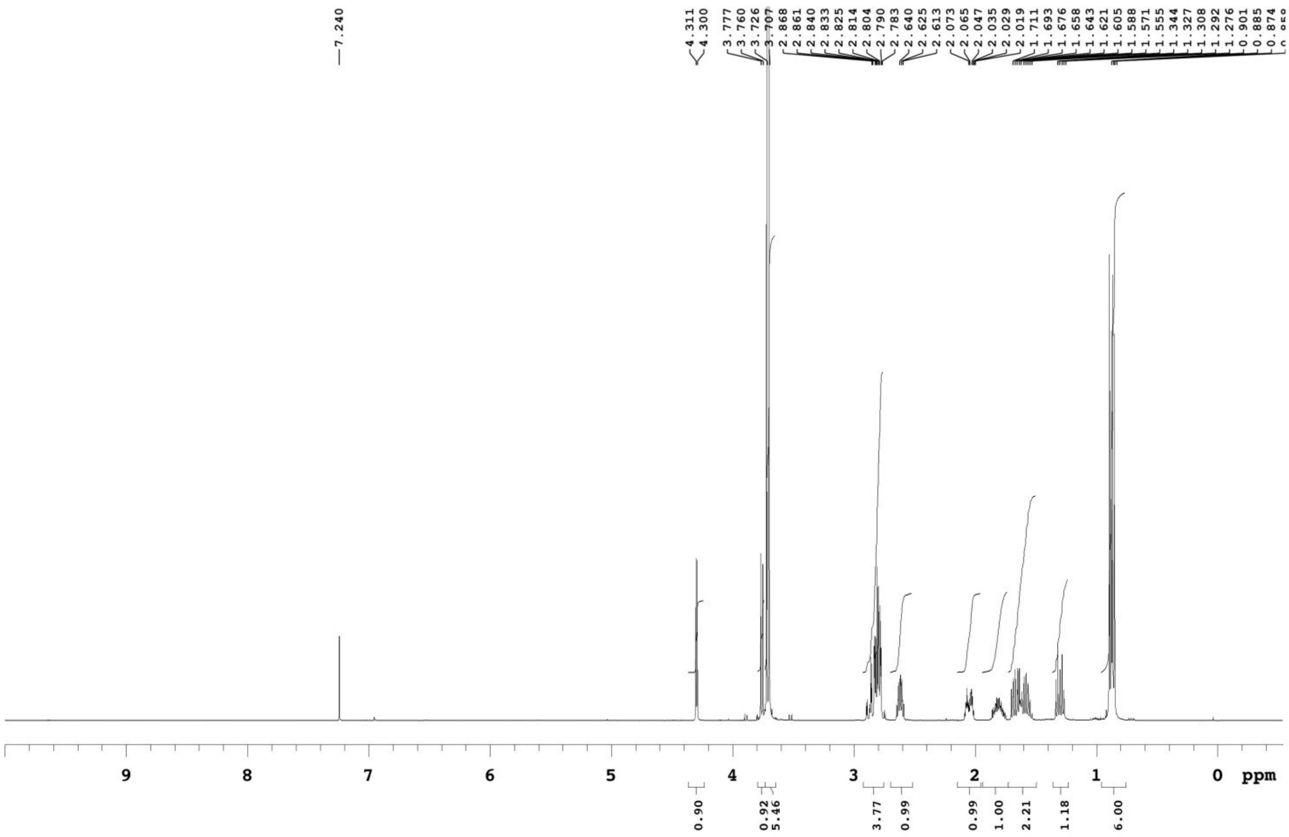
7.240
4.311
4.300
3.777
3.760
3.726
3.707
2.868
2.861
2.840
2.833
2.825
2.814
2.804
2.790
2.783
2.640
2.625
2.613
2.073
2.065
2.047
2.035
2.029
2.019
1.711
1.693
1.676
1.658
1.643
1.621
1.605
1.588
1.571
1.555
1.344
1.327
1.308
1.292
1.276
0.901
0.885
0.874
9
8
7
6
5
4
3
2
1
0
ppm
0.90
0.92
5.46
3.77
0.99
0.99
1.00
2.21
1.18
6.00

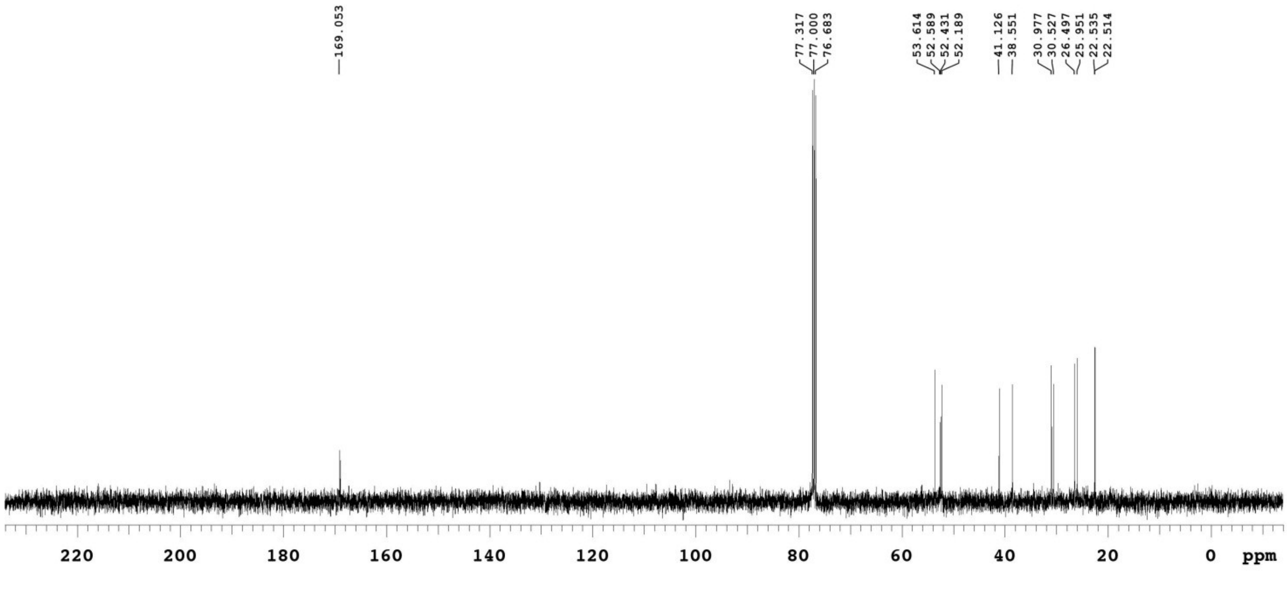
169.053
77.317
77.000
76.683
53.614
52.589
52.431
52.189
41.126
38.551
30.977
30.527
26.497
25.951
22.535
22.514
220
200
180
160
140
120
100
80
60
40
20
0
ppm

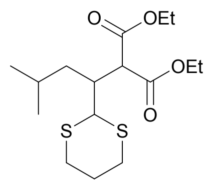
O
OEt
OEt
O
S
S

5b

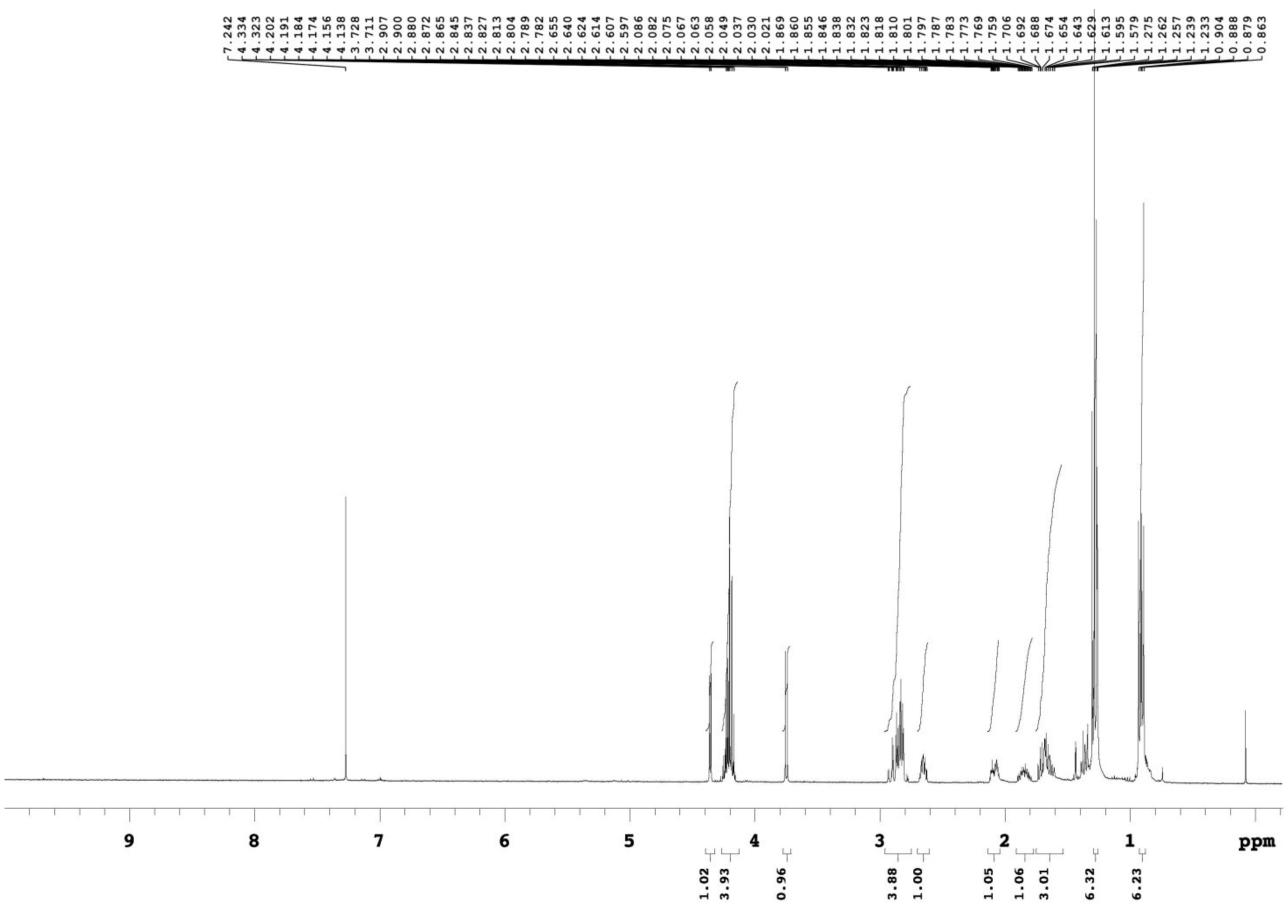
7.242
4.334
4.323
4.202
4.191
4.184
4.174
4.156
4.138
3.728
3.711
2.907
2.900
2.880
2.872
2.865
2.845
2.837
2.827
2.813
2.804
2.789
2.782
2.655
2.640
2.624
2.614
2.607
2.597
2.086
2.082
2.075
2.067
2.063
2.058
2.049
2.037
2.030
2.021
1.869
1.860
1.855
1.846
1.838
1.832
1.823
1.818
1.810
1.801
1.797
1.787
1.783
1.773
1.769
1.759
1.706
1.692
1.688
1.674
1.654
1.643
1.629
1.613
1.595
1.579
1.275
1.262
1.257
1.239
1.233
0.904
0.888
0.879
0.863
9
8
7
6
5
4
3
2
1
ppm
1.02
3.93
0.96
3.88
1.00
1.05
1.06
3.01
6.32
6.23

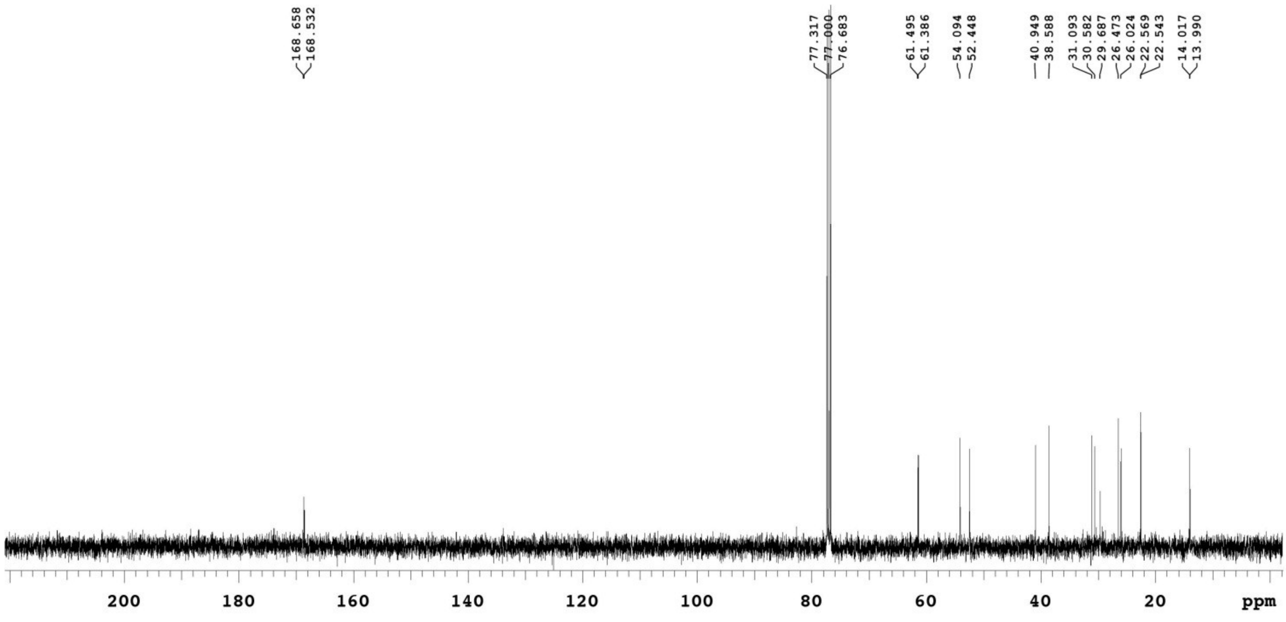
168.658
168.532
77.317
77.000
76.683
61.495
61.386
54.094
52.448
40.949
38.588
31.093
30.582
29.687
26.473
26.024
22.569
22.543
14.017
13.990
200
180
160
140
120
100
80
60
40
20
ppm

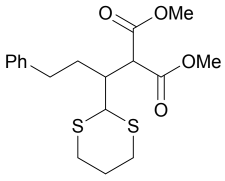

5c

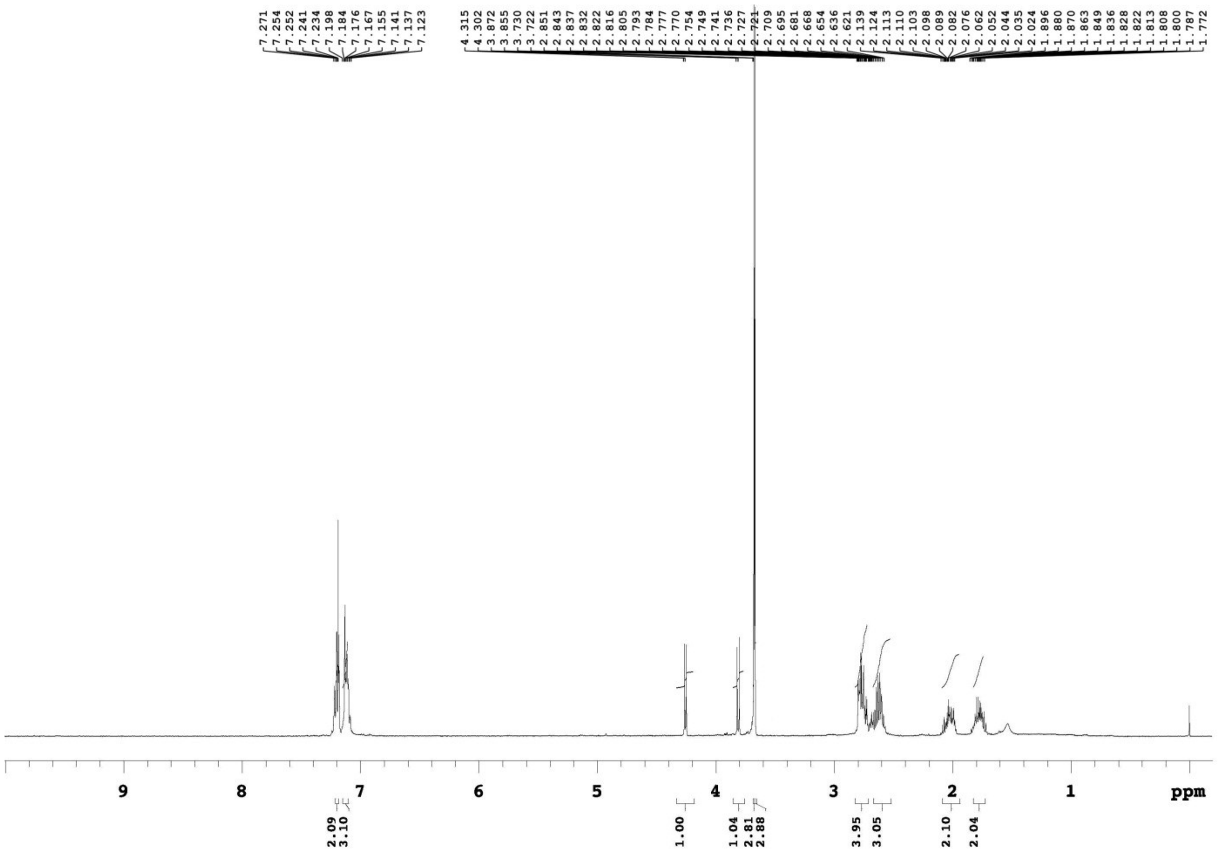

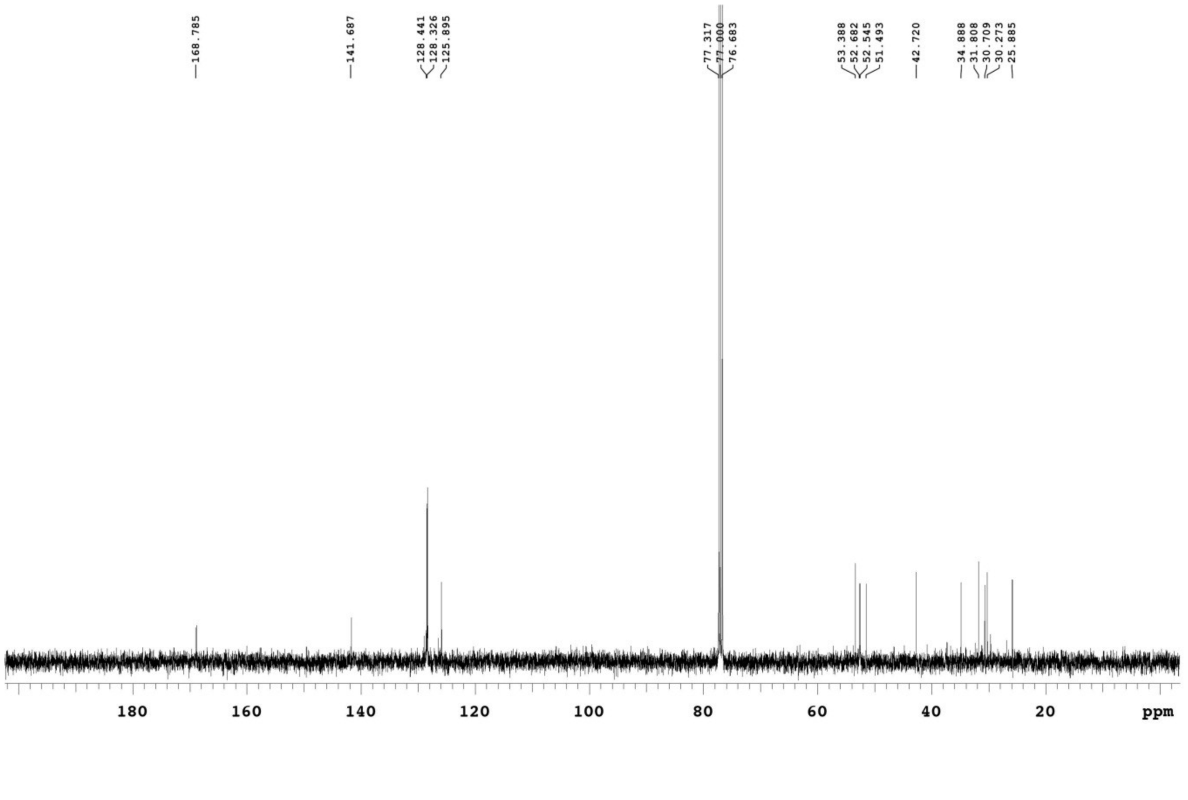

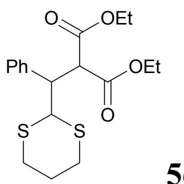
O
OEt
Ph
OEt
O
S
S

5d

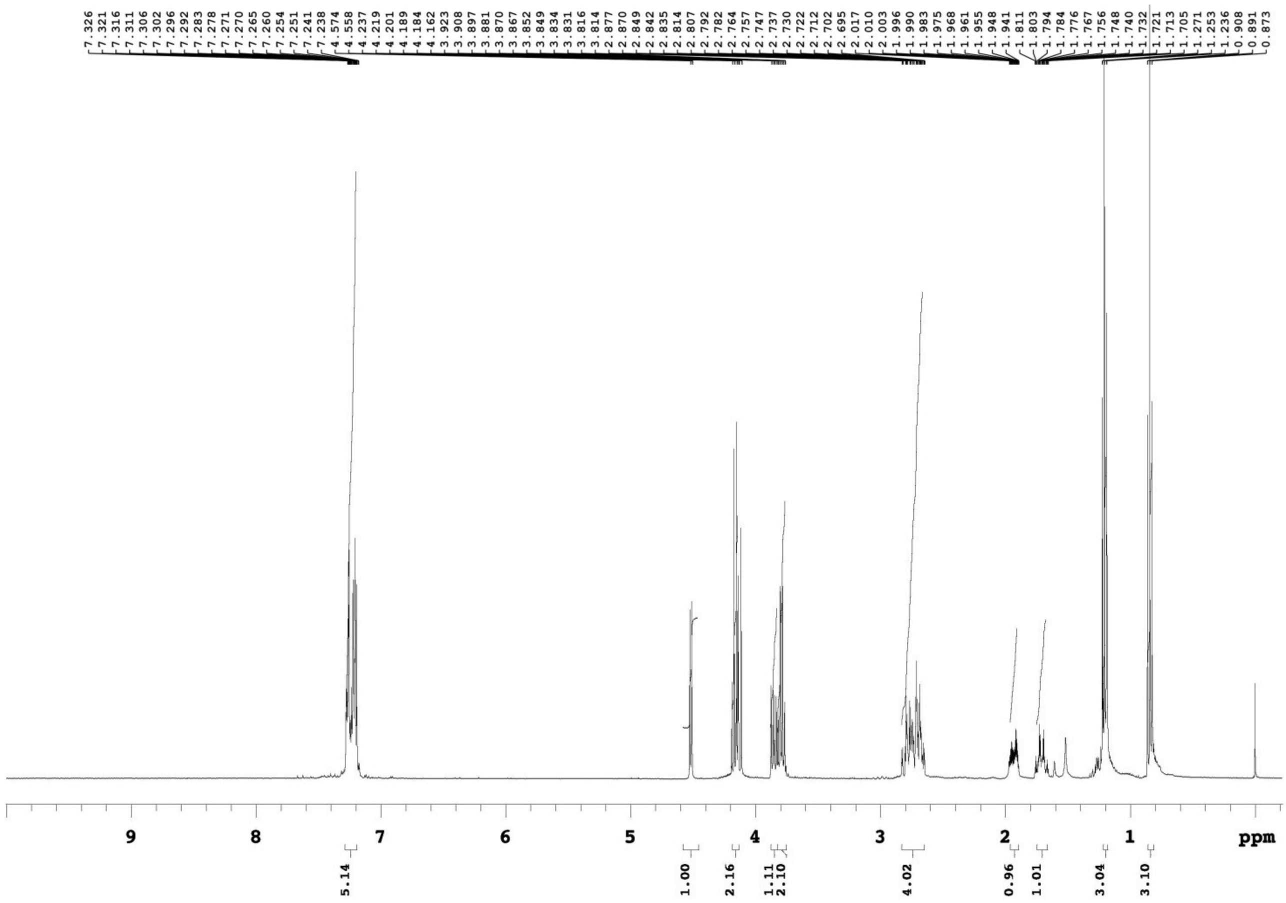
7.326
7.321
7.316
7.311
7.306
7.302
7.296
7.292
7.283
7.278
7.271
7.270
7.265
7.260
7.254
7.251
7.241
7.238
4.574
4.558
4.237
4.219
4.201
4.189
4.184
4.162
3.923
3.908
3.897
3.881
3.870
3.867
3.852
3.849
3.834
3.831
3.816
3.814
2.877
2.870
2.849
2.842
2.835
2.814
2.807
2.792
2.782
2.764
2.757
2.747
2.737
2.730
2.722
2.712
2.702
2.695
2.017
2.010
2.003
1.996
1.990
1.983
1.975
1.968
1.961
1.955
1.948
1.941
1.811
1.803
1.794
1.784
1.776
1.767
1.756
1.748
1.740
1.732
1.721
1.713
1.705
1.271
1.253
1.236
0.908
0.891
0.873
9
8
7
6
5
4
3
2
1
ppm
5.14
1.00
2.16
1.11
2.10
4.02
0.96
1.01
3.04
3.10

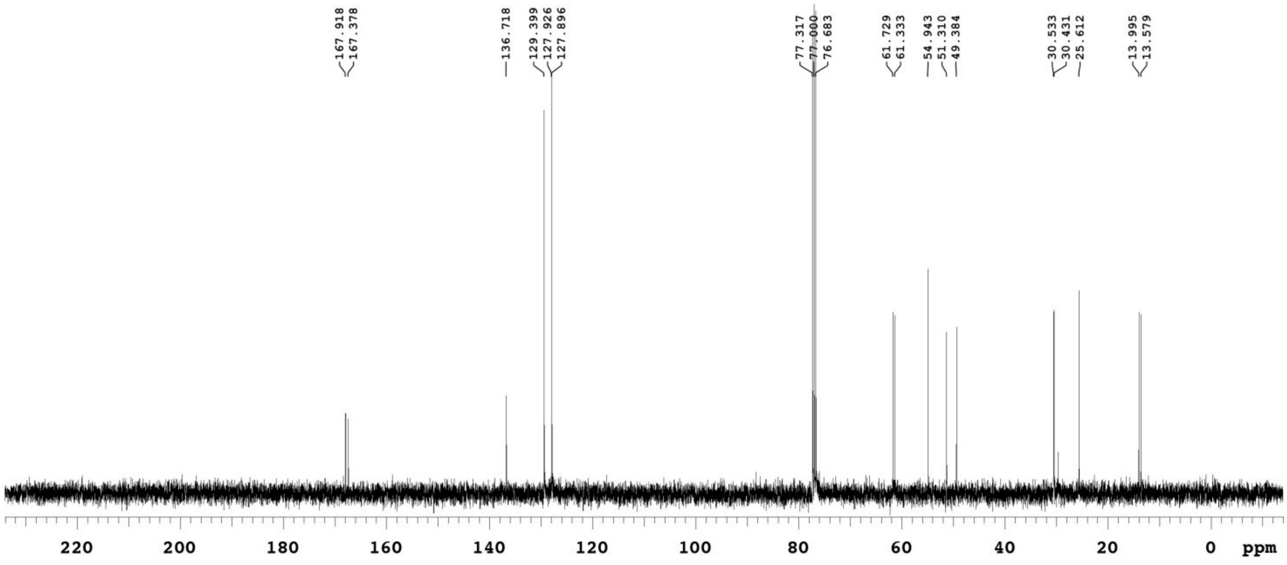
167.918
167.378
136.718
129.399
127.926
127.896
77.317
77.000
76.683
61.729
61.333
54.943
51.310
49.384
30.533
30.431
25.612
13.995
13.579
220
200
180
160
140
120
100
80
60
40
20
0
ppm

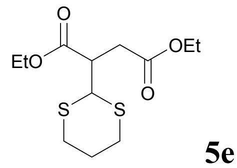

O
EtO
OEt
O
S
S

5e

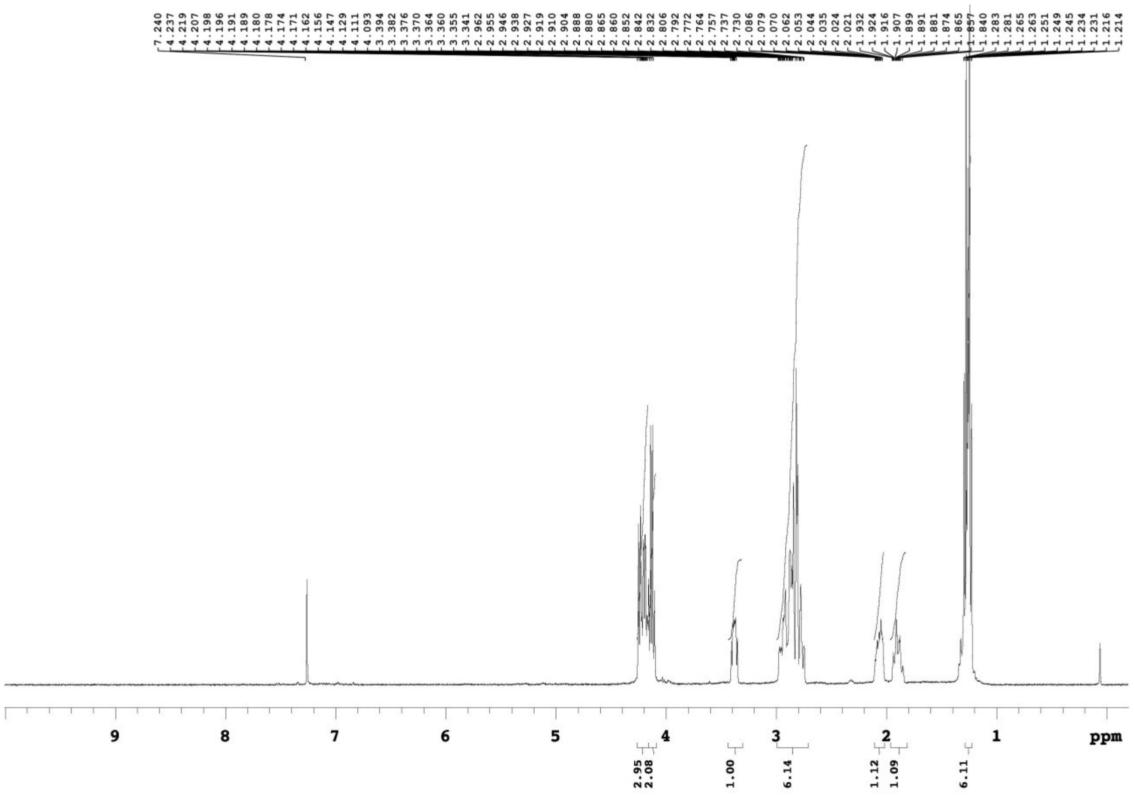

7.240 4.237 4.219 4.207 4.198 4.196 4.191 4.189 4.180 4.178 4.174 4.171 4.162 4.156 4.147 4.129 4.111 4.093 3.394 3.382 3.376 3.370 3.364 3.360 3.355 3.341 2.962 2.955 2.946 2.938 2.927 2.919 2.910 2.904 2.888 2.880 2.865 2.860 2.852 2.842 2.832 2.806 2.792 2.772 2.764 2.757 2.737 2.730 2.086 2.079 2.070 2.062 2.053 2.044 2.035 2.024 2.021 1.932 1.924 1.916 1.907 1.899 1.891 1.881 1.874 1.865 1.857 1.840 1.283 1.281 1.265 1.263 1.251 1.249 1.245 1.234 1.231 1.216 1.214
9 8 7 6 5 4 3 2 1 ppm
2.95 2.08 1.00 6.14 1.12 1.09 6.11

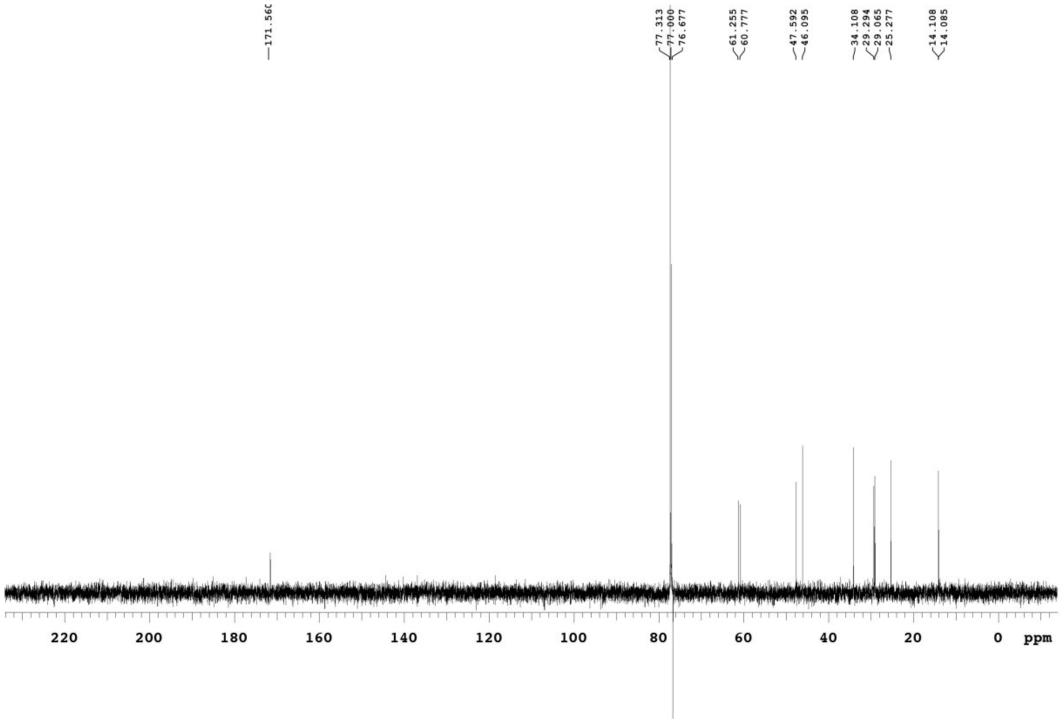

171.560
77.313 77.000 76.677
61.255 60.777
47.592 46.095
34.108 29.294 29.065 25.277
14.108 14.085
220 200 180 160 140 120 100 80 60 40 20 0 ppm

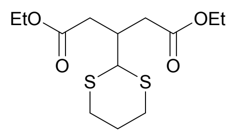
EtO
OEt
O
O
S
S

5f

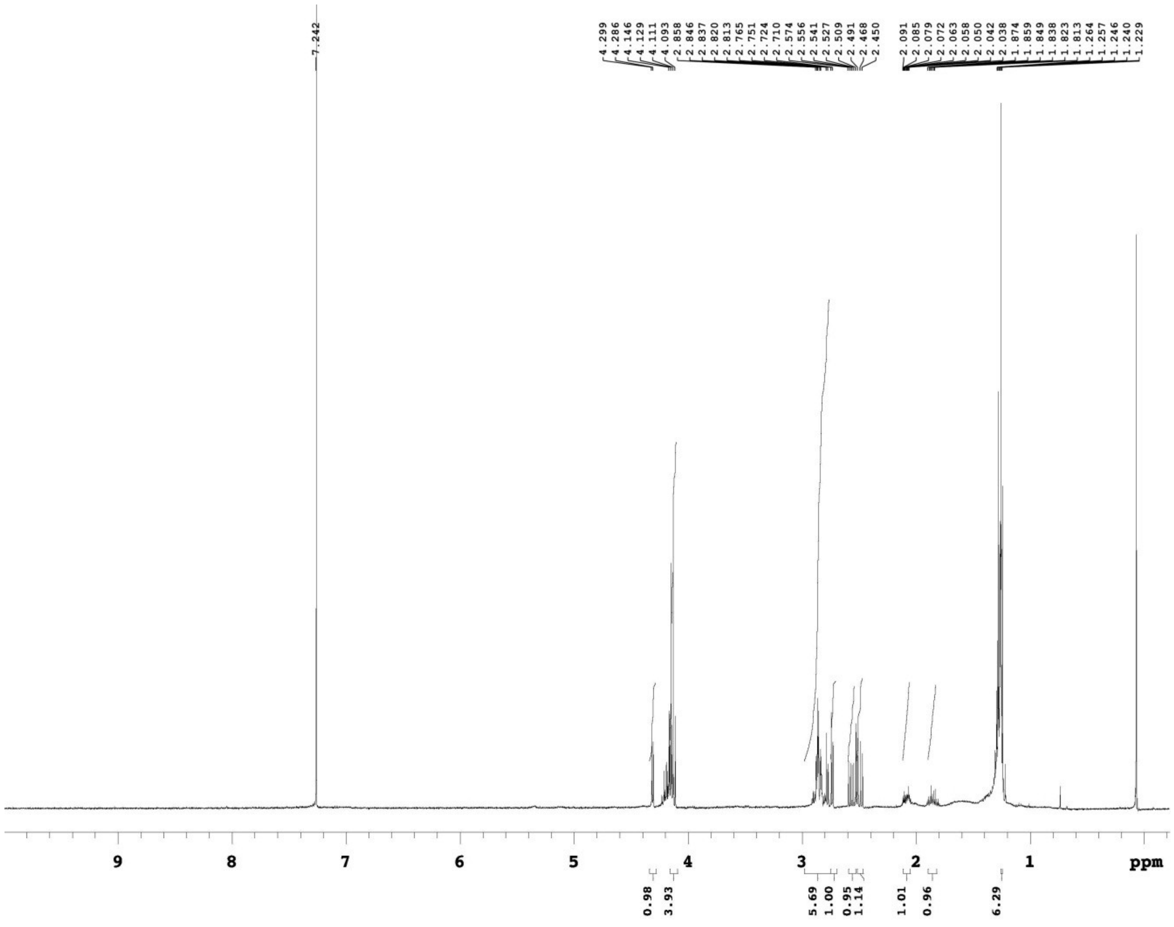
7.242
4.299
4.286
4.146
4.129
4.111
4.093
2.858
2.846
2.837
2.820
2.813
2.765
2.751
2.724
2.710
2.574
2.556
2.541
2.527
2.509
2.491
2.468
2.450
2.091
2.085
2.079
2.072
2.063
2.058
2.050
2.042
2.038
1.874
1.859
1.849
1.838
1.823
1.813
1.264
1.257
1.246
1.240
1.229
9
8
7
6
5
4
3
2
1
ppm
0.98
3.93
5.69
1.00
0.95
1.14
1.01
0.96
6.29

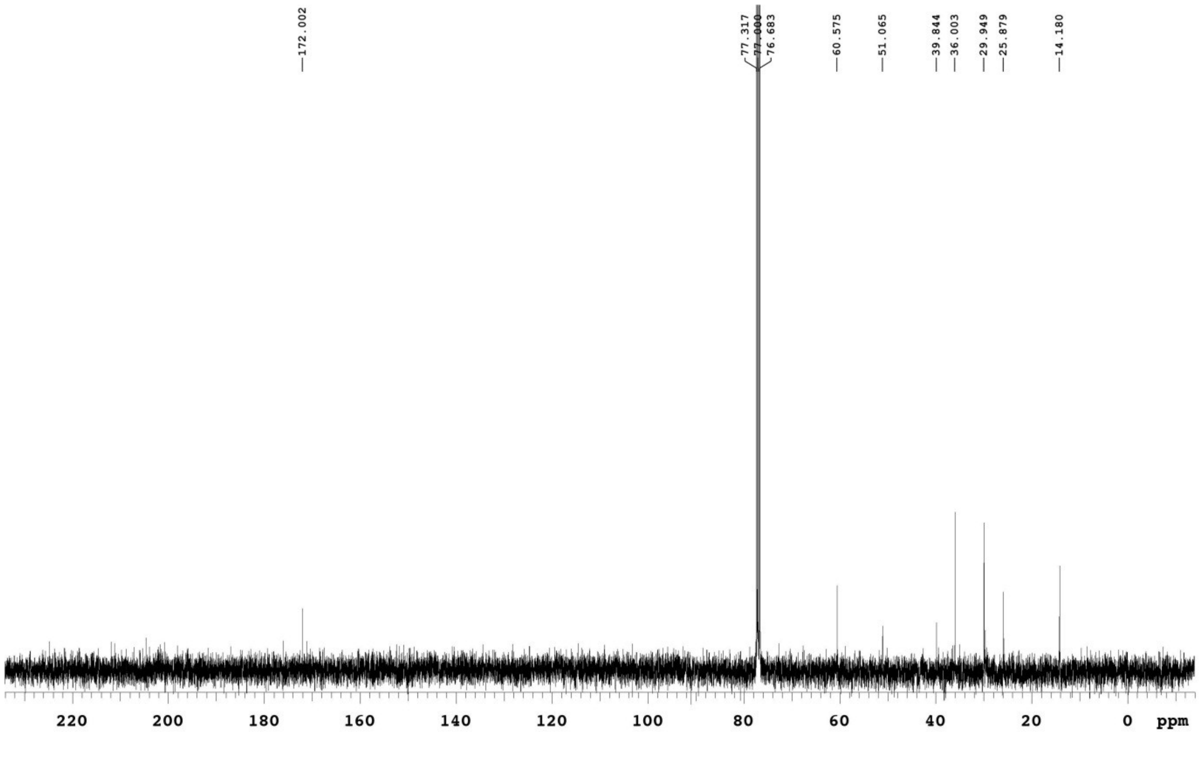
172.002
77.317
77.000
76.683
60.575
51.065
39.844
36.003
29.949
25.879
14.180
220
200
180
160
140
120
100
80
60
40
20
0
ppm

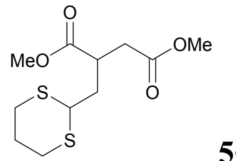

O
MeO
OMe
O
S
S

5g

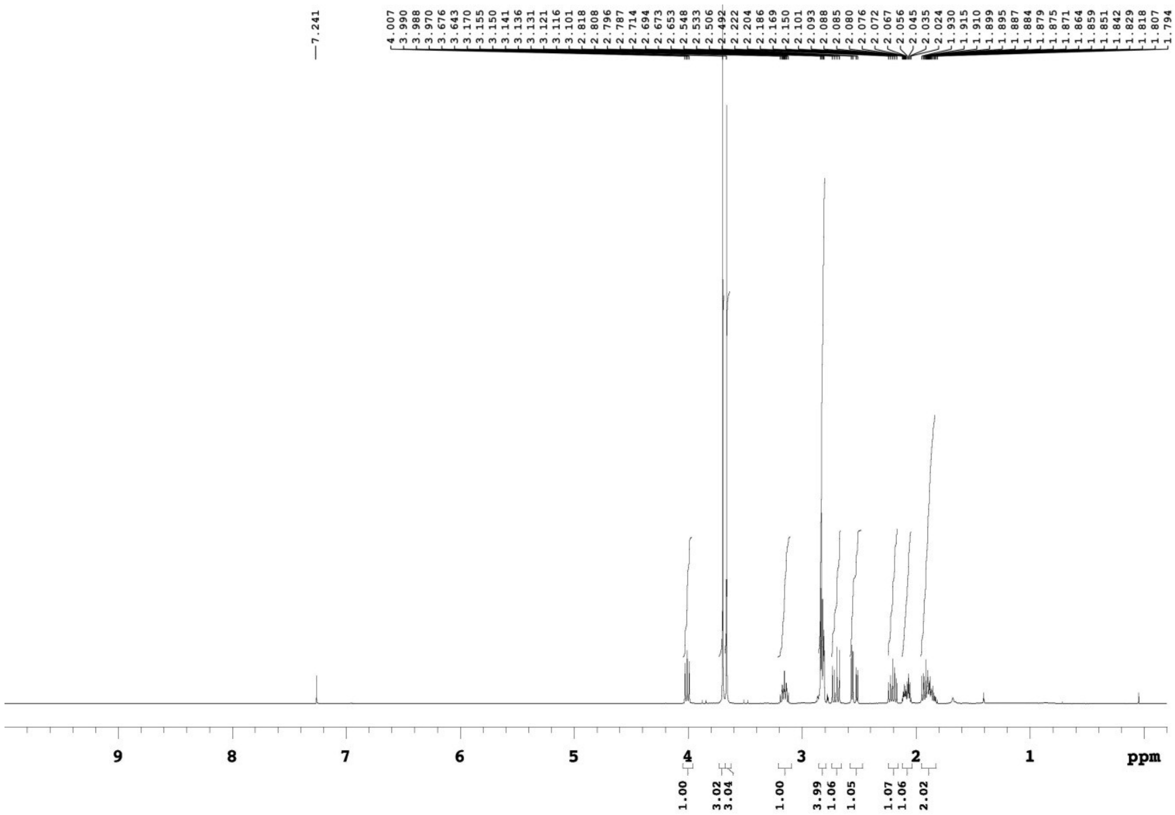

7.241
4.007
3.990
3.988
3.970
3.676
3.643
3.170
3.155
3.150
3.141
3.136
3.131
3.121
3.116
3.101
2.818
2.808
2.796
2.787
2.714
2.694
2.673
2.653
2.548
2.533
2.506
2.492
2.222
2.204
2.186
2.169
2.150
2.101
2.093
2.088
2.085
2.080
2.076
2.072
2.067
2.056
2.045
2.035
2.024
1.930
1.915
1.910
1.899
1.895
1.887
1.884
1.879
1.875
1.871
1.864
1.859
1.851
1.842
1.829
1.818
1.807
1.794
9
8
7
6
5
4
3
2
1
ppm
1.00
3.02
3.04
1.00
3.99
1.06
1.05
1.07
1.06
2.02

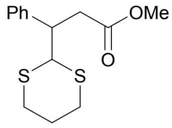

5h

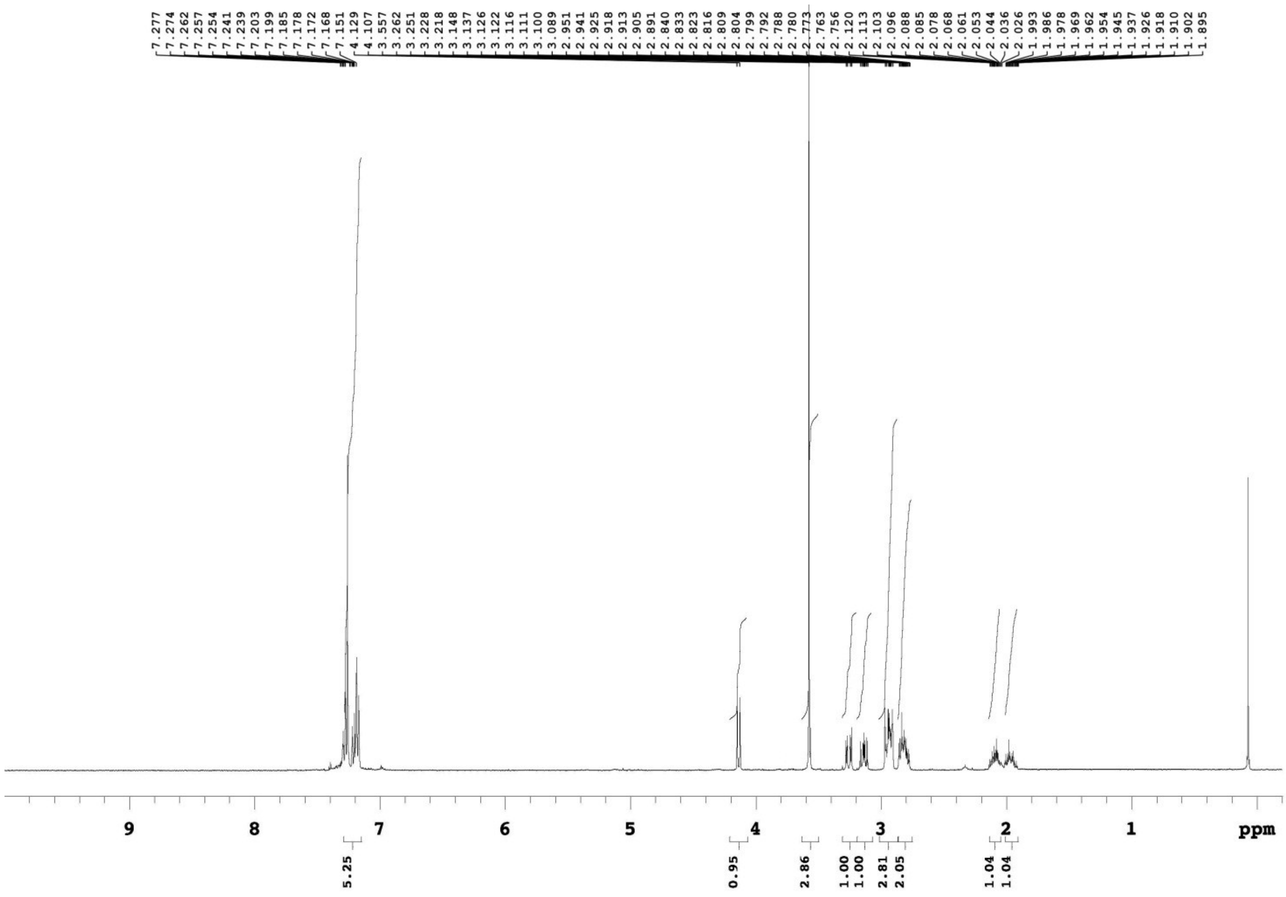

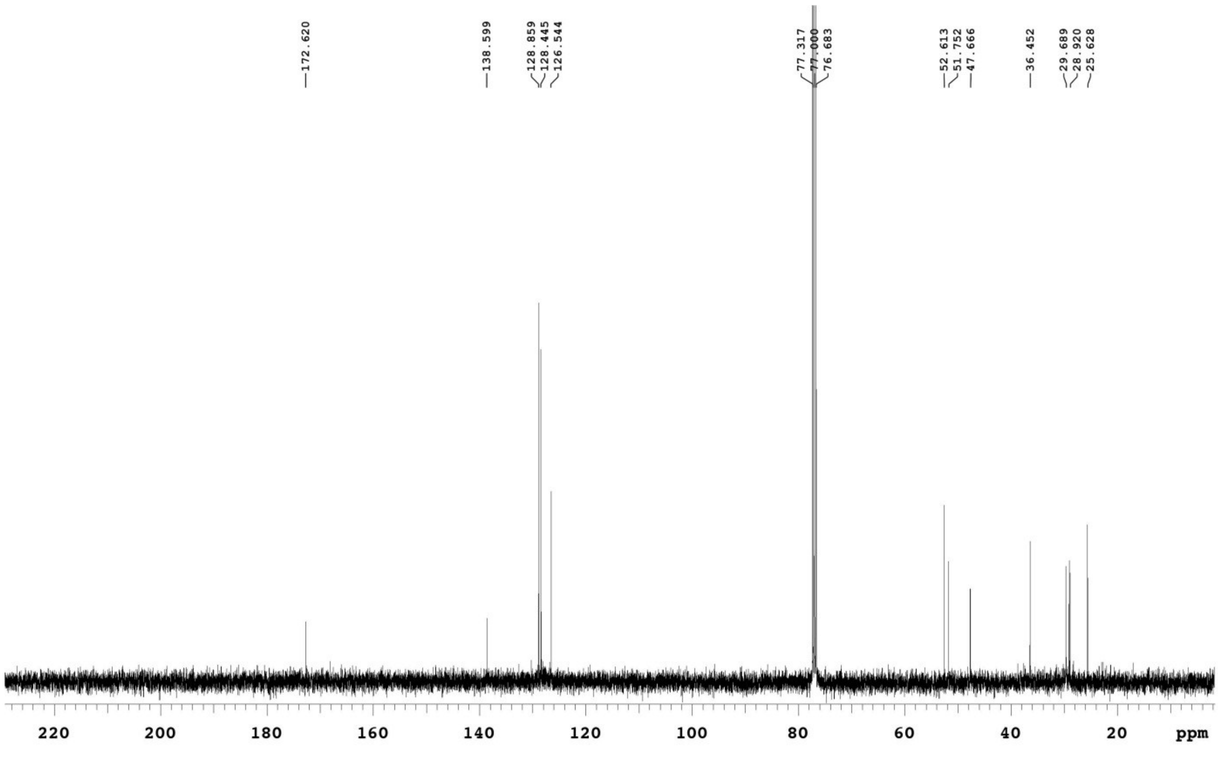

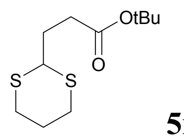
OtBu
O
S
S

5i

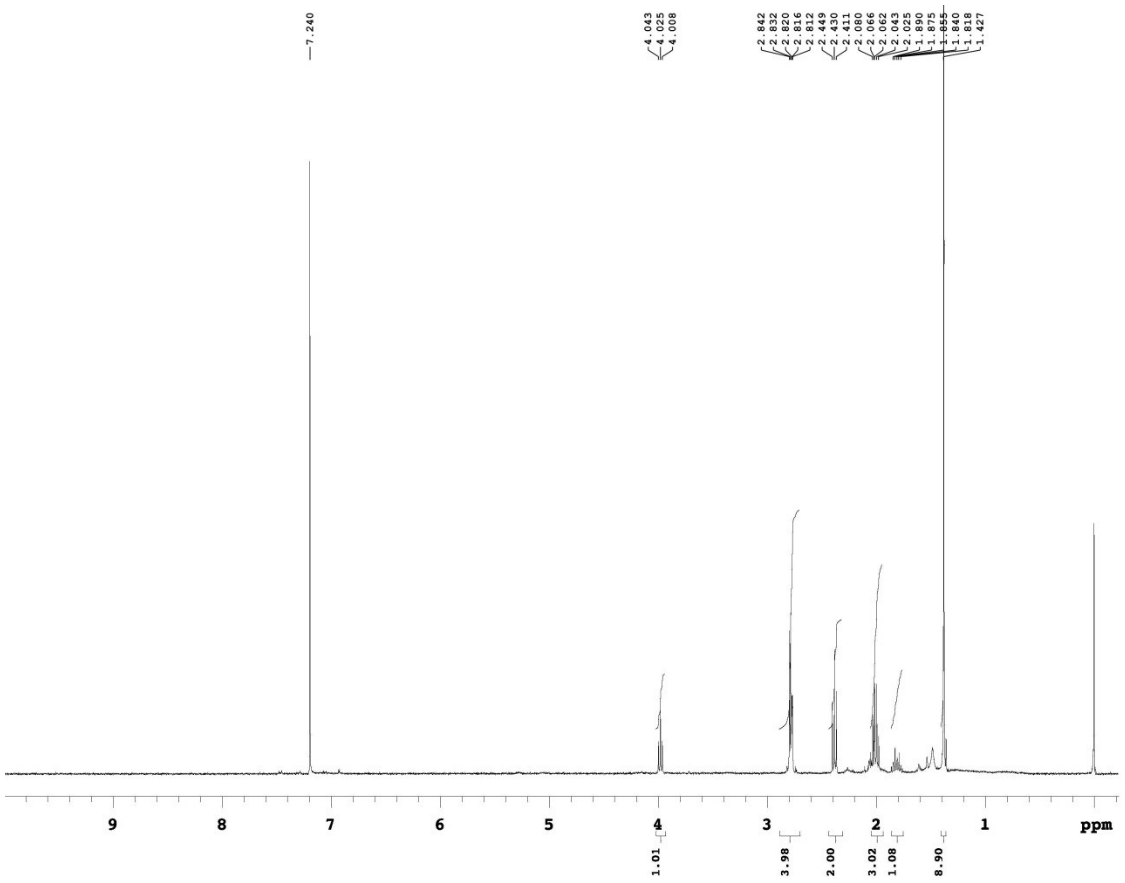
7.240
4.043
4.025
4.008
2.842
2.832
2.820
2.816
2.812
2.449
2.430
2.411
2.080
2.066
2.062
2.043
2.025
1.890
1.875
1.855
1.840
1.818
1.427
9
8
7
6
5
4
3
2
1
ppm
1.01
3.98
2.00
3.02
1.08
8.90

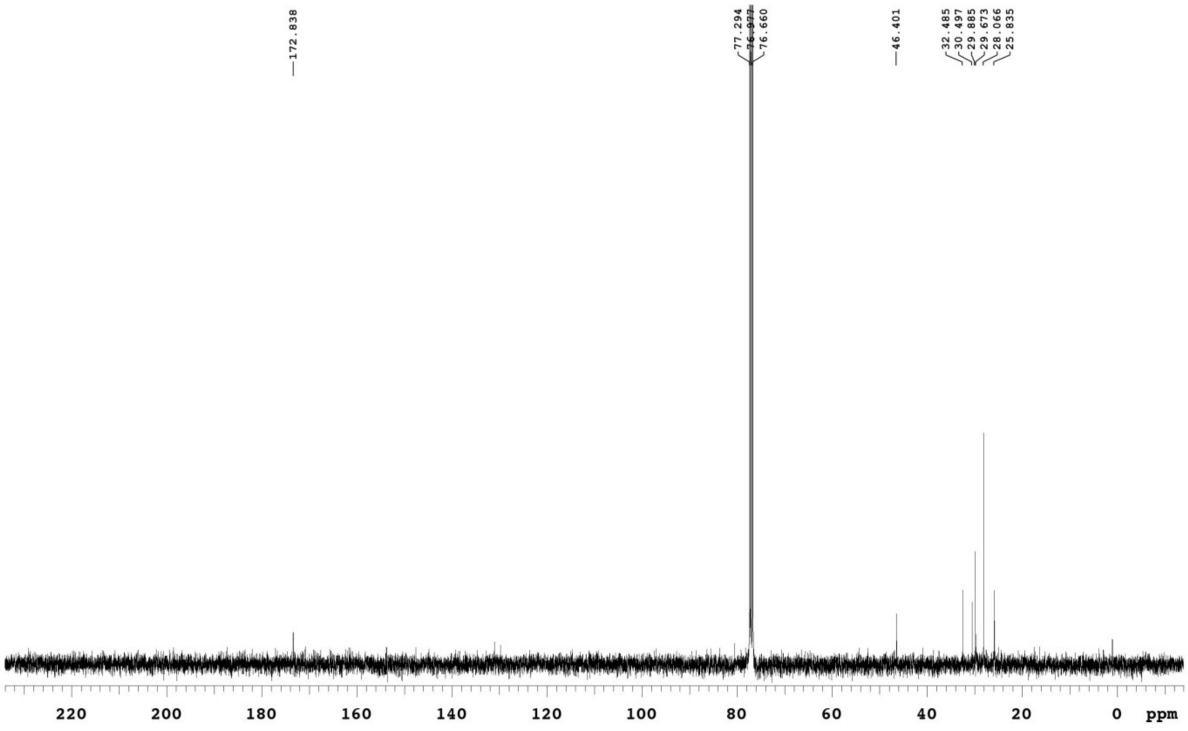
172.838
77.294
76.977
76.660
46.401
32.485
30.497
29.885
29.673
28.066
25.835
220
200
180
160
140
120
100
80
60
40
20
0
ppm

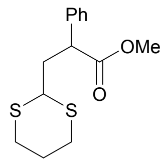
Ph
OMe
O
S
S

5j

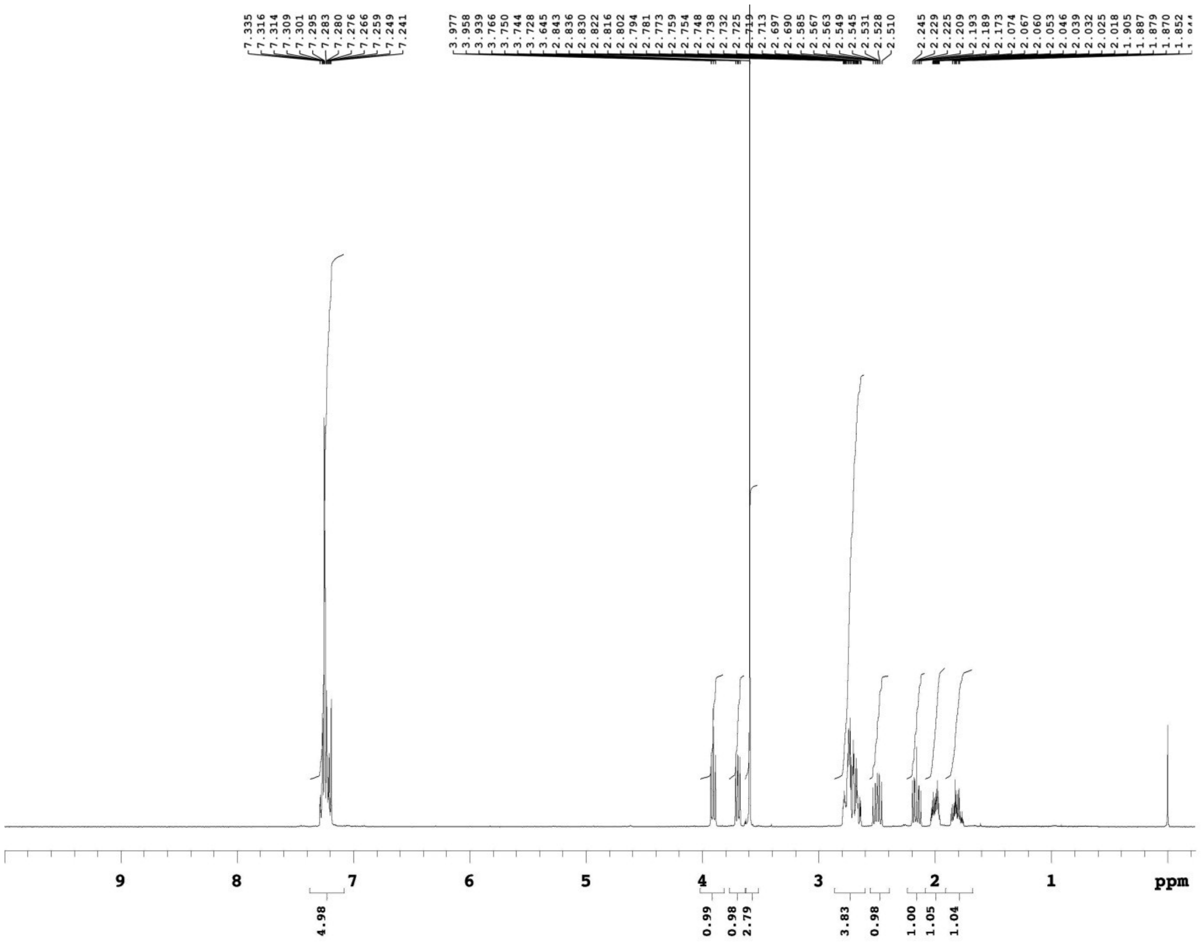
7.335
7.316
7.314
7.309
7.301
7.295
7.283
7.280
7.276
7.266
7.259
7.249
7.241
3.977
3.958
3.939
3.766
3.750
3.744
3.728
3.645
2.843
2.836
2.830
2.822
2.816
2.802
2.794
2.781
2.773
2.759
2.754
2.748
2.738
2.732
2.725
2.719
2.713
2.697
2.690
2.585
2.567
2.563
2.549
2.545
2.531
2.528
2.510
2.245
2.229
2.225
2.209
2.193
2.189
2.173
2.074
2.067
2.060
2.053
2.046
2.039
2.032
2.025
2.018
1.905
1.887
1.879
1.870
1.852
9
8
7
6
5
4
3
2
1
ppm
4.98
0.99
0.98
2.79
3.83
0.98
1.00
1.05
1.04

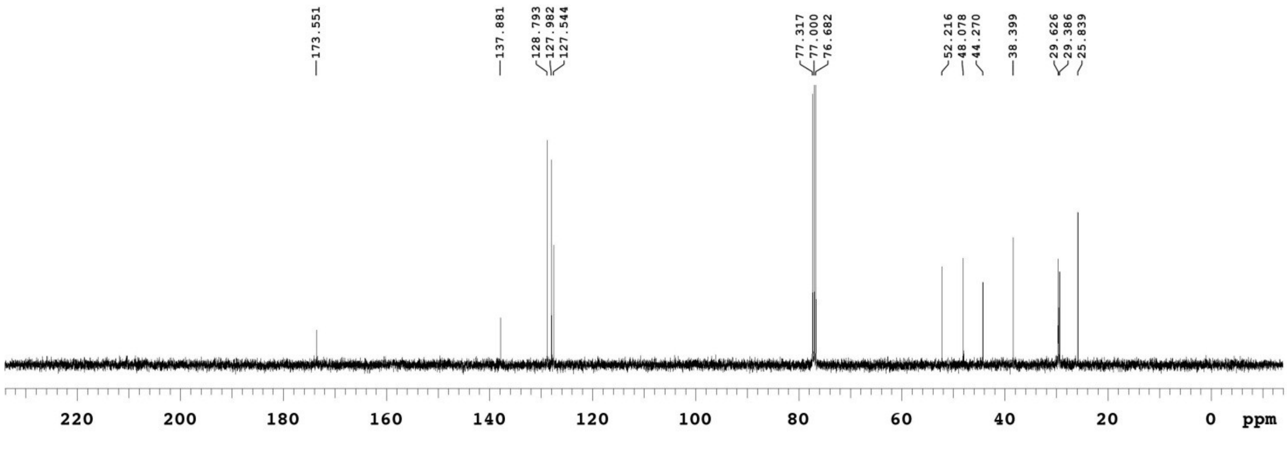
173.551
137.881
128.793
127.982
127.544
77.317
77.000
76.682
52.216
48.078
44.270
38.399
29.626
29.386
25.839
220
200
180
160
140
120
100
80
60
40
20
0
ppm

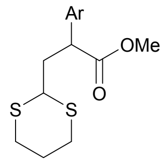

Ar = 4-Me-C_6H_4 **5k**

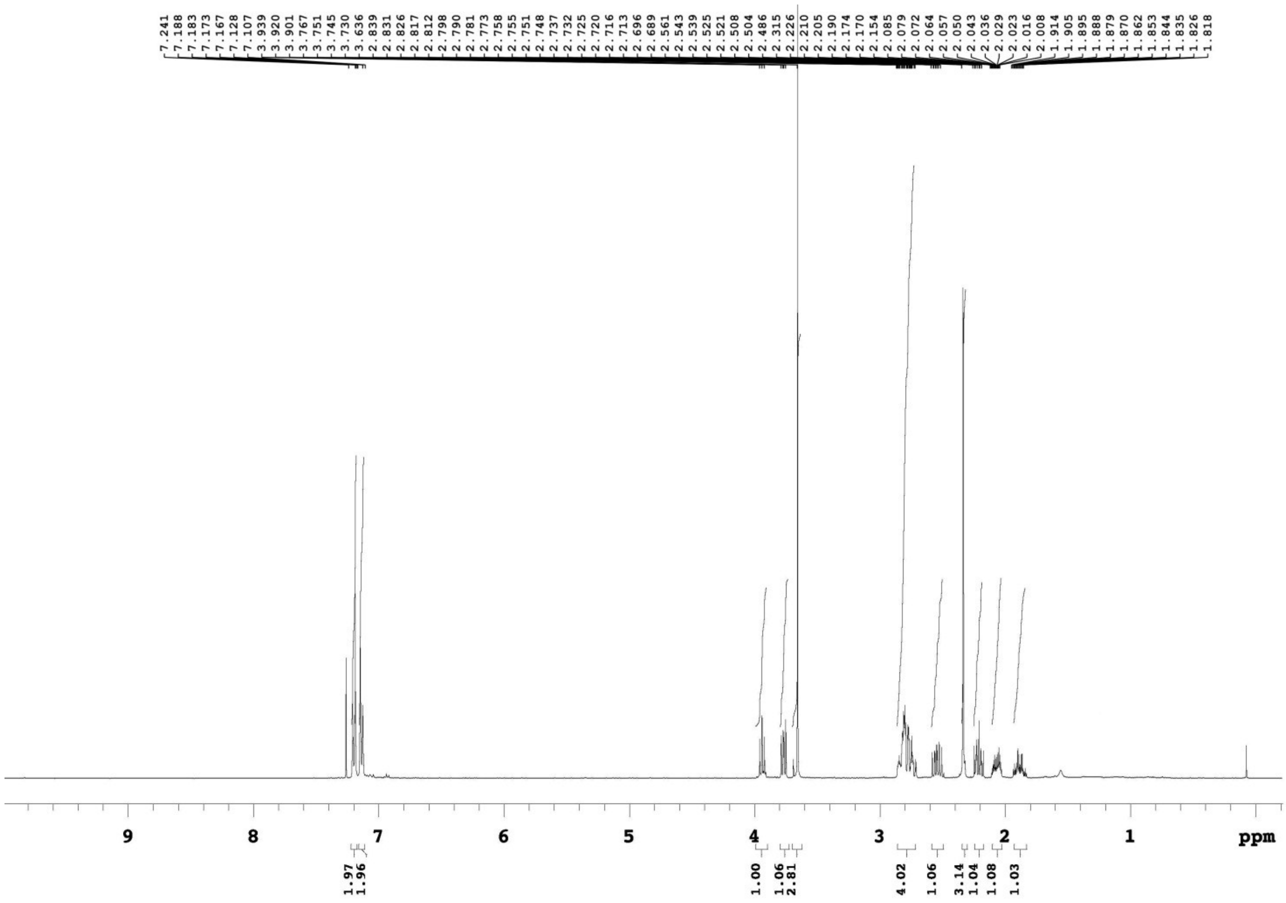

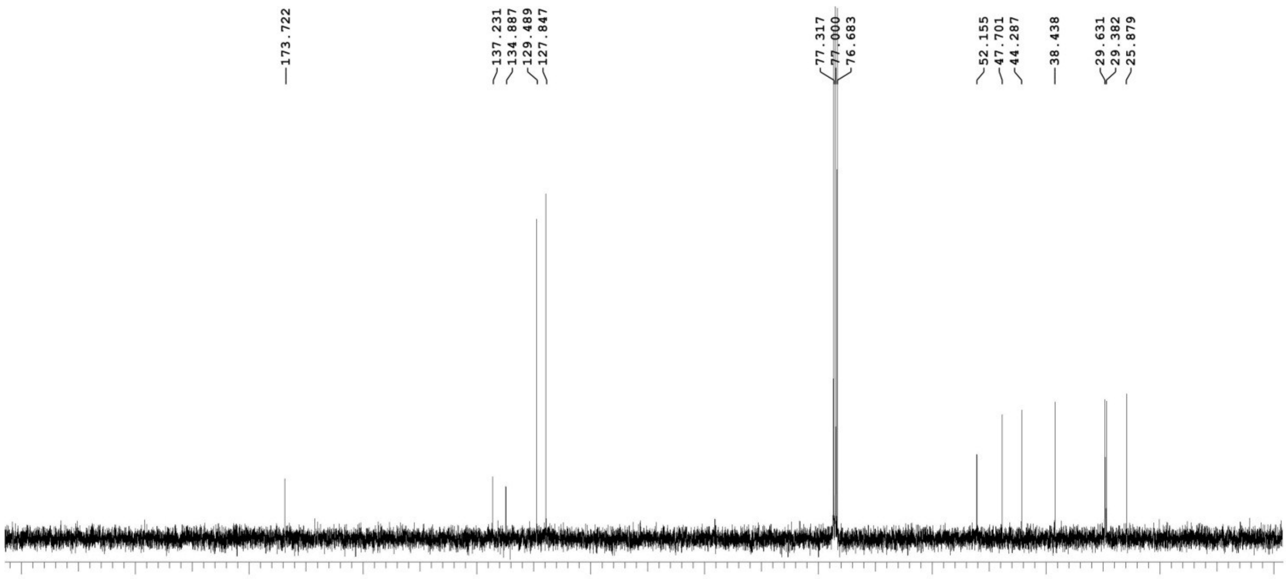

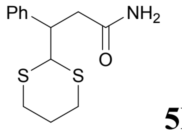
Ph
NH2
O
S
S

5l

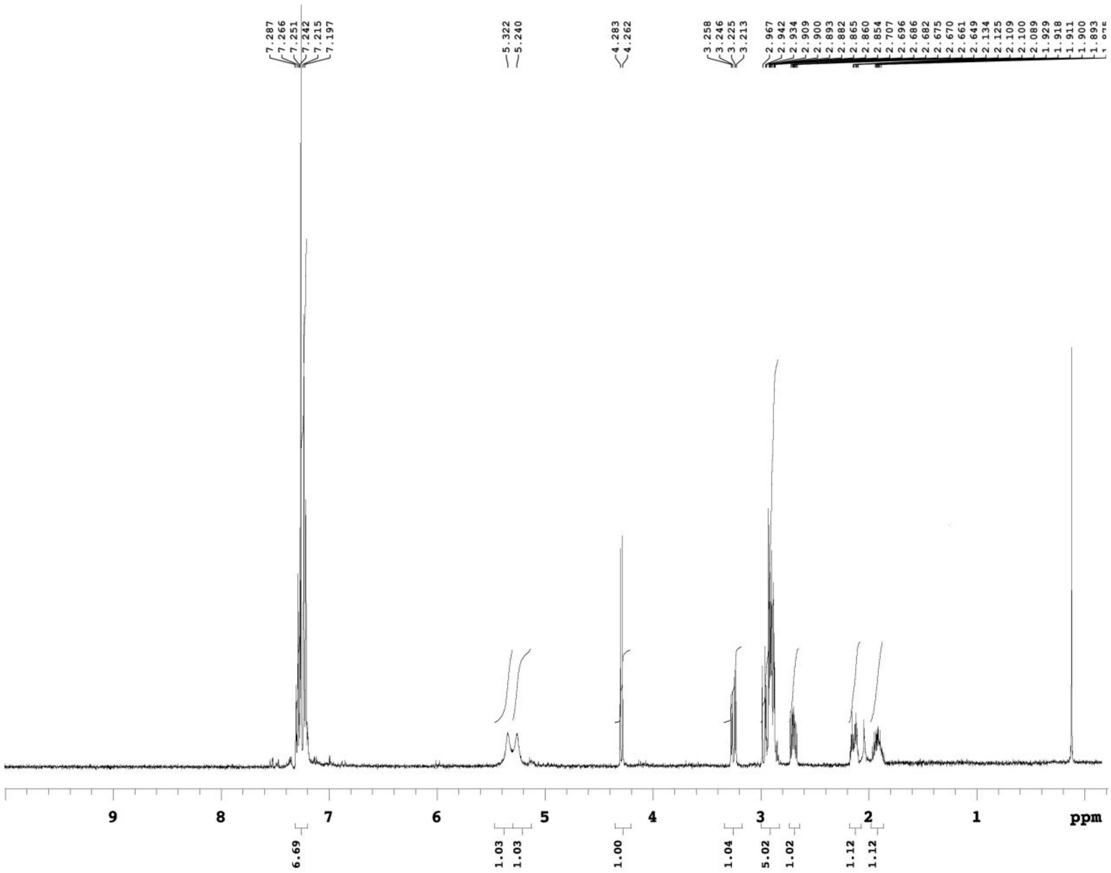
7.287
7.266
7.251
7.242
7.215
7.197
5.322
5.240
4.283
4.262
3.258
3.246
3.225
3.213
2.967
2.942
2.934
2.909
2.900
2.893
2.882
2.865
2.860
2.854
2.707
2.696
2.686
2.682
2.675
2.670
2.661
2.649
2.134
2.125
2.109
2.100
2.089
1.929
1.918
1.911
1.900
1.893
[illegible]
9
8
7
6
5
4
3
2
1
ppm
6.69
1.03
1.03
1.00
1.04
5.02
1.02
1.12
1.12

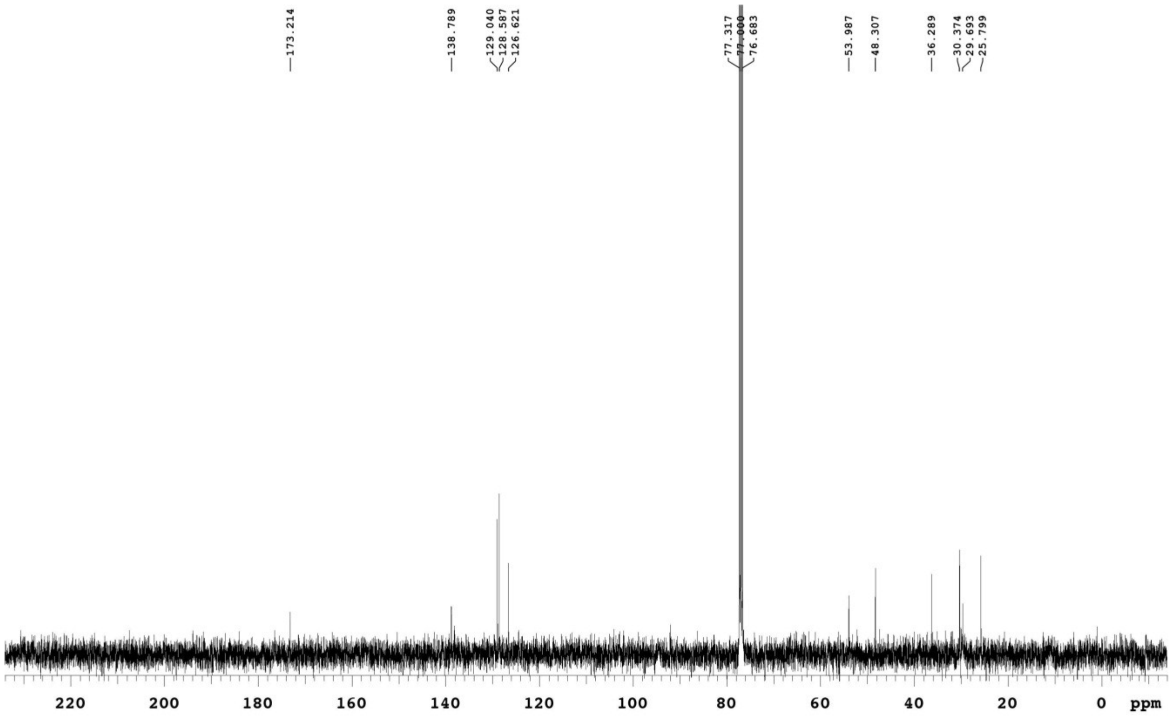
173.214
138.789
129.040
128.587
126.621
77.317
77.000
76.683
53.987
48.307
36.289
30.374
29.693
25.799
220
200
180
160
140
120
100
80
60
40
20
0
ppm

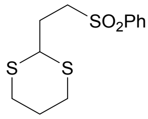

5m

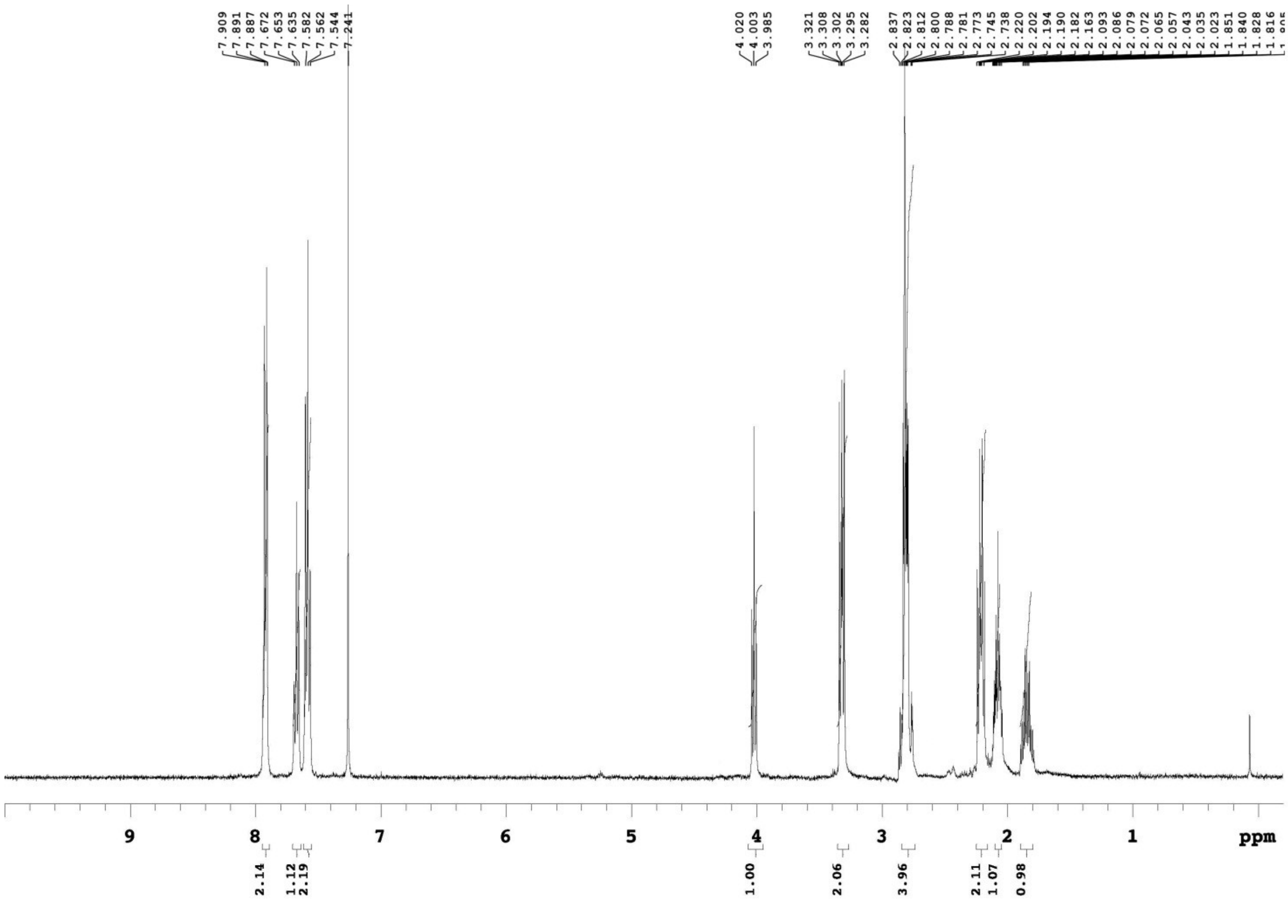

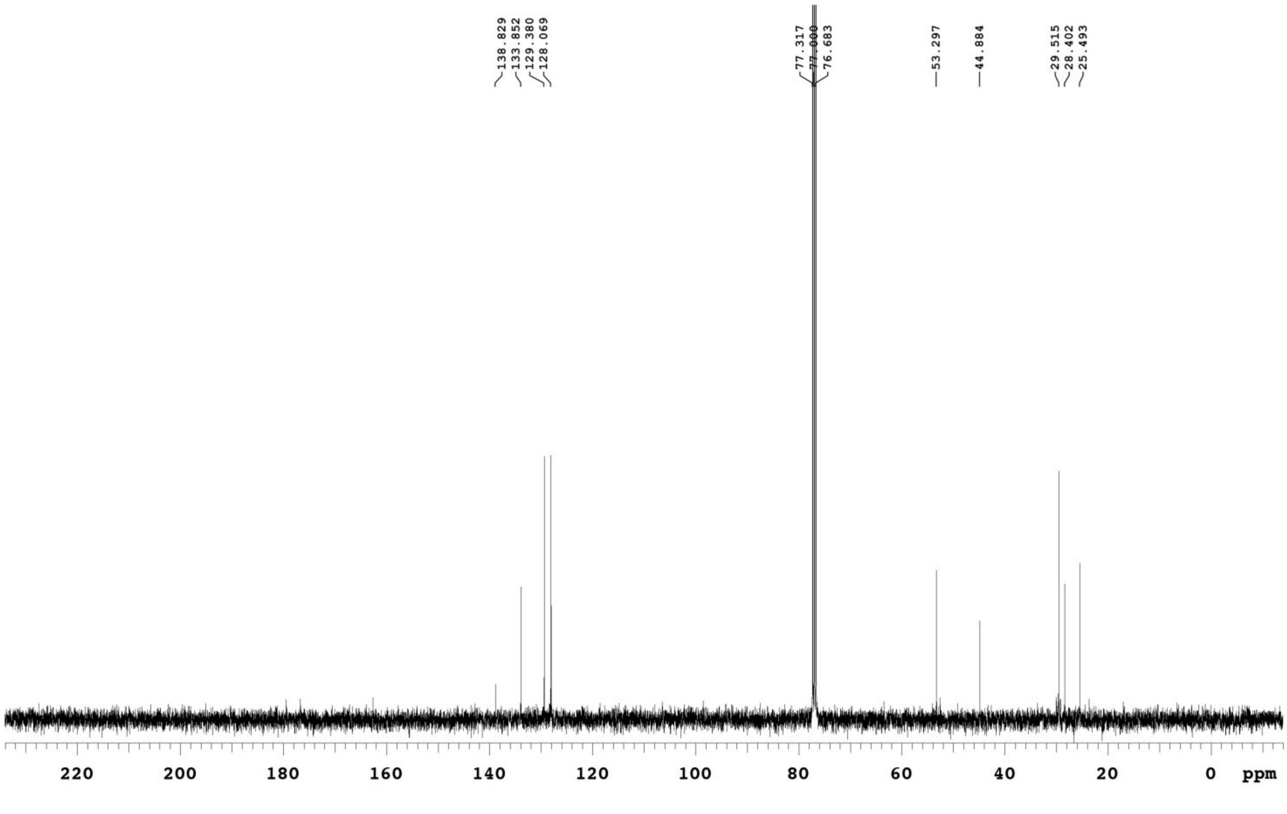

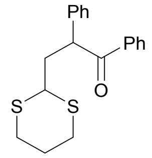
Ph
Ph
O
S
S

5n

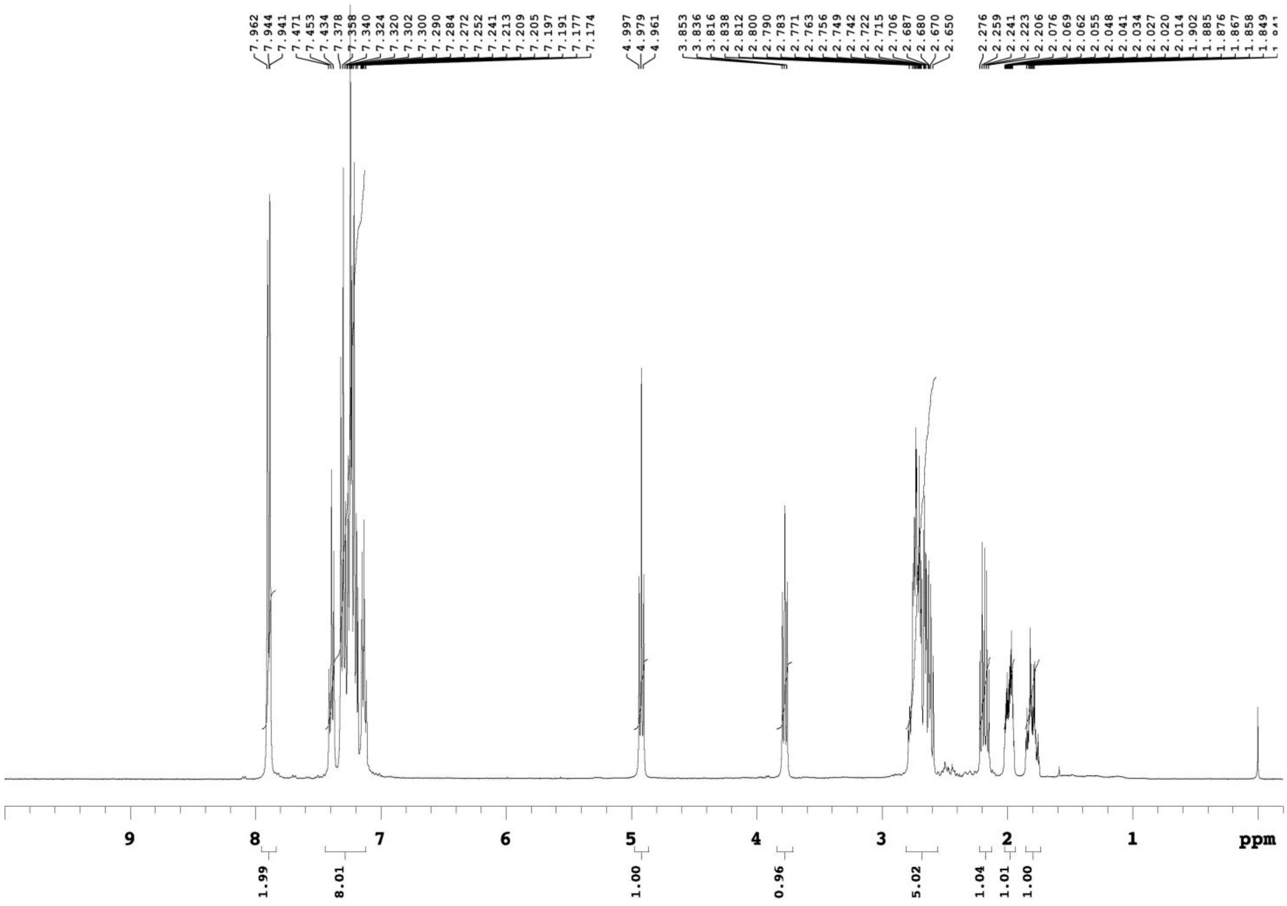
7.962
7.944
7.941
7.471
7.453
7.434
7.378
7.358
7.340
7.324
7.320
7.302
7.300
7.290
7.284
7.272
7.252
7.241
7.213
7.209
7.205
7.197
7.191
7.177
7.174
4.997
4.979
4.961
3.853
3.836
3.816
2.838
2.812
2.800
2.790
2.783
2.771
2.763
2.756
2.749
2.742
2.722
2.715
2.706
2.687
2.680
2.670
2.650
2.276
2.259
2.241
2.223
2.206
2.076
2.069
2.062
2.055
2.048
2.041
2.034
2.027
2.020
2.014
1.902
1.885
1.876
1.867
1.858
1.849
9
8
7
6
5
4
3
2
1
ppm
1.99
8.01
1.00
0.96
5.02
1.04
1.01
1.00

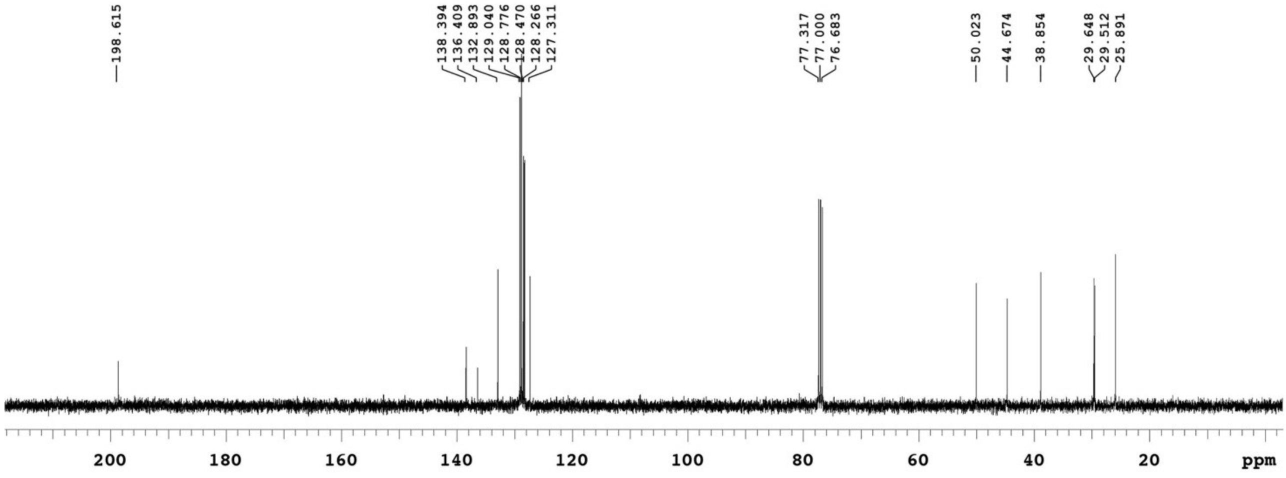
198.615
138.394
136.409
132.893
129.040
128.776
128.470
128.266
127.311
77.317
77.000
76.683
50.023
44.674
38.854
29.648
29.512
25.891
200
180
160
140
120
100
80
60
40
20
ppm

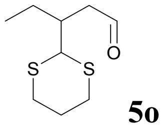
S
S
O

5o

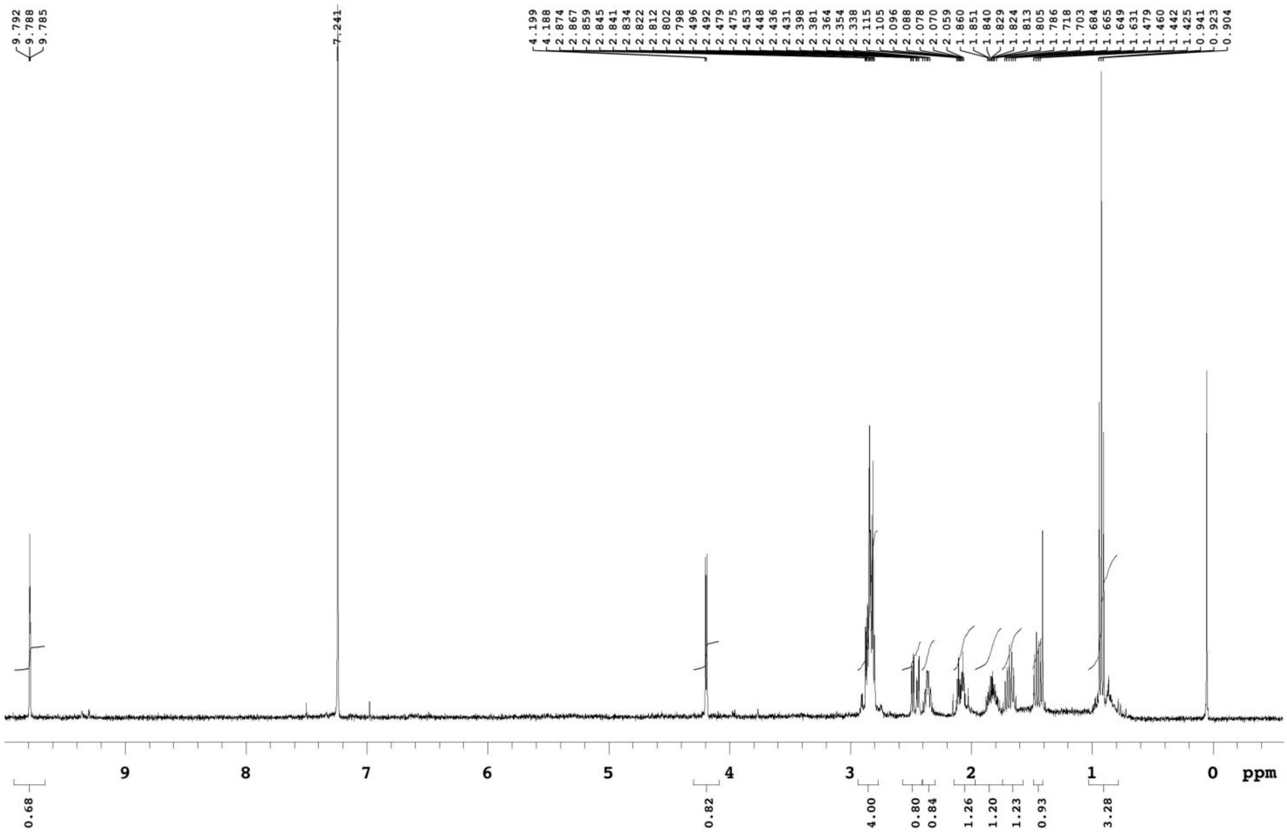
9.792
9.788
9.785
7.241
4.199
4.188
2.874
2.867
2.859
2.845
2.841
2.834
2.822
2.812
2.802
2.798
2.496
2.492
2.479
2.475
2.453
2.448
2.436
2.431
2.398
2.381
2.364
2.354
2.338
2.115
2.105
2.096
2.088
2.078
2.070
2.059
1.860
1.851
1.840
1.829
1.824
1.813
1.805
1.786
1.718
1.703
1.684
1.665
1.649
1.631
1.479
1.460
1.442
1.425
0.941
0.923
0.904
9
8
7
6
5
4
3
2
1
0
ppm
0.68
0.82
4.00
0.80
0.84
1.26
1.20
1.23
0.93
3.28

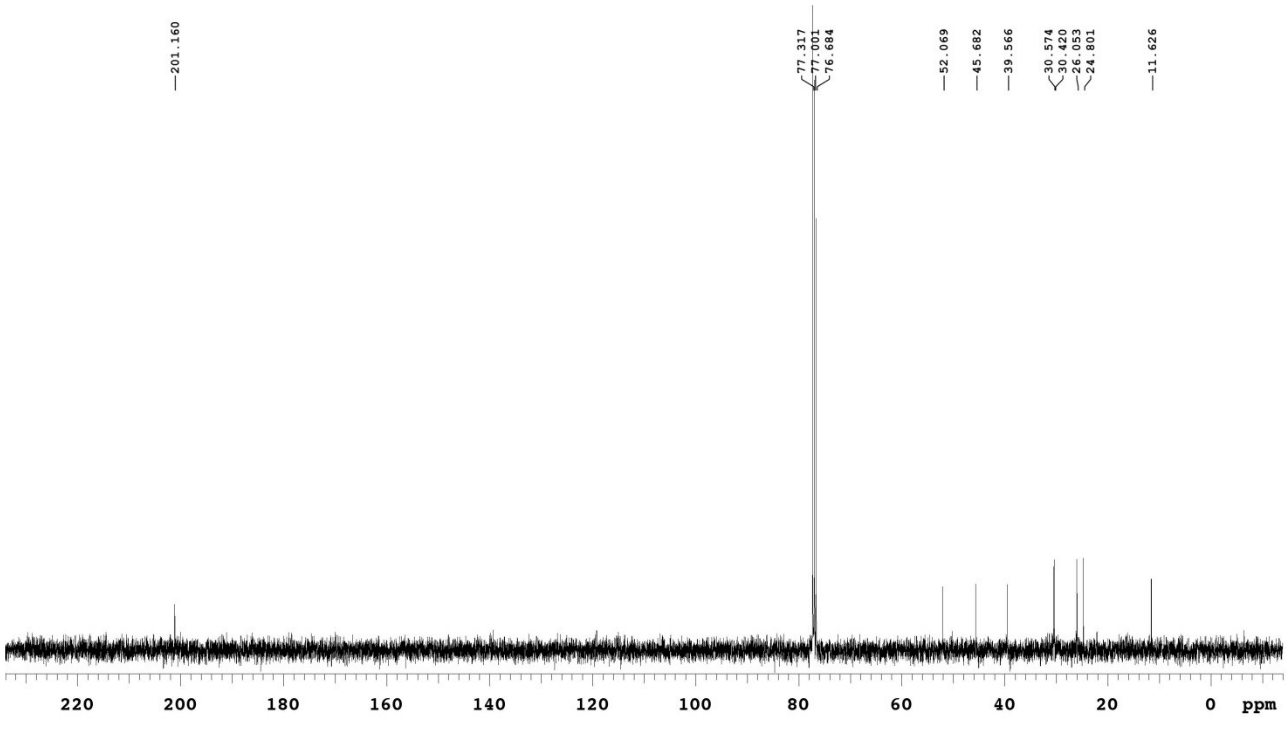
201.160
77.317
77.001
76.684
52.069
45.682
39.566
30.574
30.420
26.053
24.801
11.626
220
200
180
160
140
120
100
80
60
40
20
0
ppm

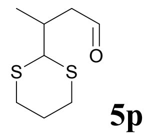
S
S
O

5p

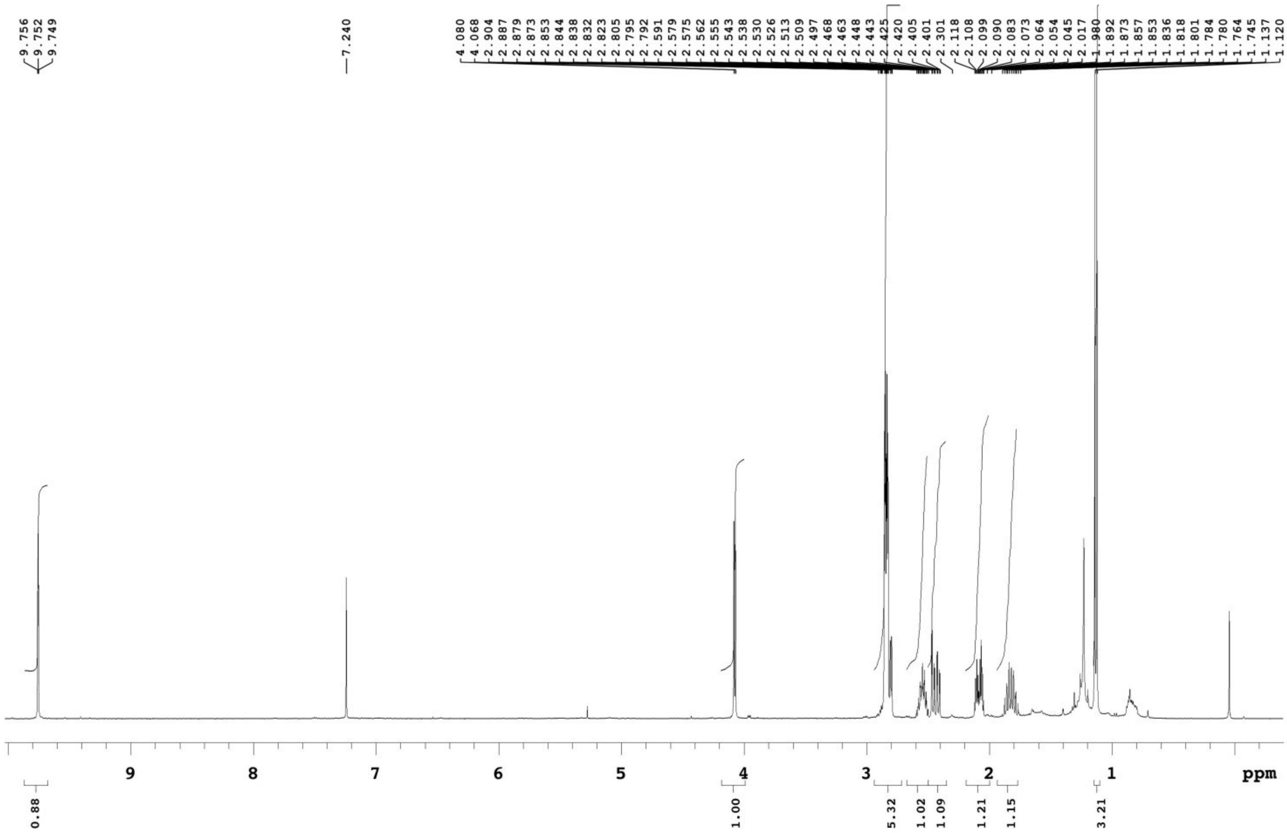
9.756
9.752
9.749
7.240
4.080
4.068
2.904
2.887
2.879
2.873
2.853
2.844
2.838
2.832
2.823
2.805
2.795
2.792
2.591
2.579
2.575
2.562
2.555
2.543
2.538
2.530
2.526
2.513
2.509
2.497
2.468
2.463
2.448
2.443
2.425
2.420
2.405
2.401
2.301
2.118
2.108
2.099
2.090
2.083
2.073
2.064
2.054
2.045
2.017
1.980
1.892
1.873
1.857
1.853
1.836
1.818
1.801
1.784
1.780
1.764
1.745
1.137
1.120
9
8
7
6
5
4
3
2
1
ppm
0.88
1.00
5.32
1.02
1.09
1.21
1.15
3.21

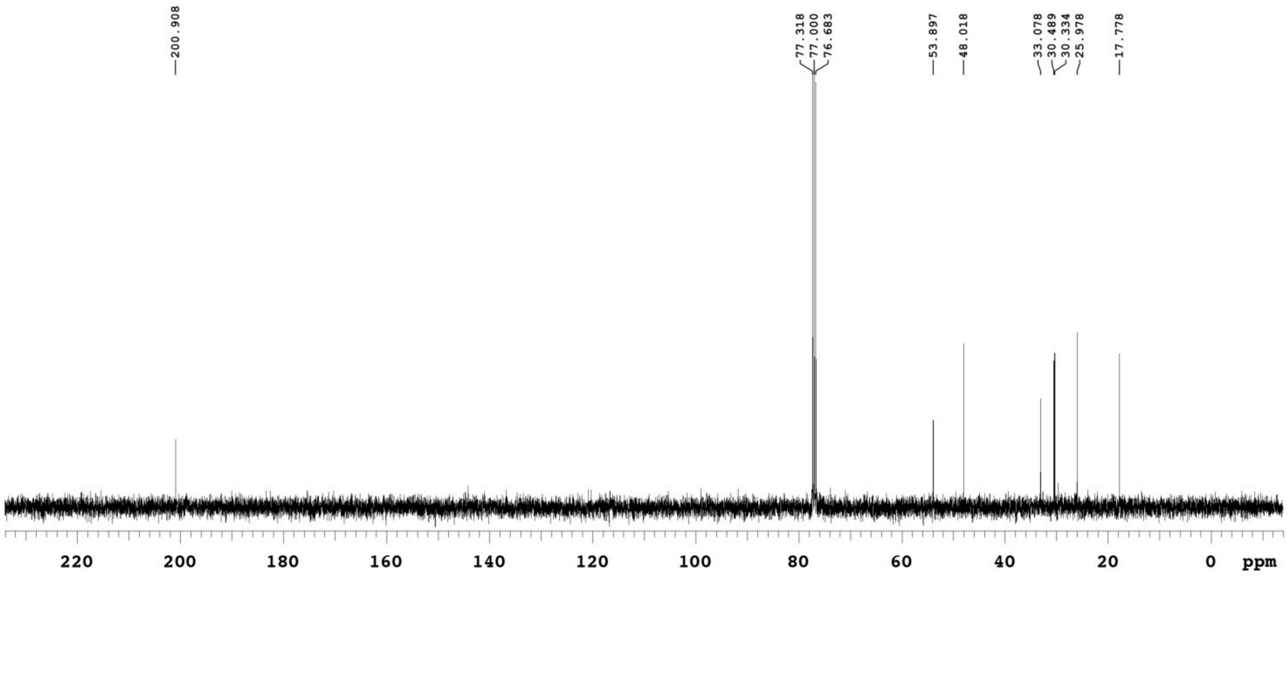
200.908
77.318
77.000
76.683
53.897
48.018
33.078
30.489
30.334
25.978
17.778
220
200
180
160
140
120
100
80
60
40
20
0
ppm

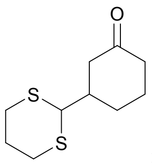
O
S
S

5q

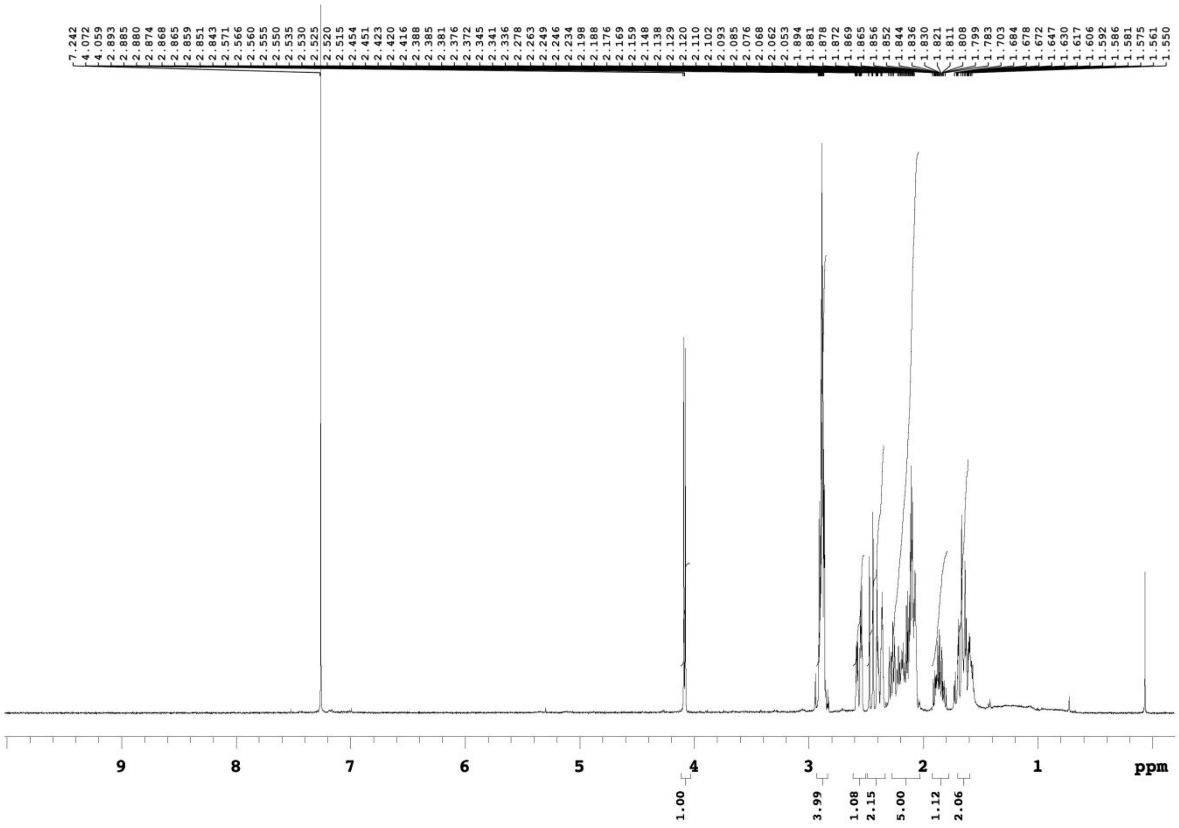
7.242
4.072
4.059
2.893
2.885
2.880
2.874
2.868
2.865
2.859
2.851
2.843
2.571
2.566
2.560
2.555
2.550
2.535
2.530
2.525
2.520
2.515
2.454
2.451
2.423
2.420
2.416
2.388
2.385
2.381
2.376
2.372
2.345
2.341
2.336
2.278
2.263
2.249
2.246
2.234
2.198
2.188
2.176
2.169
2.159
2.148
2.138
2.129
2.120
2.110
2.102
2.093
2.085
2.076
2.068
2.062
2.053
1.894
1.881
1.878
1.872
1.869
1.865
1.856
1.852
1.844
1.836
1.830
1.821
1.811
1.808
1.799
1.783
1.703
1.684
1.678
1.672
1.647
1.630
1.617
1.606
1.592
1.586
1.581
1.575
1.561
1.550
9
8
7
6
5
4
3
2
1
ppm
1.00
3.99
1.08
2.15
5.00
1.12
2.06

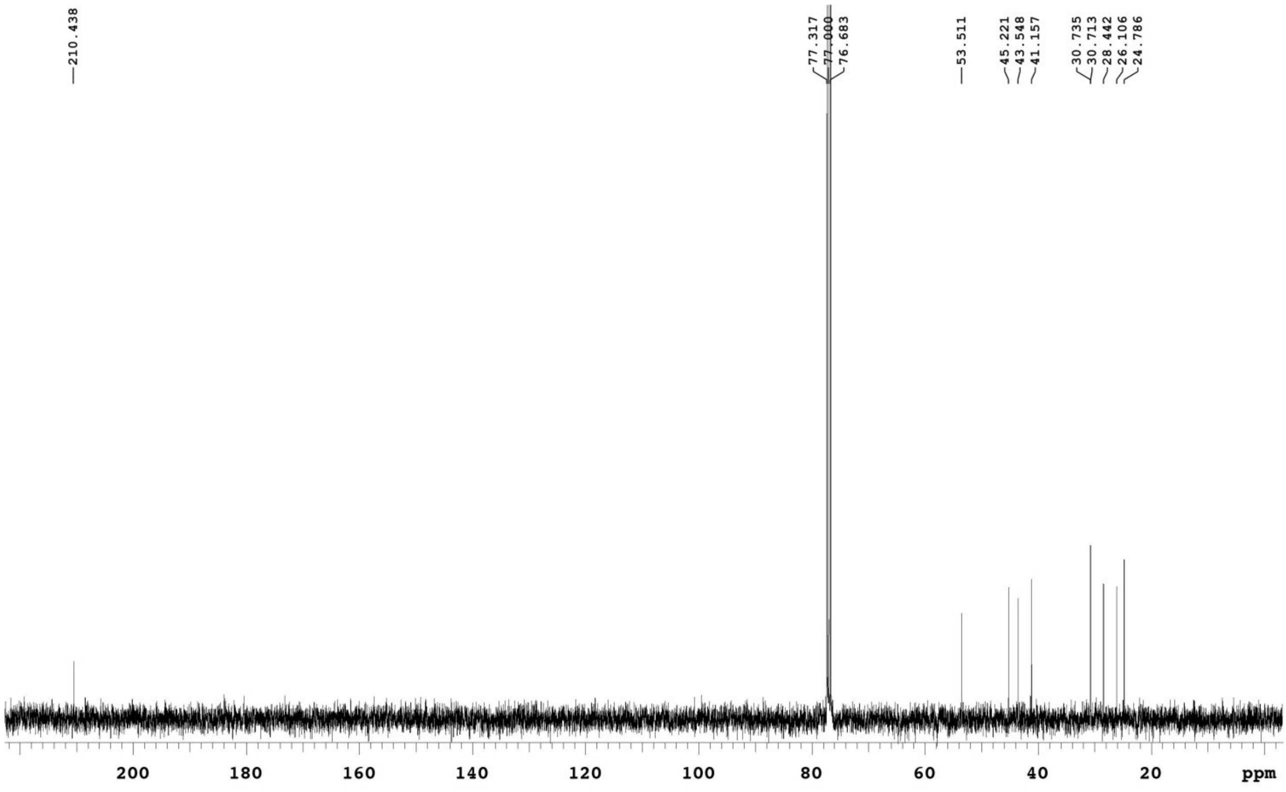
210.438
77.317
77.000
76.683
53.511
45.221
43.548
41.157
30.735
30.713
28.442
26.106
24.786
200
180
160
140
120
100
80
60
40
20
ppm

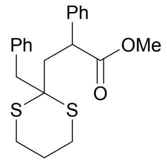

Ph
Ph
OMe
O
S
S

5r

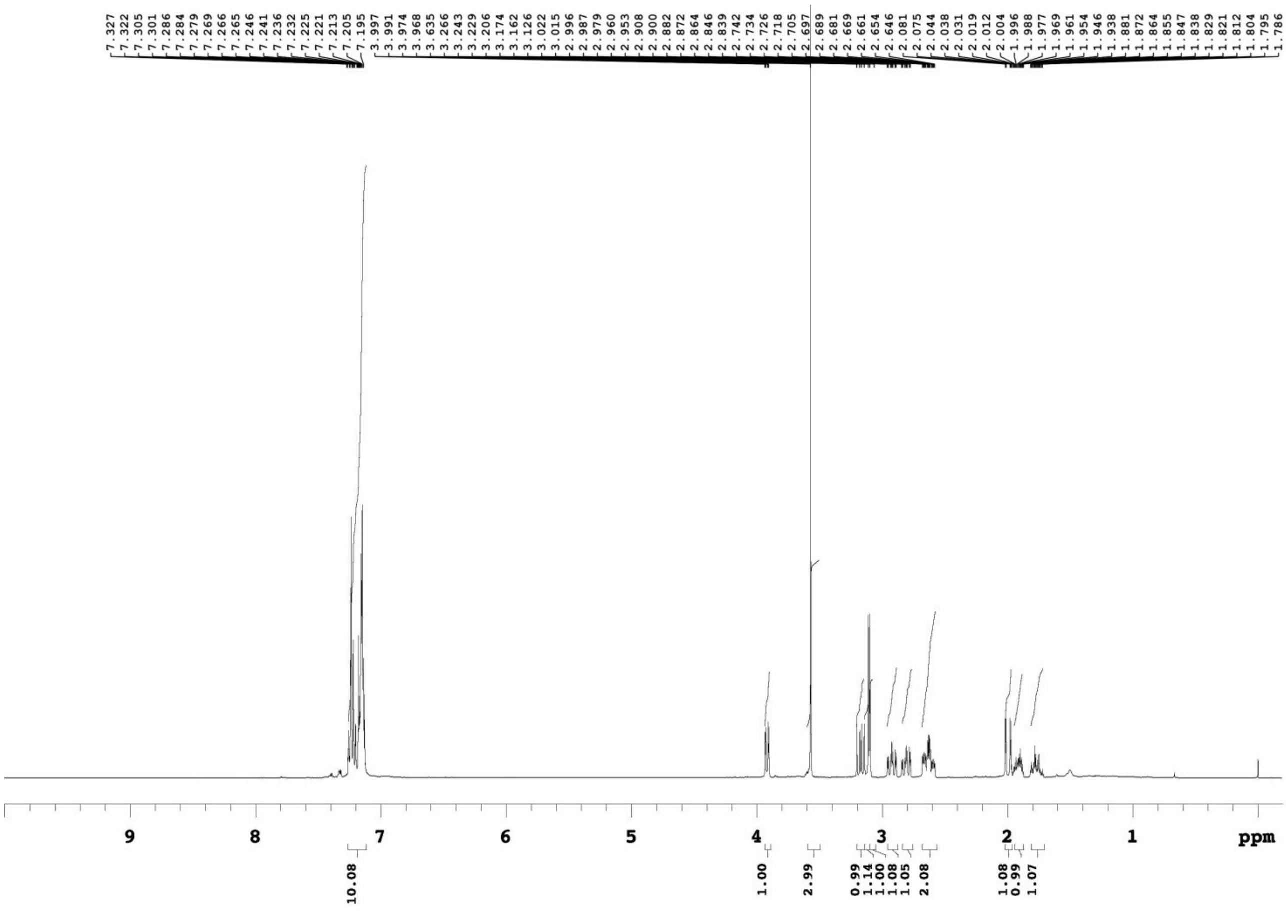

7.327 7.322 7.305 7.301 7.286 7.284 7.279 7.269 7.266 7.265 7.246 7.241 7.236 7.232 7.225 7.221 7.213 7.205 7.195 3.997 3.991 3.974 3.968 3.635 3.266 3.243 3.229 3.206 3.174 3.162 3.126 3.022 3.015 2.996 2.987 2.979 2.960 2.953 2.908 2.900 2.882 2.872 2.864 2.846 2.839 2.742 2.734 2.726 2.718 2.705 2.697 2.689 2.681 2.669 2.661 2.654 2.646 2.081 2.075 2.044 2.038 2.031 2.019 2.012 2.004 1.996 1.988 1.977 1.969 1.961 1.954 1.946 1.938 1.881 1.872 1.864 1.855 1.847 1.838 1.829 1.821 1.812 1.804 1.795 1.786
9 8 7 6 5 4 3 2 1 ppm
10.08 1.00 2.99 0.99 1.14 1.00 1.08 1.05 2.08 1.08 0.99 1.07

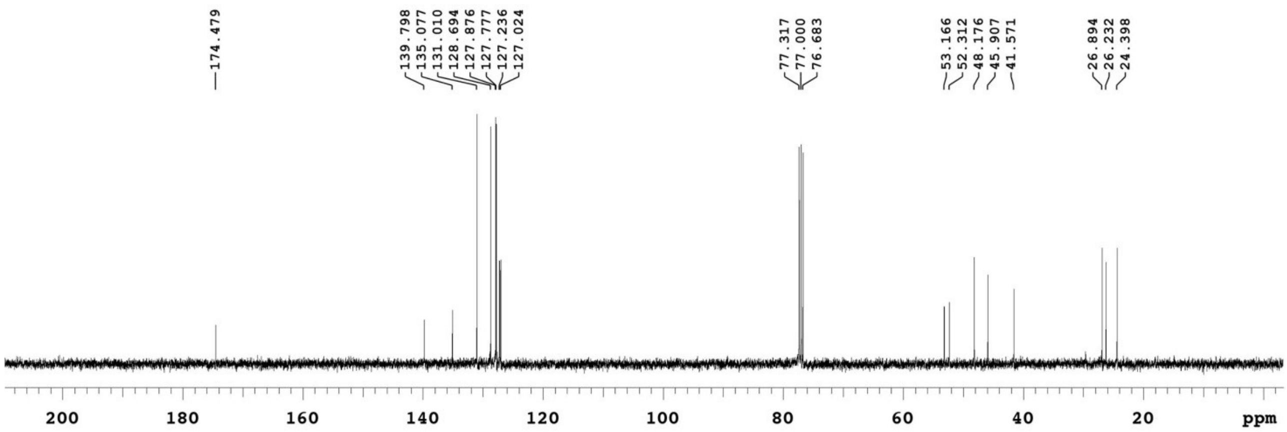

174.479
139.798 135.077 131.010 128.694 127.876 127.777 127.236 127.024
77.317 77.000 76.683
53.166 52.312 48.176 45.907 41.571
26.894 26.232 24.398
200 180 160 140 120 100 80 60 40 20 ppm

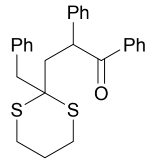

Ph
Ph
Ph
O
S
S

5s

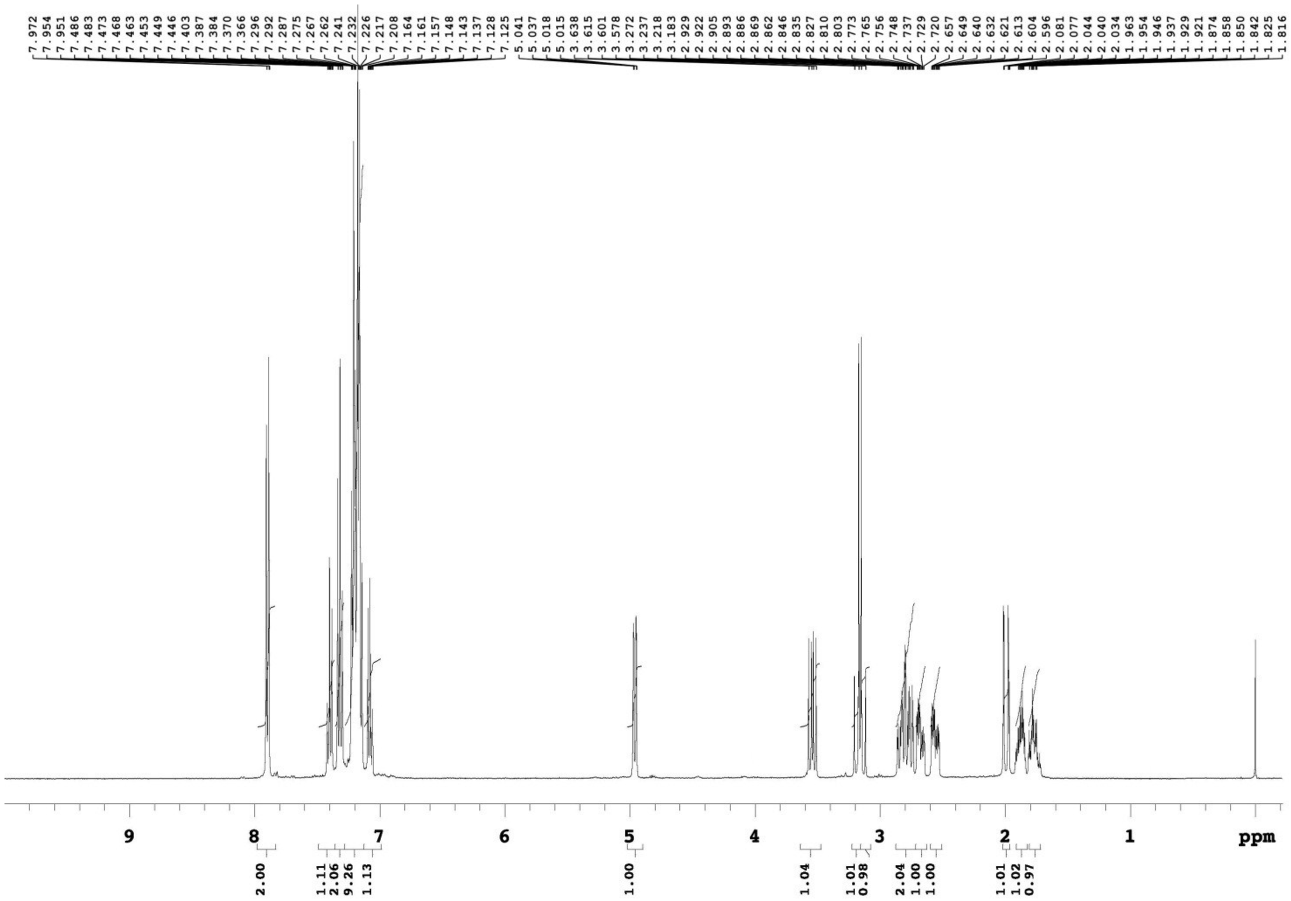

7.972
7.954
7.951
7.486
7.483
7.473
7.468
7.463
7.453
7.449
7.446
7.403
7.387
7.384
7.370
7.366
7.296
7.292
7.287
7.275
7.267
7.262
7.241
7.232
7.226
7.217
7.208
7.164
7.161
7.157
7.148
7.143
7.137
7.128
7.125
5.041
5.037
5.018
5.015
3.638
3.615
3.601
3.578
3.272
3.237
3.218
3.183
2.929
2.922
2.905
2.893
2.886
2.869
2.862
2.846
2.835
2.827
2.810
2.803
2.773
2.765
2.756
2.748
2.737
2.729
2.720
2.657
2.649
2.640
2.632
2.621
2.613
2.604
2.596
2.081
2.077
2.044
2.040
2.034
1.963
1.954
1.946
1.937
1.929
1.921
1.874
1.858
1.850
1.842
1.825
1.816
9
8
7
6
5
4
3
2
1
ppm
2.00
1.11
2.06
9.26
1.13
1.00
1.04
1.01
0.98
2.04
1.00
1.00
1.01
1.02
0.97

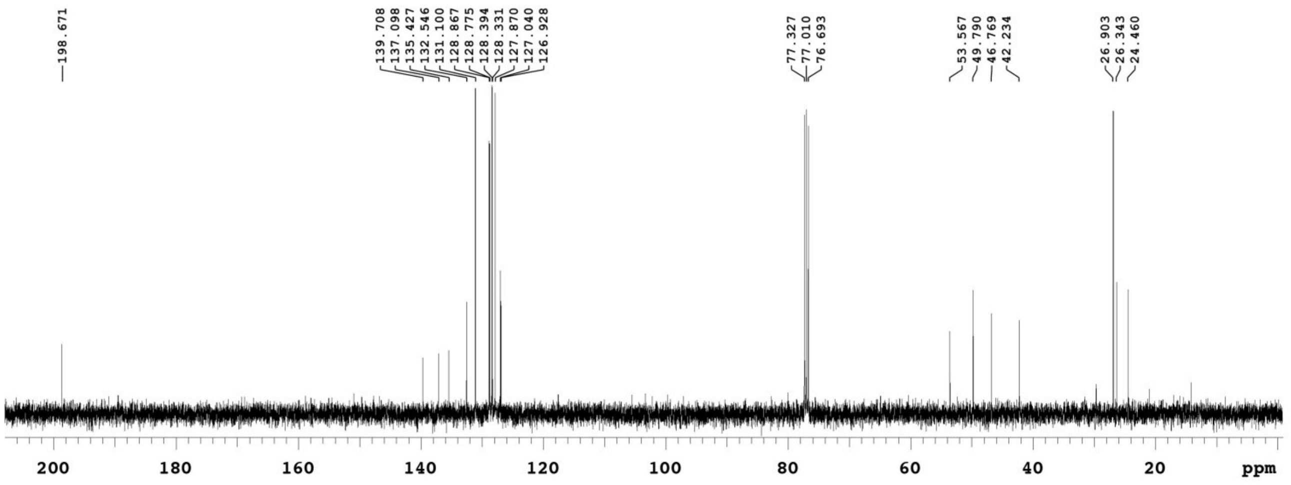

198.671
139.708
137.098
135.427
132.546
131.100
128.867
128.775
128.394
128.331
127.870
127.040
126.928
77.327
77.010
76.693
53.567
49.790
46.769
42.234
26.903
26.343
24.460
200
180
160
140
120
100
80
60
40
20
ppm

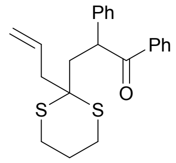
Ph
Ph
O
S
S

5t

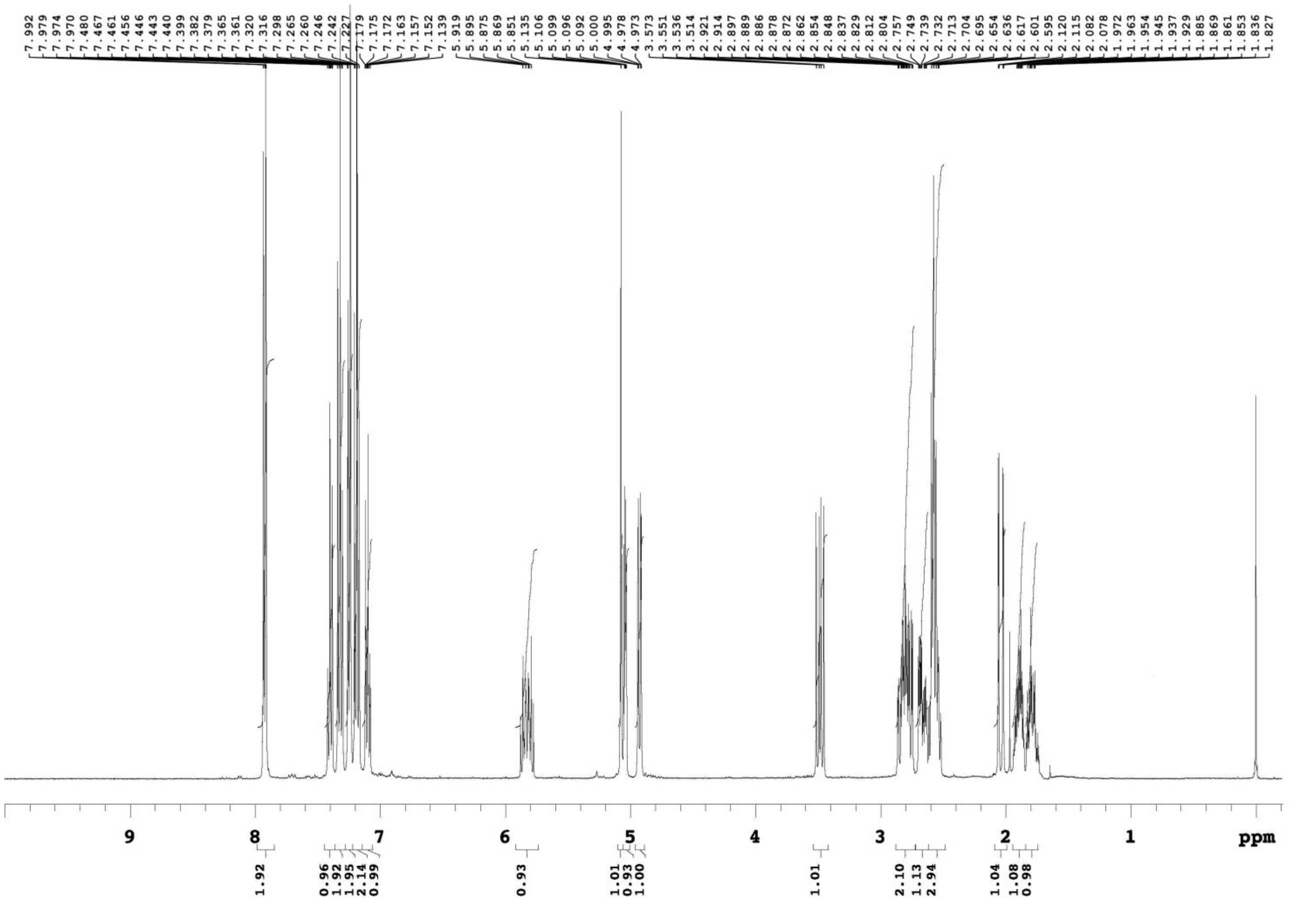
7.992
7.979
7.974
7.970
7.480
7.467
7.461
7.456
7.446
7.443
7.440
7.399
7.382
7.379
7.365
7.361
7.320
7.316
7.298
7.265
7.260
7.246
7.242
7.227
7.179
7.175
7.172
7.163
7.157
7.152
7.139
5.919
5.895
5.875
5.869
5.851
5.135
5.106
5.099
5.096
5.092
5.000
4.995
4.978
4.973
3.573
3.551
3.536
3.514
2.921
2.914
2.897
2.889
2.886
2.878
2.872
2.862
2.854
2.848
2.837
2.829
2.812
2.804
2.757
2.749
2.739
2.732
2.713
2.704
2.695
2.654
2.636
2.617
2.601
2.595
2.120
2.115
2.082
2.078
1.972
1.963
1.954
1.945
1.937
1.929
1.885
1.869
1.861
1.853
1.836
1.827
9
8
7
6
5
4
3
2
1
ppm
1.92
0.96
1.92
1.95
2.14
0.99
0.93
1.01
0.93
1.00
1.01
2.10
1.13
2.94
1.04
1.08
0.98

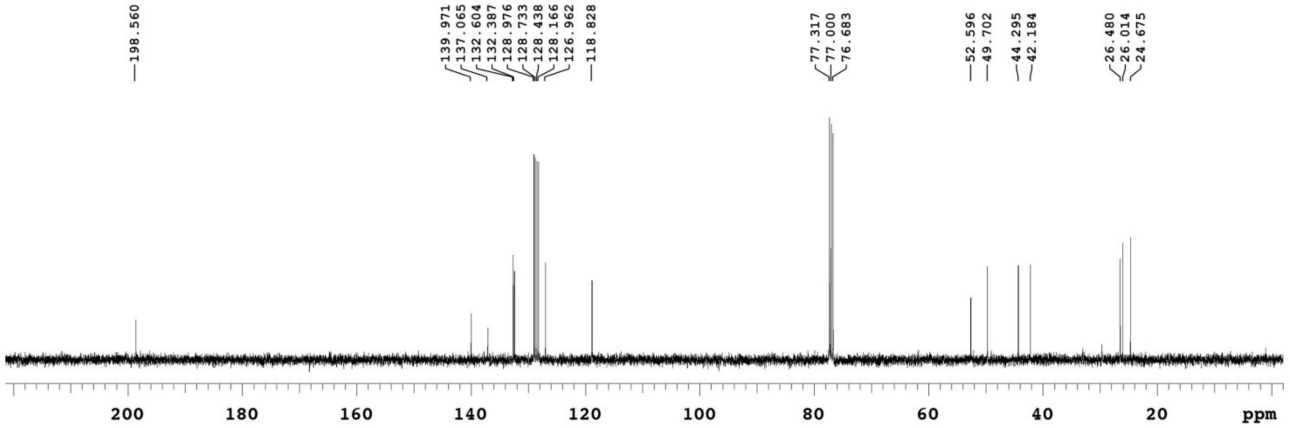
198.560
139.971
137.065
132.604
132.387
128.976
128.733
128.438
128.166
126.962
118.828
77.317
77.000
76.683
52.596
49.702
44.295
42.184
26.480
26.014
24.675
200
180
160
140
120
100
80
60
40
20
ppm

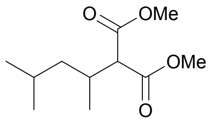
O
OMe
OMe
O

6

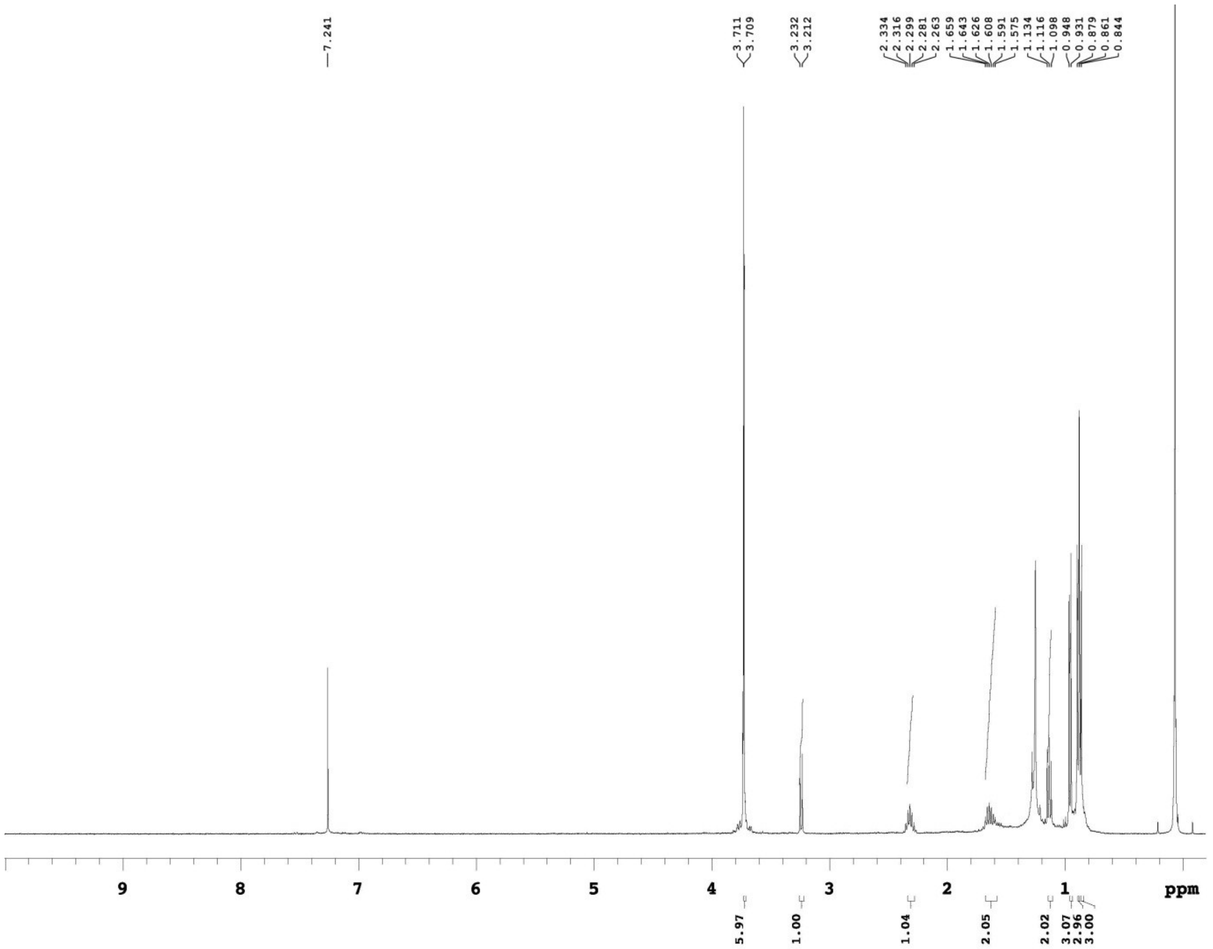
7.241
3.711
3.709
3.232
3.212
2.334
2.316
2.299
2.281
2.263
1.659
1.643
1.626
1.608
1.591
1.575
1.134
1.116
1.098
0.948
0.931
0.879
0.861
0.844
9
8
7
6
5
4
3
2
1
ppm
5.97
1.00
1.04
2.05
2.02
3.07
2.96
3.00

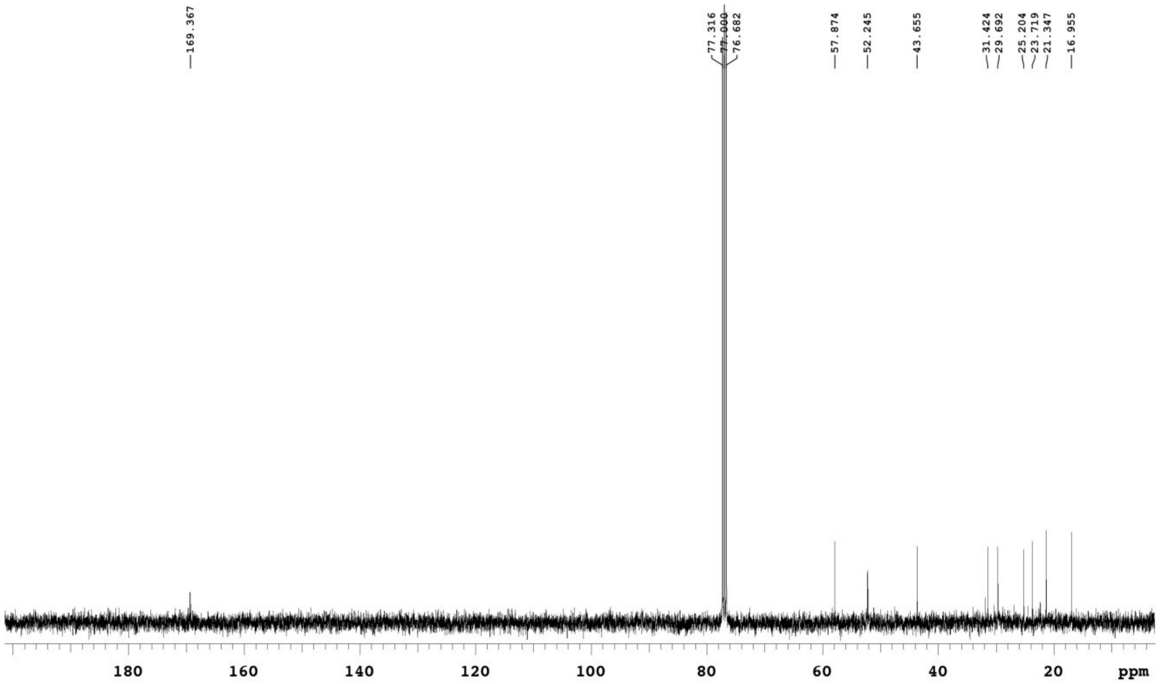
169.367
77.316
77.000
76.682
57.874
52.245
43.655
31.424
29.692
25.204
23.719
21.347
16.955
180
160
140
120
100
80
60
40
20
ppm

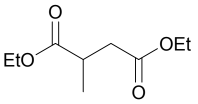

7

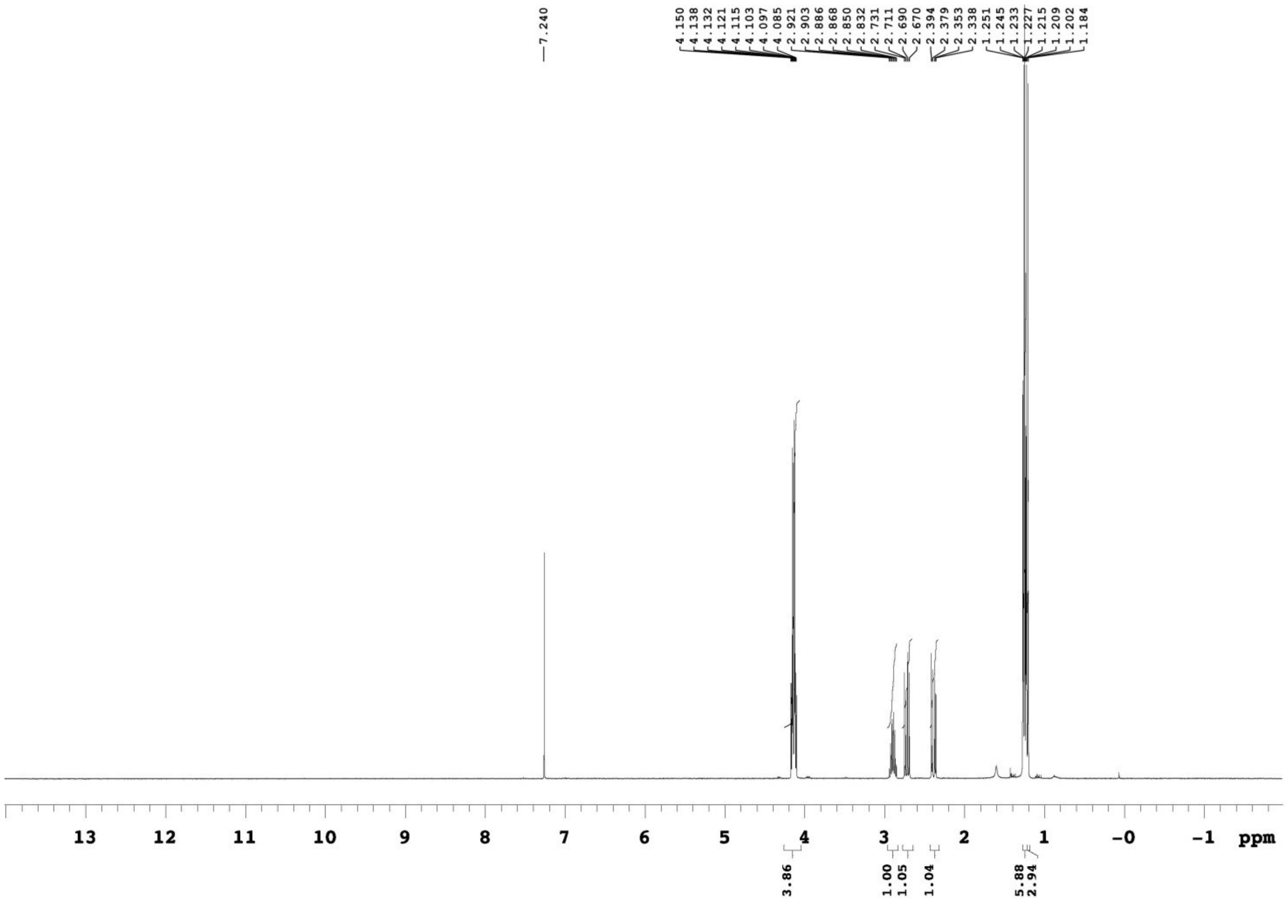
7.240
4.150 4.138 4.132 4.121 4.115 4.103 4.097 4.085 2.921 2.903 2.886 2.868 2.850 2.832 2.731 2.711 2.690 2.670 2.394 2.379 2.353 2.338 1.251 1.245 1.233 1.227 1.215 1.209 1.202 1.184
13 12 11 10 9 8 7 6 5 4 3 2 1 -0 -1 ppm
3.86 1.00 1.05 1.04 5.88 2.94

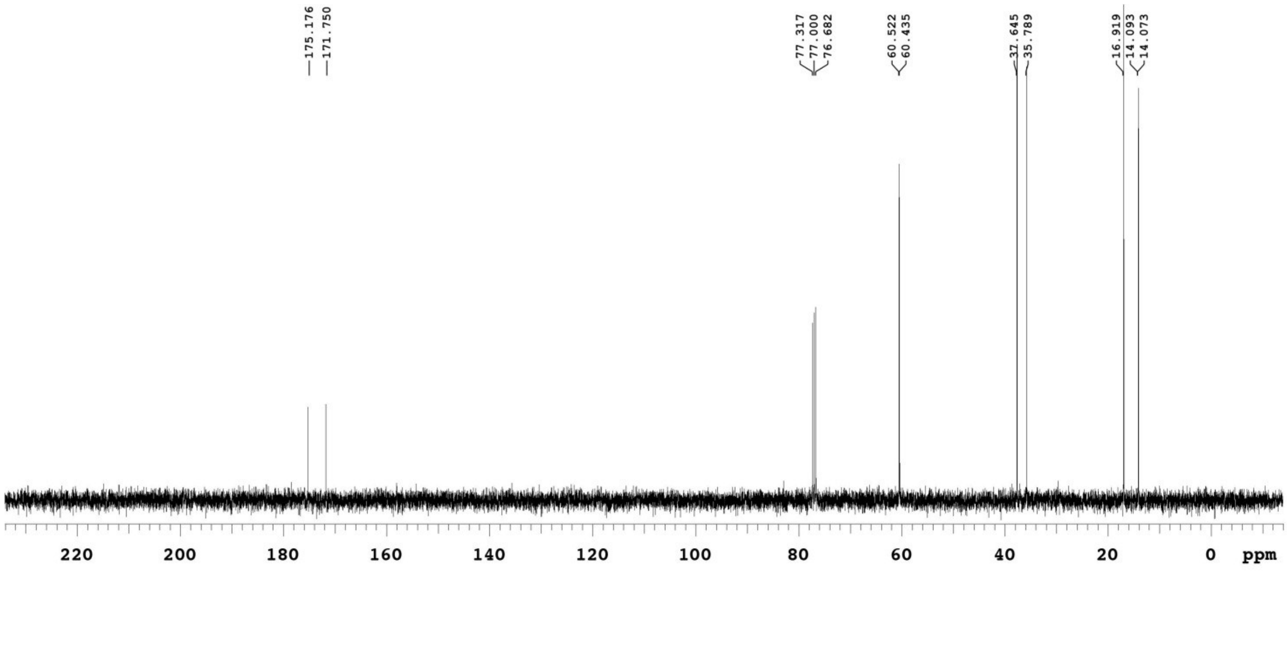
175.176 171.750
77.317 77.000 76.682
60.522 60.435
37.645 35.789
16.919 14.093 14.073
220 200 180 160 140 120 100 80 60 40 20 0 ppm

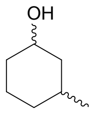

8

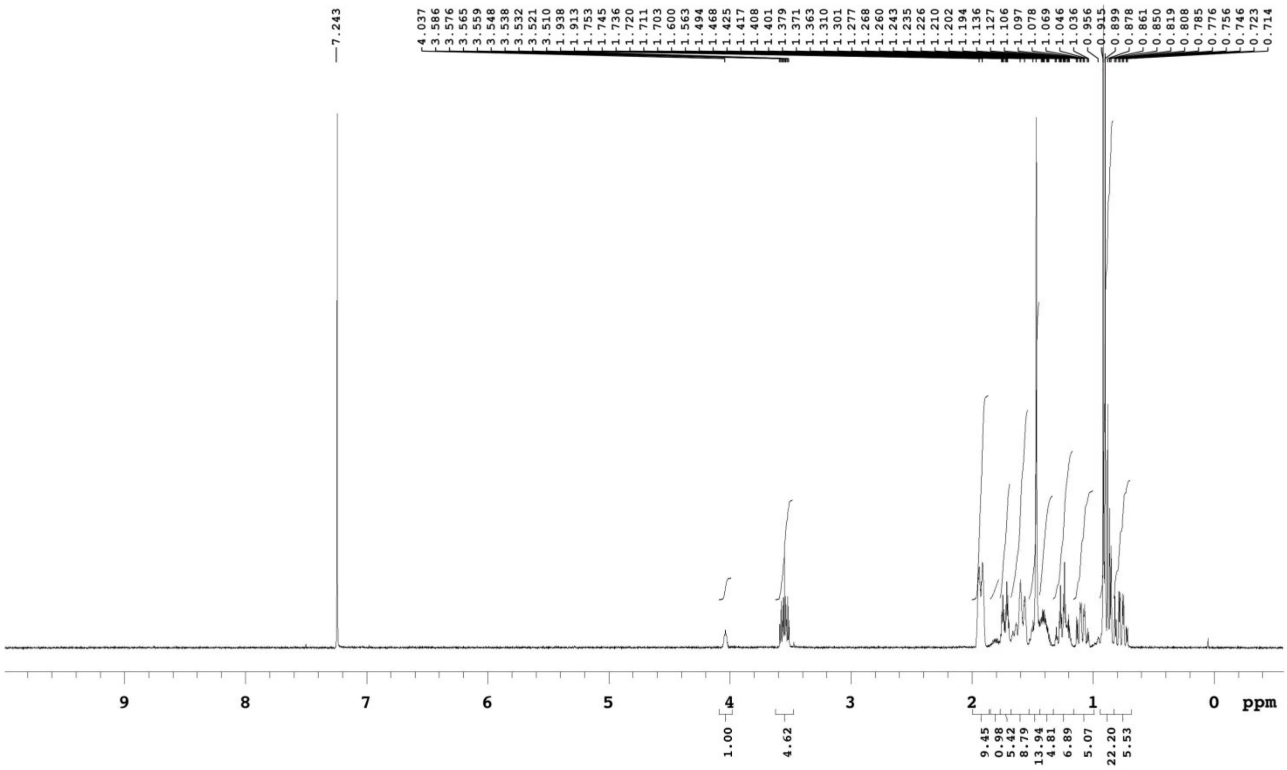

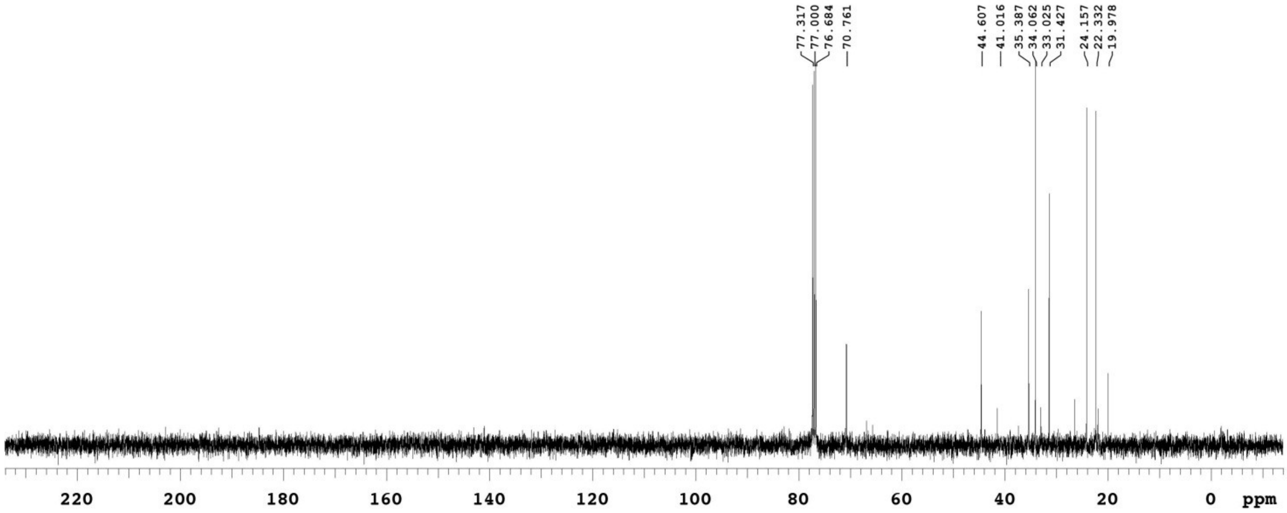

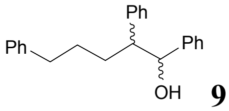
Ph
Ph
Ph
OH

9

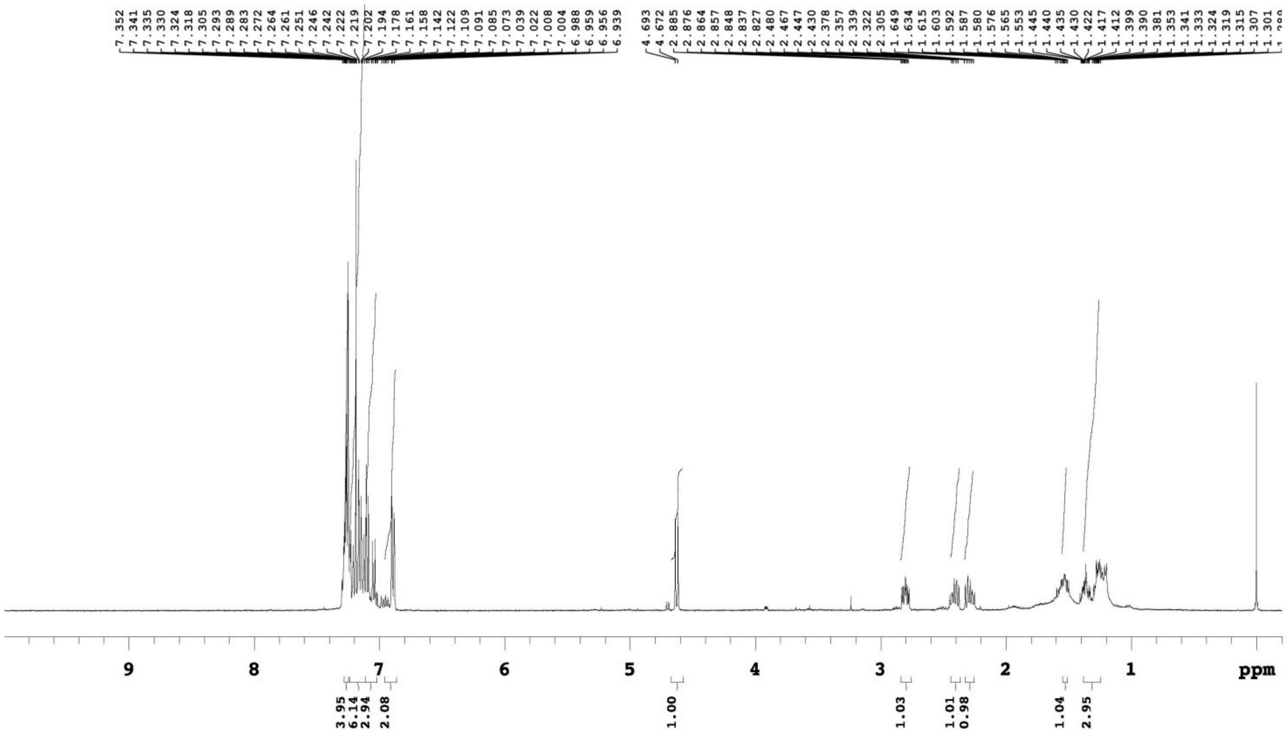
7.352
7.341
7.335
7.330
7.324
7.318
7.305
7.293
7.289
7.283
7.272
7.264
7.261
7.251
7.246
7.242
7.222
7.219
7.202
7.194
7.178
7.161
7.158
7.142
7.122
7.109
7.091
7.085
7.073
7.039
7.022
7.008
7.004
6.988
6.959
6.956
6.939
4.693
4.672
2.885
2.876
2.864
2.857
2.848
2.837
2.827
2.480
2.467
2.447
2.430
2.378
2.357
2.339
2.322
2.305
1.649
1.634
1.615
1.603
1.592
1.587
1.580
1.576
1.565
1.553
1.445
1.440
1.435
1.430
1.422
1.417
1.412
1.399
1.390
1.381
1.353
1.341
1.333
1.324
1.319
1.315
1.307
1.301
9
8
7
6
5
4
3
2
1
ppm
3.95
6.14
2.94
2.08
1.00
1.03
1.01
0.98
1.04
2.95

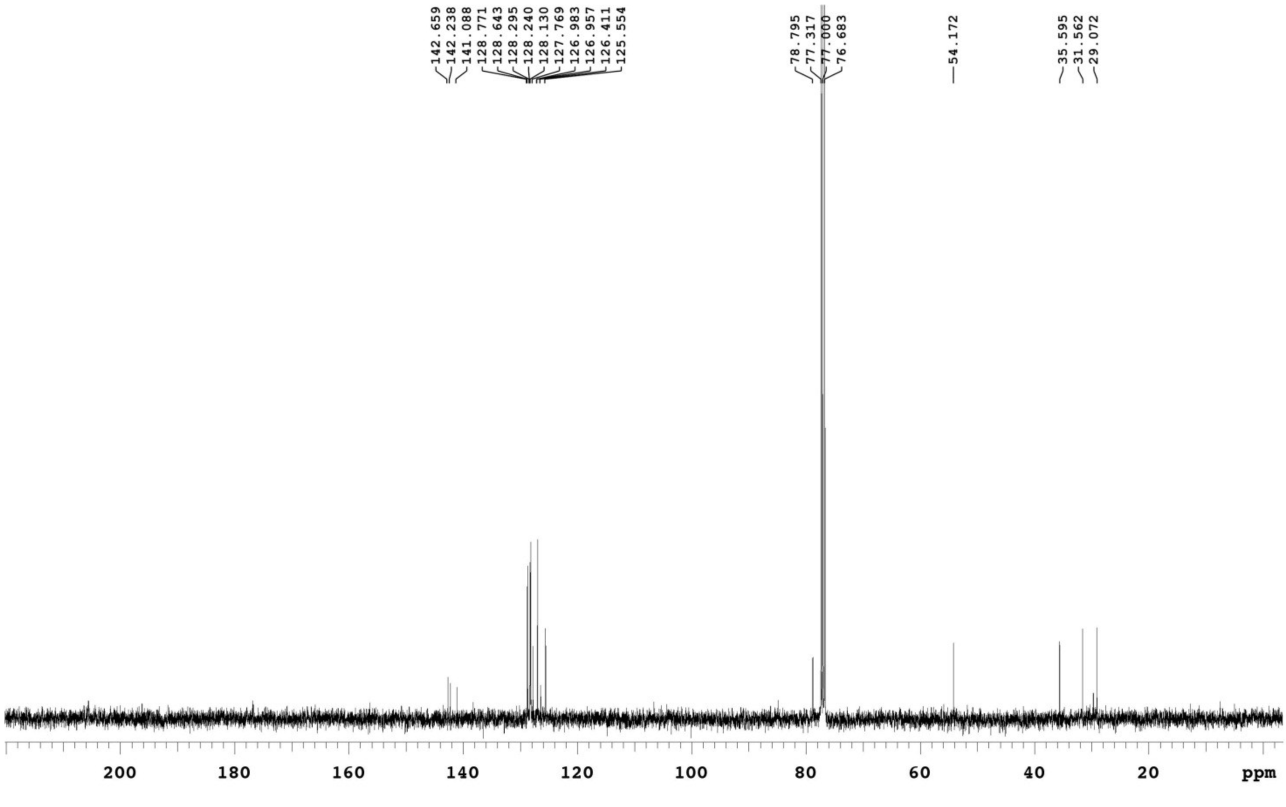
142.659
142.238
141.088
128.771
128.643
128.295
128.240
128.130
127.769
126.983
126.957
126.411
125.554
78.795
77.317
77.000
76.683
54.172
35.595
31.562
29.072
200
180
160
140
120
100
80
60
40
20
ppm

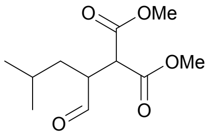
O
OMe
OMe
O
O

10

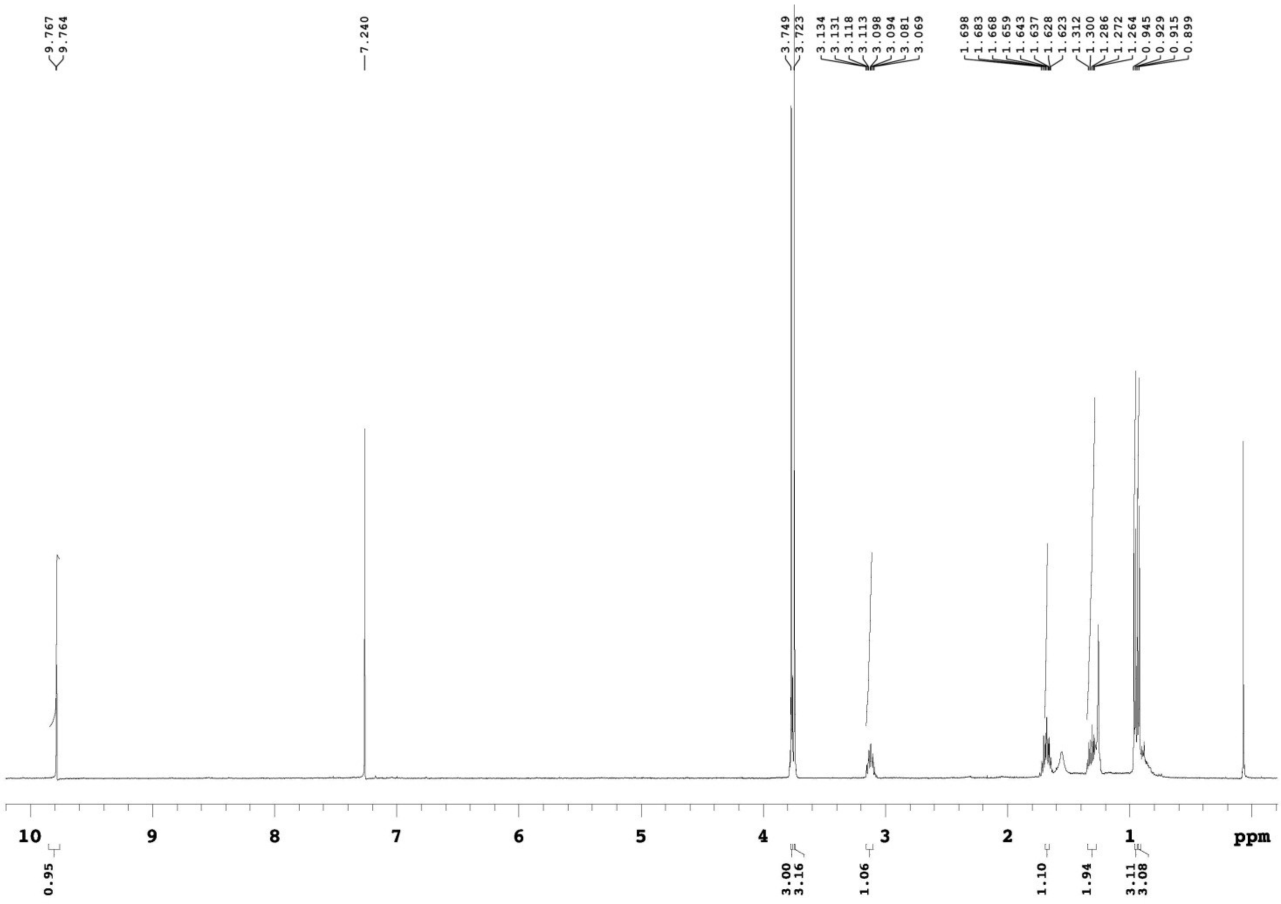
9.767
9.764
7.240
3.749
3.723
3.134
3.131
3.118
3.113
3.098
3.094
3.081
3.069
1.698
1.683
1.668
1.659
1.643
1.637
1.628
1.623
1.312
1.300
1.286
1.272
1.264
0.945
0.929
0.915
0.899
10
9
8
7
6
5
4
3
2
1
ppm
0.95
3.00
3.16
1.06
1.10
1.94
3.11
3.08

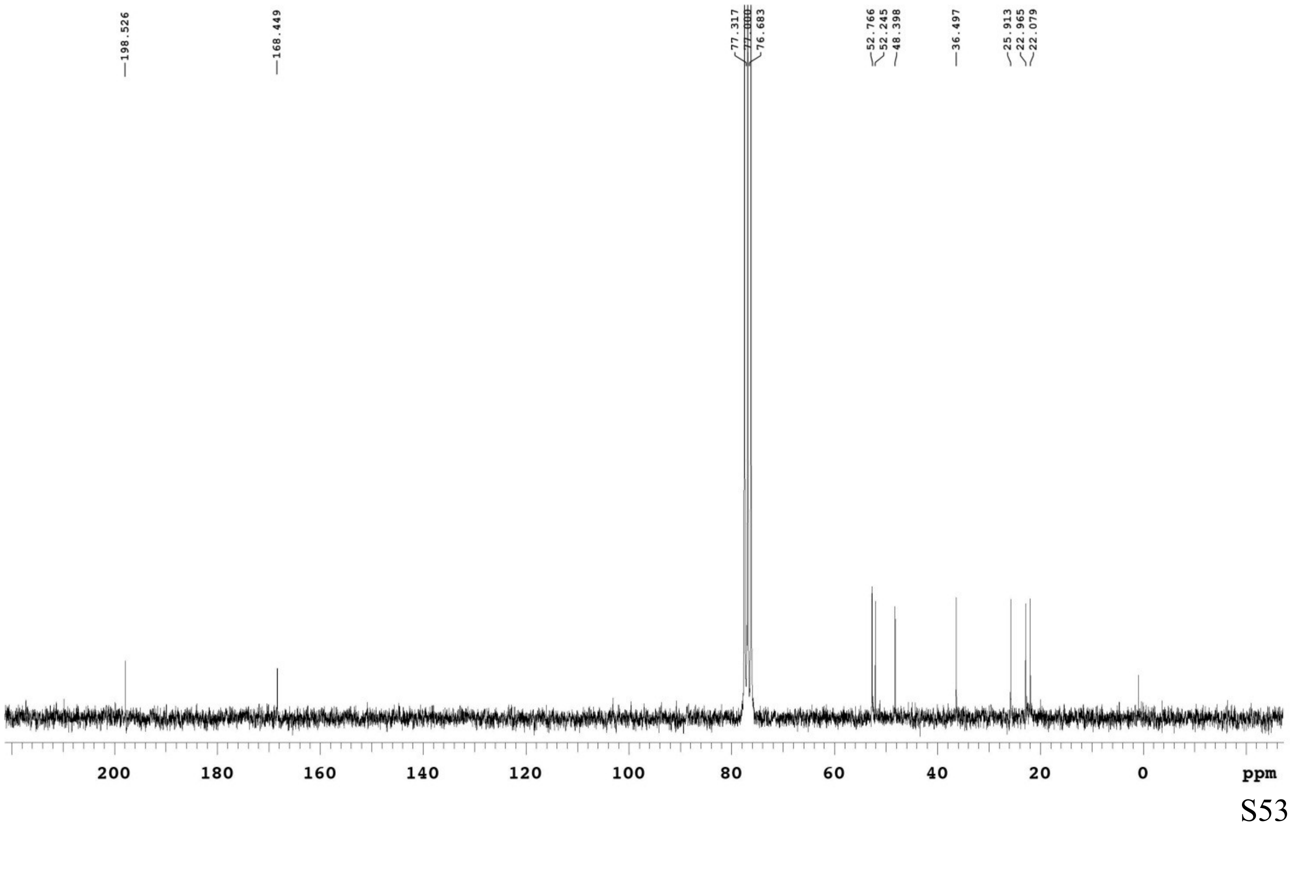
198.526
168.449
77.317
76.683
52.766
52.245
48.398
36.497
25.913
22.965
22.079
200
180
160
140
120
100
80
60
40
20
0
ppm

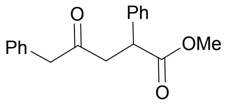

O
Ph
Ph
OMe
O

11

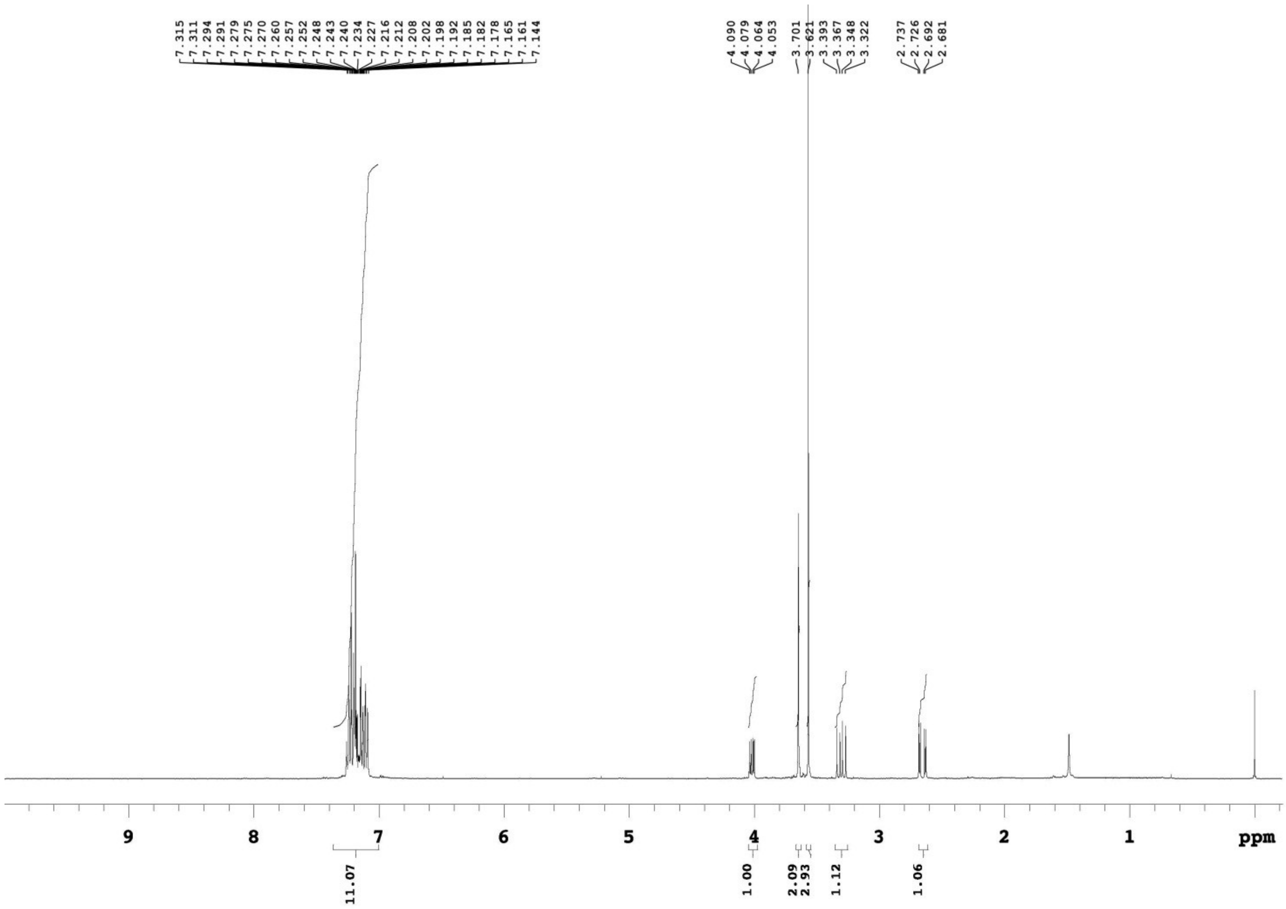

7.315
7.311
7.294
7.291
7.279
7.275
7.270
7.260
7.257
7.252
7.248
7.243
7.240
7.234
7.227
7.216
7.212
7.208
7.202
7.198
7.192
7.185
7.182
7.178
7.165
7.161
7.144
4.090
4.079
4.064
4.053
3.701
3.621
3.393
3.367
3.348
3.322
2.737
2.726
2.692
2.681
9
8
7
6
5
4
3
2
1
ppm
11.07
1.00
2.09
2.93
1.12
1.06

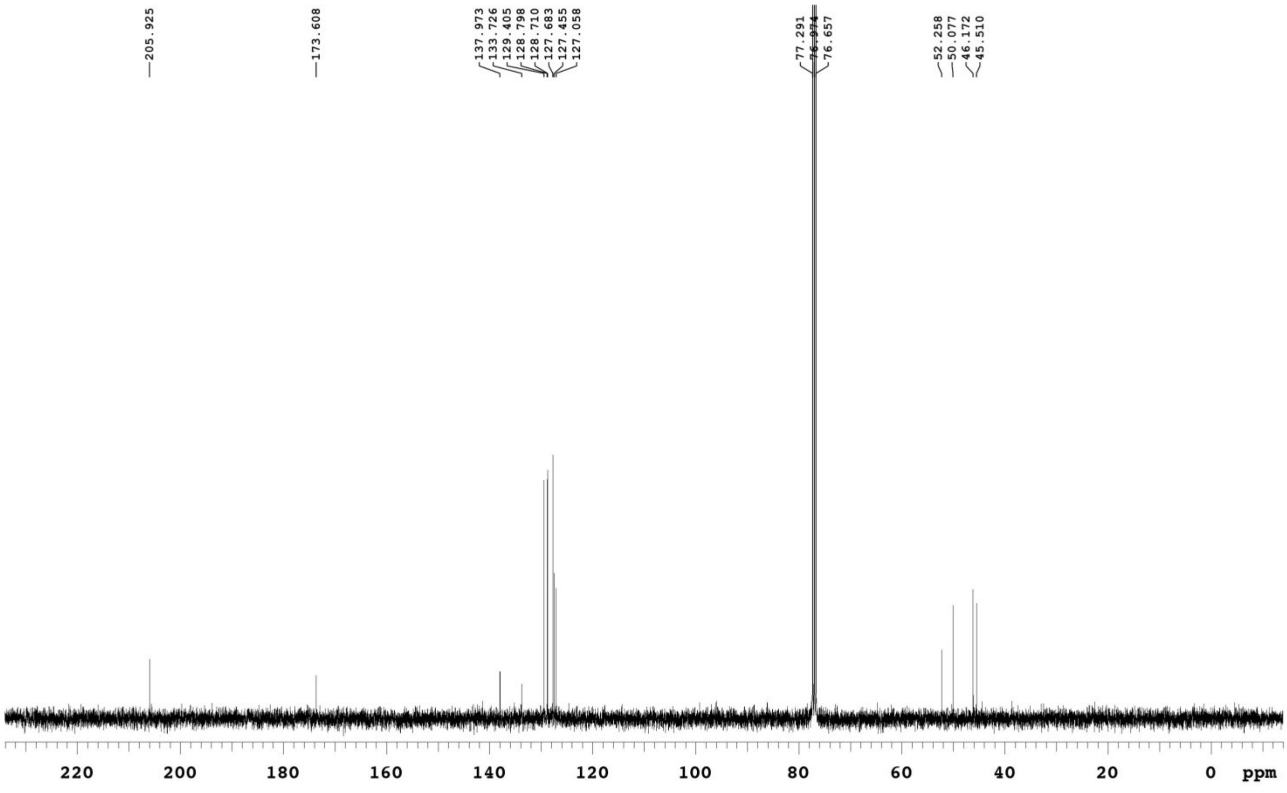

205.925
173.608
137.973
133.726
129.405
128.798
128.710
127.683
127.455
127.058
77.291
76.657
52.258
50.077
46.172
45.510
220
200
180
160
140
120
100
80
60
40
20
0
ppm

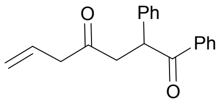

O
Ph
Ph
O

12

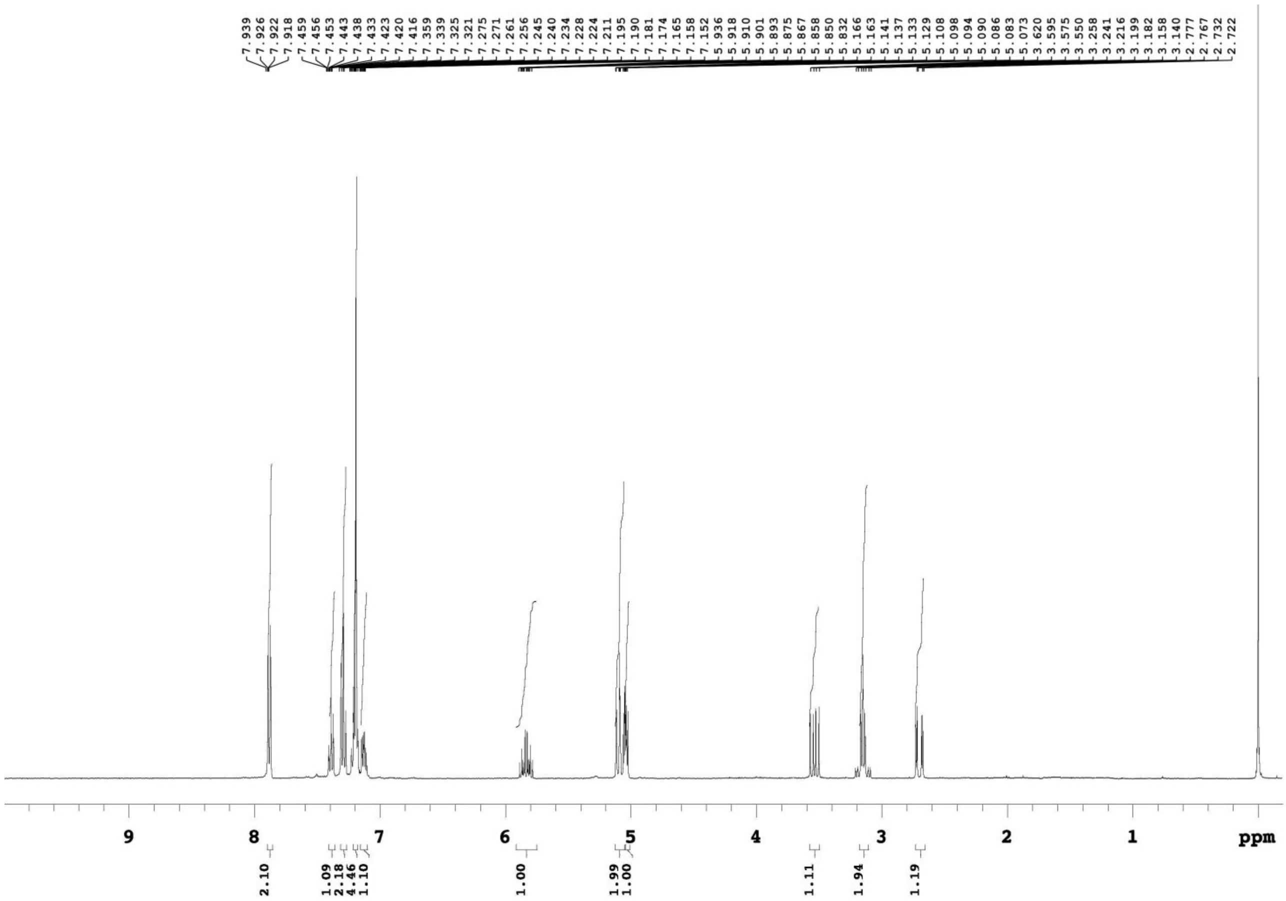

7.939
7.926
7.922
7.918
7.459
7.456
7.453
7.443
7.438
7.433
7.433
7.423
7.420
7.416
7.359
7.339
7.325
7.321
7.275
7.271
7.261
7.256
7.245
7.240
7.234
7.228
7.224
7.211
7.195
7.190
7.181
7.174
7.165
7.158
7.152
5.936
5.918
5.910
5.901
5.893
5.875
5.867
5.858
5.850
5.832
5.166
5.163
5.141
5.137
5.133
5.129
5.108
5.098
5.094
5.090
5.086
5.083
5.073
3.620
3.595
3.575
3.550
3.258
3.241
3.216
3.199
3.182
3.158
3.140
2.777
2.767
2.732
2.722
9
8
7
6
5
4
3
2
1
ppm
2.10
1.09
2.18
4.46
1.10
1.00
1.99
1.00
1.11
1.94
1.19

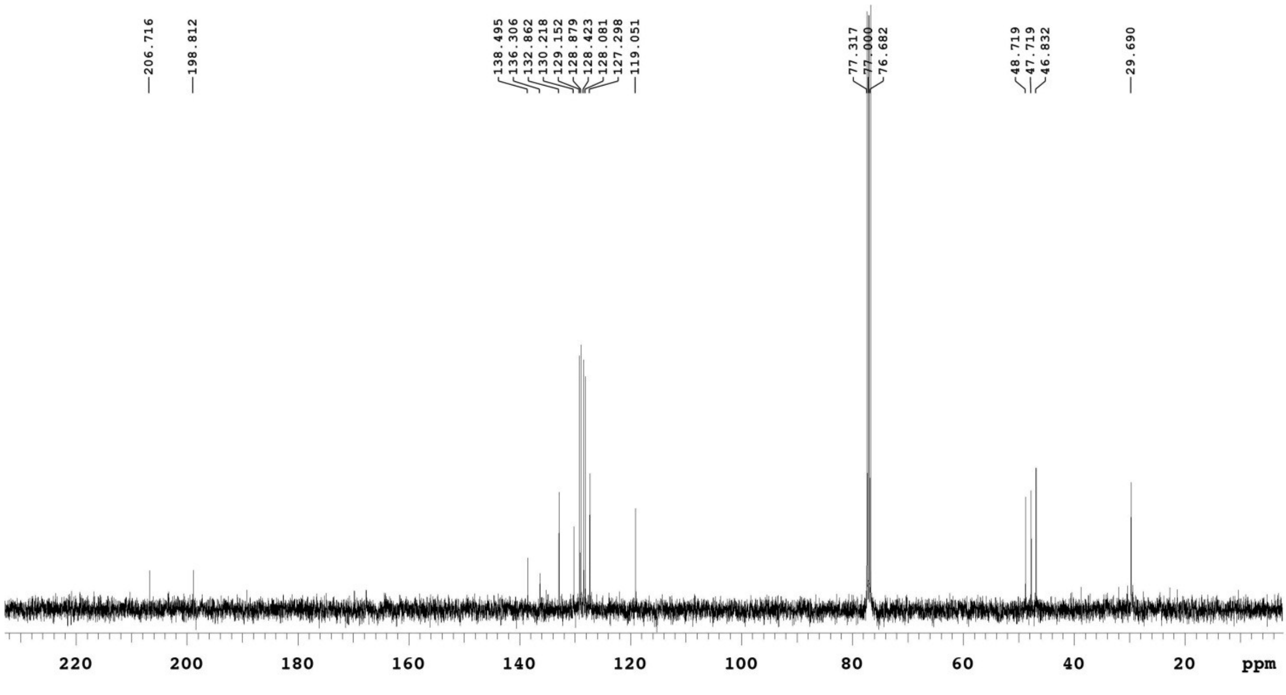

206.716
198.812
138.495
136.306
132.862
130.218
129.152
128.879
128.423
128.081
127.298
119.051
77.317
77.000
76.682
48.719
47.719
46.832
29.690
220
200
180
160
140
120
100
80
60
40
20
ppm

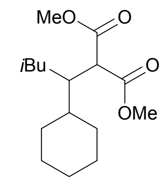
MeO
O
iBu
O
OMe

14

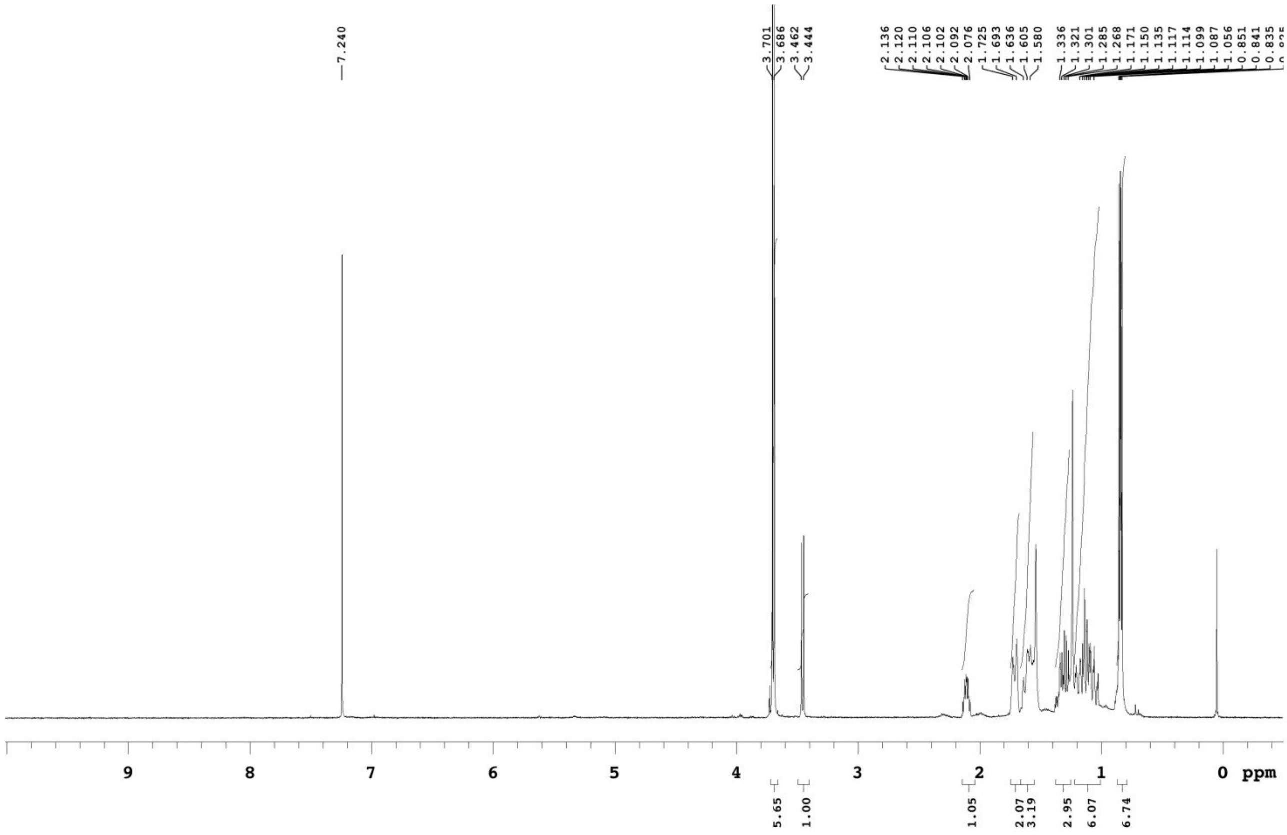
7.240
3.701
3.686
3.462
3.444
2.136
2.120
2.110
2.106
2.102
2.092
2.076
1.725
1.693
1.636
1.605
1.580
1.336
1.321
1.301
1.285
1.268
1.171
1.150
1.135
1.117
1.114
1.099
1.087
1.056
0.851
0.841
0.835
9
8
7
6
5
4
3
2
1
0 ppm
5.65
1.00
1.05
2.07
3.19
2.95
6.07
6.74

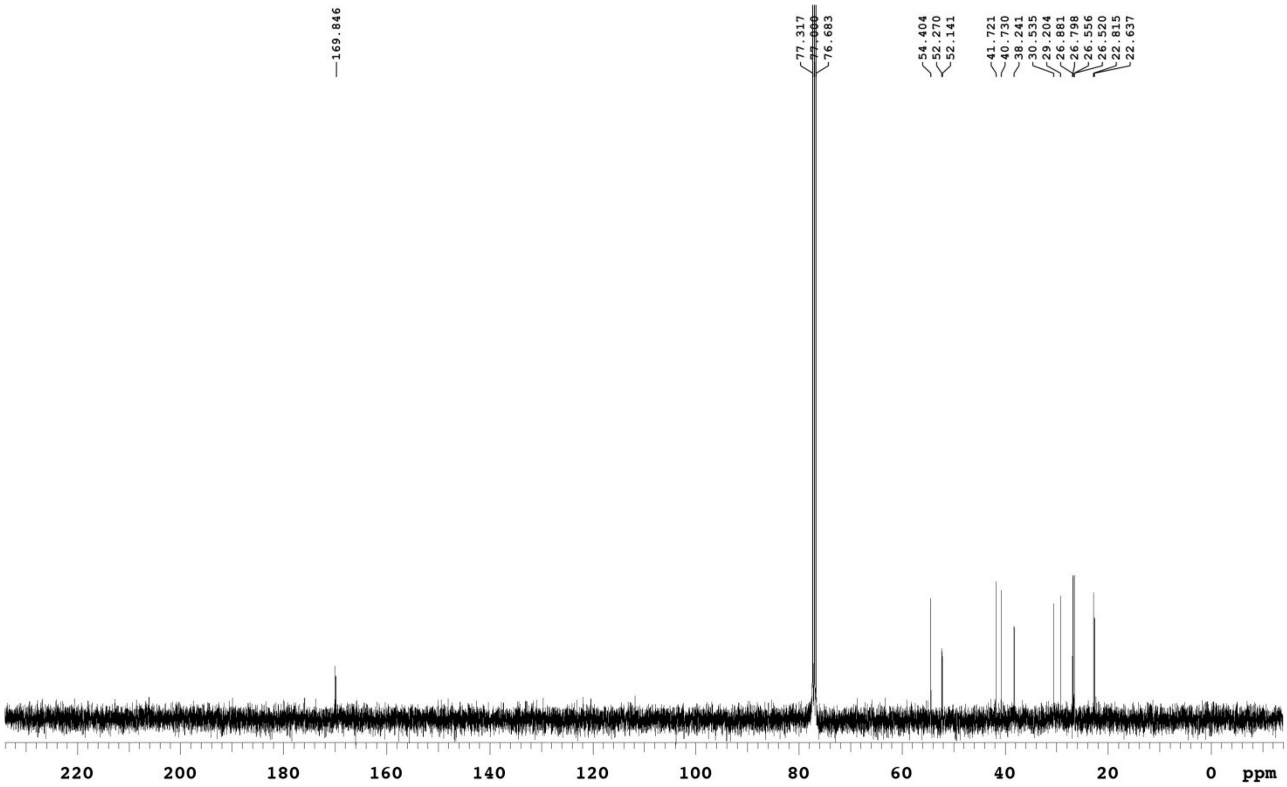
169.846
77.317
77.000
76.683
54.404
52.270
52.141
41.721
40.730
38.241
30.535
29.204
26.881
26.798
26.556
26.520
22.815
22.637
220
200
180
160
140
120
100
80
60
40
20
0
ppm

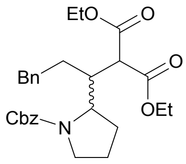
EtO
O
Bn
O
OEt
Cbz
N

16

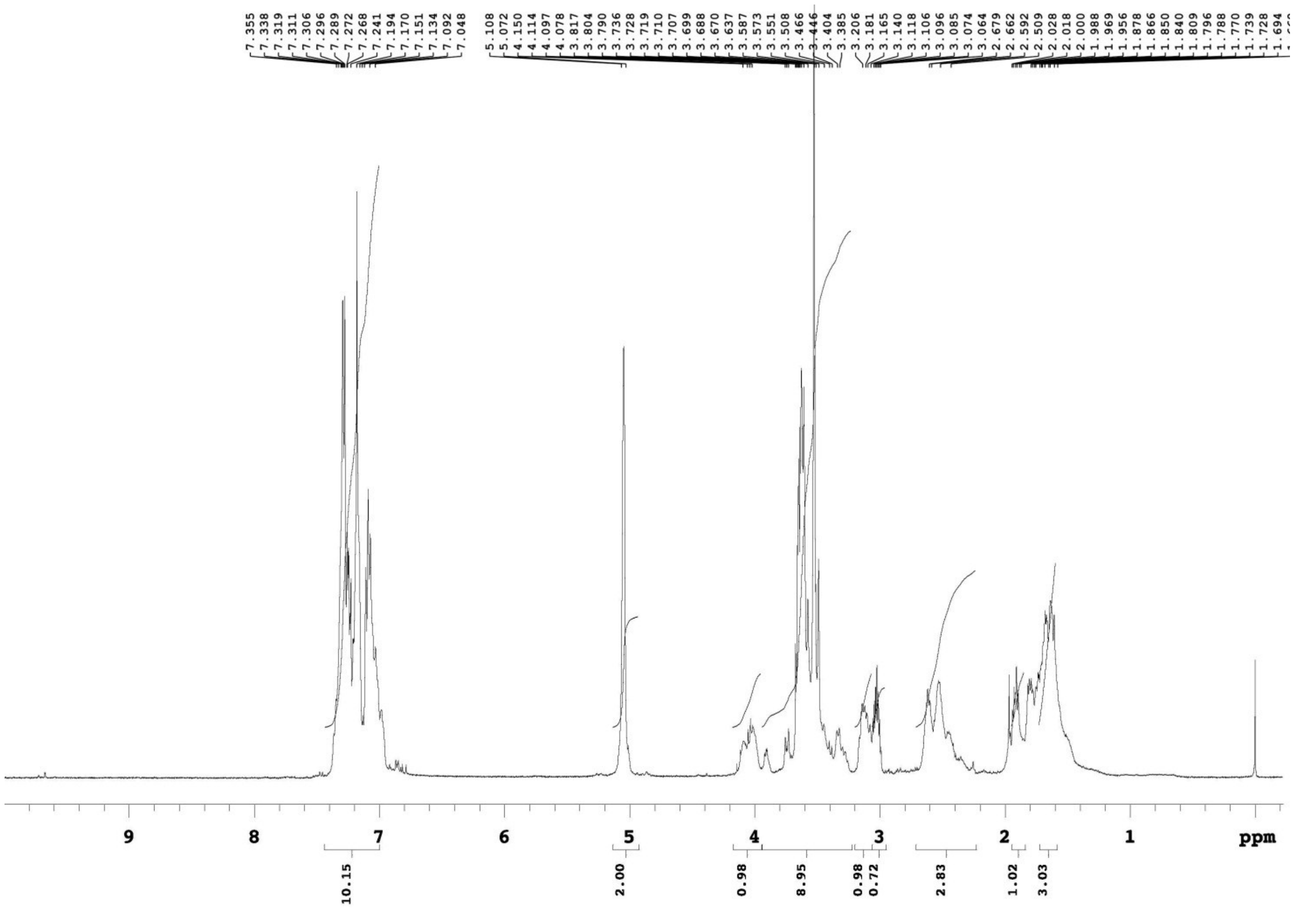
7.355
7.338
7.319
7.311
7.306
7.296
7.289
7.272
7.268
7.241
7.194
7.170
7.151
7.134
7.092
7.048
5.108
5.072
4.150
4.114
4.097
4.078
3.817
3.804
3.790
3.736
3.728
3.719
3.710
3.707
3.699
3.688
3.670
3.637
3.587
3.573
3.551
3.508
3.466
3.446
3.404
3.385
3.206
3.181
3.165
3.140
3.118
3.106
3.096
3.085
3.074
3.064
2.679
2.662
2.592
2.509
2.028
2.018
2.000
1.988
1.969
1.956
1.878
1.866
1.850
1.840
1.809
1.796
1.788
1.770
1.739
1.728
1.694
9
8
7
6
5
4
3
2
1
ppm
10.15
2.00
0.98
8.95
0.98
0.72
2.83
1.02
3.03

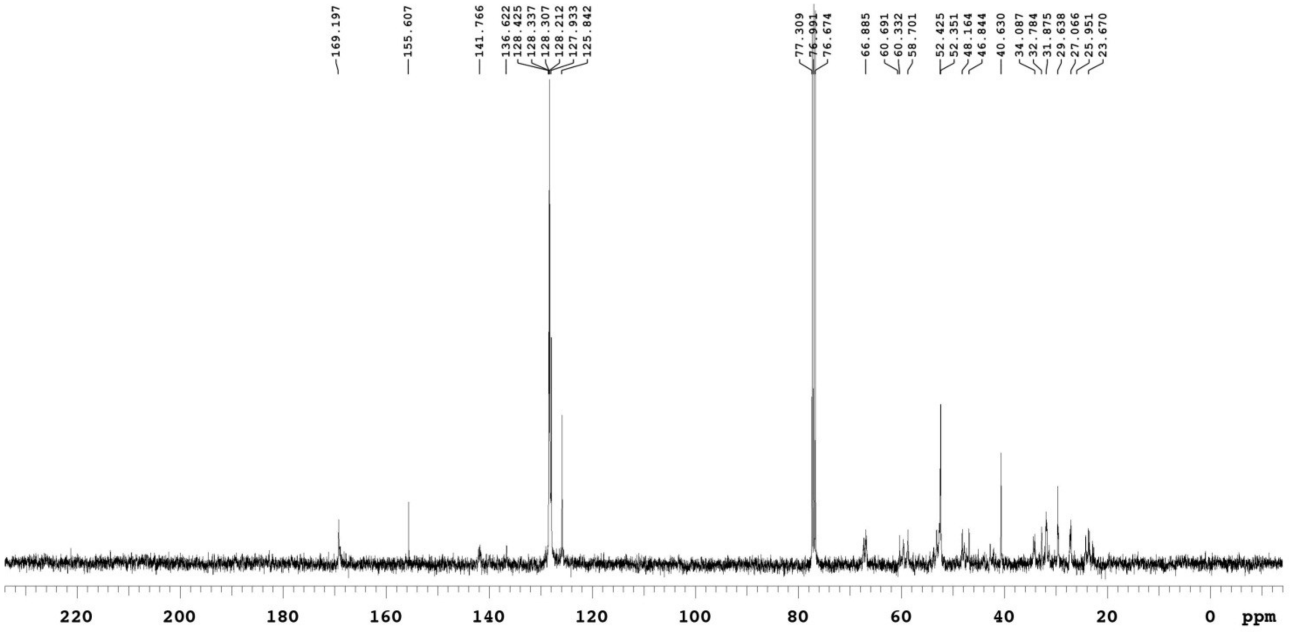
169.197
155.607
141.766
136.622
128.425
128.337
128.307
128.212
127.933
125.842
77.309
76.991
76.674
66.885
60.691
60.332
58.701
52.425
52.351
48.164
46.844
40.630
34.087
32.784
31.875
29.638
27.066
25.951
23.670
220
200
180
160
140
120
100
80
60
40
20
0
ppm

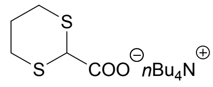

S
S
COO⊖nBu4N⊕

17

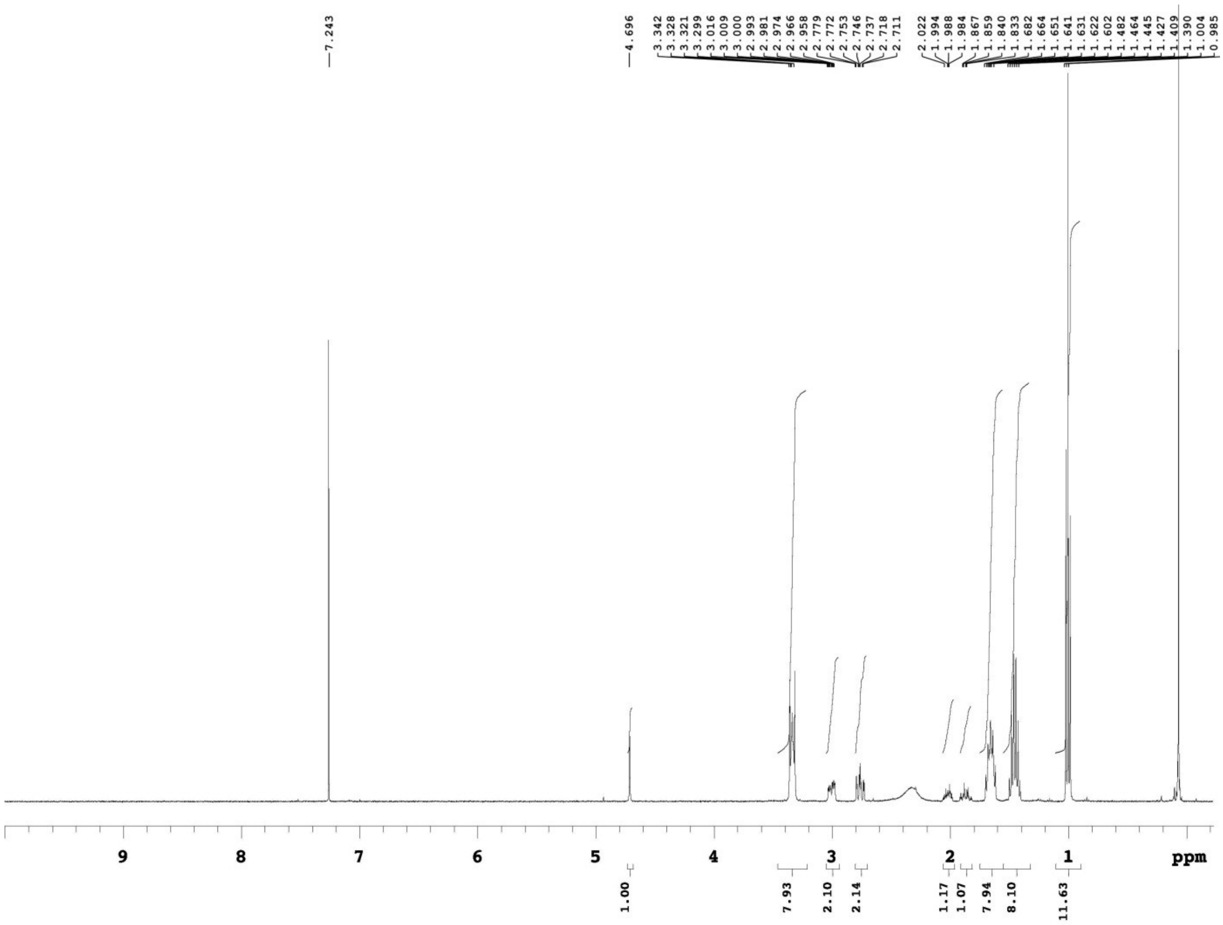

7.243
4.696
3.342 3.328 3.321 3.299 3.016 3.009 3.000 2.993 2.981 2.974 2.966 2.958 2.779 2.772 2.753 2.746 2.737 2.718 2.711
2.022 1.994 1.988 1.984 1.867 1.859 1.840 1.833 1.682 1.664 1.651 1.641 1.631 1.622 1.602 1.482 1.464 1.445 1.427 1.409 1.390 1.004 0.985
9 8 7 6 5 4 3 2 1 ppm
1.00
7.93
2.10
2.14
1.17
1.07
7.94
8.10
11.63

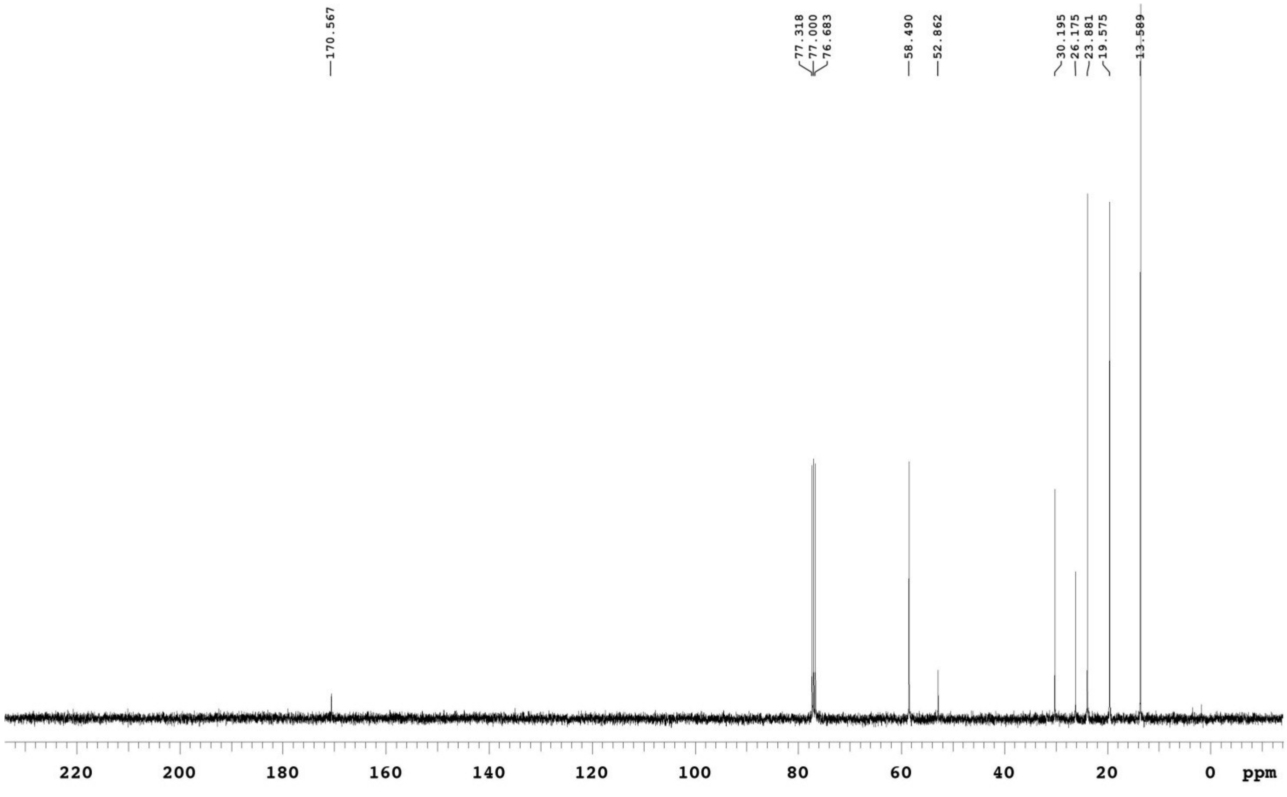

170.567
77.318 77.000 76.683
58.490
52.862
30.195 26.175 23.881 19.575
13.589
220 200 180 160 140 120 100 80 60 40 20 0 ppm

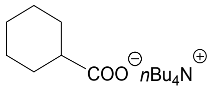
COO⊖ nBu4N⊕
18

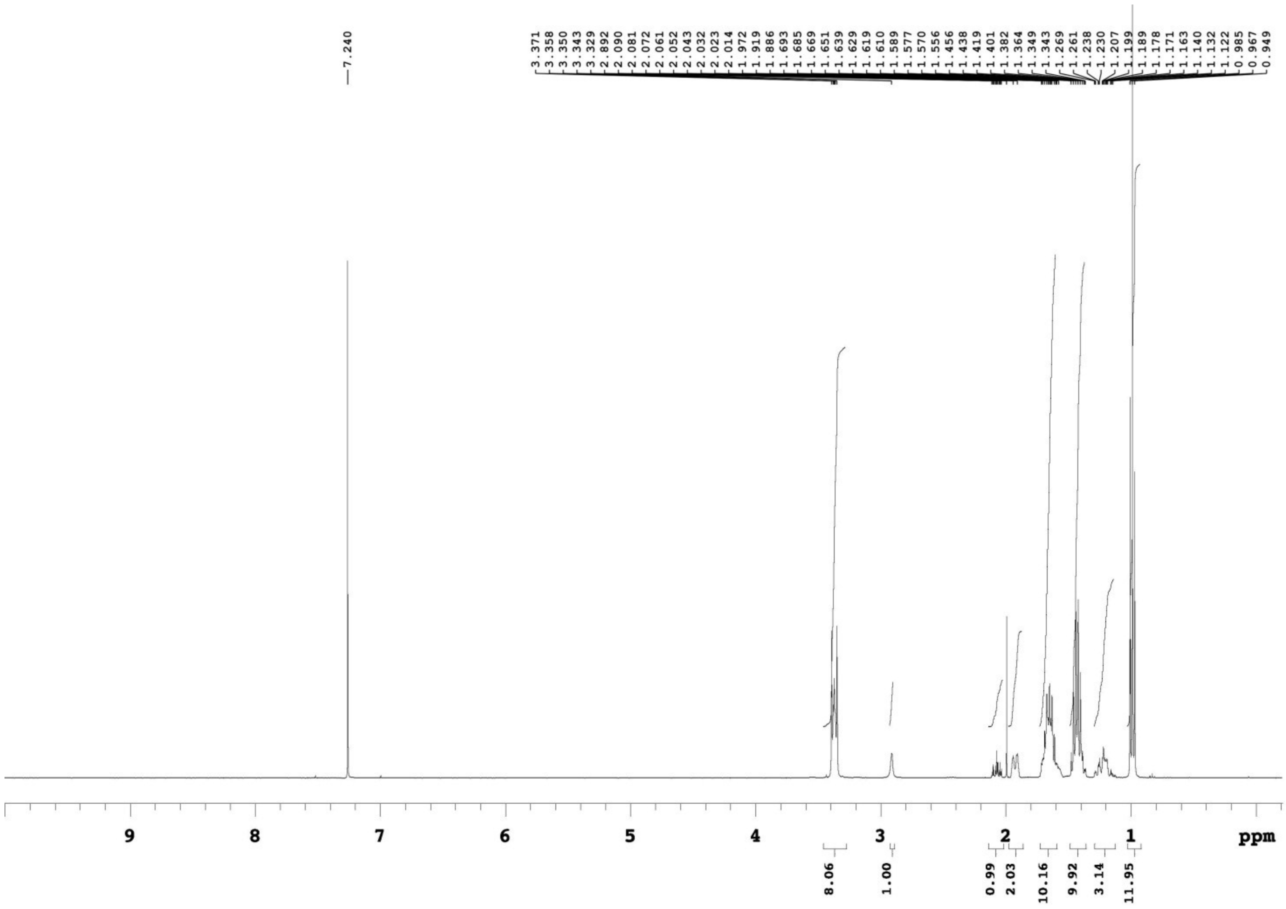
7.240
3.371 3.358 3.350 3.343 3.329 2.892 2.090 2.081 2.072 2.061 2.052 2.043 2.032 2.023 2.014 1.972 1.919 1.886 1.693 1.685 1.669 1.651 1.639 1.629 1.619 1.610 1.589 1.577 1.570 1.556 1.456 1.438 1.419 1.401 1.382 1.364 1.349 1.343 1.269 1.261 1.238 1.230 1.207 1.199 1.189 1.178 1.171 1.163 1.140 1.132 1.122 0.985 0.967 0.949
9 8 7 6 5 4 3 2 1 ppm
8.06 1.00 0.99 2.03 10.16 9.92 3.14 11.95

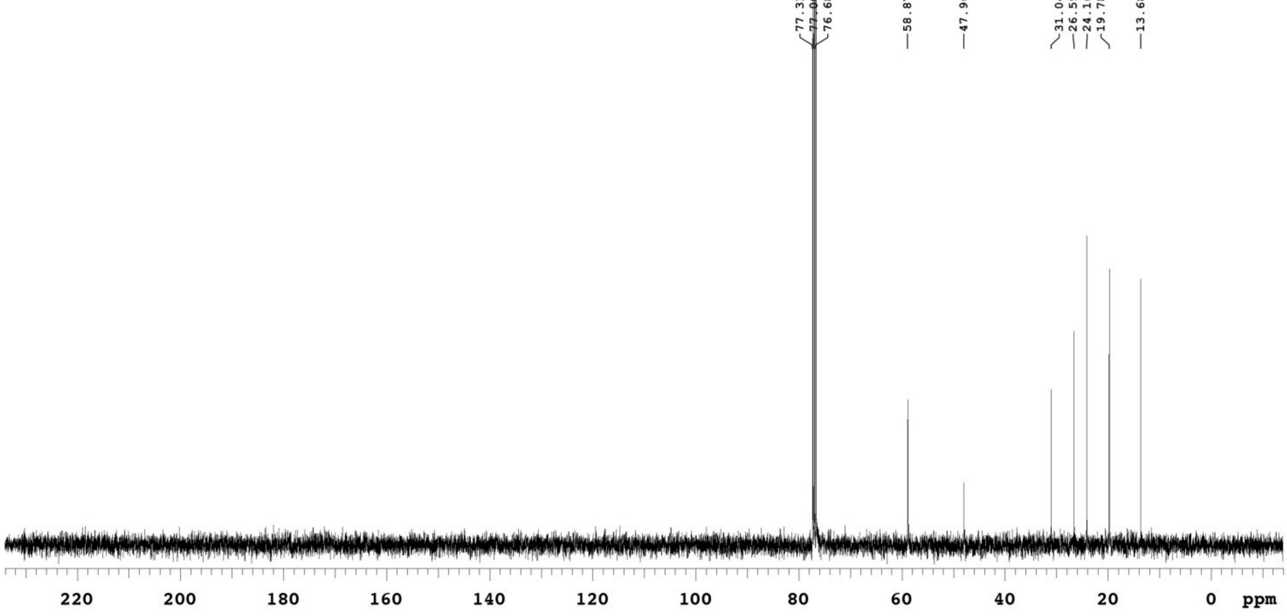
77.317 77.000 76.683 58.871 47.966 31.048 26.597 24.167 19.784 13.685
220 200 180 160 140 120 100 80 60 40 20 0 ppm

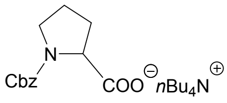
Cbz
N
COO⊖ nBu4N⊕

19

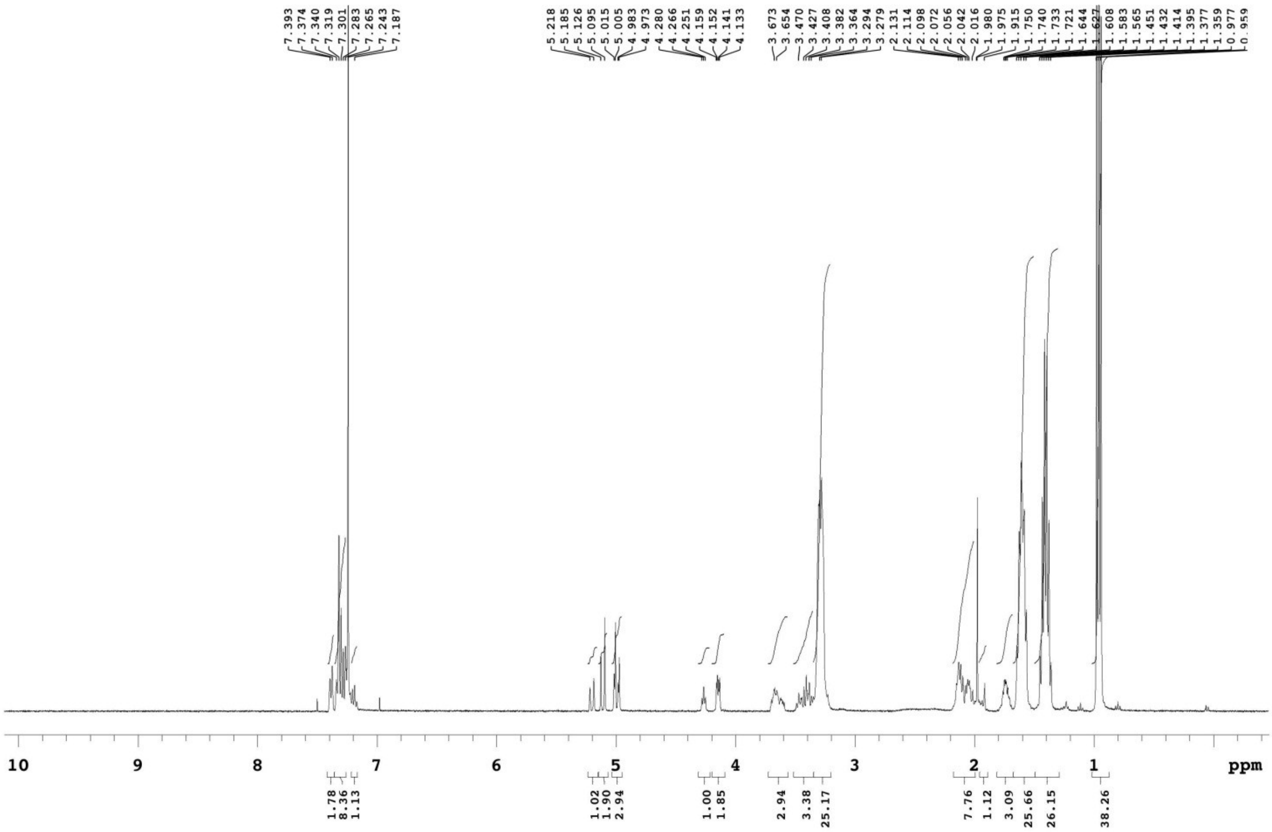
7.393
7.374
7.340
7.319
7.301
7.283
7.265
7.243
7.187
5.218
5.185
5.126
5.095
5.015
5.005
4.983
4.973
4.280
4.266
4.251
4.159
4.152
4.141
4.133
3.673
3.654
3.470
3.427
3.408
3.382
3.364
3.294
3.279
2.131
2.114
2.098
2.072
2.056
2.042
2.016
1.980
1.975
1.915
1.750
1.740
1.733
1.721
1.644
1.627
1.608
1.583
1.565
1.451
1.432
1.414
1.395
1.377
1.359
0.977
0.959
10
9
8
7
6
5
4
3
2
1
ppm
1.78
8.36
1.13
1.02
1.90
2.94
1.00
1.85
2.94
3.38
25.17
7.76
1.12
3.09
25.66
26.15
38.26

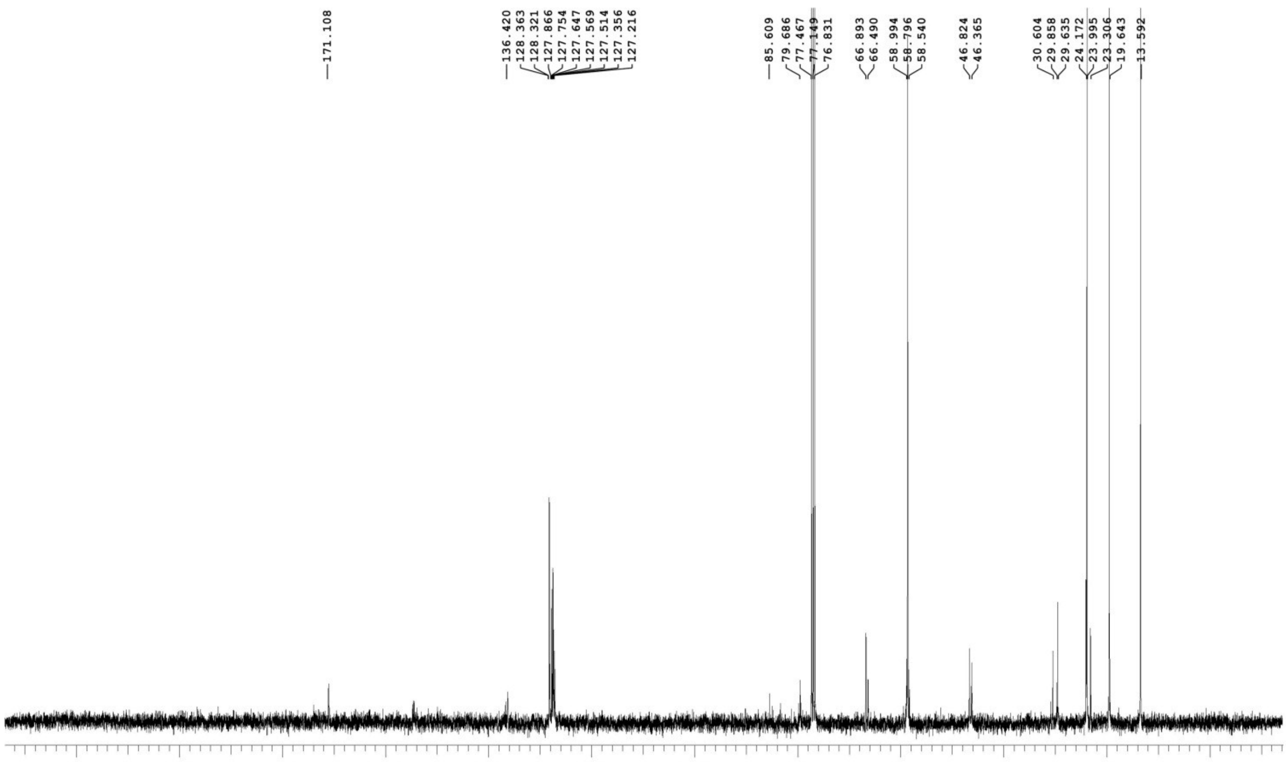
171.108
136.420
128.363
128.321
127.866
127.754
127.647
127.569
127.514
127.356
127.216
85.609
79.686
77.467
77.149
76.831
66.893
66.490
58.994
58.796
58.540
46.824
46.365
30.604
29.858
29.635
24.172
23.995
23.306
19.643
13.592
220
200
180
160
140
120
100
80
60
40
20
0
ppm

Copies of IR spectra (all spectra were recorded neat)

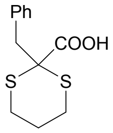

4b

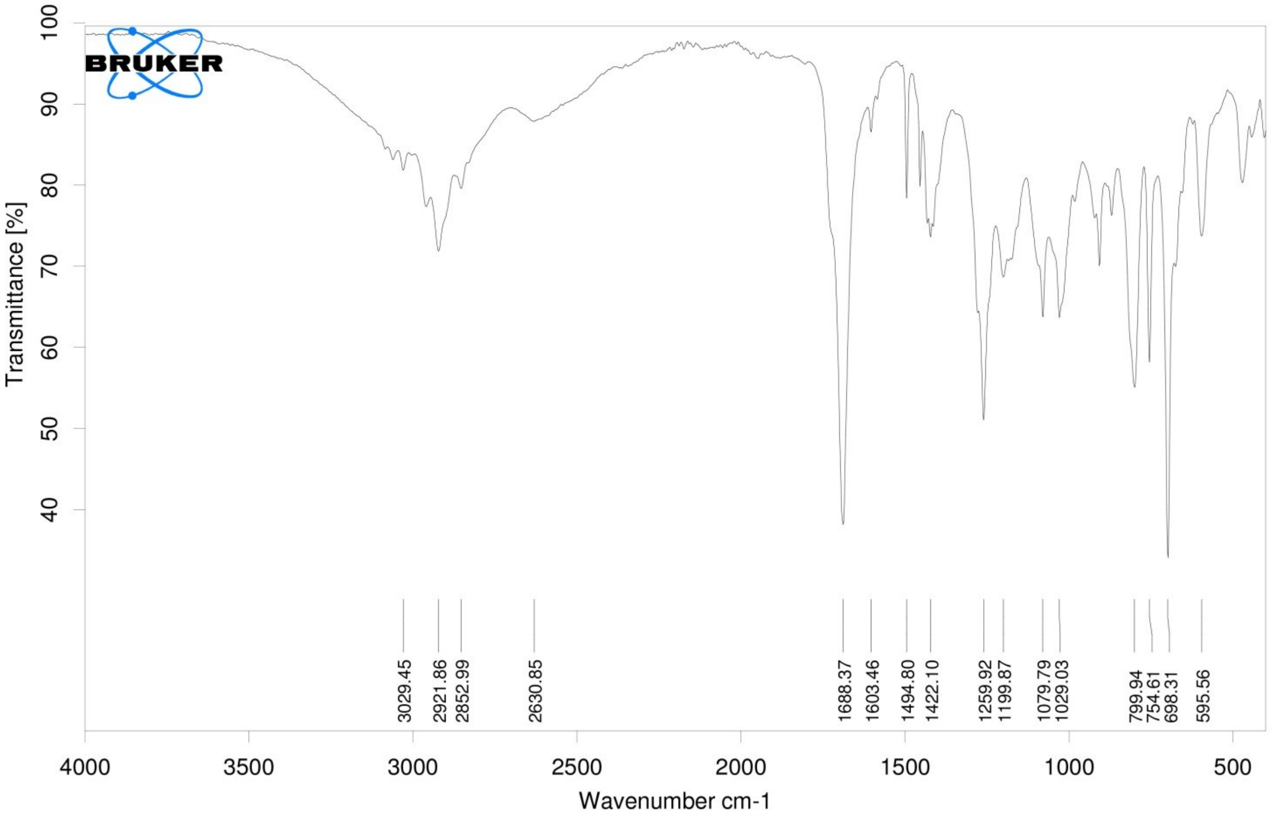

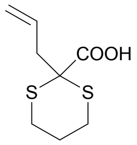
COOH
S
S

4c

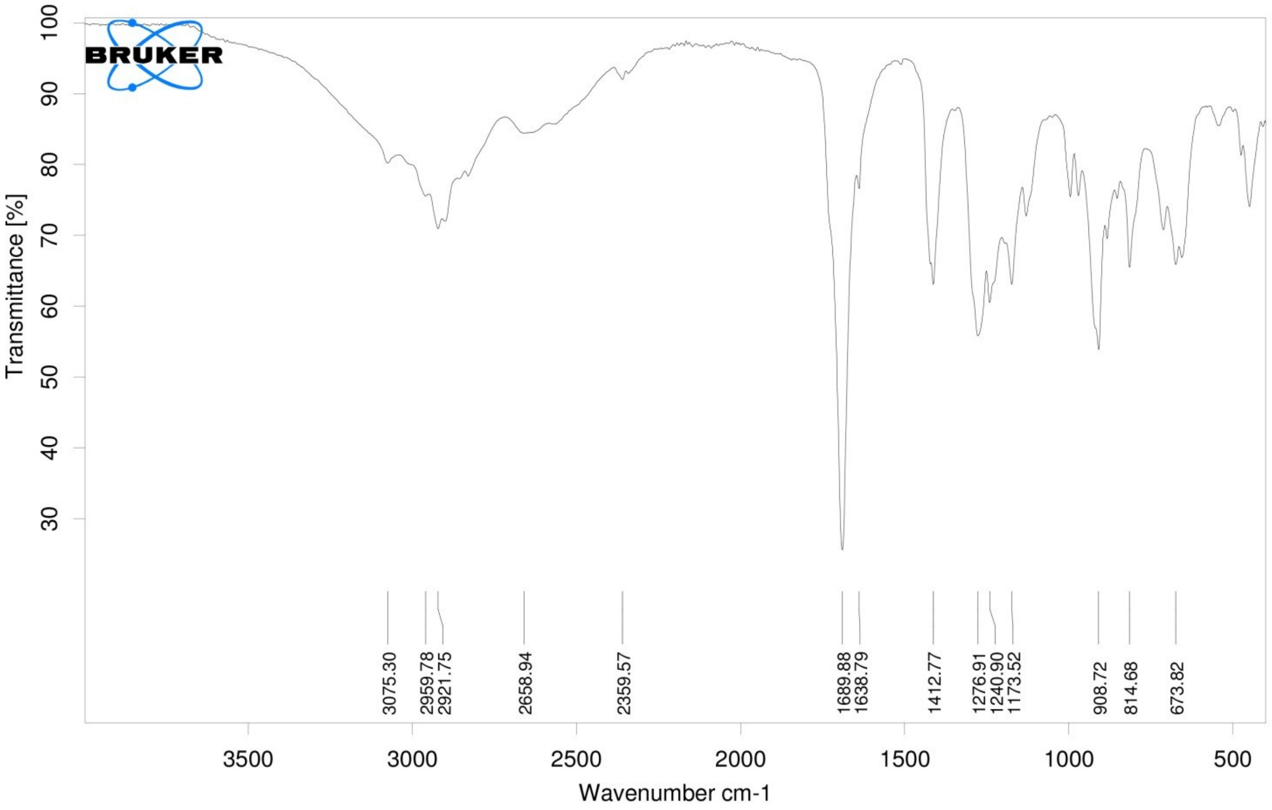
BRUKER
Transmittance [%]
Wavenumber cm-1
3075.30
2959.78
2921.75
2658.94
2359.57
1689.88
1638.79
1412.77
1276.91
1240.90
1173.52
908.72
814.68
673.82

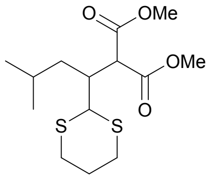

5a

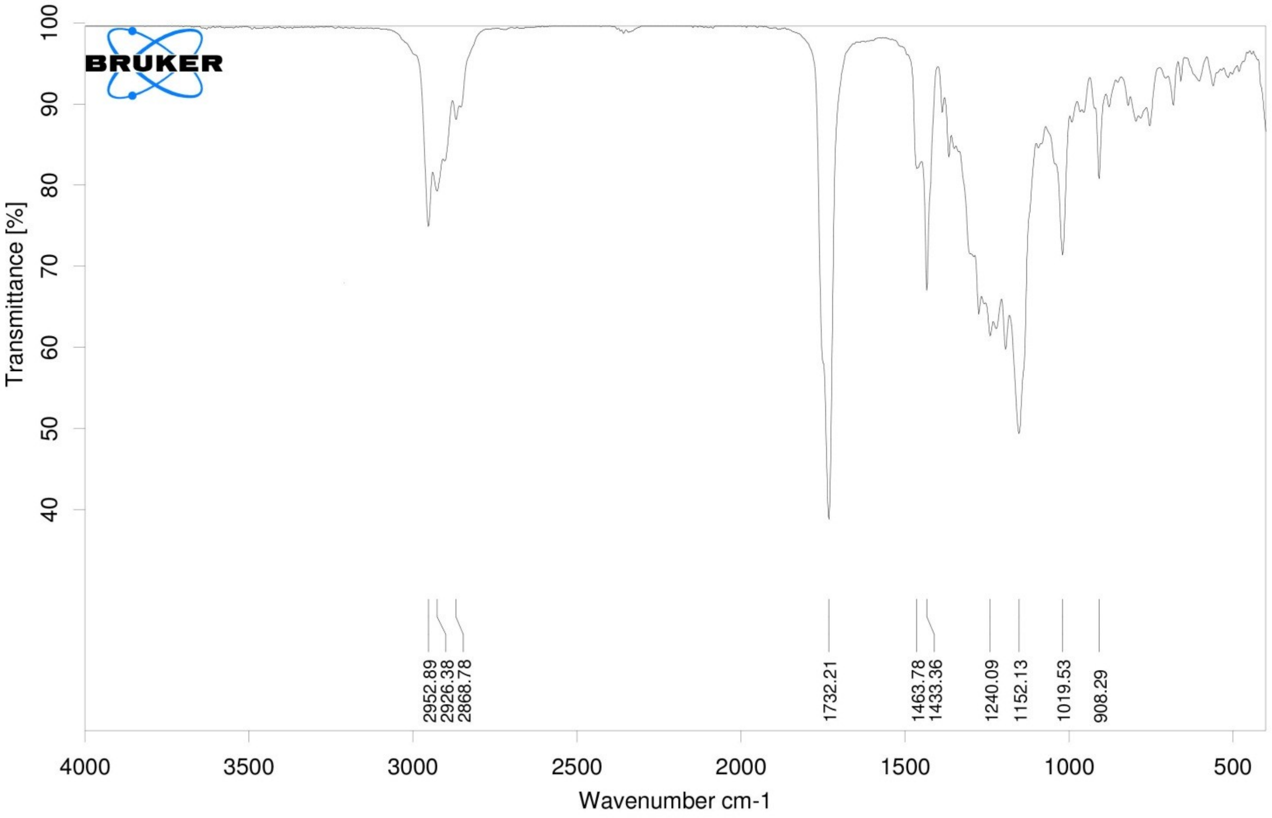

BRUKER
Transmittance [%]
Wavenumber cm-1
2952.89
2926.38
2868.78
1732.21
1463.78
1433.36
1240.09
1152.13
1019.53
908.29

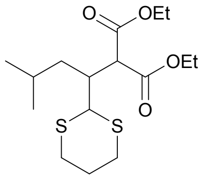
O
OEt
OEt
O
S
S

5b

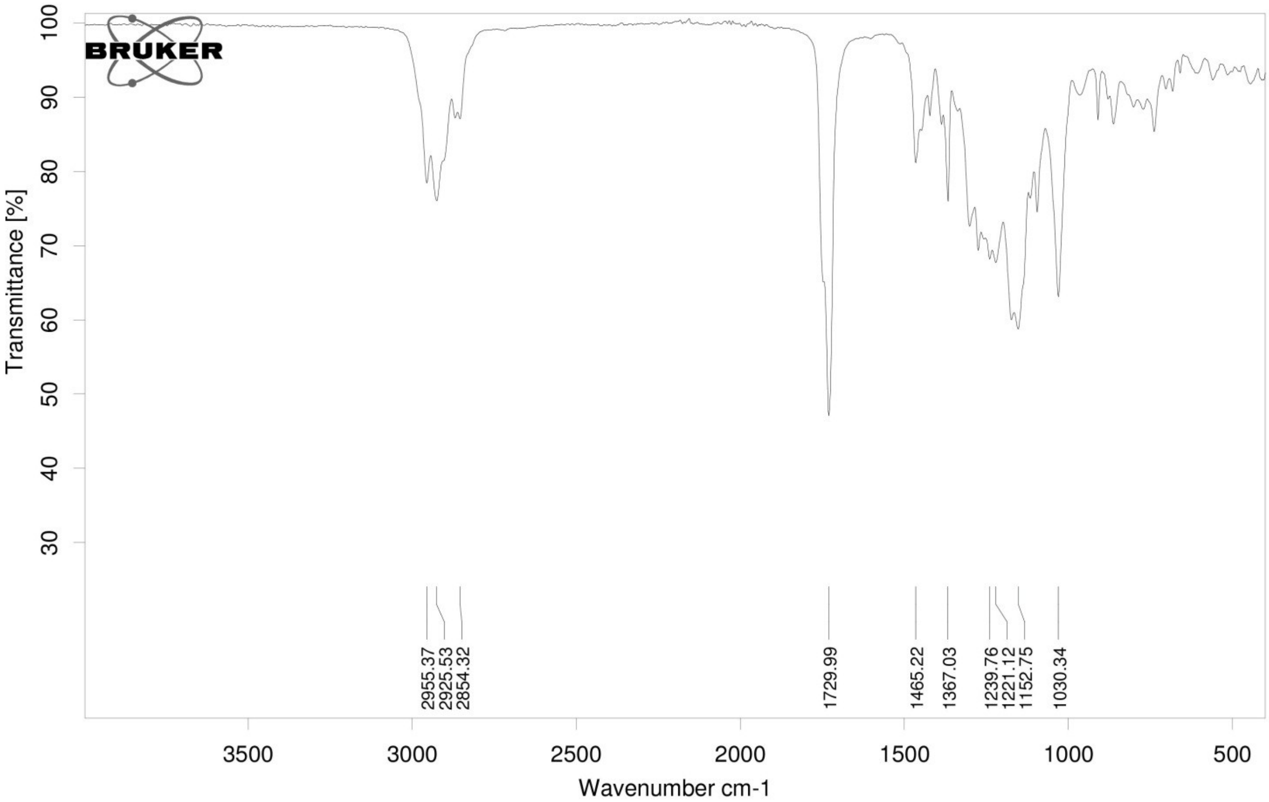
BRUKER
Transmittance [%]
100
90
80
70
60
50
40
30
2955.37
2925.53
2854.32
1729.99
1465.22
1367.03
1239.76
1221.12
1152.75
1030.34
3500
3000
2500
2000
1500
1000
500
Wavenumber cm-1

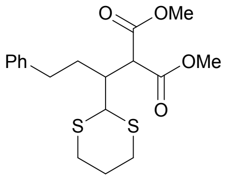
OMe
O
Ph
OMe
O
S
S

5c

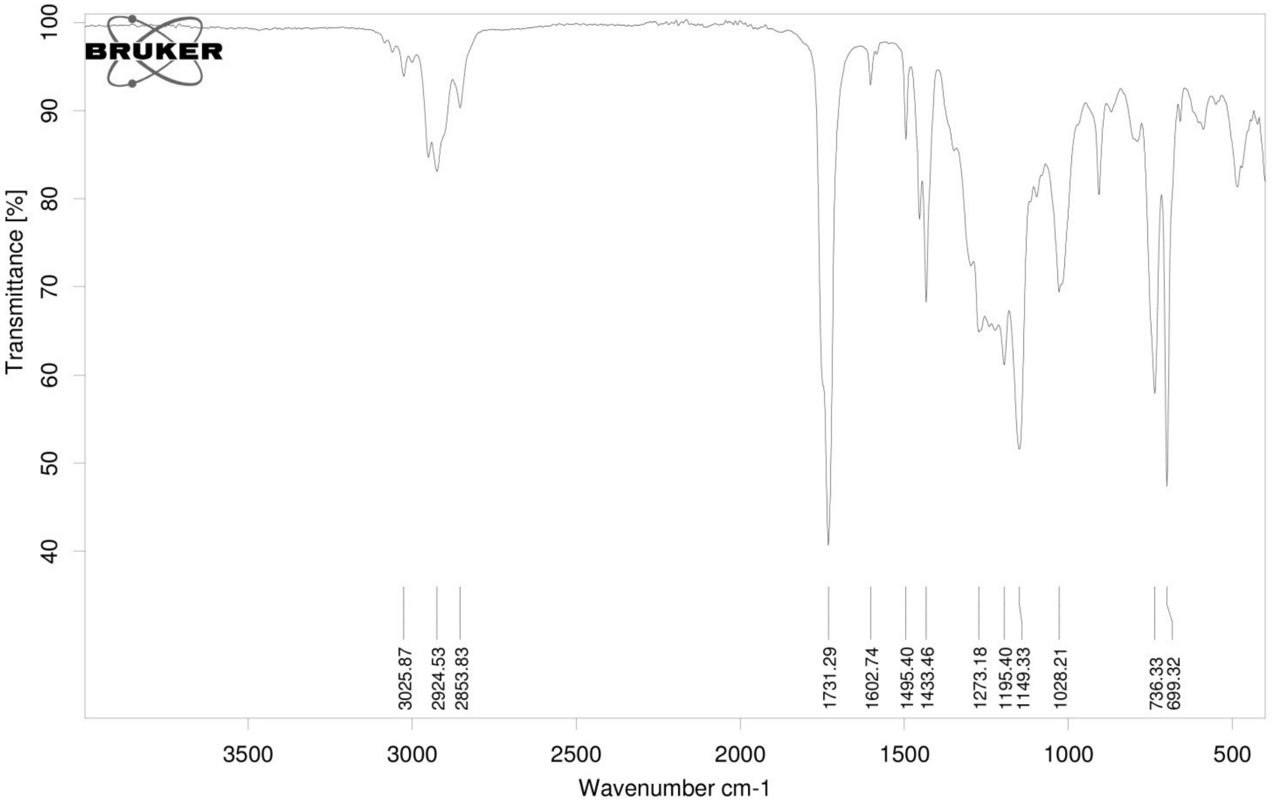
BRUKER
Transmittance [%]
100
90
80
70
60
50
40
3025.87
2924.53
2853.83
1731.29
1602.74
1495.40
1433.46
1273.18
1195.40
1149.33
1028.21
736.33
699.32
3500
3000
2500
2000
1500
1000
500
Wavenumber cm-1

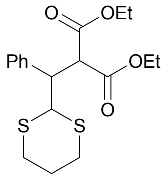

5d

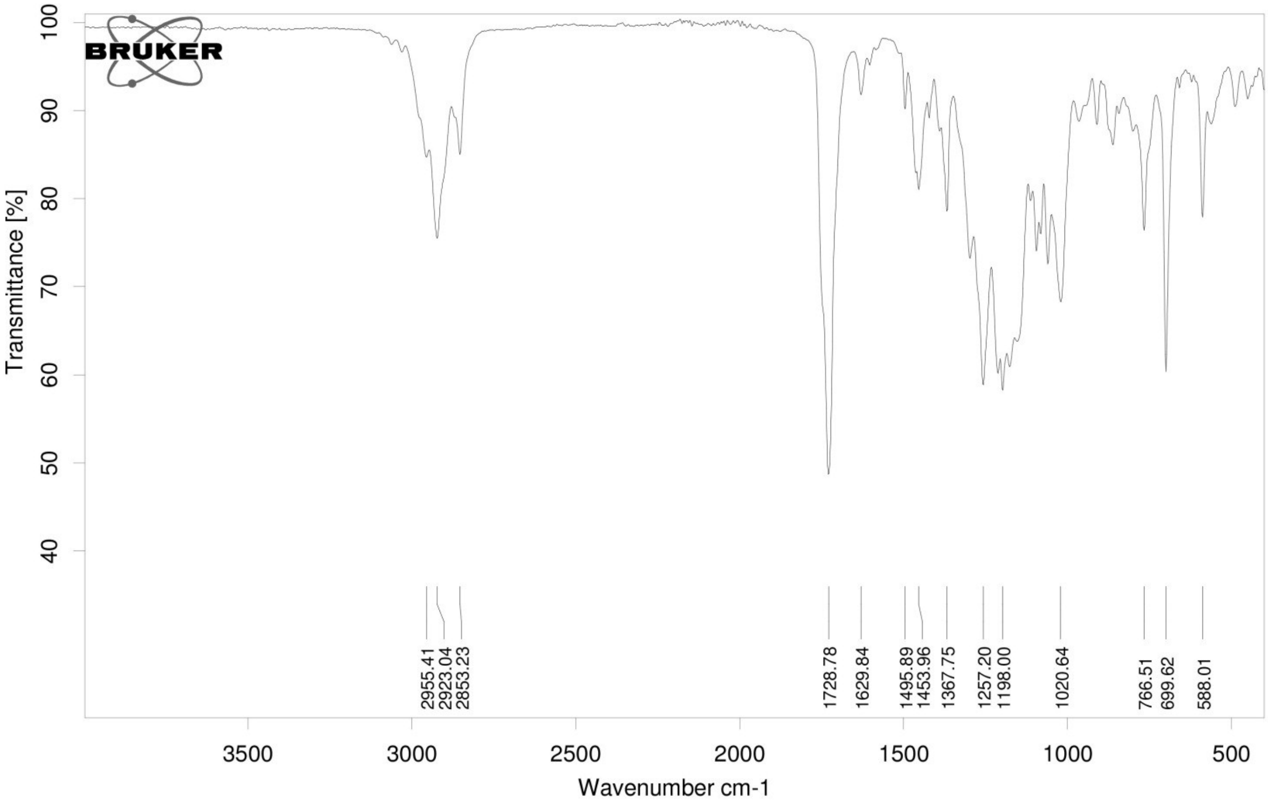
BRUKER
Transmittance [%]
100
90
80
70
60
50
40
2955.41
2923.04
2853.23
1728.78
1629.84
1495.89
1453.96
1367.75
1257.20
1198.00
1020.64
766.51
699.62
588.01
3500
3000
2500
2000
1500
1000
500
Wavenumber cm-1

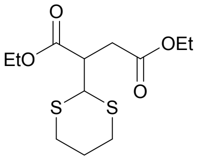

5e

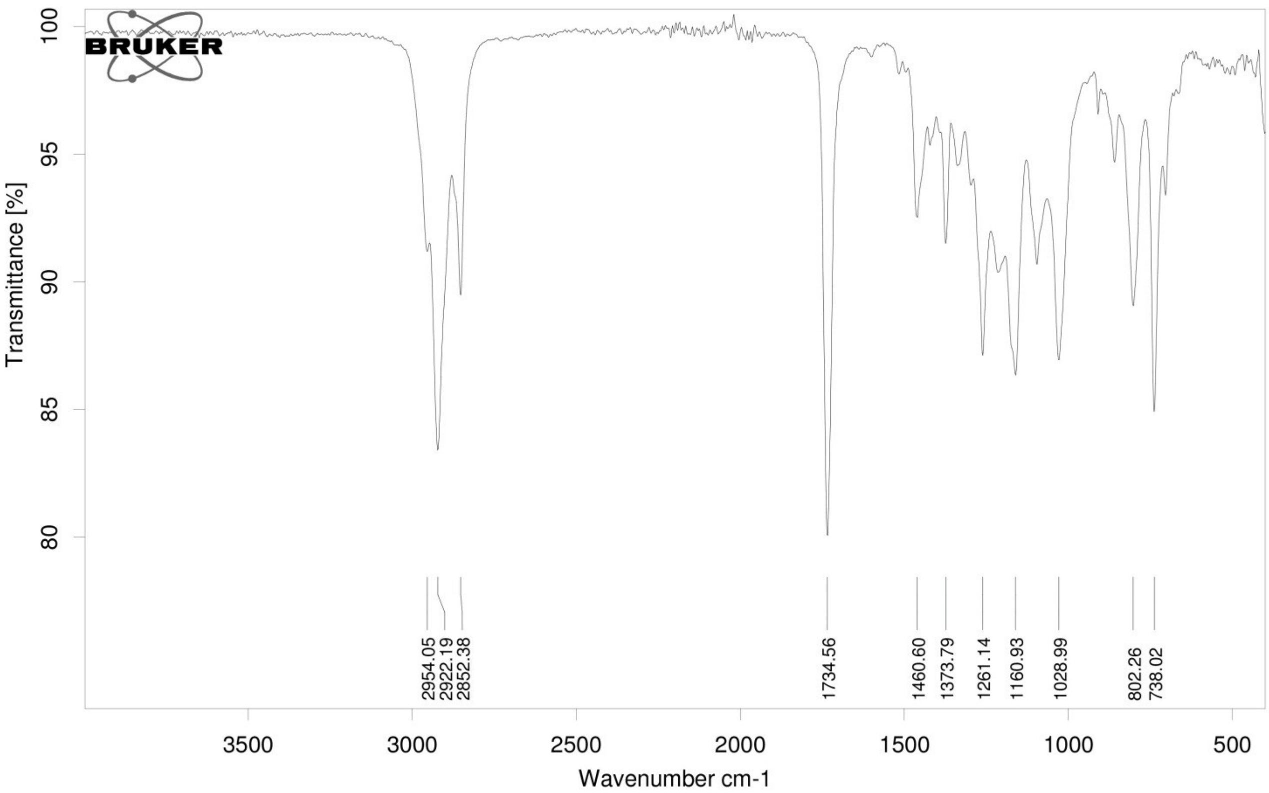

BRUKER
Transmittance [%]
100
95
90
85
80
2954.05
2922.19
2852.38
1734.56
1460.60
1373.79
1261.14
1160.93
1028.99
802.26
738.02
3500
3000
2500
2000
1500
1000
500
Wavenumber cm-1

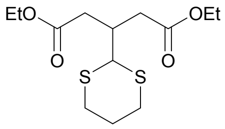

5f

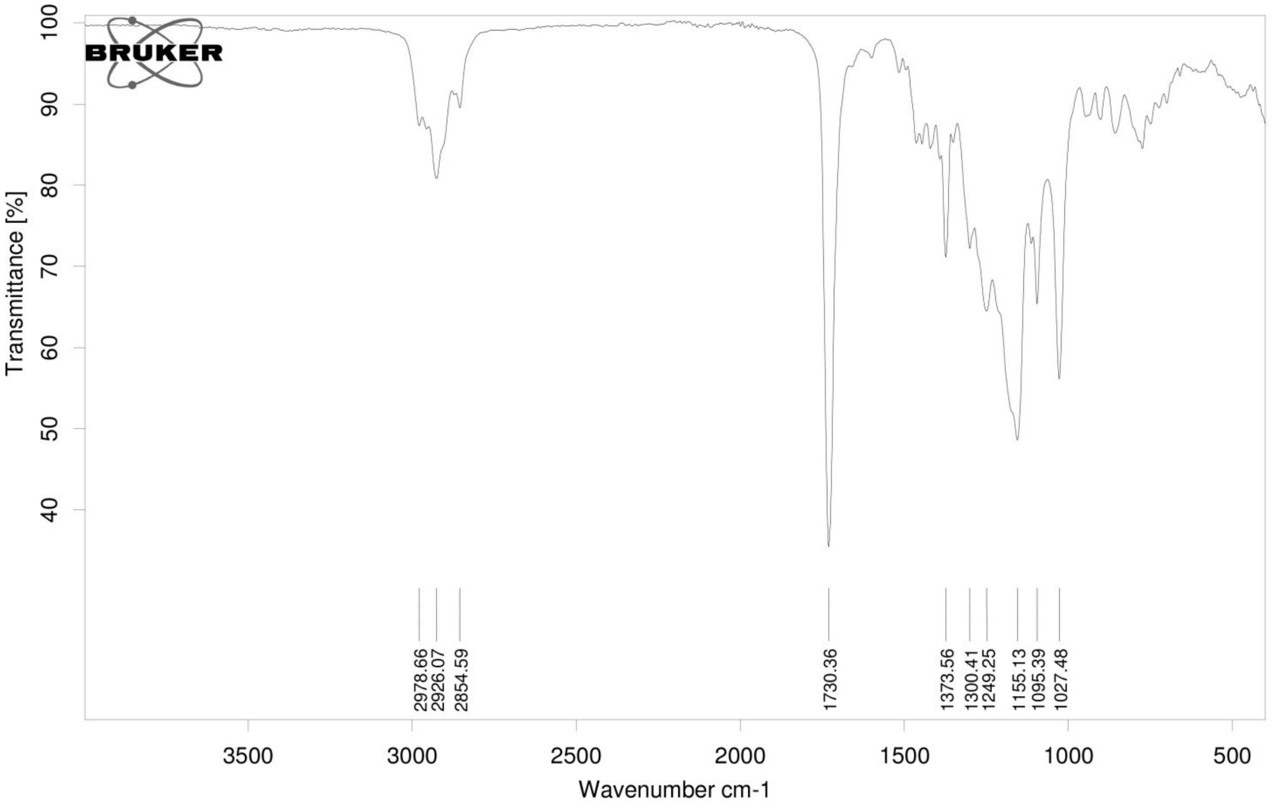

BRUKER
Transmittance [%]
100
90
80
70
60
50
40
2978.66
2926.07
2854.59
1730.36
1373.56
1300.41
1249.25
1155.13
1095.39
1027.48
3500
3000
2500
2000
1500
1000
500
Wavenumber cm-1

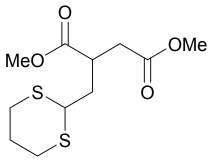

5g

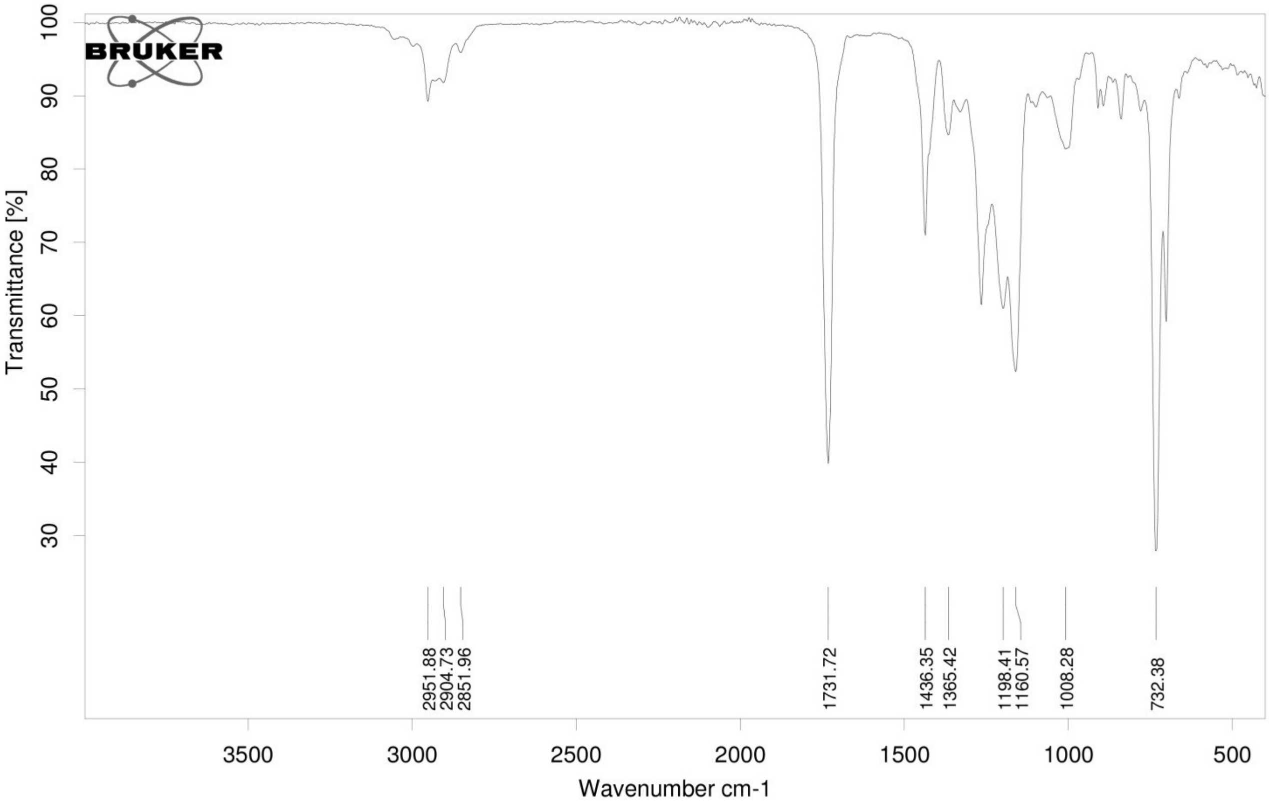
BRUKER
Transmittance [%]
100
90
80
70
60
50
40
30
2951.88
2904.73
2851.96
1731.72
1436.35
1365.42
1198.41
1160.57
1008.28
732.38
3500
3000
2500
2000
1500
1000
500
Wavenumber cm-1

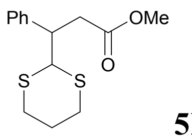
Ph
OMe
O
S
S

5h

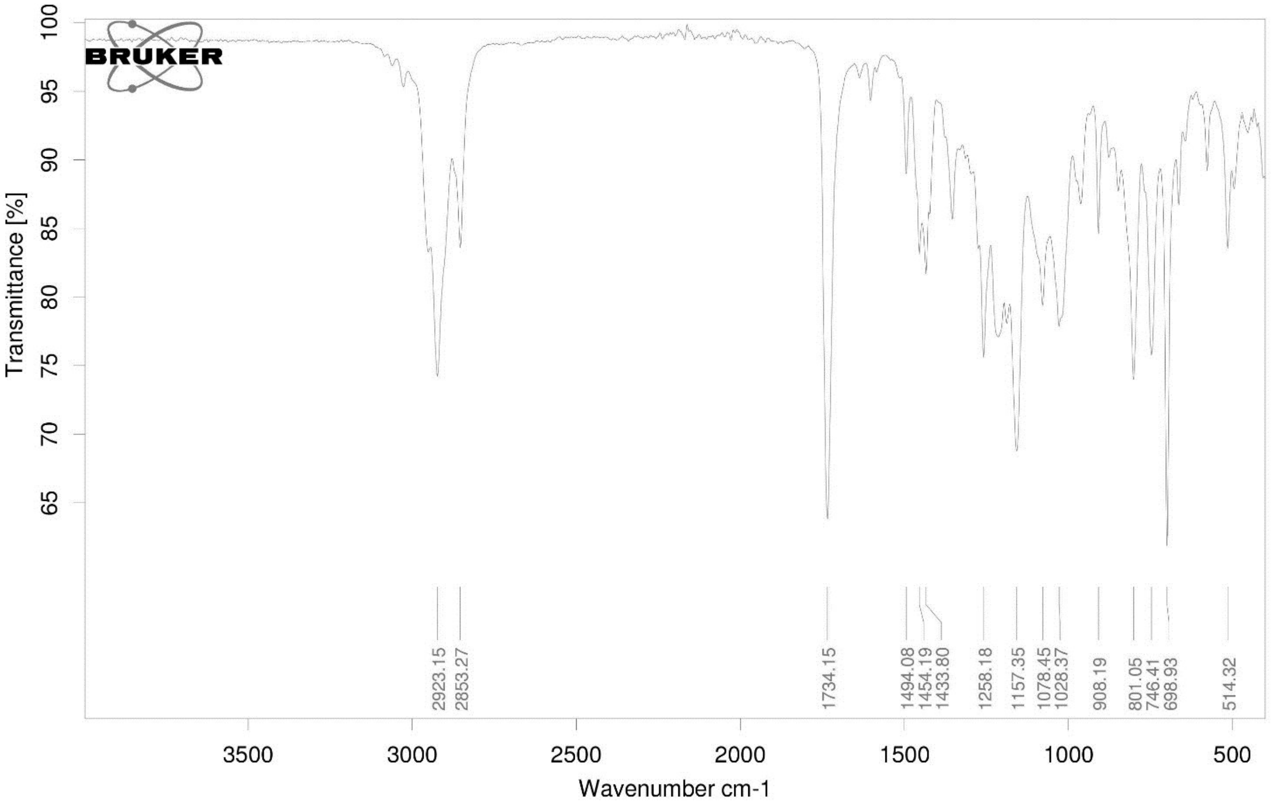
BRUKER
Transmittance [%]
Wavenumber cm-1
100
95
90
85
80
75
70
65
3500
3000
2500
2000
1500
1000
500
2923.15
2853.27
1734.15
1494.08
1454.19
1433.80
1258.18
1157.35
1078.45
1028.37
908.19
801.05
746.41
698.93
514.32

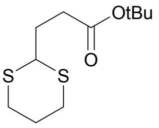
OtBu
O
S
S

5i

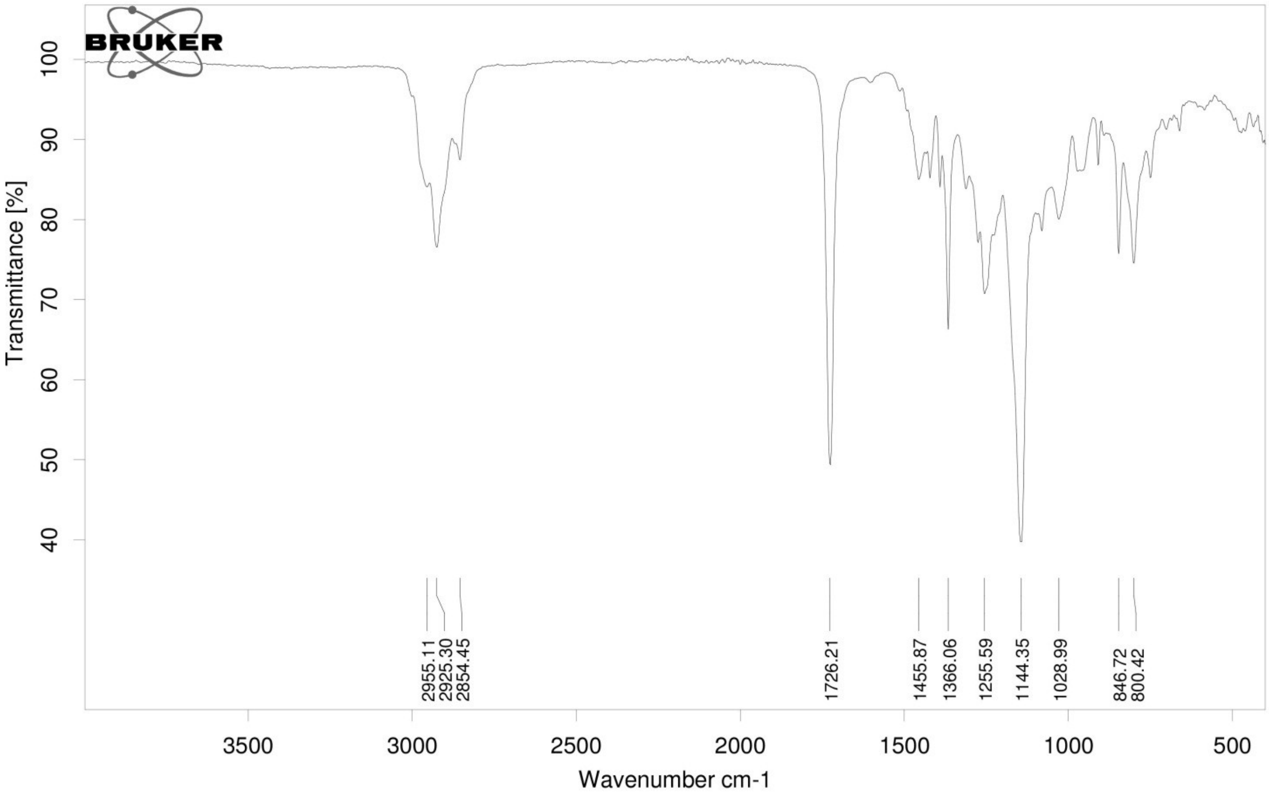
BRUKER
Transmittance [%]
100
90
80
70
60
50
40
2955.11
2925.30
2854.45
1726.21
1455.87
1366.06
1255.59
1144.35
1028.99
846.72
800.42
3500
3000
2500
2000
1500
1000
500
Wavenumber cm-1

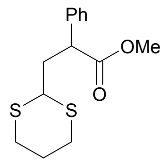
Ph
OMe
O
S
S

5j

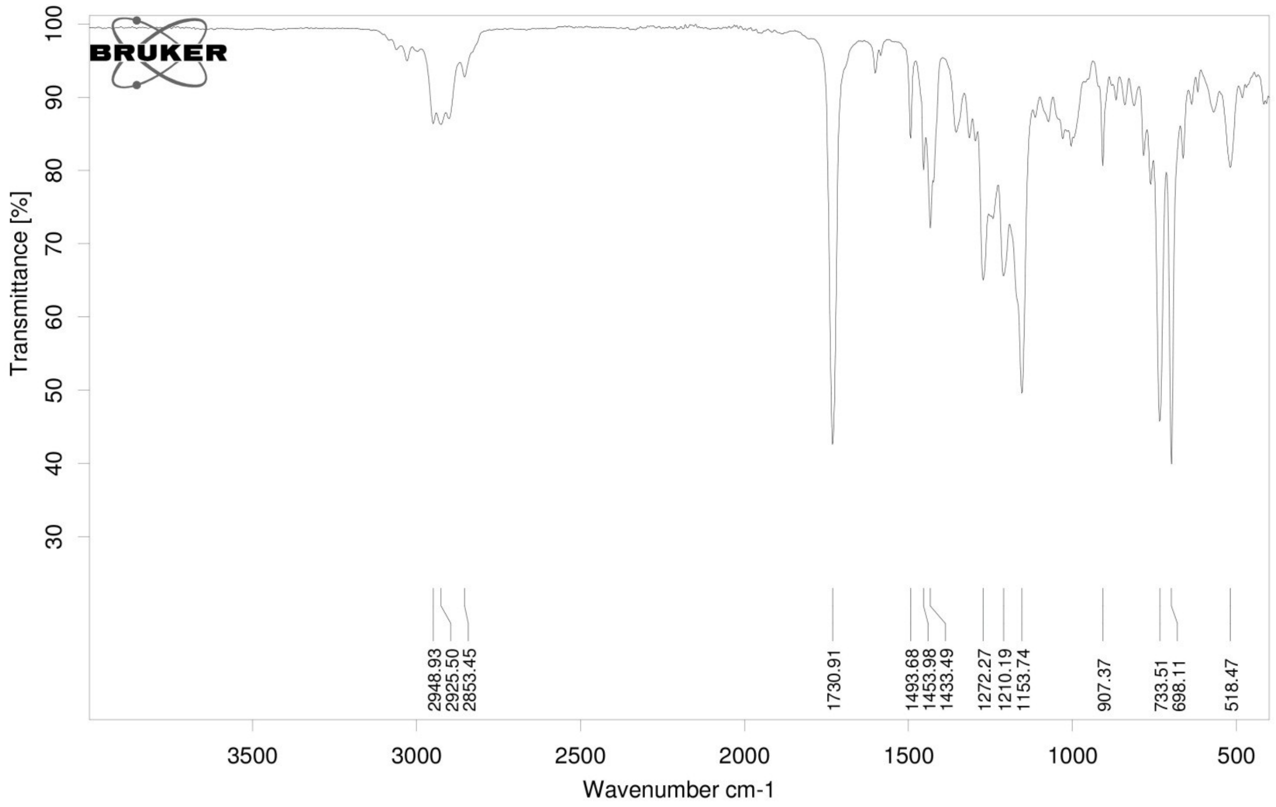
BRUKER
Transmittance [%]
100
90
80
70
60
50
40
30
2948.93
2925.50
2853.45
1730.91
1493.68
1453.98
1433.49
1272.27
1210.19
1153.74
907.37
733.51
698.11
518.47
3500
3000
2500
2000
1500
1000
500
Wavenumber cm-1

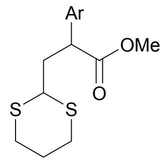

Ar = 4-Me-C_6H_4 **5k**

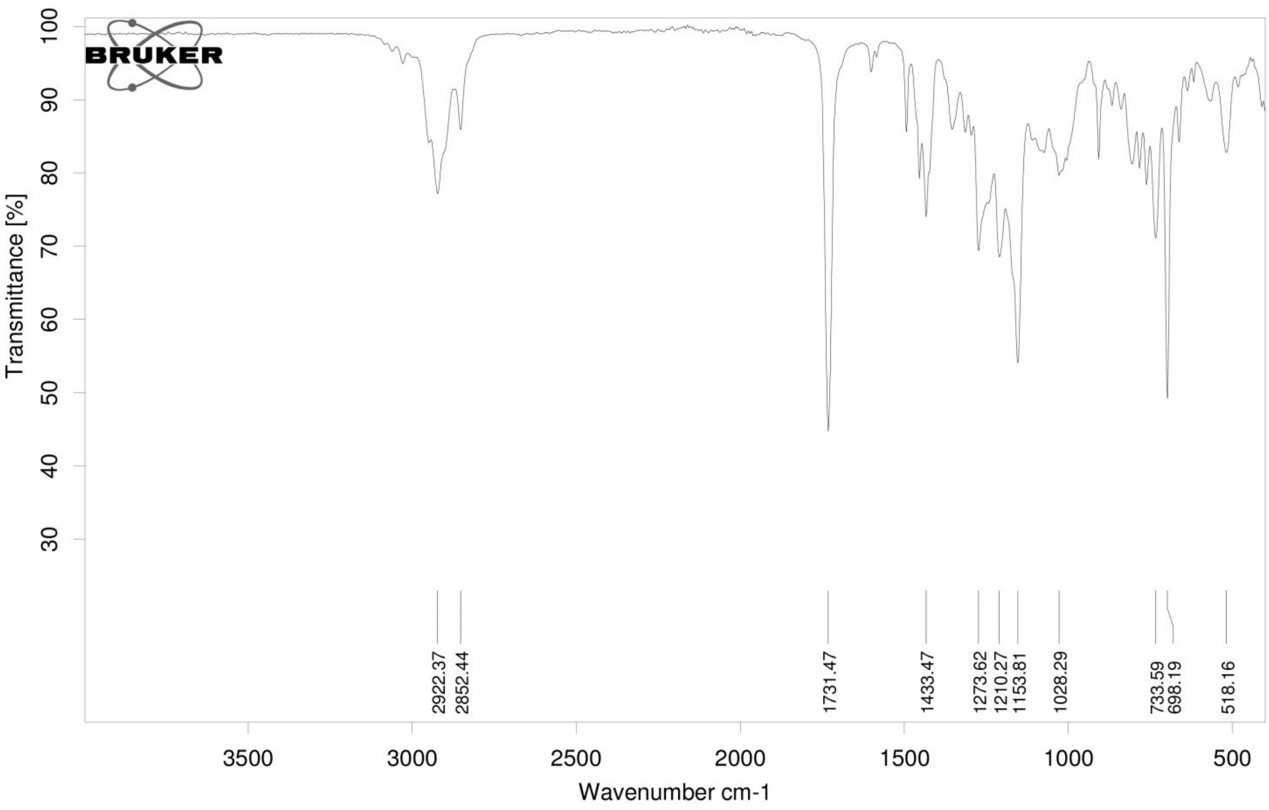

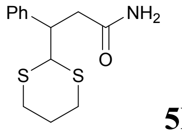
Ph
NH_2
O
S
S

5l

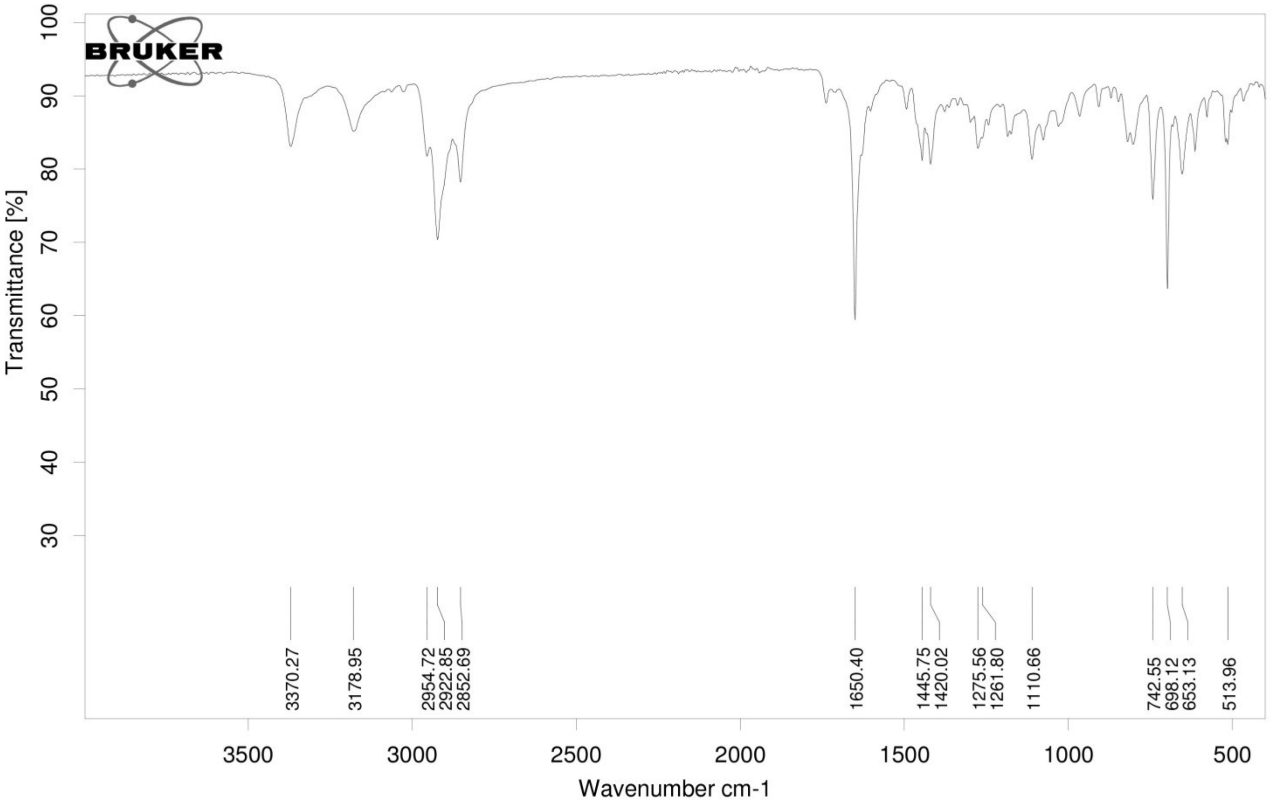
BRUKER
Transmittance [%]
100
90
80
70
60
50
40
30
3370.27
3178.95
2954.72
2922.85
2852.69
1650.40
1445.75
1420.02
1275.56
1261.80
1110.66
742.55
698.12
653.13
513.96
3500
3000
2500
2000
1500
1000
500
Wavenumber cm-1

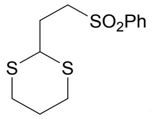
SO2Ph
S
S

5m

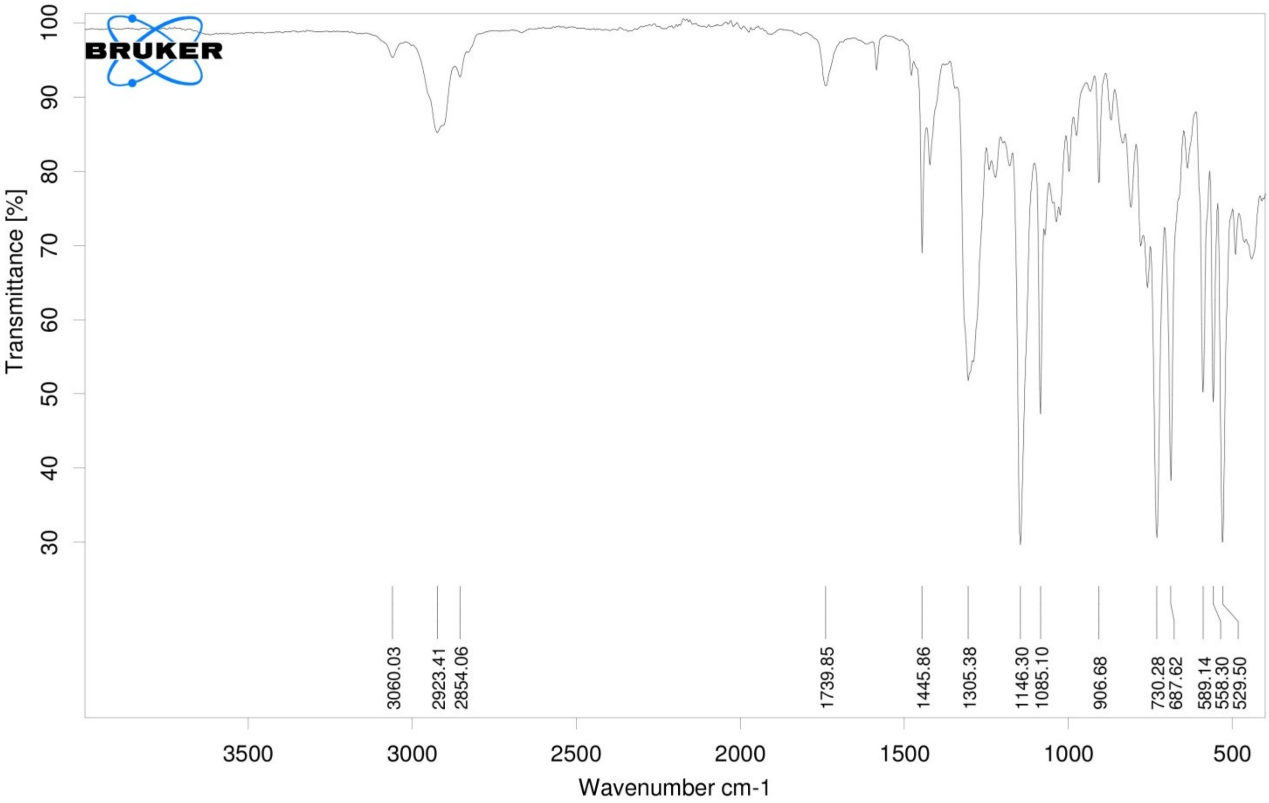
BRUKER
Transmittance [%]
Wavenumber cm-1
3060.03
2923.41
2854.06
1739.85
1445.86
1305.38
1146.30
1085.10
906.68
730.28
687.62
589.14
558.30
529.50

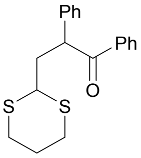

5n

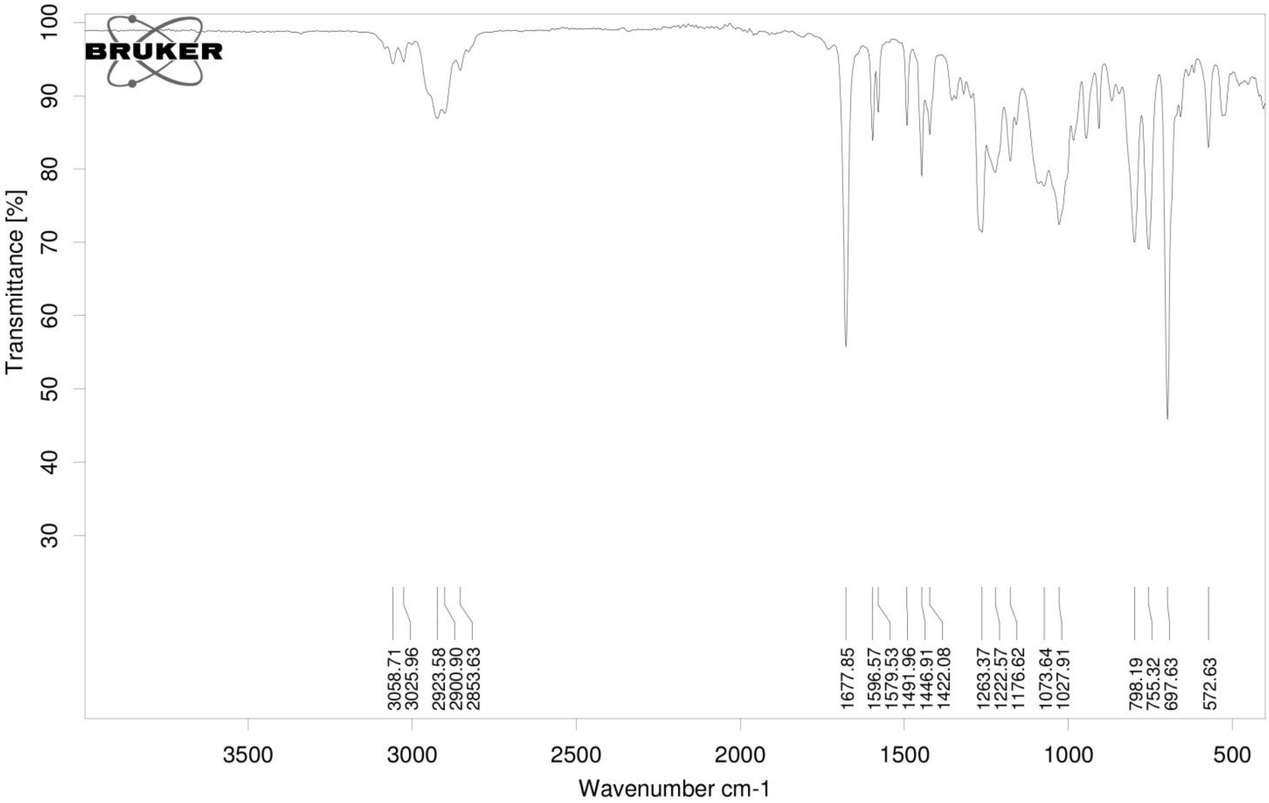
BRUKER
Transmittance [%]
Wavenumber cm-1
3058.71
3025.96
2923.58
2900.90
2853.63
1677.85
1596.57
1579.53
1491.96
1446.91
1422.08
1263.37
1222.57
1176.62
1073.64
1027.91
798.19
755.32
697.63
572.63

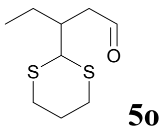

5o

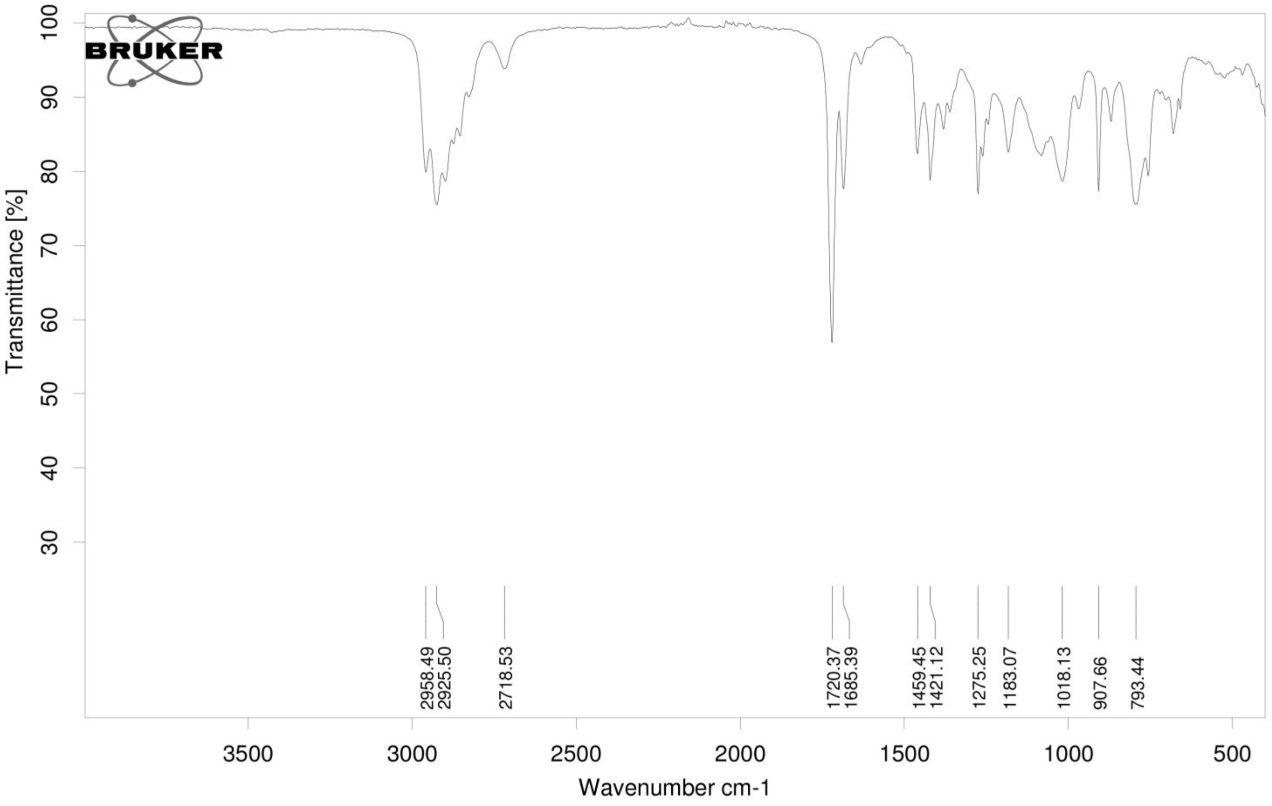

BRUKER
Transmittance [%]
100
90
80
70
60
50
40
30
2958.49
2925.50
2718.53
1720.37
1685.39
1459.45
1421.12
1275.25
1183.07
1018.13
907.66
793.44
3500
3000
2500
2000
1500
1000
500
Wavenumber cm-1

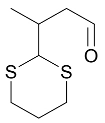
S
S
O

5p

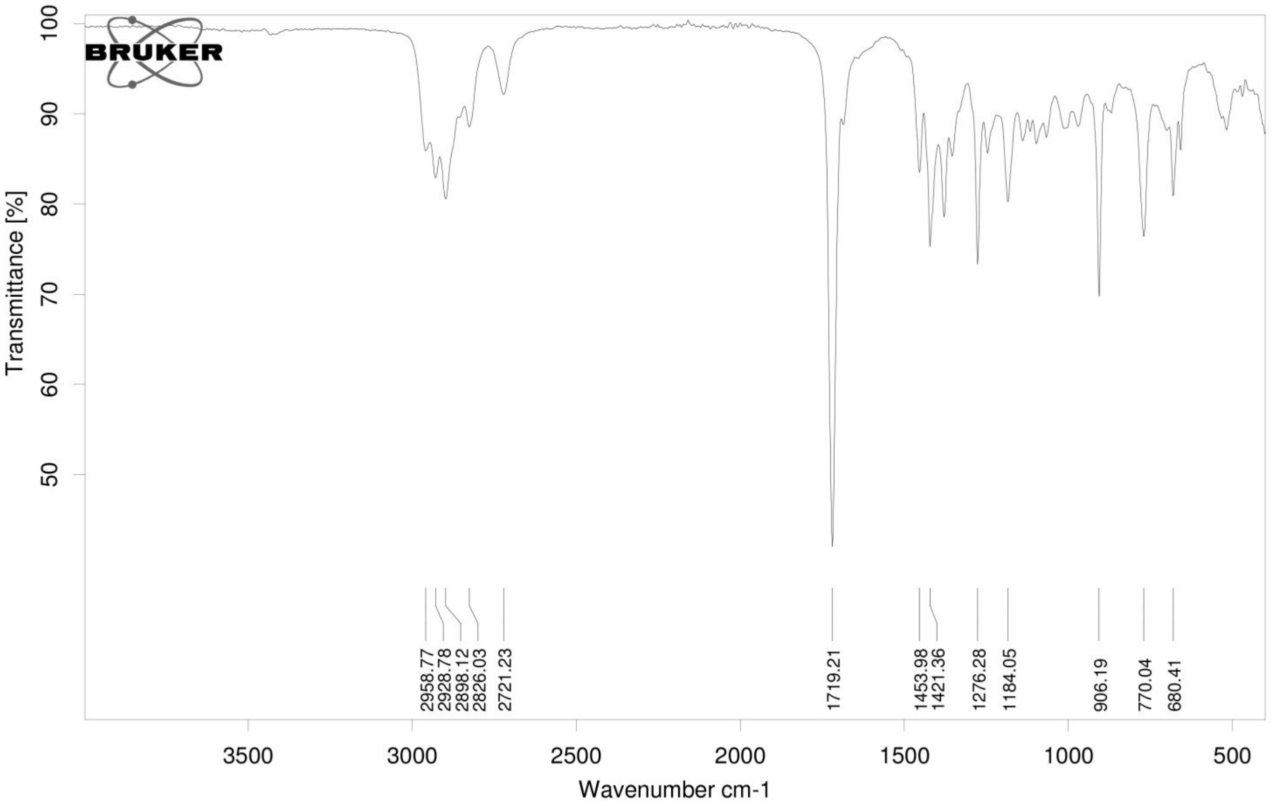
BRUKER
Transmittance [%]
100
90
80
70
60
50
2958.77
2928.78
2898.12
2826.03
2721.23
1719.21
1453.98
1421.36
1276.28
1184.05
906.19
770.04
680.41
3500
3000
2500
2000
1500
1000
500
Wavenumber cm-1

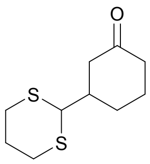
O
S
S

5q

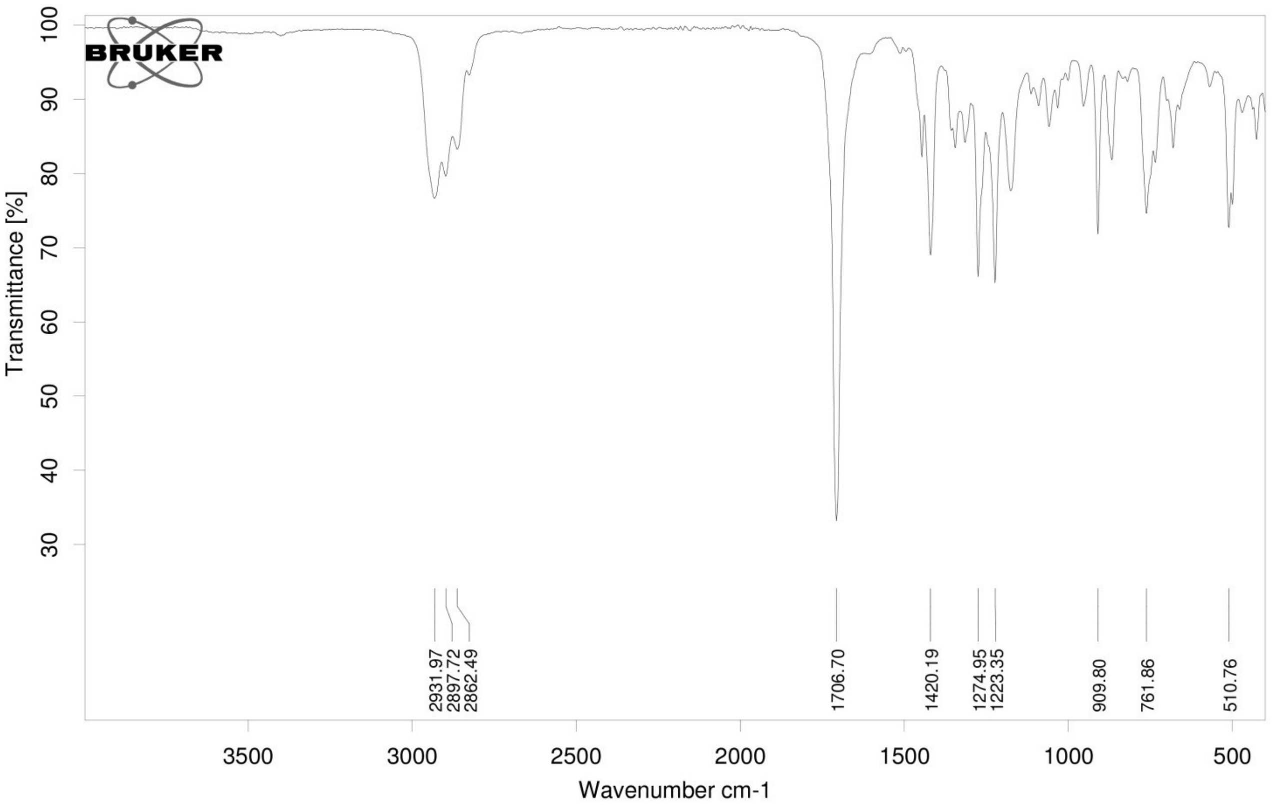
BRUKER
Transmittance [%]
100
90
80
70
60
50
40
30
2931.97
2897.72
2862.49
1706.70
1420.19
1274.95
1223.35
909.80
761.86
510.76
3500
3000
2500
2000
1500
1000
500
Wavenumber cm-1

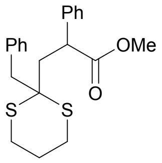
Ph
Ph
OMe
O
S
S

5r

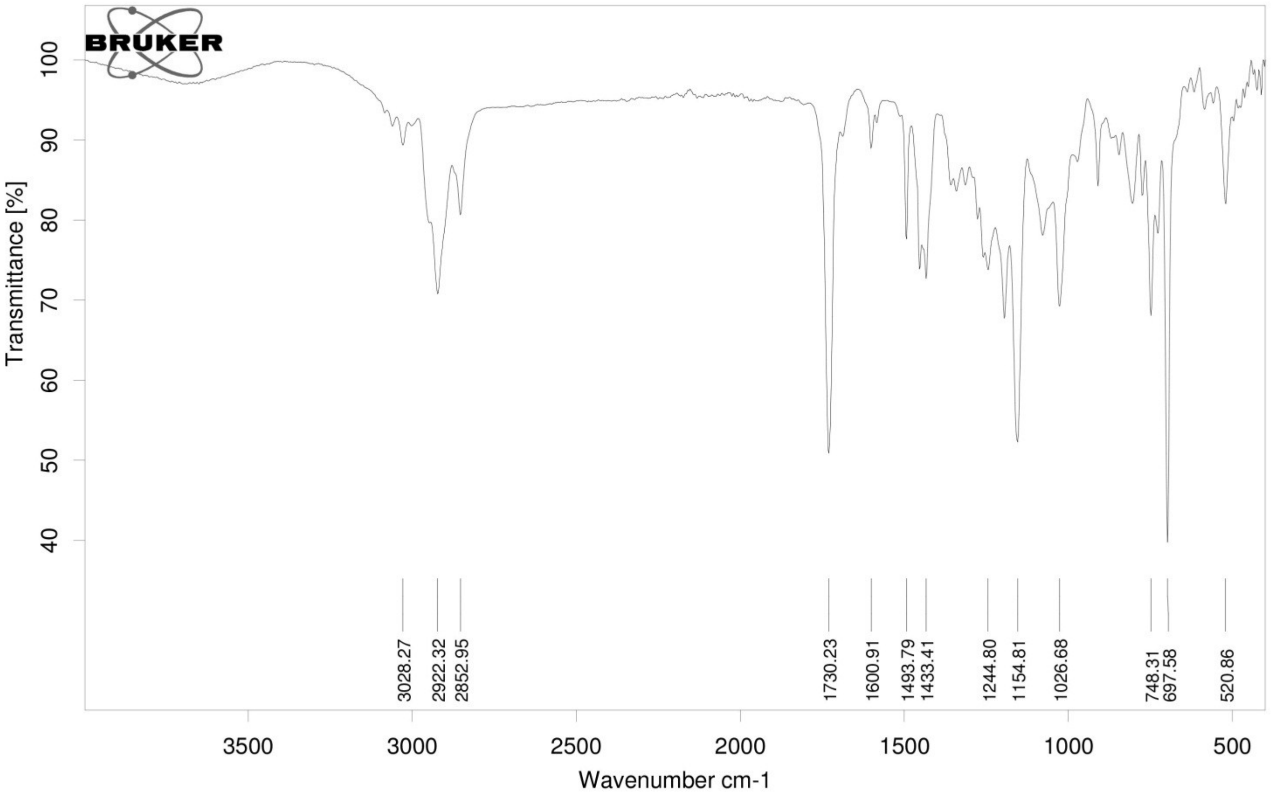
BRUKER
Transmittance [%]
100
90
80
70
60
50
40
3028.27
2922.32
2852.95
1730.23
1600.91
1493.79
1433.41
1244.80
1154.81
1026.68
748.31
697.58
520.86
3500
3000
2500
2000
1500
1000
500
Wavenumber cm-1

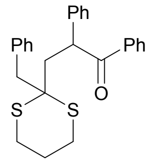
Ph
Ph
Ph
O
S
S

5s

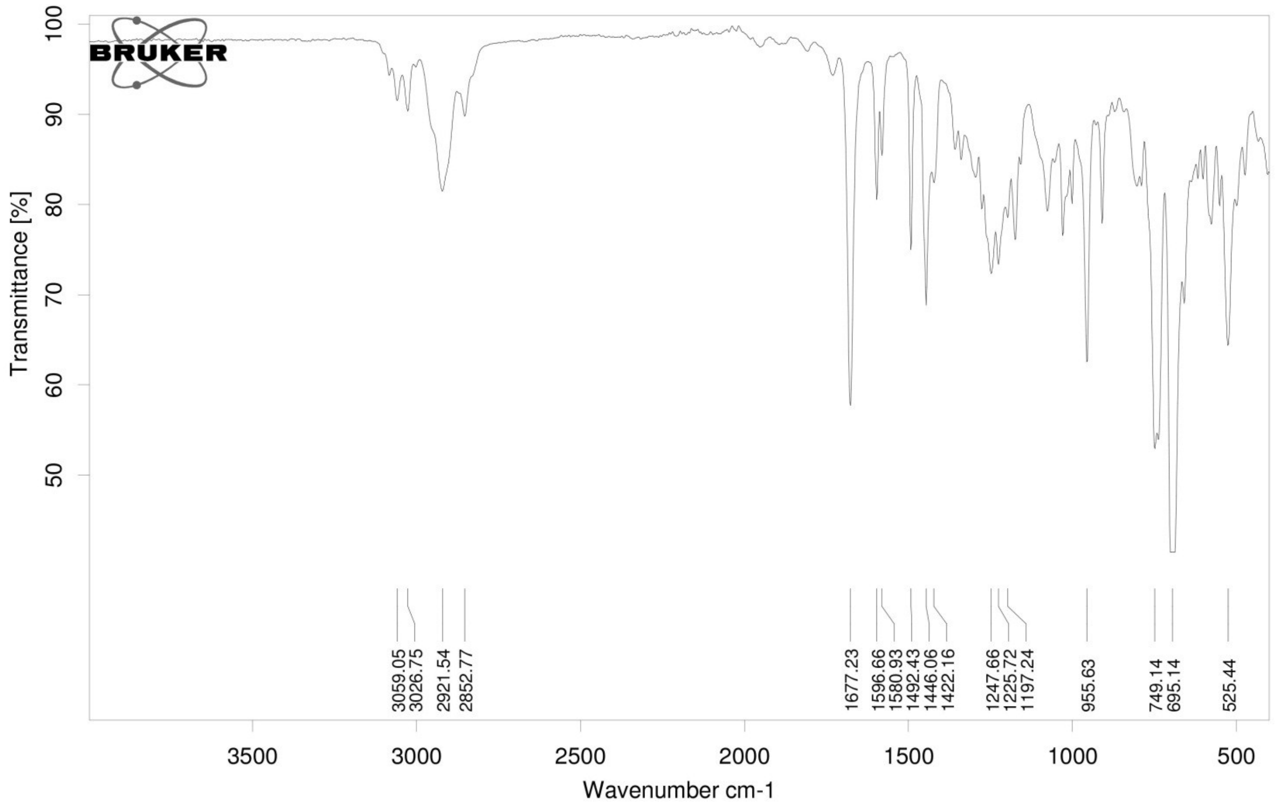
BRUKER
Transmittance [%]
100
90
80
70
60
50
3059.05
3026.75
2921.54
2852.77
1677.23
1596.66
1580.93
1492.43
1446.06
1422.16
1247.66
1225.72
1197.24
955.63
749.14
695.14
525.44
3500
3000
2500
2000
1500
1000
500
Wavenumber cm-1

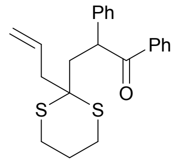

5t

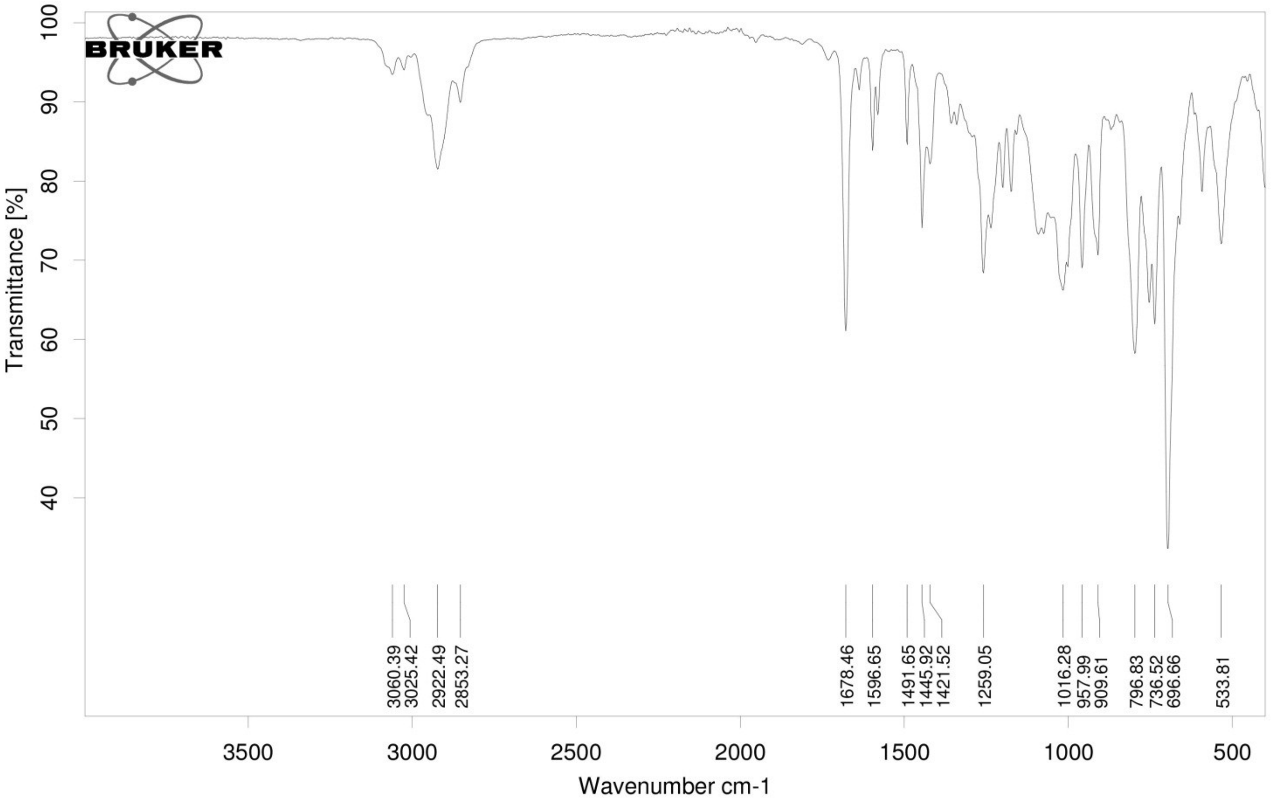
BRUKER
Transmittance [%]
Wavenumber cm-1
3060.39
3025.42
2922.49
2853.27
1678.46
1596.65
1491.65
1445.92
1421.52
1259.05
1016.28
957.99
909.61
796.83
736.52
696.66
533.81

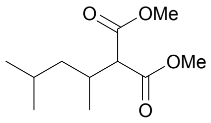
O
OMe
OMe
O

6

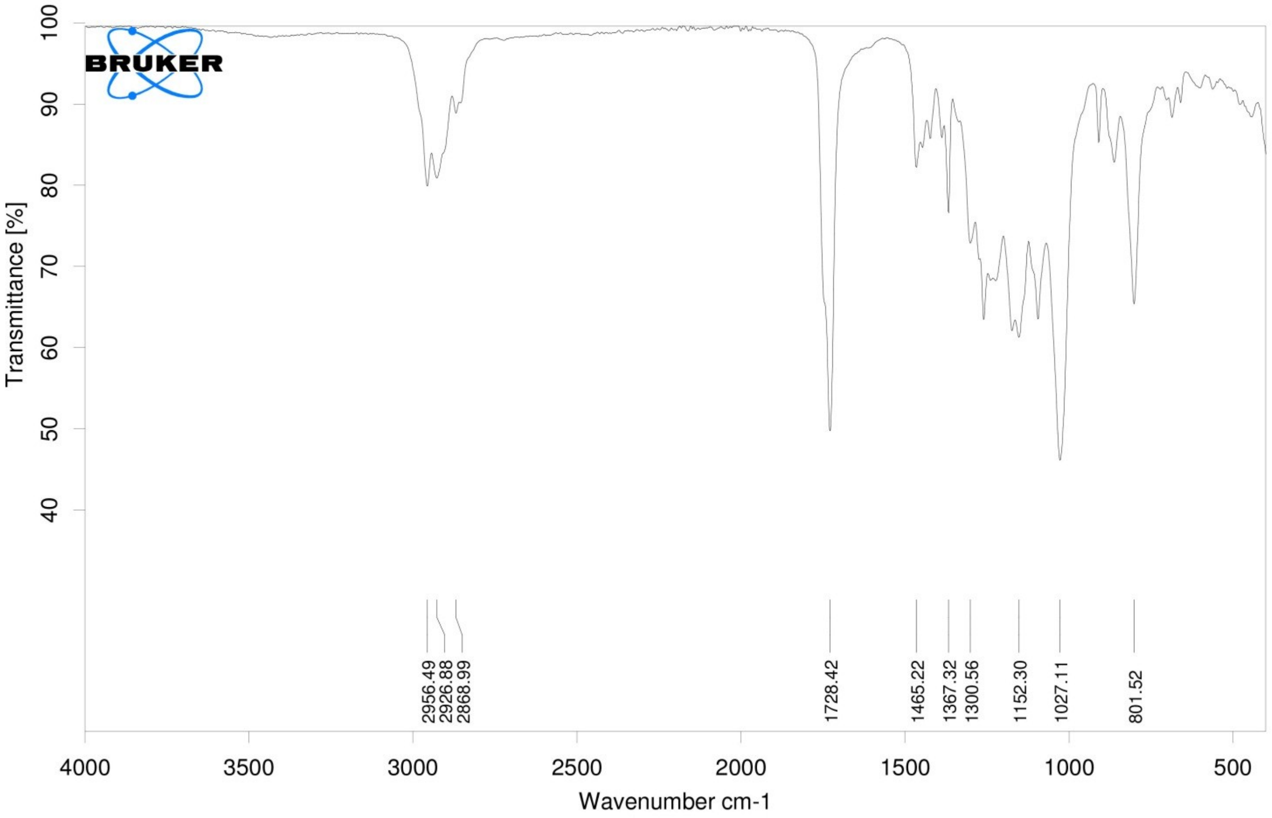
BRUKER
Transmittance [%]
100
90
80
70
60
50
40
2956.49
2926.88
2868.99
1728.42
1465.22
1367.32
1300.56
1152.30
1027.11
801.52
4000
3500
3000
2500
2000
1500
1000
500
Wavenumber cm-1

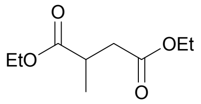

7

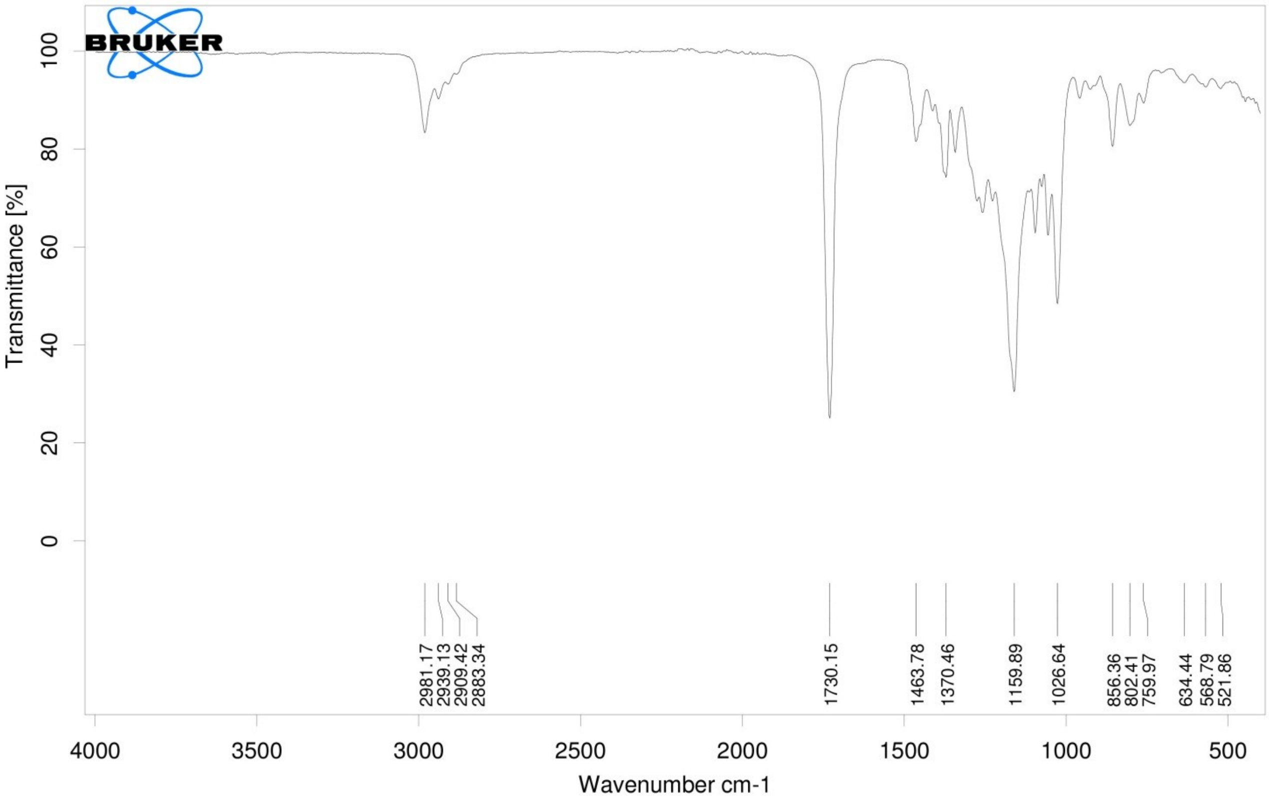

BRUKER
Transmittance [%]
100
80
60
40
20
0
2981.17
2939.13
2909.42
2883.34
1730.15
1463.78
1370.46
1159.89
1026.64
856.36
802.41
759.97
634.44
568.79
521.86
4000
3500
3000
2500
2000
1500
1000
500
Wavenumber cm-1

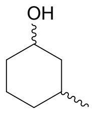
OH

8

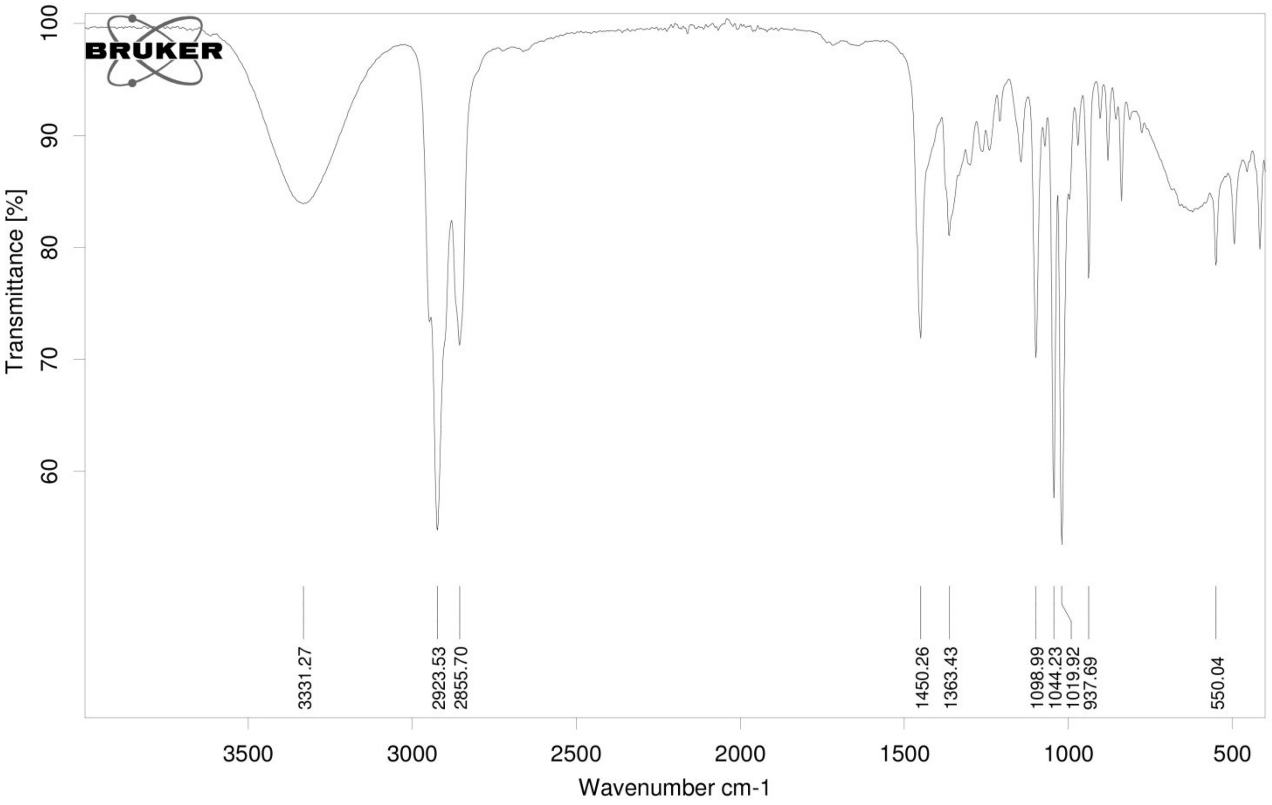
Transmittance [%]
100
90
80
70
60
3331.27
2923.53
2855.70
1450.26
1363.43
1098.99
1044.23
1019.92
937.69
550.04
3500
3000
2500
2000
1500
1000
500
Wavenumber cm-1

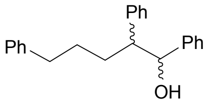
Ph
Ph
Ph
OH

9

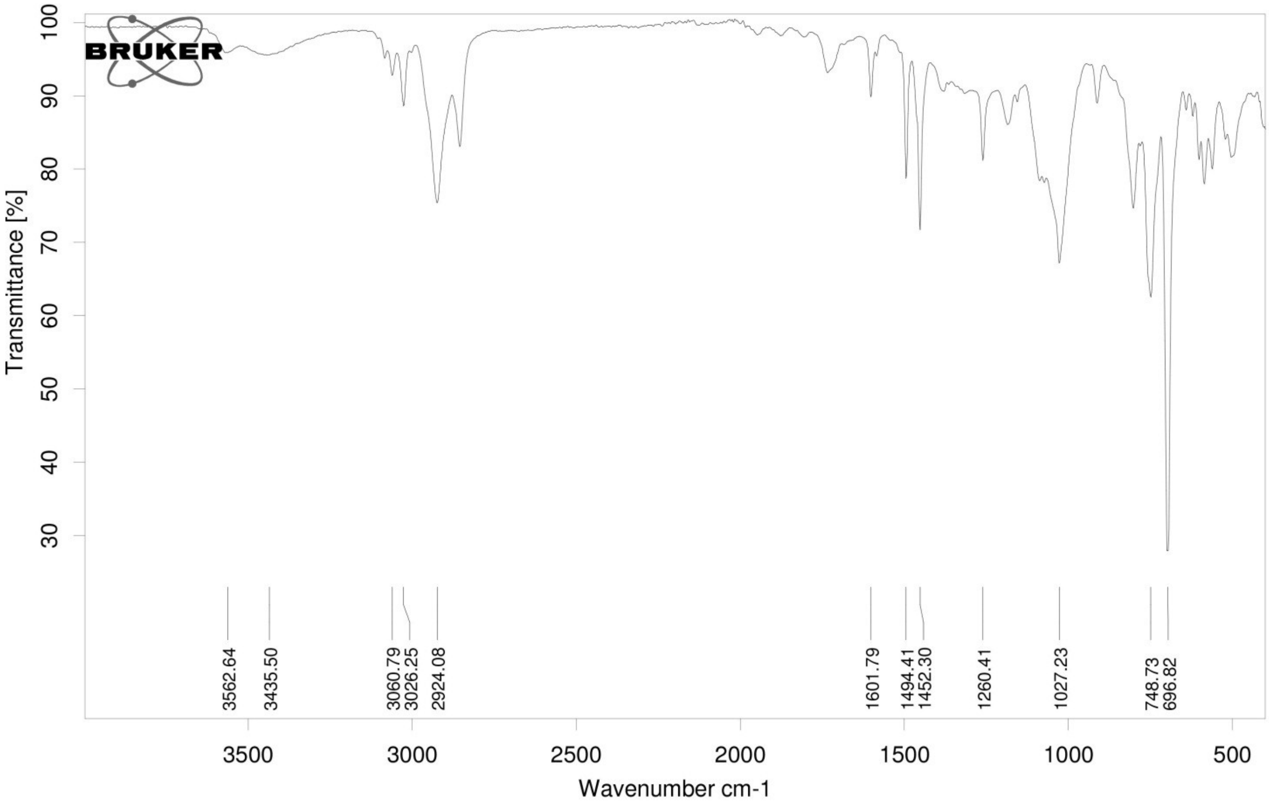
BRUKER
Transmittance [%]
100
90
80
70
60
50
40
30
3562.64
3435.50
3060.79
3026.25
2924.08
1601.79
1494.41
1452.30
1260.41
1027.23
748.73
696.82
3500
3000
2500
2000
1500
1000
500
Wavenumber cm-1

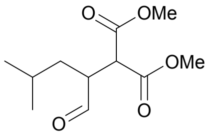

OMe
OMe
O
O
O

10

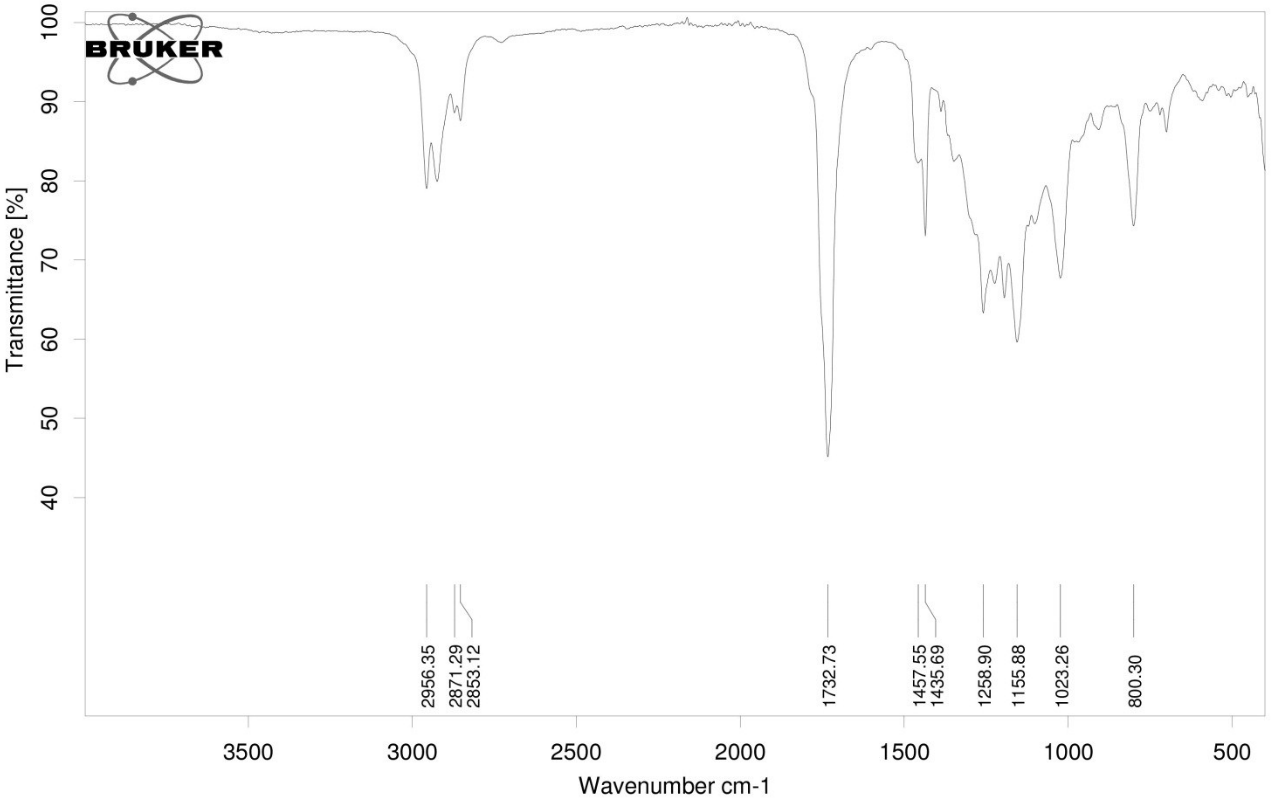

BRUKER
Transmittance [%]
100
90
80
70
60
50
40
2956.35
2871.29
2853.12
1732.73
1457.55
1435.69
1258.90
1155.88
1023.26
800.30
3500
3000
2500
2000
1500
1000
500
Wavenumber cm-1

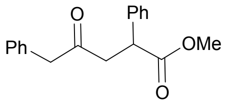

11

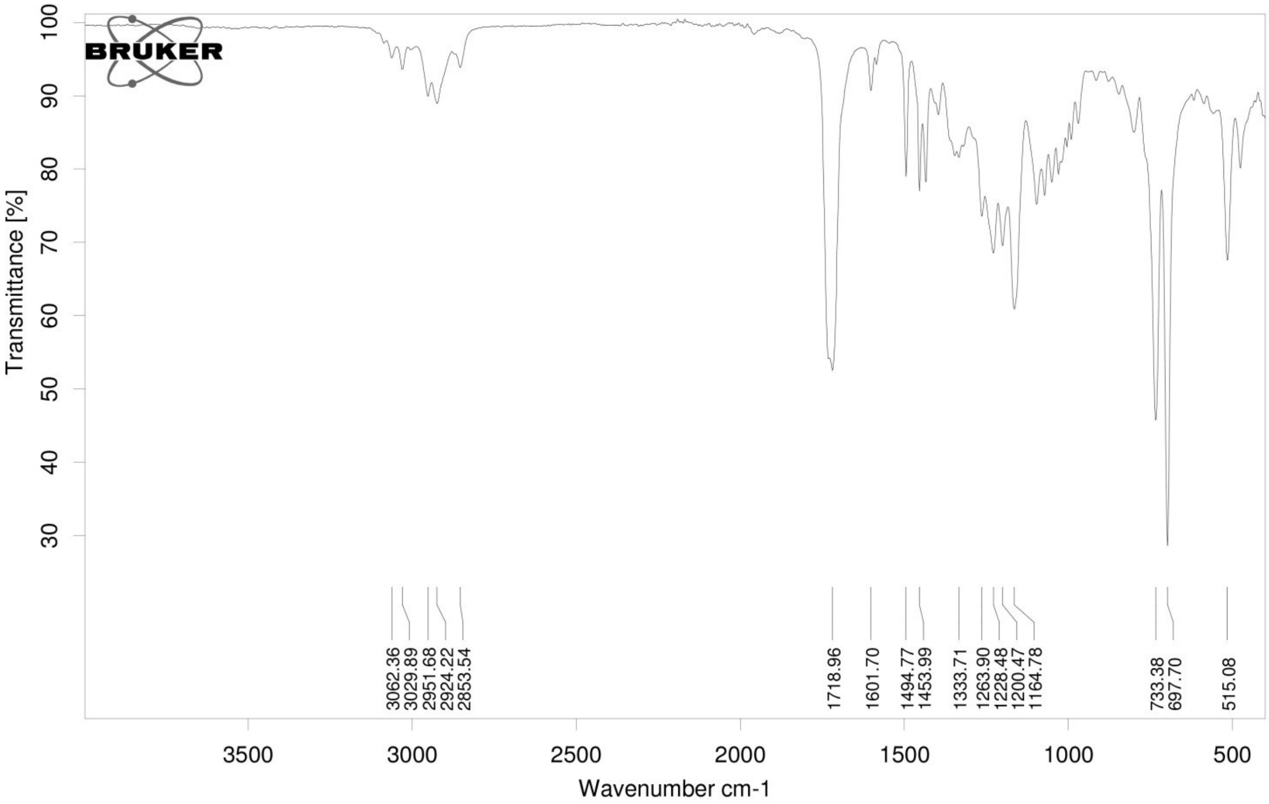
BRUKER
Transmittance [%]
100
90
80
70
60
50
40
30
3062.36
3029.89
2951.68
2924.22
2853.54
1718.96
1601.70
1494.77
1453.99
1333.71
1263.90
1228.48
1200.47
1164.78
733.38
697.70
515.08
3500
3000
2500
2000
1500
1000
500
Wavenumber cm-1

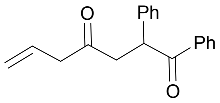

12

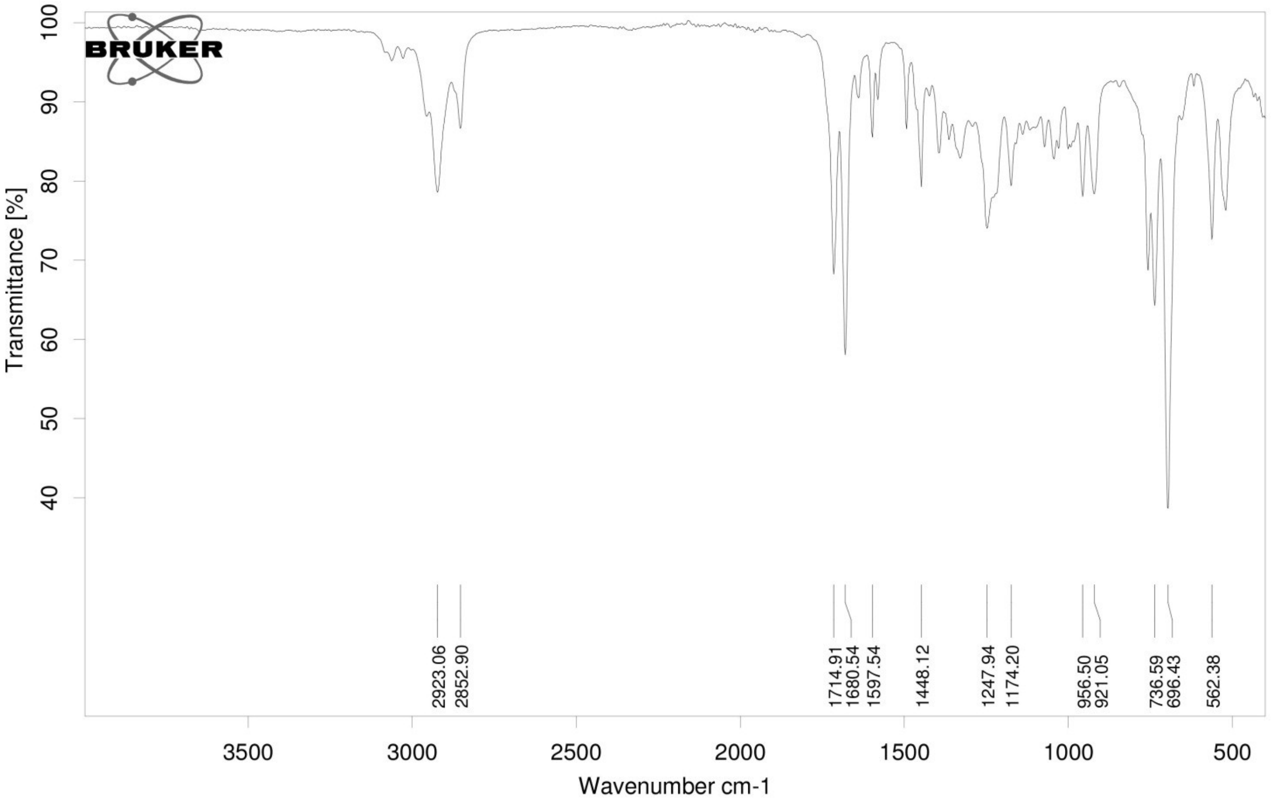

BRUKER
Transmittance [%]
100
90
80
70
60
50
40
2923.06
2852.90
1714.91
1680.54
1597.54
1448.12
1247.94
1174.20
956.50
921.05
736.59
696.43
562.38
3500
3000
2500
2000
1500
1000
500
Wavenumber cm-1

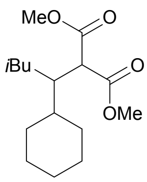
MeO
O
iBu
O
OMe

14

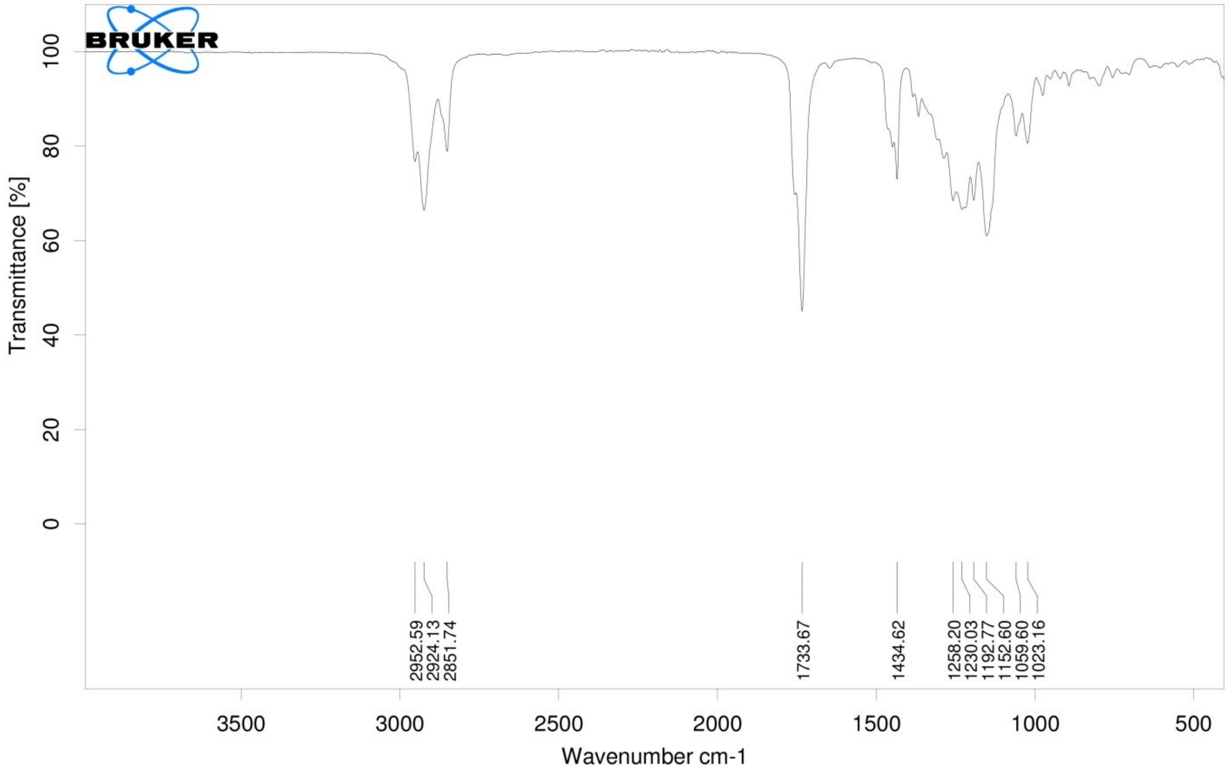
BRUKER
Transmittance [%]
Wavenumber cm-1
2952.59
2924.13
2851.74
1733.67
1434.62
1258.20
1230.03
1192.77
1152.60
1059.60
1023.16

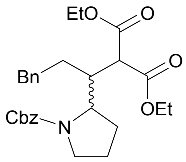
EtO
O
O
Bn
OEt
Cbz
N

16

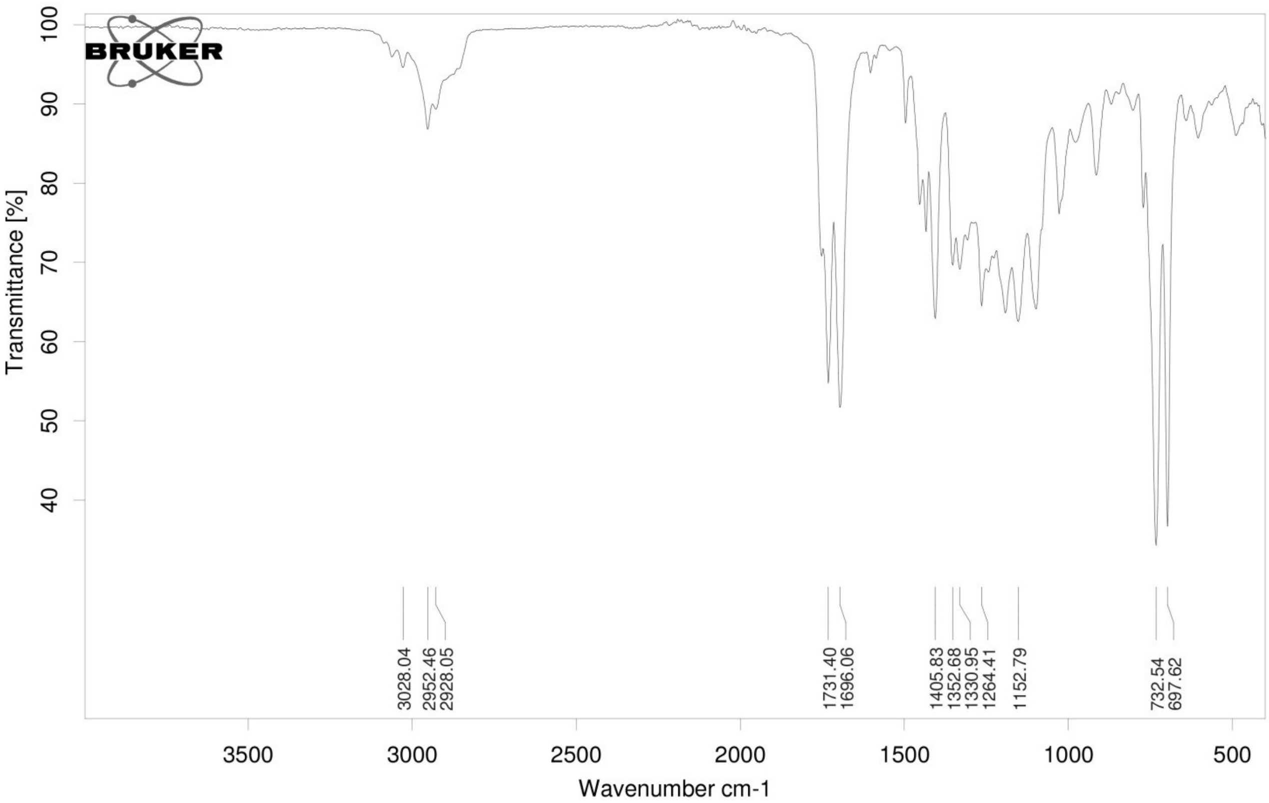
BRUKER
Transmittance [%]
100
90
80
70
60
50
40
3028.04
2952.46
2928.05
1731.40
1696.06
1405.83
1352.68
1330.95
1264.41
1152.79
732.54
697.62
3500
3000
2500
2000
1500
1000
500
Wavenumber cm-1

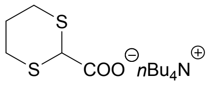
S
S
COO
nBu4N

17

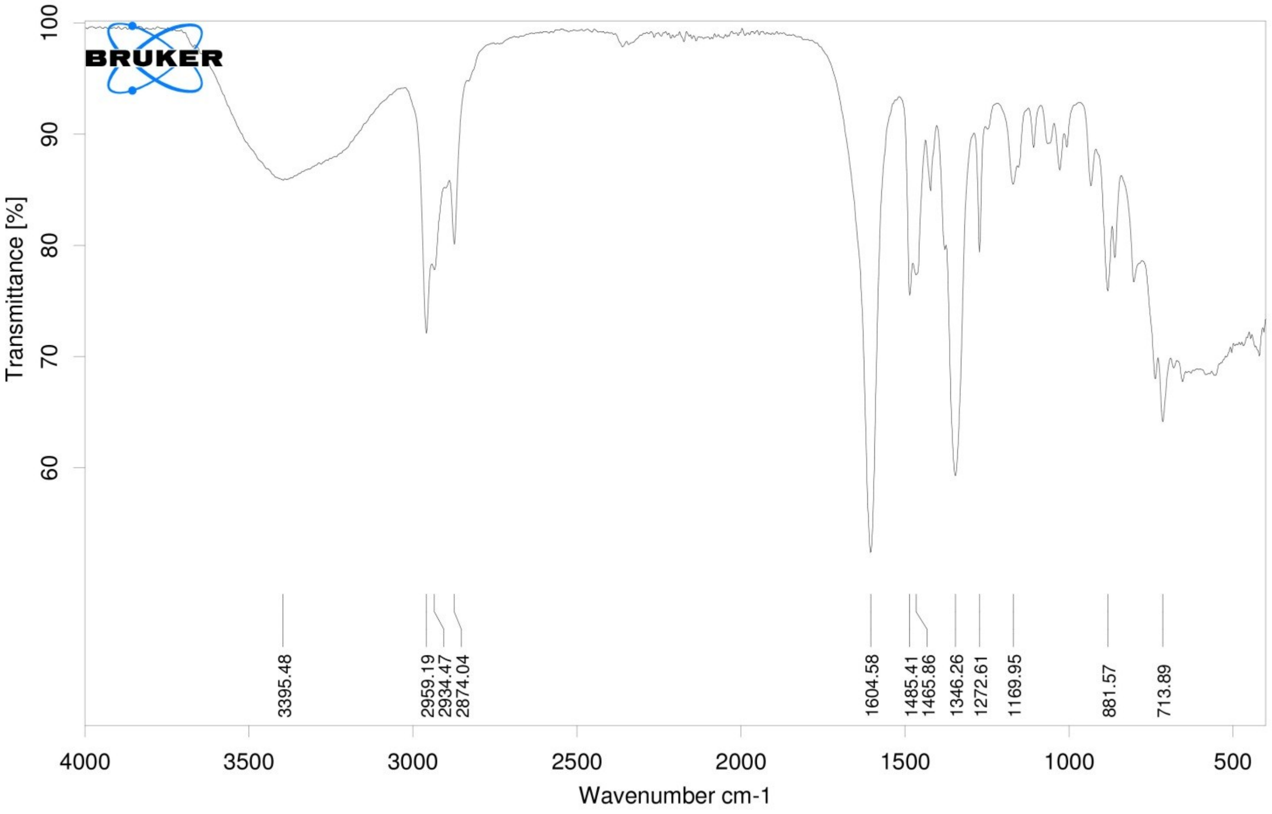
BRUKER
Transmittance [%]
100
90
80
70
60
3395.48
2959.19
2934.47
2874.04
1604.58
1485.41
1465.86
1346.26
1272.61
1169.95
881.57
713.89
4000
3500
3000
2500
2000
1500
1000
500
Wavenumber cm-1

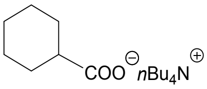
COO⊖ nBu4N⊕

18

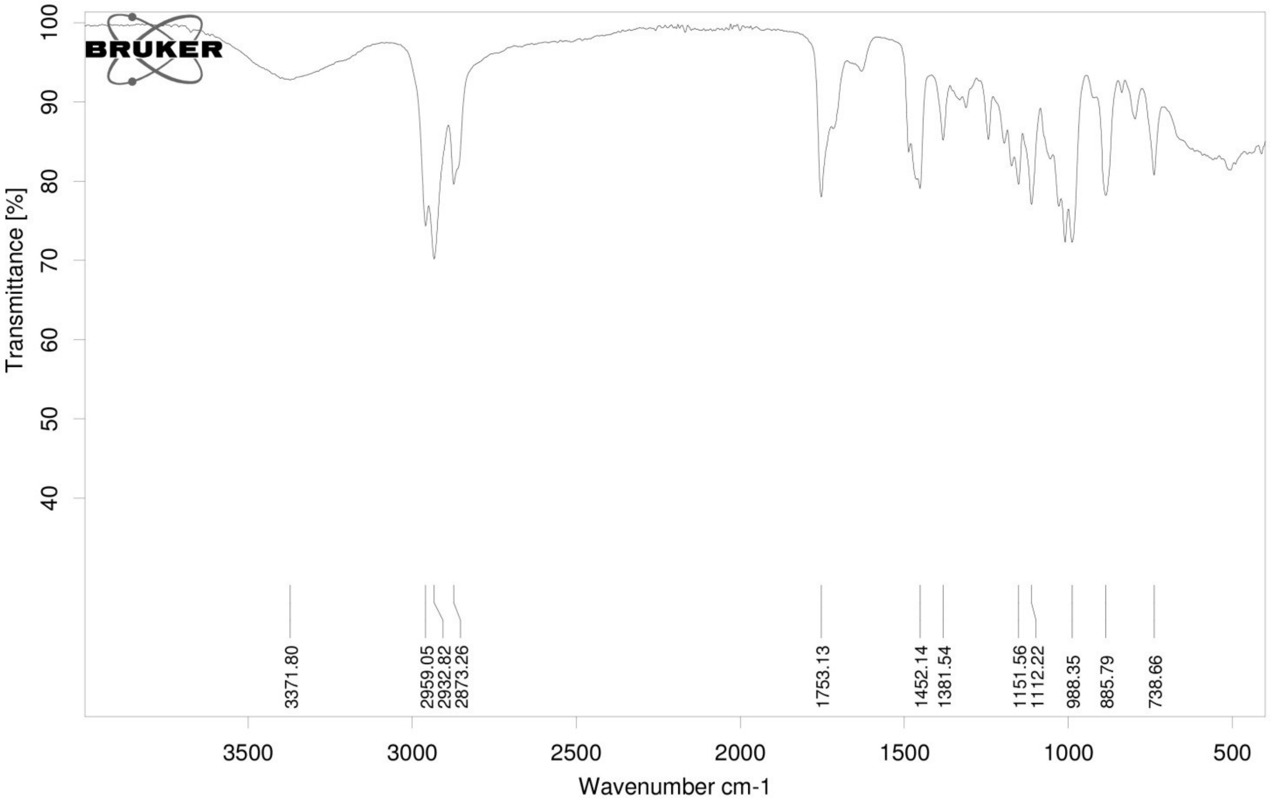
BRUKER
Transmittance [%]
100
90
80
70
60
50
40
3371.80
2959.05
2932.82
2873.26
1753.13
1452.14
1381.54
1151.56
1112.22
988.35
885.79
738.66
3500
3000
2500
2000
1500
1000
500
Wavenumber cm-1

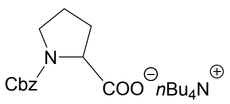
N
Cbz
COO
nBu4N

19

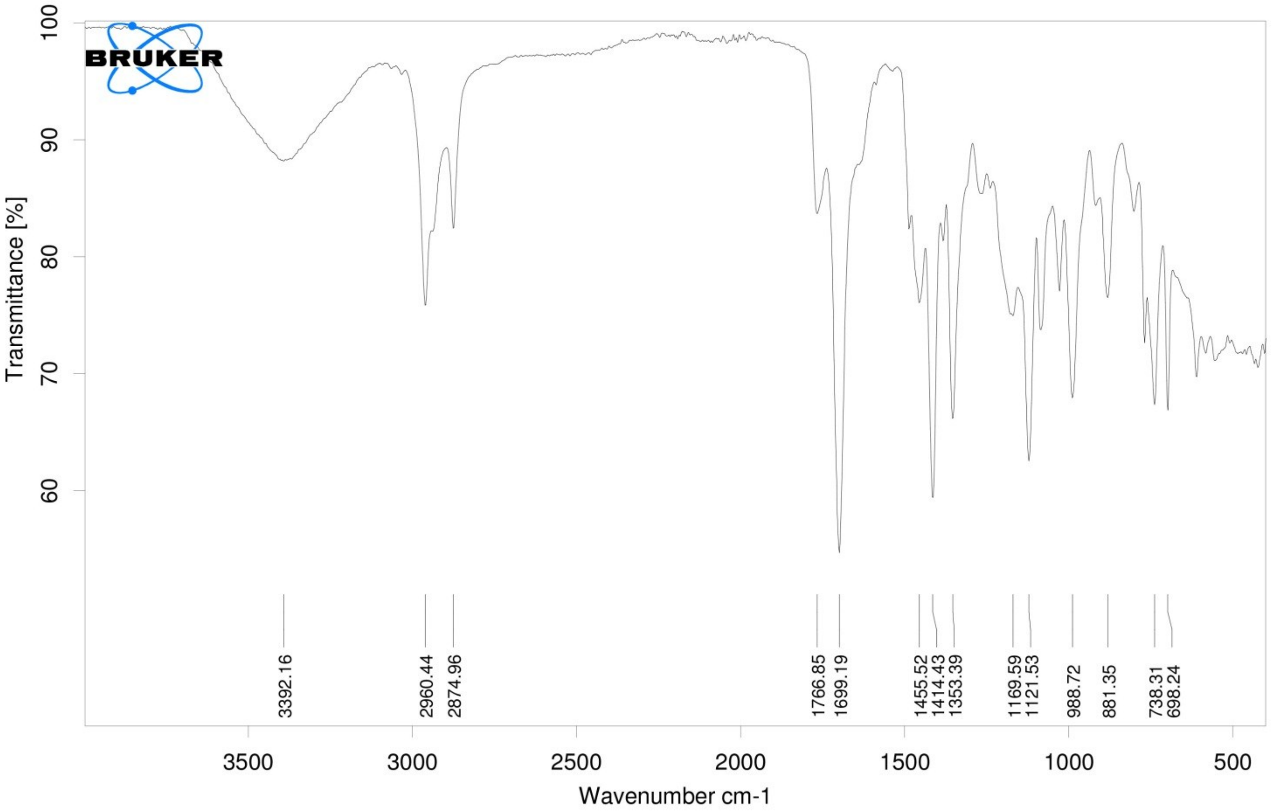
BRUKER
Transmittance [%]
100
90
80
70
60
3392.16
2960.44
2874.96
1766.85
1699.19
1455.52
1414.43
1353.39
1169.59
1121.53
988.72
881.35
738.31
698.24
3500
3000
2500
2000
1500
1000
500
Wavenumber cm-1
